# Selective Synthesis of Boron-Functionalized Indenes and Benzofulvenes by BCl_3_-Promoted Cyclizations of *ortho*-Alkynylstyrenes

**DOI:** 10.1021/acs.orglett.4c02092

**Published:** 2024-07-29

**Authors:** Marcos Humanes, Ester Sans-Panadés, Cintia Virumbrales, Ana Milián, Roberto Sanz, Patricia García-García, Manuel A. Fernández-Rodríguez

**Affiliations:** † Departamento de Química Orgánica y Química Inorgánica, Instituto de Investigación Química “Andrés M. del Río” (IQAR), Universidad de Alcalá (IRYCIS), 28805 Alcalá de Henares, Madrid, Spain; ‡ Área de Química Orgánica, Departamento de Química, Facultad de Ciencias, Universidad de Burgos, Pza. Misael Bañuelos s/n, 09001 Burgos, Spain

## Abstract

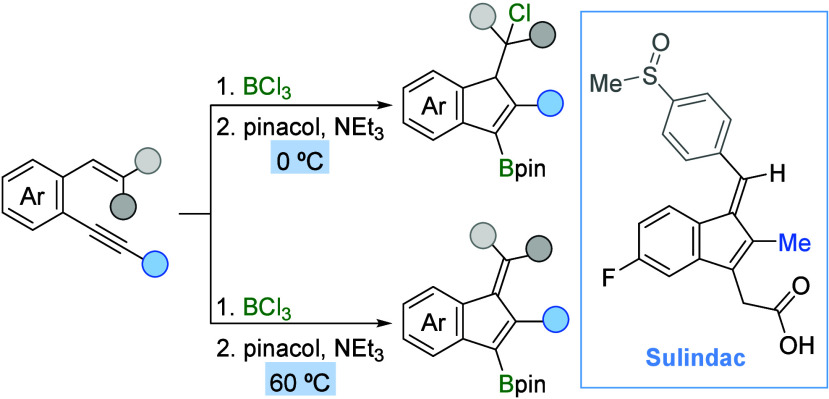

A selective, metal-free synthesis of boron-functionalized indenes and benzofulvenes via BCl_3_-mediated cyclization of *o*-alkynylstyrenes is described. The method allows precise control over product formation by adjusting reaction conditions. These borylated products were utilized in diverse C–B bond derivatizations and in the total synthesis of Sulindac, a nonsteroidal anti-inflammatory drug, demonstrating the versatility and practicality of the developed methodology for synthetic applications.

Compounds featuring indene or benzofulvene cores have attracted significant attention due to their applications in materials science,^1^ their roles as ligands in metal complexes,^2^ and their frequent occurrence in natural products, biologically active molecules and pharmaceuticals (Scheme 1a).^3^ A representative example is Sulindac, a nonsteroidal anti-inflammatory agent (NSAID) with a benzofulvene skeleton, used for treating pain and inflammation associated with arthritis, gout and other inflammatory diseases.^3e,3f^ Numerous synthetic approaches for indenes and benzofulvenes have been described,^4^ including electrophilic cyclizations of *o*-alkynylstyrenes promoted by iodonium sources or catalyzed by gold(I) complexes or B(C_6_F_5_)_3_/PPh_3_ frustrated Lewis pair (Scheme 1b).^5^ However, the development of more sustainable, ideally metal-free, strategies for synthesizing easily fuctionalizable derivatives of these privileged scaffolds remains of great importance.

**Scheme 1:**
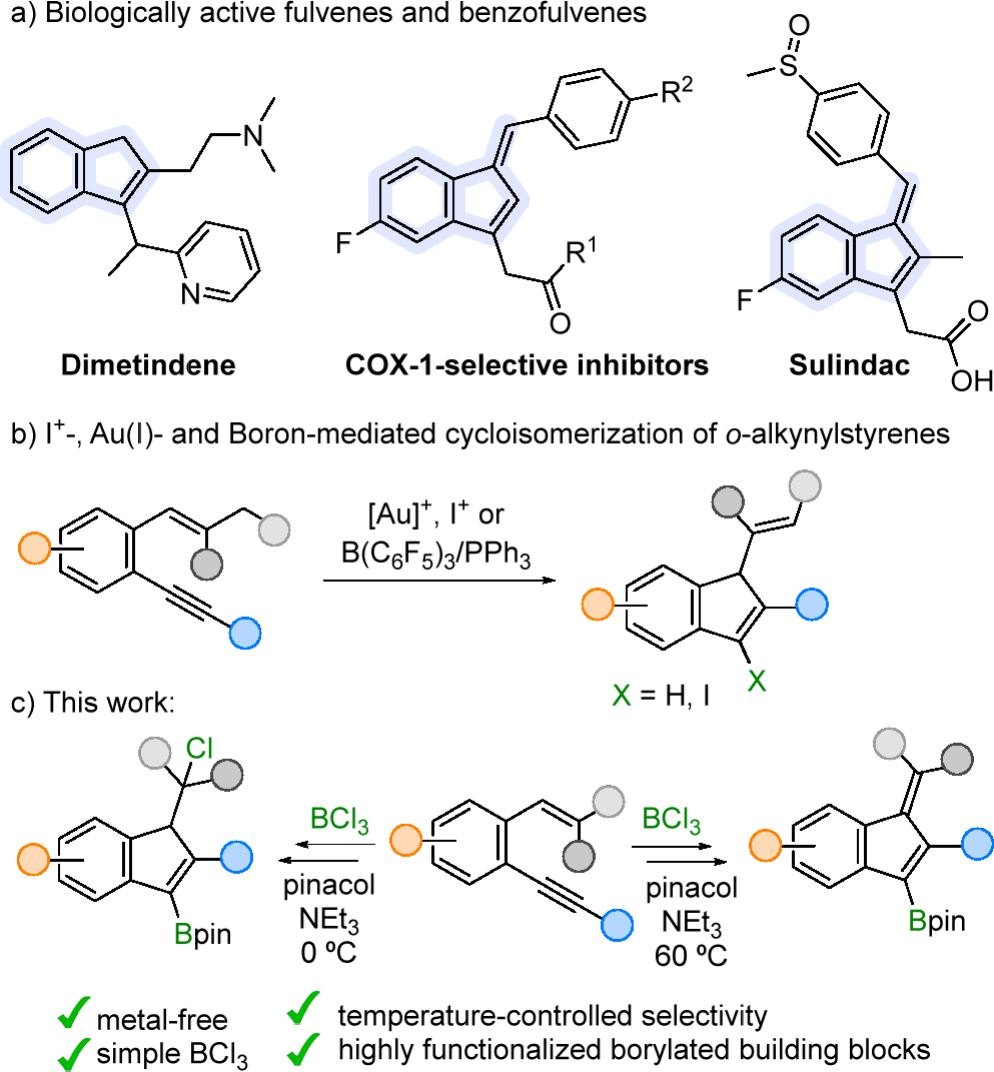
Background and Proposal

The formation of C–B bonds is crucial in organic chemistry, given the great utility of boron-containing organic compounds in areas such as materials science and medicinal chemistry,^6^ as well as synthetic intermediates. Boronic acids and their ester derivatives, known for their stability, ease of storage and handling, high functional group tolerance, and low toxicity, serve as essential and versatile building blocks in several chemical transformations.^7^

Traditionally, the synthesis of organoboronates involves the use of organometallic reagents and/or transition metal complexes.^8^ These conventional methods often suffer from limitations such as functional group incompatibility, the necessity for harsh reaction conditions or prefunctionalized substrates, and the constraints imposed by the intrinsic selectivity of the starting materials. In the past decade, metal-free C–B bond formation strategies have emerged as more sustainable and practical alternatives, overcoming many of these issues. In this context, borylative heterocyclizations provide a straightforward and regioselective access to heterocycles bearing easily functionalizable boron substituents in their structure.^8a,8h,9^ This innovative methodology simultaneously forms a heterocycle and a C–B bond via an intramolecular cyclization triggered by a nucleophilic attack on a triple bond activated by a boron electrophile. Ingleson developed the analogous carbocyclizations to synthesize B-containing dihydronaphtalenes, using alkynyl substrates with an arene acting as an internal nucleophile and simple BCl_3_ as activator.^10^ This method was later applied to deliver B- and B,N-doped polycyclic aromatic hydrocarbons.^11^ The same group reported the formation of various borylated cycloadducts, including benzofulvenes, in an interesting study on the cyclization of diynes that provides useful mechanistic insights but shows limited synthetic application.^12^ Recently, the first synthetically useful metal-free borylative enyne cyclization has been reported, using BCl_3_ as promoter and *o*-alkenyl-*o*′-alkynylbiaryls as substrates, producing borylated phenathrenes through a notable skeletal rearrangement.^13^

Herein, we describe the selective synthesis of boron-functionalized indenes and benzofulvenes via a metal-free BCl_3_-mediated cyclization of suitable *o*-alkynylstyrenes (Scheme 1c). Additionally, the utility of the synthesized borylated indenes and the developed metal-free methodology has been demonstrated by a range of C–B bond derivatizations and the total synthesis of Sulindac.

At first, we selected 2′,2′-dimethyl *o*-(phenylethynyl)styrene **1a** as a model substrate and tested its cyclization in the presence of BCl_3_ (Scheme 2). Initial experiments using different solvents, temperatures and reaction times led, upon treatment with pinacol and NEt_3_, to mixtures of products among which variable amounts of indene **2a** and benzofulvene **4a** were observed (see Supporting Information for details). After some optimization, selective cyclization to **2a**-BCl_2_ was achieved when the reaction was performed at 0 °C using dichloromethane (DCM) as solvent. Remarkably, the conditions subsequently used for the formation of the pinacol ester determined the nature of the product finally obtained. Thus, when it was carried out at 0 °C in short reaction times indene **2a** was selectively formed, whereas heating at 60 °C and using prolonged reaction times allowed the exclusive formation of benzofulvene **3a**, coming from the elimination of HCl from the initial cyclization product. Compound **2a** proved to be unstable during purification by column chromatography with silica gel, leading to **4a**. However, **2a** could be isolated in good yield by using deactivated silica gel for the purification. Moreover, an increased yield was achieved for the bulkier Bepin derivative.^14^ The formation of **2a**-BCl_2_ is proposed to proceed via activation of the alkyne by coordination to BCl_3_ followed by nucleophilic attack of the alkene moiety promoting a 5-*endo-dig* cyclization. The carbocationic intermediate would then be trapped by a chloride anion. Some experiments were performed to unveil the origin of this chloride. Thus, compound **2a** was also obtained using chlorobenzene as the solvent, which is more robust to halide loss than DCM. In addition, indene **5a**, incorporating a bromine atom, was exclusively formed using BBr_3_ instead of BCl_3_. Therefore, both experiments suggest that the halogen atom present in the final product comes from the boron source.

**Scheme 2:**
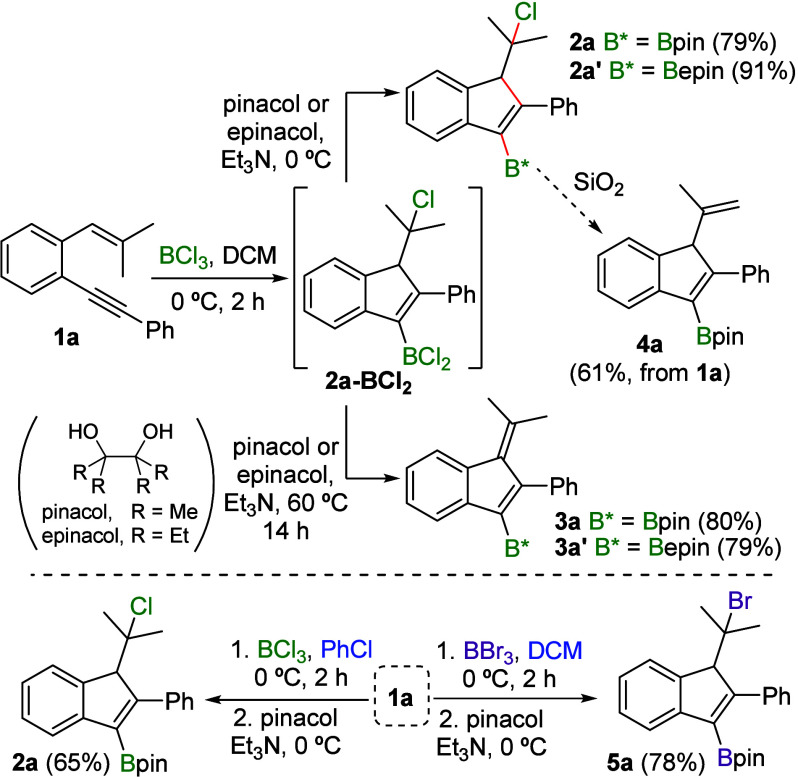
Initial Experiments

Once optimal conditions for the selective synthesis of the desired cycloadducts **2a** or **3a** had been established, we explored the scope of both transformations. The reaction of *o*-alkynylstyrenes **1** with BCl_3_ at 0 °C in CH_2_Cl_2_ followed by treatment with pinacol and NEt_3_ at 0 °C allowed the synthesis of diverse borylated indenes **2** with substituents of different electronic and steric nature (Scheme 3). Regarding the substitution at the alkyne of the starting enynes **1**, aryl groups with both electron-donating (**2b**,**c**) and electron-withdrawing substituents, in either *para-*, *meta-*, or *ortho* positions (**2d**–**f**), were well tolerated. In addition, a polycyclic aryl (**2g**) as well as a heteroaromatic ring (**2h**) in this position provided excellent results. Moreover, an alkenyl group can also be incorporated (**2i**), although in moderate yield due to a partial HCl elimination in the reaction medium, leading to **4i**. Conversely, alkyl groups are also compatible with the developed method and result in the formation of the corresponding indenes **2j**–**l** in good yields.^15^ Finally, borylated indenes bearing substituents in different positions of the benzene ring (**2m**–**o**) can also be efficiently synthesized following this methodology. Notably, the reaction can be scaled up to 1 mmol while maintaining high yields. However, a limitation of this method is that *o*-alkynylstyrenes with aryl groups as substituents of the alkene moiety do not produce indenes **2**. Instead, elimination occurs to form benzofulvenes **3p**,**q**, which is favored due to conjugation and can not be avoided.

**Scheme 3:**
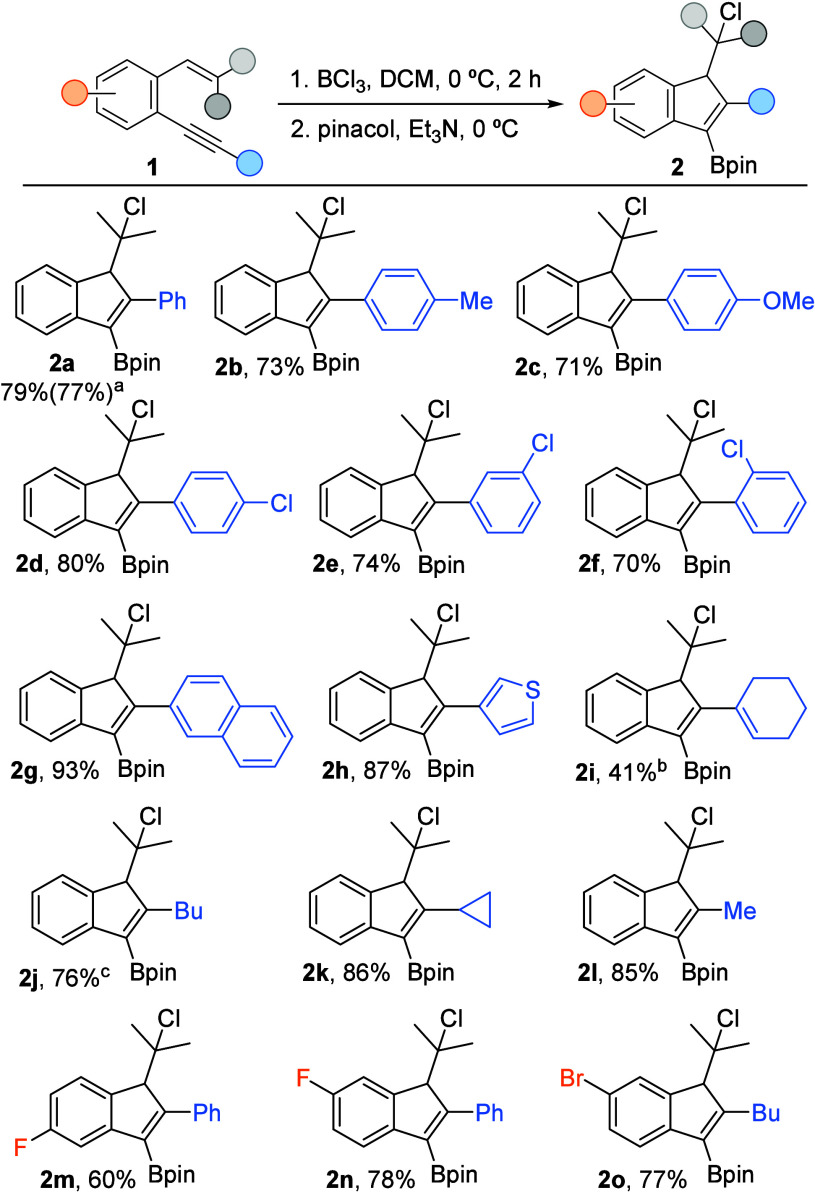
Synthesis of borylated indenes **2** (a) Reaction conducted at 1 mmol scale. (b) Accompanied with 18% of the corresponding indene **4i**. (c) Reaction conducted at −30 °C in the presence of 2,4,6-tri-*tert*-butylpyridine (TBP, 1 equiv).

On the other hand, performing the treatment with pinacol and triethylamine at 60 °C for 14 h, after the completion of the borylative cyclization, allowed the synthesis of various borylated benzofulvenes **3** (Scheme 4). Arenes bearing electron-donating (**3b**,**c**) or -withdrawing groups in either *para-* or *meta-* positions (**3d**,**e**) are suitable substituents at the alkyne of the starting enynes **1**. However, the elimination was hampered when a more sterically hindered *ortho-*substituted ring was present, resulting in a mixture of **2f** and **3f** in the crude reaction, with low conversion toward **3f** (15%). A similar result was observed when using naphthyl-substituted enyne **1g** (30% conversion toward **3g**). In addition, thiophenyl-substituted benzofulvene **3h** was prepared in lower yield due to its partial decomposition under the reaction conditions. On the contrary, both alkenyl and alkyl groups are well tolerated in this transformation, yielding the corresponding borylated benzofulvenes **3i**–**l** efficiently. Moreover, reactions involving substrates substituted at the internal arene produced the corresponding benzofulvenes **3m**,**o** in high yields. Notably, diverse substitutions can be introduced in the exocyclic alkene of the carbocycle. Thus, benzofulvenes **3p**,**q** mono- and diaryl substituted on the external alkene could be synthesized in high yields, and a cyclic substituent at that position also proved compatible with this methodology (**3r**).

**Scheme 4:**
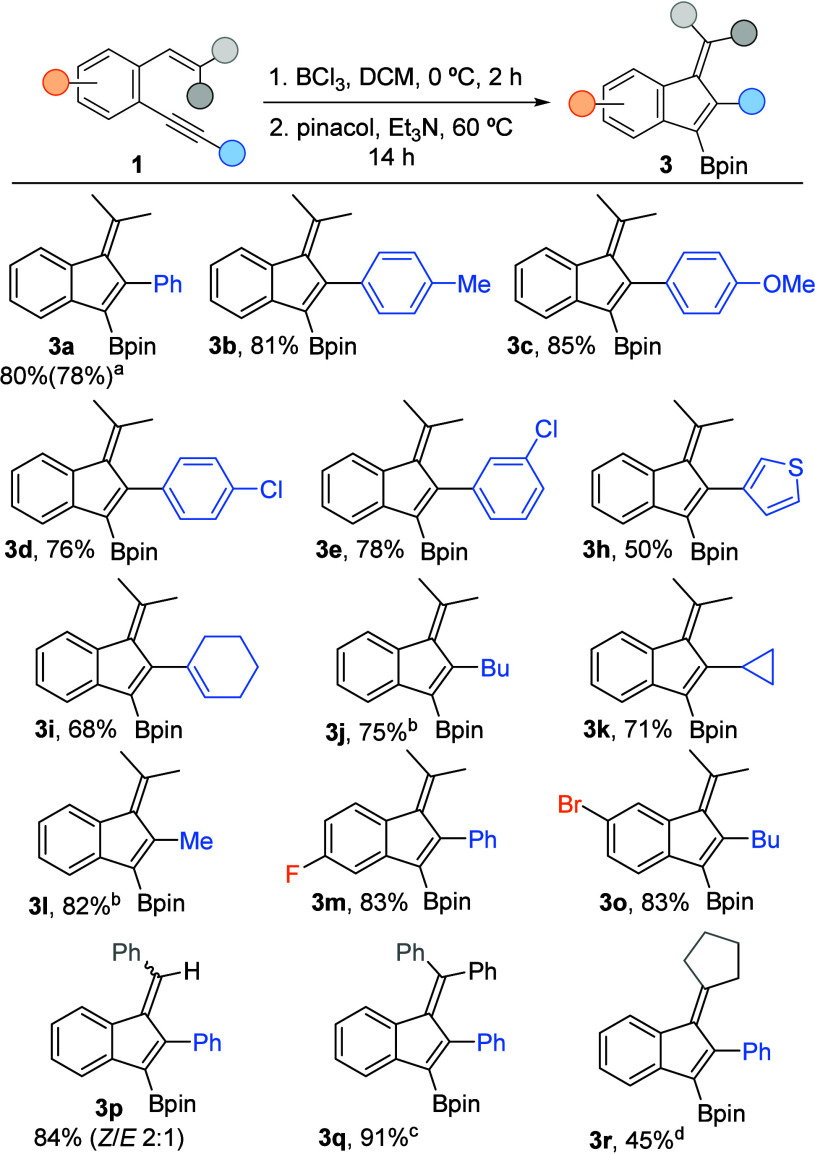
Synthesis of Borylated Fulvenes **3** (a) Reaction conducted at 1 mmol scale. (b) Purification with deactivated silica gel. (c) Reaction conducted with 4 equiv of BCl_3_ in the presence of TBP (1 equiv). (d) Accompanied with 11% of the corresponding eliminated indene **4r**.

The synthetic usefulness of borylated benzofulvenes **3** as building blocks was preliminarily demonstrated with different transformations of **3a**, selected as a model (Scheme 5). It can be easily converted into the corresponding potassium trifluoroborate salt (**6a**) or protodeborylated to yield benzofulvene **8a**. Additionally, it participates in Suzuki couplings for the introduction of aromatic substituents at position 3 (**7a**) and can be efficiently oxidized to ketone **9a**.

**Scheme 5:**
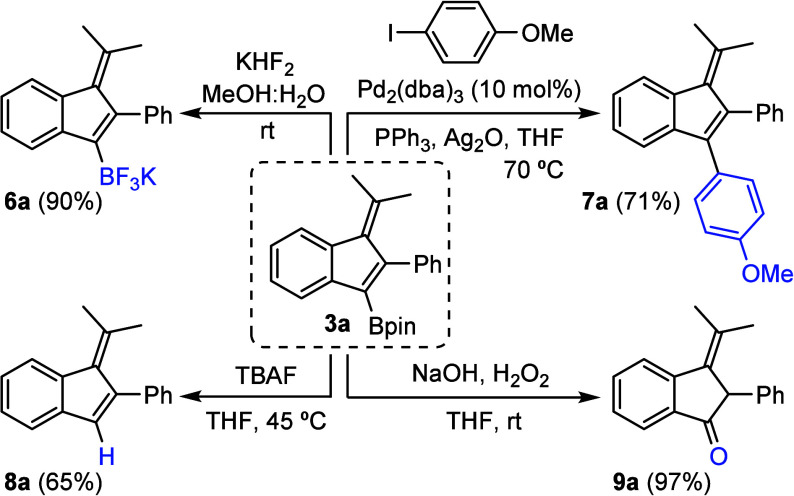
Derivatizations of Borylated Benzofulvene **3a**

Finally, to further demonstrate the utility of the developed methodology, it has been applied to the synthesis of Sulindac. To this end, *o*-alkynylstyrene **1s** was prepared from commercially available reagents and cyclized in the presence of BCl_3_ (Scheme 6). For this particular substrate, it was necessary to heat at 70 °C and employ 4 equiv of the boron electrophile to promote the reaction and to add TBP to avoid partial deborylation of the final product in the reaction medium. Under these conditions, borylated benzofulvene **3s** was obtained in high yield as a mixture of *Z*/*E* isomers. Next, a palladium catalyzed coupling between **3s** and α-bromoethyl acetate provided **10s**, also as a *Z*/*E* mixture. Treatment of **10s** with HCl in acetic acid not only performed the anticipated hydrolysis of the ester group, but also promoted the convergence of both alkene isomers into the desired *Z* olefin. Finally, Sulindac was obtained by oxidation of the sulfide to the corresponding sulfoxide.

**Scheme 6:**
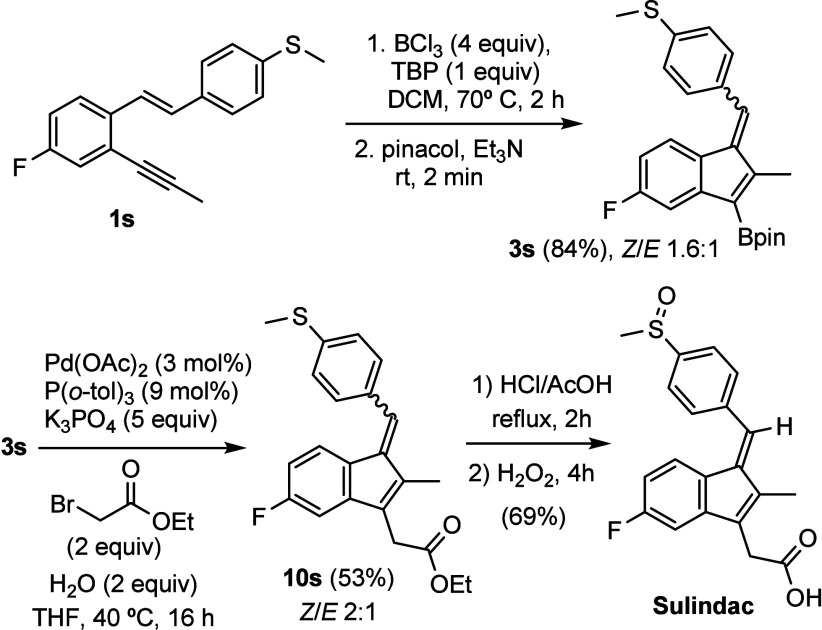
Sulindac Synthesis

In summary, we have presented a mild and versatile metal-free approach for the synthesis of borylated indenes and benzofulvenes using easily accessible *o*-alkynylstyrenes. These reactions occur in high yield, with wide scope and complete selectivity, controllable by simple adjustment of reaction temperature in the derivatization of the initially formed BCl_2_-indenes into isolable pinacol boronate esters. The utility of the resulting borylated building blocks is evidenced by their diverse functionalization at the C–B bond. Moreover, the developed methodology serves as a key step in the synthesis of Sulindac, a nonsteroidal anti-inflammatory drug. Overall, this work extends the applicability of metal-free borylative cyclizations, providing access to valuable B-indenes and benzofulvenes from readily available starting materials, complementing the existing metal-based strategies.

## Data Availability

The data underlying this study are available in the published article and its Supporting Information.

## Supporting Information Available

Experimental details and NMR spectra for all new compounds (PDF)

## References

[ref1] aChen S.; Li Q.; Sun S.; Ding Y.; Hu A. Novel Approach toward Polyfulvene: Cationic Polymerization of Enediynes. Macromolecules 2017, 50, 534–541. 10.1021/acs.macromol.6b02321.

[ref2] aFischer M.; Oswald T.; Ebert H.; Schmidtmann M.; Beckhaus R. Expanding the Scope: Monopentafulvene and -Benzofulvene Complexes of Zirconium and Hafnium. Organometallics 2018, 37, 415–421. 10.1021/acs.organomet.7b00832.

[ref3] aPrasher P.; Sharma M. Medicinal Chemistry of Indane and its Analogues: A Mini Review. ChemistrySelect 2021, 6, 2658–2677. 10.1002/slct.202100177.

[ref4] aShi C.; Feng C.; Chen Y.; Zhang S.; Lin G. Recent Advancement in Benzofulvene Synthesis. Chin. J. Org. Chem. 2020, 40, 817–830. 10.6023/cjoc201910029.

[ref5] aGarcía-García P.; Sanjuán A. M.; Rashid M. A.; Martínez-Cuezva A.; Fernández-Rodríguez M. A.; Rodríguez F.; Sanz R. Synthesis of Functionalized 1*H*-indenes and Benzofulvenes through Iodocyclization of *o*-(Alkynyl)styrenes. J. Org. Chem. 2017, 82, 1155–1165. 10.1021/acs.joc.6b02788. 27992220

[ref6] aGrams R. J.; Santos W. L.; Scorei I. R.; Abad-García A.; Rosenblum C. A.; Bita A.; Cerecetto H.; Viñas C.; Soriano-Ursúa M. A. The Rise of Boron-Containing Compounds: Advancements in Synthesis, Medicinal Chemistry, and Emerging Pharmacology. Chem. Rev. 2024, 124, 2441–2511. 10.1021/acs.chemrev.3c00663. 38382032

[ref7] aHemming D.; Fritzemeier R.; Westcott S. A.; Santos W. L.; Steel P. G. Copper-Boryl Mediated Organic Synthesis. Chem. Soc. Rev. 2018, 47, 7477–7494. 10.1039/C7CS00816C. 30206614

[ref8] aYang C.-H. BX_3_-Mediated Borylation for the Synthesis of Organoboron Compounds. Org. Chem. Front. 2023, 10, 6010–6020. 10.1039/D3QO01487H.

[ref9] aLuo L.; Tang S.; Wu J.; Jin S.; Zhang H. Transition Metal-Free Aromatic C–N, C–S and C–O Borylation. Chem. Rec. 2023, 23, e202300023 10.1002/tcr.202300023. 36850026

[ref10] Warner A. J.; Lawson J. R.; Fasano V.; Ingleson M. J. Formation of C(sp2)–Boronate Esters by Borylative Cyclization of Alkynes Using BCl_3_. Angew. Chem., Int. Ed. 2015, 54, 11245–11249. 10.1002/anie.201505810. PMC4832827 26237115

[ref11] aSans-Panadés E.; Vaquero J. J.; Fernández-Rodríguez M. A.; García-García P. Synthesis of BN-Polyarenes by a Mild Borylative Cyclization Cascade. Org. Lett. 2022, 24, 5860–5865. 10.1021/acs.orglett.2c02477. 35913827 PMC9384698

[ref12] Warner A. J.; Enright K. M.; Cole J. M.; Yuan K.; McGough J. S.; Ingleson M. J. Borylative cyclisation of diynes using BCl_3_ and borocations. Org. Biomol. Chem. 2019, 17, 5520–5525. 10.1039/C9OB00991D. 31120094

[ref13] aMilián A.; Fernández-Rodríguez M. A.; Merino E.; Vaquero J. J.; García-García P. Metal-Free Temperature-Controlled Regiodivergent Borylative Cyclizations of Enynes: BCl_3_-Promoted Skeletal Rearrangement. Angew. Chem., Int. Ed. 2022, 61, e202205651 10.1002/anie.202205651. PMC9401584 35510716

[ref14] Oka N.; Yamada T.; Sajiki H.; Akai S.; Ikawa T. Aryl Boronic Esters Are Stable on Silica Gel and Reactive Under Suzuki–Miyaura Coupling Conditions. Org. Lett. 2022, 24, 3510–3514. 10.1021/acs.orglett.2c01174. 35500272

[ref15] Reaction of *o*-alkynylstyrene **1j** under standard conditions lead to a mixture of products. However, selective formation of **2j** was achieved at – 30 °C in the presence of TBP.

