# Supplementary material for: Selective Synthesis of Boron-Functionalized Indenes and Benzofulvenes by BCl3-Promoted Cyclizations of ortho-Alkynylstyrenes
Source: Org Lett. 2024 Jul 29;26(31):6568–73. doi: 10.1021/acs.orglett.4c02092 (PMC11320658; doi:10.1021/acs.orglett.4c02092)

Supporting Information

Selective Synthesis of Boron-functionalized Indenes and Benzofulvenes by BCl_3-Promoted Cyclizations of *ortho*-Alkynylstyrenes

Marcos Humanes,† Ester Sans-Panadés,† Cintia Virumbrales,‡ Ana Milián,† Roberto Sanz,‡ Patricia García-García,†,* and Manuel A. Fernández-Rodríguez,†,*

† Universidad de Alcalá (IRYCIS). Departamento de Química Orgánica y Química Inorgánica, Instituto de Investigación Química "Andrés M. del Río" (IQAR), 28805 Alcalá de Henares, Madrid, Spain.

‡Área de Química Orgánica. Departamento de Química, Facultad de Ciencias, Universidad de Burgos, Pza. Misael Bañuelos, s/n 09001, Burgos, Spain

Table of Contents

General Experimental Details -- S2

Experimental Procedures and Characterization Data --------------------------------------- S3

Table S1. Optimization of the cyclization of **1a** --------------------------------------- S3

Synthesis of *o*-alkynylstyrenes **1**: -- S3

Characterization data of new *o*-alkynylstyrenes **1** -------------------------------------- S4

General procedure for synthesis of borylated indenes **2** ------------------------------ S6

Characterization data of borylated indenes **2** -- S6

General procedure for synthesis of borylated benzofulvenes **3** ----------------------S14

Characterization data of borylated benzofulvenes **3**---------------------------------S14

Synthetic procedures and characterization data for the derivatizations of borylated benzofulvene **3a**--S20

Synthesis of Sulindac --S21

Copies of ^{1}H and ^{13}C spectra for novel compounds and selected gCOSY, TOCSY, NOESY, gHSQC and gHMBC spectra --S25

General Experimental Details

All reactions involving air or moisture sensitive compounds were carried out under inert atmosphere (Ar). Glassware was dried in a hot oven overnight. Triethylamine was dried over calcium hydride, distilled under vacuum and stored over molecular sieves (3 Å) under inert atmosphere. Temperatures are reported as oil-bath temperatures. Dry solvents, where necessary, were dried by a MBRAUN MB-SPS-800 apparatus. Starting materials sourced from commercial suppliers were used as received unless otherwise stated. Substrates **1a**,**c-e**,**h-l**,**n-r** and their non-commercial precursors were previously described and were prepared as reported.[1] Reactions were monitored using analytical TLC plates (Merck; silica gel 60 F254, 0.25 mm), and compounds were visualized with UV radiation. Silica gel grade 60 (70-230 mesh, Merck) was used for column chromatography. All melting points were determined in open capillary tubes on a Stuart Scientific SMP3 melting point apparatus (uncorrected). ^{1}H, ^{13}C and ^{11}B NMR spectra were recorded on either Varian Mercury VX-300, Varian Unity 300, Bruker Avance Neo 400 or Varian Unity 500 MHz spectrometer at room temperature. Chemical shifts are given in ppm (δ) downfield from tetramethylsilane, with calibration on the residual protio-solvent used (δ_H = 7.26 ppm and δ_C = 77.2 ppm for $CDCl_3$). Coupling constants (*J*) are in Hertz (Hz) and signals are described as follows: s, singlet; d, doublet; t, triplet; aqui, apparent quintet; bs, broad singlet; dd, double doublet; dt, doublet of triplets; ddd, double doublet of doublets; ddt, double doublet of triplets; td, triplet of doublets; tt, triplet of triplets and m, multiplet. Resonances for the carbon directly bonded to boron are not observed in the $^{13}C\{^{1}H\}$ NMR spectra due to quadropolar relaxation effects. Structural assignments were made with additional information from gCOSY, gHSQC, and gHMBC experiments. High-resolution analyses (HRMS) were performed on an Agilent 6210 time of-flight LC/MS.

[1] a) Liu, X.-W.; Li, S.-S.; Dai, D.-T.; Zhao, M.; Shan, C.-C.; Xu, Y.-H.; Loh, T.-P. *Org. Lett.* **2020**, *21*, 3696−3700. b) Sanjuán, A. M.; Virumbrales, C.; García-García, P.; Fernández-Rodríguez, M. A.; Sanz, R. *Org. Lett.* **2016**, *18*, 1072−1075. c) Sanjuán, A. M.; Rashid, M. A.; García-García, P.; Martínez-Cuezva, A.; Fernández-Rodríguez, M. A.; Rodríguez, F.; Sanz, R. *Chem.−Eur. J.* **2015**, *21*, 3042−3052. d) Vasu, D.; Hung, H.-H.; Bhunia, S.; Gawade, S. A.; Das, A.; Liu, R.-S. *Angew. Chem. Int. Ed.* **2011**, *50*, 6911–6914. e) Sanz, R.; Martínez, A.; García-García, P.; Fernández-Rodríguez, M. A.; Rashid, M. A.; Rodríguez, F. *Chem. Commun.* **2010**, *46*, 7427−7429. f) Martínez, A.; García-García, P.; Fernández-Rodríguez, M. A.; Rodríguez, F.; Sanz, R. *Angew. Chem., Int. Ed.* **2010**, *49*, 4633−4637. g) Madhushaw, R. J.; Lo, C.-Y.; Hwang, C.-W.; Su, M.-D.; Shen, H.-C.; Pal, S.; Shaikh, I. R.; Liu, R.-S. *J. Am. Chem. Soc.* **2004**, *126*, 15560–15565.

Experimental Procedures and Characterization Data

Table S1. Optimization of the cyclization of 1a:

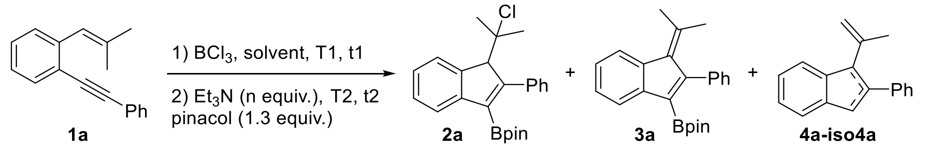

entry	BCl_3 equiv.	solvent	T1 (ºC)	t1	NEt_3 equiv.	T2 (ºC)	t2	2a/3a/4a-iso4a[a]
1	3	CH_2Cl_2	60	2 h	2	60	20 min	7/36/57
2	3	CH_2Cl_2	60	4 h	15	60	16 h	-/54/46
3	1	CH_2Cl_2	60	2 h	2	25	20 min	51/18/31
4	2	CH_2Cl_2	25	2 h	2	25	20 min	83/14/3
5	3	CH_2Cl_2	25	2 h	2	25	20 min	84/15/1
6	3	CH_2Cl_2	25	2 h	5	25	20 min	90/9/1
7	3	CH_2Cl_2	25	12 h	5	25	20 min	75/15/10
8	3	MeCN	25	2 h	5	25	20 min	-
9	3	THF	25	2 h	5	25	20 min	-
10	3	toluene	25	2 h	5	25	20 min	83/16/1
11[b]	3	CH_2Cl_2	25	2 h	5	25	20 min	57/41/2
12	3	CH_2Cl_2	0	2 h	5	25	20 min	100/-/-
13	3	CH_2Cl_2	0	2 h	5	25	4 h	39/55/6
14	3	CH_2Cl_2	0	2 h	5	25	16 h	23/67/10
15	3	CH_2Cl_2	0	2 h	5	60	20 min	19/81/-
16	3	CH_2Cl_2	0	2 h	5	60	4 h	4/96/-
17	3	CH_2Cl_2	0	2 h	15	60	16 h	-/100/-
18	3	CH_2Cl_2	60	4 h	5	60	20 min	-/51/42

[a] Conversion and proportions estimated by ^{1}H NMR (300 MHz); all the experiments were conducted with 0.2 mmol of **1a**. [b] Reaction conducted in the presence of TBP (2 equiv.).

Synthesis of *o*-alkynylstyrenes 1: Substrates **1b**,**f-g**,**m** were synthesized following previously reported procedures, grouped into Methods A and B.[1]

Method A: Substrates **1m** and **1-TIPS** were prepared from aldehydes **I** through a Sonogashira coupling with the appropriate alkyne, followed by a Wittig reaction.

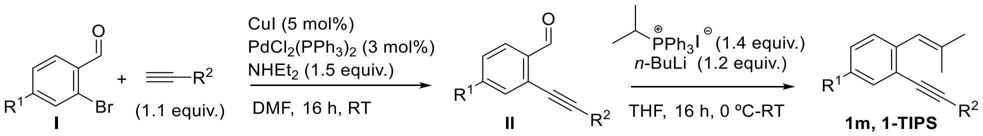

Step1: In a Schlenk tube, the appropriate acetylene (1.1 equiv.) was added to a solution of the corresponding *o*-bromoaldehyde **I** (1 equiv.), Et_2NH (1.5 equiv.), CuI (5 mol%) and $PdCl_2(PPh_3)_2$ (3 mol%) in anhydrous DMF (2 M). The resulting mixture was stirred at RT until **I** was consumed as determined by TLC. The crude mixture was partitioned between water and CH_2Cl_2 and the solvents were removed. The residue was purified by flash chromatography on silica gel using mixtures of hexane and EtOAc as eluents to obtain the corresponding benzaldehydes **II** which were used in the next step.

Step 2: *n*-BuLi (1.2 equiv., 1.6 M in hexanes) was added at 0 ºC to a solution of isopropyltriphenylphosphonium iodide (1.4 equiv.) in THF (2 M) and the mixture was stirred for 30 min at RT. A solution of the corresponding aldehyde **II** in THF (1 equiv., 1 M) was added at 0 ºC and the reaction stirred at RT until **II** was consumed as determined by TLC. The crude mixture was partitioned between water and DCM and the solvents were removed. The residue was purified by flash chromatography on silica gel using hexane as eluent to obtain *o*-(alkynyl)styrenes **1m** and **1-TIPS.**

Method B: It involves a deprotection step and a Sonogashira coupling from **1-TIPS**.

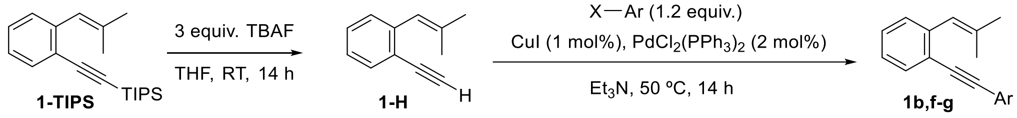

Step 1: TBAF (30 mmol, 10 mL 1.0 M in THF, 3 equiv.) was added to a solution of isolated or crude **1-TIPS** (3.1 g, 10 mmol, 1 equiv.) in THF (4 mL) at RT and the resulting mixture was stirred until complete consumption of the substrate as monitored by TLC or GC/MS. The crude mixture was partitioned between water and DCM and the solvents were removed under reduced pressure. The crude was purified by flash chromatography on silica gel using hexane as eluent to obtain *o*-(alkynyl)styrene **1-H**.

Step 2: To a solution of the appropriate haloarene (1.2 equiv.), CuI (1 mol%), $PdCl_2(PPh_3)_2$ (2 mol%) in anhydrous and degassed Et_3N (0.22 M), **1-H** (1 equiv.) was added. The resulting mixture was stirred at 50 ºC until complete consumption of **1-H**, as monitored by TLC. Water and DCM or EtOAc were added to the cooled reaction mixture. The separated aqueous phase was extracted with DCM or EtOAc and the combined organic layers were dried over anhydrous Na_2SO_4, filtered and evaporated. The residue was purified by flash chromatography on silica gel using mixtures of hexane and EtOAc as eluents to obtain the *o*-(alkynyl)styrenes **1**.

Characterization data of new *o*-alkynylstyrenes 1.

1-(2-Methylprop-1-en-1-yl)-2-((triisopropylsilyl)ethynyl)benzene (1-TIPS)

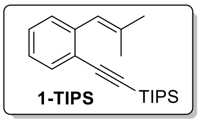

Obtained as colourless oil (2.57g mg, 16.45 mmol, 87%) following method A. R_f = 0.90 (Hexane/EtOAc 98:2).

^{1}H-NMR (400 MHz, $CDCl_3$) δ (ppm) 7.50 (d, *J* = 7.6, Hz, 1H), 7.31–7.25 (m, 2H), 7.20–7.10 (m, 1H), 6.57 (bs, 1H), 1.93 (d, *J* = 1.5 Hz, 3H), 1.83 (d, *J* = 1.4 Hz, 3H), 1.24–1.09 (m, 21H).

^{13}C-NMR (100 MHz, $CDCl_3$) δ (ppm) 141.2 (C), 136.3 (C), 132.6 (CH), 129.0 (CH), 127.9 (CH), 125.8 (CH), 124.4 (CH), 123.1 (C), 106.2 (C), 94.8 (C), 26.7 (CH_3), 19.6 (CH_3), 18.8 (6 x CH_3), 11.5 (3 x CH).

HRMS (ESI-TOF) m/z: $[M+H]^+$ Calcd for $C_{21}H_{33}Si$ 313.2346. Found 313.2347.

1-(2-Methylprop-1-en-1-yl)-2-((4-tolyl)ethynyl)benzene (1b)

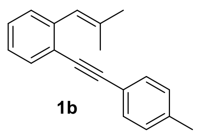

1b

Obtained as white solid (207 mg, 0.84 mmol, 84%) following method B. R_f = 0.57 (Hexane/EtOAc 98:2). M.p.: 46−48 ºC.

^{1}H-NMR (400 MHz, $CDCl_3$) δ (ppm) 7.53 (dt, J = 7.4, 1.0 Hz, 1H), 7.47–7.40 (m, 2H), 7.34–7.24 (m, 2H), 7.24–7.14 (m, 3H), 6.59 (bs, 1H), 2.38 (s, 3H), 1.99 (d, J = 1.5 Hz, 3H), 1.86 (d, J = 1.4 Hz, 3H).

^{13}C-NMR (100 MHz, $CDCl_3$) δ (ppm) 140.6 (C), 138.4 (C), 136.7 (C), 132.1 (CH), 131.5 (2 x CH), 129.23 (2 x CH), 129.20 (CH), 127.8 (CH), 126.0 (CH), 124.1 (CH), 122.9 (C), 120.7 (C), 93.7 (C), 88.2 (C), 26.9 (CH_3), 21.6 (CH_3), 19.8 (CH_3).

HRMS (ESI-TOF) m/z: $[M+H]^+$ Calcd for $C_{19}H_{19}$ 247.1481. Found 247.1486.

1-(2-Methylprop-1-en-1-yl)-2-((2-chlorophenyl)ethynyl)benzene (1f)

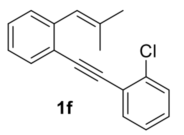

1f

Obtained as pale yellow oil (458 mg, 1.72 mmol, 86%) following method B. R_f = 0.61 (Hexane/EtOAc 98:2).

^{1}H-NMR (300 MHz, $CDCl_3$) δ (ppm) 7.62–7.50 (m, 2H), 7.48–7.39 (m, 1H), 7.35–7.30 (m, 2H), 7.29–7.15 (m, 3H), 6.68 (bs, 1H), 1.97 (d, J = 1.5 Hz, 3H), 1.86 (d, J = 1.4 Hz, 3H).

^{13}C-NMR (100 MHz, $CDCl_3$) δ (ppm) 141.0 (C), 137.0 (C), 136.0 (C), 133.3 (CH), 132.4 (CH), 129.4 (CH), 129.21 (CH), 129.19 (CH), 128.3 (CH), 126.6 (CH), 126.0 (CH), 124.2 (CH), 123.7 (C), 122.3 (C), 94.1 (C), 90.4 (C), 26.9 (CH_3), 19.8 (CH_3).

HRMS (ESI-TOF) m/z: $[M+H]^+$ Calcd for $C_{18}H_{16}Cl$ 267.0935. Found 267.0936.

1-(2-Methylprop-1-en-1-yl)-2-((2-naphthyl)ethynyl)benzene (1g)

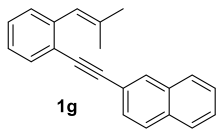

1g

Obtained as pale yellow oil (499 mg, 1.77 mmol, 88%) following method B. R_f = 0.52 (Hexane/EtOAc 98:2).

^{1}H-NMR (300 MHz, $CDCl_3$) δ (ppm) 8.17 (bs, 1 H), 7.97–7.85 (m, 3H), 7.78–7.67 (m, 2H), 7.63–7.53 (m, 2H), 7.50–7.37 (m, 2H), 7.32 (td, J = 7.3, 2.0 Hz, 1H), 6.81 (bs, 1H), 2.14 (bs, 3H), 2.00 (bs, 3H).

^{13}C-NMR (75 MHz, $CDCl_3$) δ (ppm) 140.7 (C), 136.8 (C), 133.1 (C), 132.8 (C), 132.3 (CH), 131.3 (CH), 129.2 (CH), 128.5 (CH), 128.1 (CH), 128.0 (CH), 127.8 (2 x CH), 126.64 (CH), 126.59 (CH), 126.0 (CH), 124.1 (CH), 122.7 (C), 121.0 (C), 94.0 (C), 89.3(C), 26.9 (CH_3), 19.8 (CH_3).

HRMS (ESI-TOF) m/z: $[M+H]^+$ Calcd for $C_{22}H_{19}$ 283.1481. Found: 283.1482.

4-Fluoro-1-(2-methylprop-1-en-1-yl)-2-(phenylethynyl)benzene (1m)

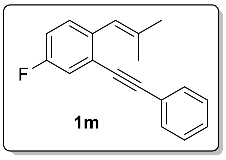

Obtained as colourless oil (185 mg, 0.74 mmol, 66%) following method A. R_f = 0.35 (Hexane).

^{1}H-NMR (400 MHz, $CDCl_3$) δ (ppm) 7.57–7.50 (m, 2H), 7.40–7.33 (m, 3H), 7.23 (dd, *J* = 9.1, 2.8 Hz, 2H), 7.01 (td, *J* = 8.5, 2.8 Hz, 1H), 6.50 (bs, 1H), 1.98 (d, *J* = 1.5 Hz, 3H), 1.83 (d, *J* = 1.3 Hz, 3H).

^{13}C-NMR (100 MHz, $CDCl_3$) δ (ppm) 160.7 (C, d, $^{1}J_{C-F}$ = 245.4 Hz), 136.95 (C, d, $^{4}J_{C-F}$ = 3.3 Hz), 136.86 (C, d, $^{6}J_{C-F}$ = 1.1 Hz), 131.7 (2 x CH), 130.7 (CH, d, $^{3}J_{C-F}$ = 8.3 Hz), 128.6 (CH), 128.5 (2 x CH), 124.3 (C, d, $^{3}J_{C-F}$ = 9.5 Hz), 123.3 (C), 123.1 (CH), 118.5 (CH, d, $^{2}J_{C-F}$ = 22.7 Hz), 115.3 (CH, d, $^{2}J_{C-F}$ = 21.2 Hz), 94.3 (C), 87.8 (C, d, $^{4}J_{C-F}$ = 3.1 Hz), 26.7 (CH_3), 19.7 (CH_3).

^{19}F-NMR (376 MHz, $CDCl_3$) δ (ppm) –116.75.

HRMS (ESI-TOF) m/z: $[M+H]^+$ Calcd for $C_{18}H_{16}F$ 251.1231. Found: 251.1233.

General procedure for the synthesis of borylated indenes 2

To a solution of the appropriate *o*-alkynylstyrene **1** (0.4 mmol, 1 equiv.) in anhydrous DCM (0.06 M) at 0 ºC and under argon, cold BCl_3 (1.0 M in heptane, 1.2 mL, 1.2 mmol, 3 equiv.) was added dropwise and the mixture was stirred for 2 h at 0 ºC. The formed intermediate **2-BCl$_2$** (that was detected and characterized by NMR for substrate **2a**) was treated with a solution of pinacol (61.5 mg, 0.52 mmol, 1.3 equiv.) in Et_3N (0.28 mL, 2 mmol, 5 equiv.) and the mixture was stirred for 20 min at 0 ºC. Then it was extracted with hexane and the organic layer washed with HCl (1M, 3 x 10 mL) and brine, dried over anhydrous Na_2SO_4, filtered and concentrated under reduced pressure. The resulting residue was purified by column chromatography on deactivated silica gel using mixtures of hexane and EtOAc as eluents to give the corresponding indenes **2**. Traces of products **3** and/or **4** were formed in particular examples.

Characterization data of borylated indenes 2

3-Dichloroboranyl-1-(2-chloropropan-2-yl)-2-phenyl-1*H*-indene (2a-BCl$_2$)

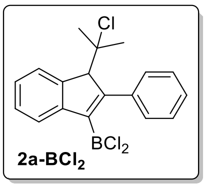

Selectively formed from **1a** (1 equiv., 14 mg, 0.06 mmol) and BCl_3 (3 equiv., 0.18 mmol, 0.18 mL) in a reaction performed at 0 ºC in CD_2Cl_2 (1 mL) in a NMR tube with a J Young valve.

^{1}H-NMR (500 MHz, CD_2Cl_2) δ (ppm) 8.02 (d, *J* = 7.8 Hz, 1H), 7.65 (d, *J* = 7.6 Hz, 1H), 7.47–7.38 (m, 4H), 7.36–7.21 (m, 3H), 4.51 (s, 1H), 1.42 (s, 3H). A methyl group is not observed as it is overlapped by the signals of heptane from the BCl_3 solution.

^{13}C-NMR (125 MHz, CD_2Cl_2) δ (ppm) 160.9 (C), 145.0 (C), 143.9 (C), 138.4 (C), 129.0 (2 x CH), 128.70 (CH), 128.3 (2 x CH), 127.8 (CH), 126.7 (CH), 125.3 (CH), 121.9 (CH), 64.5 (CH), 33.8 (CH_3), 28.5 (CH_3). Csp^2–B signal is not observed.

^{11}B-NMR (128 MHz, $CDCl_3$) δ 56.2.

1-(2-Chloropropan-2-yl)-2-phenyl-3-(4,4,5,5-tetramethyl-1,3,2-dioxaborolan-2-yl)-1*H*-indene (2a)

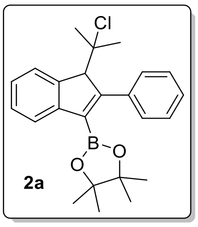

Obtained as yellow oil (122 mg, 0.3 mmol, 79%) from **1a** (93 mg, 0.4 mmol). The corresponding reaction using 232 mg (1 mmol) of **1a** afforded 304 mg of **2a** (77% yield). R$_f$ = 0.35 (Hexane/EtOAc 30:1).

^{1}H-NMR (300 MHz, CDCl$_3$) δ (ppm) 7.97 (dd, *J* = 7.5, 1.0 Hz, 1H), 7.58 (dd, *J* = 7.5, 1.0 Hz, 1H), 7.44–7.27 (m, 6H), 7.20 (td, *J* = 7.3, 1.3 Hz, 1H), 4.42 (s, 1H), 1.35 (s, 3H), 1.27 (s, 12H), 1.19 (s, 3H).

^{13}C-NMR (75 MHz, CDCl$_3$) δ (ppm) 159.0 (C), 147.7 (C), 144.7 (C), 139.3 (C), 129.2 (2 x CH), 127.9 (2 x CH), 127.70 (CH), 127.66 (CH), 126.4 (CH), 124.7 (CH), 122.2 (CH), 83.9 (2 x C), 73.2 (C), 64.5 (CH), 34.7 (CH$_3$), 28.0 (CH$_3$), 25.2 (2 x CH$_3$), 24.5 (2 x CH$_3$). Csp^2–B signal is not observed.

^{11}B-NMR (128 MHz, CDCl$_3$) δ 30.6.

HRMS (ESI-TOF) m/z: [M+H]$^+$ Calcd for $C_{24}H_{29}BClO_2$ 395.1944. Found 395.1934.

1-(2-Chloropropan-2-yl)-2-phenyl-3-(4,4,5,5-tetraethyl-1,3,2-dioxaborolan-2-yl)-1*H*-indene (2a')

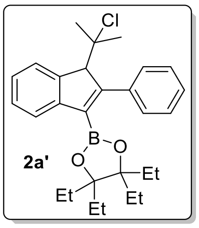

Obtained as yellow oil (164 mg, 0.36 mmol, 91%) from **1a** (93 mg, 0.4 mmol) using ethylpinacol. R$_f$ = 0.50 (Hexane/EtOAc 30:1).

^{1}H-NMR (400 MHz, CDCl$_3$) δ (ppm) 7.90 (d, *J* = 7.6 Hz, 1H), 7.63 (d, *J* = 7.6 Hz, 1H), 7.34–7.16 (m, 6H), 7.11 (td, *J* = 7.5, 1.2 Hz, 1H), 4.29 (s, 1H), 1.74–1.45 (m, 8H), 1.25 (s, 3H), 1.12 (s, 3H), 0.80–0.68 (m, 12H).

^{13}C-NMR (100 MHz, CDCl$_3$) δ (ppm) 160.2 (C), 148.0 (C), 144.6 (C), 139.6 (C), 129.4 (2 x CH), 127.68 (CH), 127.66 (2 x CH), 127. (CH), 126.3 (CH), 124.6 (CH), 122.6 (CH), 88.8 (2 x C), 73.3 (C), 65.2 (CH), 34.7 (CH$_3$), 28.2 (CH$_3$), 26.4 (2 x CH$_2$), 26.1 (2 x CH$_2$), 9.0 (2 x CH$_3$), 8.8 (2 x CH$_3$). Csp^2–B signal is not observed.

^{11}B-NMR (128 MHz, CDCl$_3$) δ 29.5.

HRMS (ESI-TOF) m/z: [M+H]$^+$ Calcd for $C_{28}H_{37}BClO_2$ 451.2570. Found 451.2558.

2-Phenyl-1-(prop-1-en-2-yl)-3-(4,4,5,5-tetramethyl-1,3,2-dioxaborolan-2-yl)-1*H*-indene (4a)

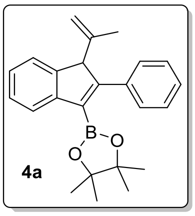

Obtained as yellow oil (87 mg, 0.24 mmol, 61% from **1a**) after stirring **2a** with silica gel for 2 h. R$_f$ = 0.30 (Hexane/EtOAc 50:1).

^{1}H-NMR (300 MHz, CDCl$_3$) δ (ppm) 7.65 (d, *J* = 7.5 Hz, 1H), 7.53–7.46 (m, 2H), 7.42–7.22 (m, 5H), 7.18 (td, *J* = 7.4, 1.2 Hz, 1H), 5.20 (s, 1H), 4.94 (s, 1H), 4.62 (s, 1H), 1.38 (s, 6H), 1.36 (s, 6H), 1.06 (s, 3H).

^{13}C-NMR (75 MHz, CDCl$_3$) δ (ppm) 160.9 (C), 147.8 (C), 146.0 (C), 144.4 (C), 136.8 (C), 129.1 (2 x CH), 127.88 (CH), 127.85 (2 x CH), 127.3 (CH), 125.1 (CH), 123.2 (CH), 122.2 (CH), 115.4 (CH$_2$), 83.9 (2 x C), 62.1 (CH), 25.3 (2 x CH$_3$), 24.7 (2 x CH$_3$), 17.2 (CH$_3$). Signal of the Csp^2 attached to the boron is not observed.

^{11}B-NMR (128 MHz, CDCl$_3$) δ 30.3.

HRMS (ESI-TOF) m/z: [M+H]$^+$ Calcd for $C_{24}H_{28}BO_2$ 359.2177. Found 359.2190.

1-(2-Bromopropan-2-yl)-2-phenyl-3-(4,4,5,5-tetramethyl-1,3,2-dioxaborolan-2-yl)-1*H*-indene (5a)

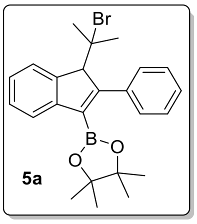

Obtained as orange oil (137 mg, 0.31 mmol, 78%) from **1a** (93 mg, 0.4 mmol) using BBr_3 instead of BCl_3. R_f = 0.28 (Hexane/EtOAc 50:1).

^{1}H-NMR (400 MHz, $CDCl_3$) δ (ppm) 7.97 (d, *J* = 7.7 Hz, 1H), 7.49 (dt, *J* = 7.7 Hz, 1H), 7.35–7.15 (m, 6H), 7.12 (td, *J* = 7.5, 1.3 Hz, 1H), 4.43 (s, 1H), 1.56 (s, 3H), 1.29 (s, 3H), 1.18 (bs, 12H).

^{13}C-NMR (100 MHz, $CDCl_3$) δ (ppm) 158.9 (C), 147.8 (C), 144.9 (C), 139.4 (C), 129.2 (2 x CH), 127.9 (2 x CH), 127.8 (CH), 127.7 (CH), 126.1 (CH), 124.6 (CH), 122.2 (CH), 83.9 (2 x C), 69.2 (C), 65.1 (CH), 36.9 (CH_3), 30.3 (CH_3), 25.2 (2 x CH_3), 24.6 (2 x CH_3). Csp^2–B signal is not observed.

^{11}B-NMR (128 MHz, $CDCl_3$) δ 30.5.

HRMS (ESI-TOF) m/z: $[M+H]^+$ Calcd for $C_{24}H_{29}BBrO_2$ 439.1438. Found 439.1450.

1-(2-Chloropropan-2-yl)-3-(4,4,5,5-tetramethyl-1,3,2-dioxaborolan-2-yl)-2-tolyl-1*H*-indene (2b)

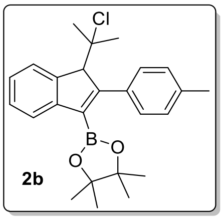

Obtained as pale yellow oil (119 mg, 0.29 mmol, 73%) from **1b** (99 mg, 0.4 mmol). R_f = 0.33 (Hexane/EtOAc 50:1).

^{1}H-NMR (400 MHz, $CDCl_3$) δ (ppm) 7.96 (d, *J* = 7.6 Hz, 1H), 7.56 (dt, *J* = 7.6, 1.0 Hz, 1H), 7.33–7.25 (m 3H), 7.20–7.13 (m 3H), 4.40 (s, 1H), 2.38 (s, 3H), 1.34 (s, 3H), 1.28 (s, 12H), 1.16 (s, 3H).

^{13}C-NMR (100 MHz, $CDCl_3$) δ (ppm) 159.0 (C), 147.9 (C), 144.7 (C), 137.4 (C), 136.4 (C), 129.1 (2 x CH), 128.6 (2 x CH), 127.7 (CH), 126.4 (CH), 124.6 (CH), 122.1 (CH), 83.6 (2 x C), 73.4 (C), 64.4 (CH), 34.8 (CH_3), 27.9 (CH_3), 25.2 (2 x CH_3), 24.6 (2 x CH_3), 21.5 (CH_3). Signal of the sp^2 carbon attached to the boron is not observed.

^{11}B-NMR (128 MHz, $CDCl_3$) δ 30.9.

HRMS (ESI-TOF) m/z: $[M+H]^+$ Calcd for $C_{25}H_{31}BClO_2$ 409.2100. Found 409.2104.

1-(2-Chloropropan-2-yl)-2-(4-methoxyphenyl)-3-(4,4,5,5-tetramethyl-1,3,2-dioxaborolan-2-yl)-1*H*-indene (2c)

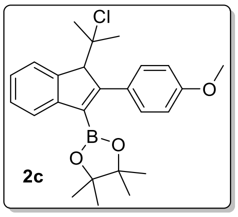

Obtained as yellow oil (120 mg, 0.28 mmol, 71%) from **1c** (105 mg, 0.4 mmol). R_f = 0.22 (Hexane/EtOAc 100:1).

^{1}H-NMR (400 MHz, $CDCl_3$) δ (ppm) 7.86 (d, *J* = 7.5 Hz, 1H), 7.49 (d, *J* = 7.5 Hz, 1H), 7.26–7.19 (m, 3H), 7.09 (td, *J* = 7.5, 1.3 Hz, 1H), 6.79 (d, *J* = 8.7 Hz, 2H), 4.28 (s, 1H), 3.76 (s, 3H), 1.26 (s, 3H), 1.20 (s, 12H), 1.09 (s, 3H).

^{13}C-NMR (75 MHz, $CDCl_3$) δ (ppm) 159.4 (C), 158.9 (C), 147.9 (C), 144.6 (C), 131.9 (C), 130.4 (2 x CH), 127.7 (CH), 126.3 (CH), 124.5 (CH), 122.0 (CH), 113.3 (2 x CH), 83.8 (2 x C), 73.5 (C), 64.5 (CH), 55.4 (CH_3), 34.7 (CH_3), 28.0 (CH_3), 25.2 (2 x CH_3), 24.6 (2 x CH_3). Csp^2–B signal is not observed.

^{11}B-NMR (128 MHz, $CDCl_3$) δ 29.9.

HRMS (ESI-TOF) m/z: $[M+H]^+$ Calcd for $C_{25}H_{31}BClO_3$ 425.2049. Found 425.2055.

2-(4-Chlorophenyl)-1-(2-Chloropropan-2-yl)-3-(4,4,5,5-tetramethyl-1,3,2-dioxaborolan-2-yl)-1*H*-indene (2d)

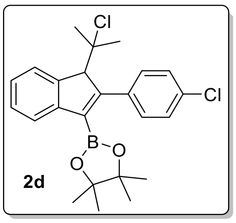

Obtained as pale yellow oil (137 mg, 0.32 mmol, 80%) from **1d** (106 mg, 0.4 mmol). R_f = 0.32 (Hexane/EtOAc 30:1).

^{1}H-NMR (400 MHz, CDCl$_3$) δ (ppm) 7.85 (d, *J* = 7.6 Hz, 1H), 7.52 (d, *J* = 7.6 Hz, 1H), 7.29–7.20 (m, 5H), 7.12 (td, *J* = 7.6, 1.2 Hz, 1H), 4.28 (s, 1H), 1.26 (s, 3H), 1.20 (s, 12H), 1.15 (s, 3H).

^{13}C-NMR (100 MHz, CDCl$_3$) δ (ppm) 157.8 (C), 147.5 (C), 144.5 (C), 137.9 (C), 133.6 (C), 130.6 (2 x CH), 128.0 (2 x CH), 127.8 (CH), 126.3 (CH), 125.0 (CH), 122.5 (CH), 84.0 (2 x C), 72.7 (C), 64.7 (CH), 34.3 (CH$_3$), 28.6 (CH$_3$), 25.2 (2 x CH$_3$), 24.6 (2 x CH$_3$). Csp^2–B signal is not observed.

^{11}B-NMR (128 MHz, CDCl$_3$) δ 30.05.

HRMS (ESI-TOF) m/z: [M+H]$^+$ Calcd for $C_{24}H_{28}BCl_2O_2$ 429.1554. Found 429.1552.

2-(3-Chlorophenyl)-1-(2-Chloropropan-2-yl)-3-(4,4,5,5-tetramethyl-1,3,2-dioxaborolan-2-yl)-1*H*-indene (2e)

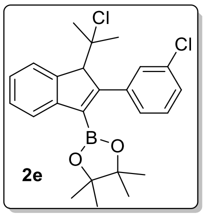

Obtained as pale yellow oil (127 mg, 0.30 mmol, 74%) from **1e** (106 mg, 0.4 mmol). R_f = 0.39 (Hexane/EtOAc 30:1).

^{1}H-NMR (400 MHz, CDCl$_3$) δ (ppm) 7.84 (d, *J* = 7.6 Hz, 1H), 7.51 (d, *J* = 7.6 Hz, 1H), 7.35–7.29 (m, 1H), 7.23 (td, *J* = 7.5, 1.2 Hz, 1H), 7.20–7.13 (m, 3H), 7.11 (td, *J* = 7.6, 1.2 Hz, 1H), 4.29 (s, 1H), 1.27 (s, 3H), 1.19 (s, 12H), 1.14 (s, 3H).

^{13}C-NMR (100 MHz, CDCl$_3$) δ (ppm) 157.3 (C), 147.3 (C), 144.5 (C), 141.0 (C), 133.7 (C), 129.1 (CH), 129.0 (CH), 127.8 (CH), 127.6 (CH), 127.4 (CH), 126.2 (CH), 125.1 (CH), 122.5 (CH), 84.0 (2 x C), 72.6 (C), 64.5 (CH), 34.4 (CH$_3$), 28.5 (CH$_3$), 25.2 (2 x CH$_3$), 24.6 (2 x CH$_3$). Csp^2–B signal is not observed.

^{11}B-NMR (128 MHz, CDCl$_3$) δ 30.4.

HRMS (ESI-TOF) m/z: [M+H]$^+$ Calcd for $C_{24}H_{28}BCl_2O_2$ 429.1554. Found 429.1562.

2-(2-Chlorophenyl)-1-(2-Chloropropan-2-yl)-3-(4,4,5,5-tetramethyl-1,3,2-dioxaborolan-2-yl)-1*H*-indene (2f)

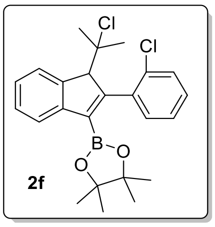

Obtained as pale yellow oil (120 mg, 0.28 mmol, 70%) from **1f** (106 mg, 0.4 mmol). R_f = 0.37 (Hexane/EtOAc 30:1). Due to the limited rotation of the chlorophenyl group, the product appears on NMR spectra as a pair of rotamers in 1.0:0.8 ratio.

^{1}H-NMR (400 MHz, CDCl$_3$) δ (ppm) 8.04 (d, *J* = 7.6 Hz, 1H, min), 7.91 (d, *J* = 7.6 Hz, 1H, maj), 7.80–7.71 (m, 1H, min), 7.66 (d, *J* = 7.6 Hz, 1H, maj), 7.39–7.28 (m, 5H), 7.27–7.16 (m, 7H), 4.85 (s, 1H, maj), 4.34 (s, 1H, min), 1.43 (s, 3H, maj), 1.38 (s, 3H, min), 1.35 (s, 3H, maj), 1.31 (s, 3H, min) 1.24 (s, 6H, min), 1.22 (s, 6H, min), 1.18 (s, 6H, maj), 1.16 (s, 6H, maj).

^{13}C-NMR (100 MHz, CDCl$_3$) δ (ppm) 157.7 (C, maj), 154.5 (C, min), 147.13 (C, maj), 147.07 (C, min), 144.50 (C, min), 144.47 (C, maj), 138.9 (C, min), 138.7 (C, maj), 133.3 (C, maj), 132.9 (C, min), 132.3 (CH, maj), 130.8 (CH, min), 129.53 (CH, min), 129.48

(CH, maj), 128.9 (CH, maj), 128.3 (CH, min), 127.8 (CH, min), 127.5 (CH, maj), 126.27 (CH, maj), 126.20 (CH, min), 126.19 (CH, maj), 125.8 (CH, min), 125.12 (CH, min), 125.06 (CH, maj), 122.75 (CH, min), 122.68 (CH, maj), 83.6 (2 x C, maj), 83.5 (2 x C, min), 73.7 (C, min), 72.5 (C, maj), 66.8 (CH, min), 63.8 (CH, maj), 34.5 (CH_3, min), 32.4 (CH_3, maj), 29.4 (CH_3, maj), 28.2 (CH_3, min), 25.3 (2 x CH_3, min), 25.1 (2 x CH_3, maj), 24.7 (2 x CH_3, min), 24.5 (2 x CH_3, maj). Csp^2–B signal is not observed.

^{11}B-NMR (128 MHz, $CDCl_3$) δ 30.0.

HRMS (ESI-TOF) m/z: $[M+Na]^+$ Calcd for $C_{24}H_{27}BCl_2NaO_2$ 451.1373. Found 451.1391.

1-(2-Chloropropan-2-yl)-2-(2-naphthyl)-3-(4,4,5,5-tetramethyl-1,3,2-dioxaborolan-2-yl)-1*H*-indene (2g)

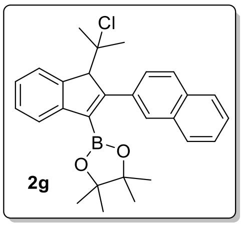

Obtained as pale yellow oil (165 mg, 0.37 mmol, 93%) from **1g** (112 mg, 0.4 mmol). R_f = 0.39 (Hexane/EtOAc 30:1).

^{1}H-NMR (400 MHz, $CDCl_3$) δ (ppm) 8.02 (d, *J* = 7.6 Hz, 1H), 7.91–7.80 (m, 4H), 7.64 (d, *J* = 7.5 Hz, 1H), 7.57–7.47 (m, 3H), 7.36 (td, *J* = 7.5, 1.2 Hz, 1H), 7.24 (td, *J* = 7.5, 1.2 Hz, 1H), 4.58 (s, 1H), 1.37 (s, 3H), 1.27 (s, 6H), 1.25 (s, 9H).

^{13}C-NMR (100 MHz, $CDCl_3$) δ (ppm) 158.7 (C), 147.8 (C), 144.8 (C), 137.0 (C), 133.1 (C), 132.9 (C), 128.1 (CH), 127.9 (CH), 127.8 (CH), 127.70 (CH), 127.66 (CH), 127.4 (CH), 126.4 (CH), 126.3 (CH), 126.0 (CH), 124.9 (CH), 122.3 (CH), 83.9 (2 x C), 73.2 (C), 64.5 (CH), 34.7 (CH_3), 28.2 (CH_3), 25.3 (2 x CH_3), 24.5 (2 x CH_3). Csp^2–B signal is not observed.

^{11}B-NMR (128 MHz, $CDCl_3$) δ 30.5.

HRMS (ESI-TOF) m/z: $[M+H]^+$ Calcd for $C_{28}H_{31}BClO_2$ 445.2100. Found 445.2111.

1-(2-Chloropropan-2-yl)-2-(3-thienyl)-3-(4,4,5,5-tetramethyl-1,3,2-dioxaborolan-2-yl)-1*H*-indene (2h)

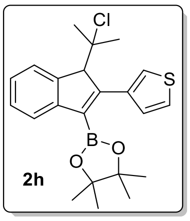

Obtained as brown oil (139 mg, 0.35 mmol, 87%) from **1h** (95 mg, 0.4 mmol). R_f = 0.33 (Hexane/EtOAc 30:1).

^{1}H-NMR (400 MHz, $CDCl_3$) δ (ppm) 7.85 (d, *J* = 7.6, 1H), 7.48 (d, *J* = 7.4, 1H), 7.26–7.18 (m, 3H), 7.11 (dd, *J* = 7.6, 1.3 Hz, 1H), 7.07 (dd, *J* = 5.0, 1.3 Hz, 1H), 4.21 (s, 1H), 1.32 (s, 3H), 1.22 (s, 12H), 1.11 (s, 3H).

^{13}C-NMR (100 MHz, $CDCl_3$) δ (ppm) 153.4 (C), 147.6 (C), 144.4 (C), 139.8 (C), 129.3 (CH), 127.7 (CH), 126.3 (CH), 124.7 (2 x CH), 123.3 (CH), 122.2 (CH), 83.9 (2 x C), 73.3 (C), 65.2 (CH), 34.2 (CH_3), 28.0 (CH_3), 25.1 (2 x CH_3), 24.8 (2 x CH_3). Csp^2–B signal is not observed.

^{11}B-NMR (128 MHz, $CDCl_3$) δ 30.2.

HRMS (ESI-TOF) m/z: $[M+H]^+$ Calcd for $C_{22}H_{27}BClO_2S$ 401.1508. Found 401.1512.

1-(2-Chloropropan-2-yl)-2-(cyclohex-1-en-1-yl)-3-(4,4,5,5-tetramethyl-1,3,2-dioxaborolan-2-yl)-1*H*-indene (2i)

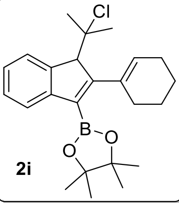
2i

Obtained as pale yellow oil (65 mg, 0.16 mmol, 41%) from **1i** (94 mg, 0.4 mmol) accompanied with indene **4i** (18%). The products could not be separated. R_f = 0.44 (Hexane/EtOAc 50:1). The following data has been extracted from the mixture.

^{1}H-NMR (400 MHz, CDCl$_3$) δ (ppm) 7.89–7.76 (m, 1H), 7.51–7.38 (m, 1H), 7.32–7.18 (m, 1H), 7.11 (td, *J* = 7.4, 1.2 Hz, 1H), 5.70 (bs, 1H), 4.06 (s, 1H), 2.60–2.37 (m, 1H), 2.28–2.03 (m, 4H), 1.75 (s, 3H), 1.75–1.47 (m, 4H), 1.34 (s, 2 x 6H), 1.30 (s, 3H).

^{13}C-NMR (100 MHz, CDCl$_3$) δ (ppm) 162.4 (C), 147.6 (C), 145.7 (C), 144.2 (C), 137.7 (C), 127.4 (CH), 126.2 (CH), 126.1 (CH), 124.1 (CH), 121.6 (CH), 83.4 (2 x C), 73.3 (C), 62.3 (CH), 31.8 (CH_3), 30.0 (CH_3), 28.4 (CH_2), 25.7 (CH_2), 25.3 (2 x CH_3), 24.5 (2 x CH_3), 22.8 (CH_2), 22.1 (CH_2). Csp^2–B signal is not observed.

^{11}B-NMR (128 MHz, CDCl$_3$) δ 30.7.

HRMS (ESI-TOF) m/z: [M+Na]$^+$ Calcd for $C_{24}H_{32}BClNaO_2$ 421.2076. Found 421.2074.

2-Butyl-1-(2-Chloropropan-2-yl)-3-(4,4,5,5-tetramethyl-1,3,2-dioxaborolan-2-yl)-1*H*-indene (2j)

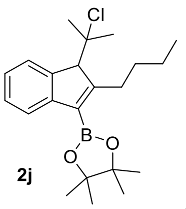
2j

Obtained as pale yellow oil (112 mg, 0.3 mmol, 76%) from **1j** (95 mg, 0.4 mmol) in a reaction conducted at −30 ºC in the presence of 2,4,6-tri-*tert*-butylpyridine (1 equiv, 0.4 mmol, 99 mg), formation of indene **4j** (16%) occurred during the purification. R_f = 0.50 (Hexane/EtOAc 50:1).

^{1}H-NMR (300 MHz, CDCl$_3$) δ (ppm) 7.72 (d, *J* = 7.3 Hz, 1H), 7.58 (d, *J* = 7.7 Hz, 1H), 7.32–7.21 (m, 1H), 7.08 (td, *J* = 7.5, 1.2 Hz, 1H), 3.94 (s, 1H), 3.25 (ddd, *J* = 13.3, 9.5, 6.8 Hz, 1H), 2.89 (ddd, *J* = 13.6, 9.1, 4.9 Hz, 1H), 1.83 (s, 3H), 1.77–1.66 (m, 1H), 1.50–1.30 (m, 3H), 1.39 (s, 12H), 1.38 (s, 3H), 0.94 (t, *J* = 7.3 Hz, 3H).

^{13}C-NMR (75 MHz, CDCl$_3$) δ (ppm) 167.6 (C), 148.2 (C), 143.5 (C), 127.5 (CH), 125.6 (CH), 123.5 (CH), 122.1 (CH), 83.2 (2 x C), 72.5 (C), 62.7 (CH), 33.7 (CH_3), 33.5 (CH_2), 31.1 (CH_2), 28.9 (CH_3), 25.2 (2 x CH_3), 25.0 (2 x CH_3), 22.8 (CH_2), 14.1 (CH_3). Csp^2–B signal is not observed.

^{11}B-NMR (128 MHz, CDCl$_3$) δ 30.2.

HRMS (ESI-TOF) m/z: [M+H]$^+$ Calcd for $C_{22}H_{33}BClO_2$ 375.2257. Found 375.2251.

1-(2-Chloropropan-2-yl)-2-cyclopropyl-3-(4,4,5,5-tetramethyl-1,3,2-dioxaborolan-2-yl)-1*H*-indene (2k)

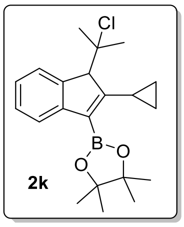

Obtained as yellow oil (123 mg, 0.34 mmol, 86%) from **1k** (78 mg, 0.4 mmol). R_f = 0.33 (Hexane/EtOAc 30:1).

^{1}H-NMR (400 MHz, $CDCl_3$) δ (ppm) 7.66 (d, *J* = 7.7 Hz, 1H), 7.45 (d, *J* = 7.5 Hz, 1H), 7.19–7.13 (m, 1H), 7.01 (td, *J* = 7.5, 1.3 Hz, 1H), 3.74 (s, 1H), 1.89–1.78 (m, 1H), 1.75 (s, 3H), 1.39 (s, 3H), 1.33 (s, 6H), 1.31 (s, 6H), 0.98–0.87 (m, 1H), 0.77–0.68 (m, 1H), 0.59–0.44 (m, 2H).

^{13}C-NMR (100 MHz, $CDCl_3$) δ (ppm) 163.7 (C), 147.7 (C), 144.0 (C), 127.3 (CH), 125.5 (CH), 124.0 (C), 121.6 (CH), 83.5 (2 x C), 72.8 (C), 65.1 (CH), 32.5 (CH_3), 29.6 (CH_3), 25.4 (2 x CH_3), 24.8 (2 x CH_3), 14.4 (CH), 11.1 (CH_2), 7.5 (CH_2). Csp^2–B signal is not observed.

^{11}B-NMR (128 MHz, $CDCl_3$) δ 30.4.

HRMS (ESI-TOF) m/z: $[M+H]^+$ Calcd for $C_{21}H_{29}BClO_2$ 359.1944. Found 359.1951.

1-(2-Chloropropan-2-yl)-2-methyl-3-(4,4,5,5-tetramethyl-1,3,2-dioxaborolan-2-yl)-1*H*-indene (2l)

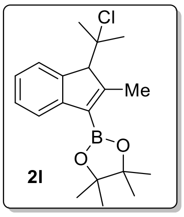

Pale yellow oil (113 mg, 0.34 mmol, 85%) from **1l** (68 mg, 0.4 mmol). R_f = 0.25 (Hexane/EtOAc 30:1).

^{1}H-NMR (400 MHz, $CDCl_3$) δ (ppm) 7.71 (d, *J* = 7.6 Hz, 1H), 7.60 (d, *J* = 7.6 Hz, 1H), 7.25 (t, *J* = 7.4 Hz, 1H), 7.07 (td, *J* = 7.5, 1.3 Hz, 1H), 3.76 (s, 1H), 2.50 (s, 3H), 1.76 (s, 3H), 1.44 (bs, 3H), 1.38 (s, 2 x 6H).

^{13}C-NMR (100 MHz, $CDCl_3$) δ (ppm) 162.3 (C), 148.2 (C), 143.3 (C), 127.5 (CH), 125.4 (CH), 123.6 (CH), 122.0 (CH), 83.2 (2 x C), 72.2 (C), 66.3 (CH), 32.9 (CH_3), 29.6 (CH_3), 25.2 (2 x CH_3), 25.1 (2 x CH_3), 19.5 (CH_3). Csp^2–B signal is not observed.

^{11}B-NMR (128 MHz, $CDCl_3$) δ 30.0.

HRMS (ESI-TOF) m/z: $[M+H]^+$ Calcd for $C_{19}H_{27}BClO_2$ 333.1787 Found 333.1795.

1-(2-Chloropropan-2-yl)-5-fluoro-2-phenyl-3-(4,4,5,5-tetramethyl-1,3,2-dioxaborolan-2-yl)-1*H*-indene (2m)

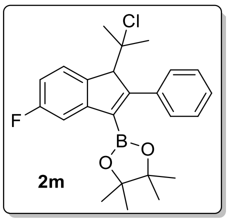

Pale yellow oil (99 mg, 0.24 mmol, 60%) from **1m** (100 mg, 0.4 mmol). R_f = 0.35 (Hexane/EtOAc 30:1).

^{1}H-NMR (300 MHz, $CDCl_3$) δ (ppm) 7.91 (dd, *J* = 8.4, 5.3 Hz, 1H), 7.41–7.22 (m, 6H), 6.88 (ddd, *J* = 9.3, 8.4, 2.6 Hz, 1H), 4.36 (s, 1H), 1.32 (s, 3H), 1.26 (s, 12H), 1.16 (s, 3H).

^{13}C-NMR (100 MHz, $CDCl_3$) δ (ppm) 163.1 (d, $^{1}J_{C-F}$ = 243.2 Hz, C), 161.5 (C), 149.6 (d, $^{3}J_{C-F}$ = 9.0 Hz, C), 140.1 (d, $^{4}J_{C-F}$ = 2.7 Hz, C), 138.9 (C), 129.2 (2 x CH), 128.0 (CH), 127.9 (2 x CH), 127.2 (d, $^{3}J_{C-F}$ = 8.7 Hz, CH), 111.2 (d, $^{2}J_{C-F}$ = 22.6 Hz, CH), 109.3 (d, $^{2}J_{C-F}$ = 22.9 Hz, CH), 84.0 (2 x C), 73.1 (C), 64.1 (CH), 34.8 (CH_3), 27.8 (CH_3), 25.2 (2 x CH_3), 24.5 (2 x CH_3). Csp^2–B signal is not observed.

^{11}B-NMR (128 MHz, $CDCl_3$) δ 30.3. **^{19}F-NMR** (376 MHz, $CDCl_3$) δ (ppm) –115.70.

HRMS (ESI-TOF) m/z: $[M+H]^+$ Calcd for $C_{24}H_{28}BClFO_2$ 413.1849 Found 413.1860.

1-(2-Chloropropan-2-yl)-6-fluoro-2-phenyl-3-(4,4,5,5-tetramethyl-1,3,2-dioxaborolan-2-yl)-1*H*-indene (2n)

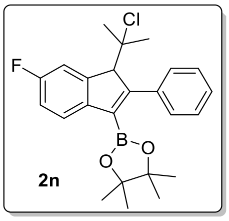

Yellow solid (128 mg, 0.31 mmol, 78%) from **1n** (100 mg, 0.4 mmol). R_f = 0.23 (Hexane/EtOAc 40:1). M.p.: 135−137 ºC.

^{1}H-NMR (300 MHz, CDCl$_3$) δ (ppm) 7.77 (dd, *J* = 9.9, 2.2 Hz, 1H), 7.53 (dd, *J* = 8.3, 5.4 Hz, 1H), 7.40–7.29 (m, 5H), 7.05 (td, *J* = 8.8, 2.5 Hz, 1H), 4.41 (s, 1H), 1.36 (s, 3H), 1.28 (s, 12H), 1.17 (s, 3H).

^{13}C-NMR (75 MHz, CDCl$_3$) δ (ppm) 161.0 (d, $^{1}J_{C\text{-}F}$ = 242.4 Hz, C), 158.8 (d, $^{5}J_{C\text{-}F}$ = 4.1 Hz, C), 146.8 (d, $^{3}J_{C\text{-}F}$ = 8.6 Hz, C), 143.6 (d, $^{4}J_{C\text{-}F}$ = 2.3 Hz, C), 139.0 (C), 129.1 (2 x CH), 127.9 (2 x CH), 127.8 (CH), 122.7 (d, $^{3}J_{C\text{-}F}$ = 8.6 Hz, CH), 114.40 (d, $^{2}J_{C\text{-}F}$ = 22.5 Hz, CH), 114.35 (d, $^{2}J_{C\text{-}F}$ = 24.6 Hz, CH), 83.9 (2 x C), 73.0 (C), 64.7 (d, $^{4}J_{C\text{-}F}$ = 2.3 Hz, CH), 35.1 (CH$_3$), 27.5 (CH$_3$), 25.2 (2 x CH$_3$), 24.5 (2 x CH$_3$). Csp^2−B signal is not observed.

^{11}B-NMR (96 MHz, CDCl$_3$) δ 30.1. **^{19}F-NMR** (471 MHz, CDCl$_3$) δ (ppm) –117.4.

HRMS (ESI-TOF) m/z: $[M+H]^+$ Calcd for $C_{24}H_{28}BClFO_2$ 413.1849 Found 413.1856.

6-Bromo-2-butyl-1-(2-Chloropropan-2-yl)-3-(4,4,5,5-tetramethyl-1,3,2-dioxaborolan-2-yl)-1*H*-indene (2o)

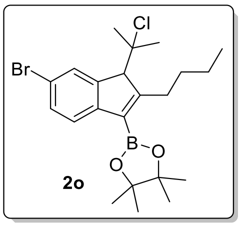

Obtained as yellow oil (139 mg, 0.31 mmol, 77%) from **1o** (116 mg, 0.4 mmol). R_f = 0.28 (Hexane/EtOAc 100:1).

^{1}H-NMR (300 MHz, CDCl$_3$) δ (ppm) 7.73 (d, *J* = 1.8 Hz, 1H), 7.59 (d, *J* = 8.1 Hz, 1H), 7.39 (dd, *J* = 8.1, 1.2 Hz, 1H), 3.91 (s, 1H), 3.24 (ddd, *J* = 13.4, 9.5, 6.8 Hz, 1H), 2.84 (td, *J* = 8.7, 4.5 Hz, 1H), 1.76 (s, 3H), 1.74–1.60 (m, 1H), 1.51–1.29 (m, 3H), 1.42 (s, 3H), 1.40 (s, 12H), 0.93 (t, *J* = 7.2 Hz, 3H).

^{13}C-NMR (75 MHz, CDCl$_3$) δ (ppm) 168.1 (C), 147.1 (C), 145.6 (C), 130.4 (CH), 128.7 (CH), 123.4 (CH), 117.6 (C), 83.3 (2 x C), 71.9 (C), 62.8 (CH), 33.4 (CH$_3$), 33.2 (CH$_2$), 31.1 (CH$_2$), 29.4 (CH$_3$), 25.2 (2 x CH$_3$), 24.9 (2 x CH$_3$), 22.8 (CH$_2$), 14.0 (CH$_3$). Csp^2−B signal is not observed.

^{11}B-NMR (128 MHz, CDCl$_3$) δ 29.3.

HRMS (APCI-TOF) m/z: $[M(-HCl)+H]^+$ Calcd for $C_{22}H_{31}BBrO_2$ 417.1595. Found 417.1610.

General procedure for the synthesis of borylated benzofulvenes 3.

Cold BCl_3 (1.0 M in heptane, 1.2 mL, 1.2 mmol, 3 equiv.) was added dropwise to a solution of the appropriate *o*-alkynylstyrene **1** (0.4 mmol, 1 equiv.) in anhydrous DCM (0.06 M) at 0 ºC under argon. The mixture was stirred for 2 h at 0 ºC and the formed **2a-BCl₂** intermediate was treated with a solution of pinacol (61.5 mg, 0.52 mmol, 1.3 equiv.) in Et_3N (0.83 mL, 6 mmol, 15 equiv.), the flask was sealed and the mixture was stirred for 16 h at 60 ºC. Then it was extracted with hexane and the organic layer washed with HCl (1 M, 3 x 10 mL) and brine, dried over anhydrous Na_2SO_4, filtered and concentrated under reduced pressure. The resulting residue was purified by column chromatography on silica gel using mixtures of hexane and EtOAc as eluents to give the corresponding benzofulvenes **3**.

Characterization data of borylated indenes 3

2-Phenyl-1-(propan-2-ylidene)-3-(4,4,5,5-tetramethyl-1,3,2-dioxaborolan-2-yl)-1*H*-indene (3a)

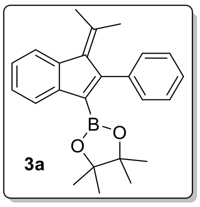

Obtained as yellow oil (115 mg, 0.32 mmol, 80%) from **1a** (93 mg, 0.4 mmol). The corresponding reaction using 232 mg (1 mmol) of **1a** afforded 280 mg of **3a** (78% yield). R_f = 0.38 (Hexane/EtOAc 30:1).

^{1}H-NMR (300 MHz, $CDCl_3$) δ (ppm) 7.73 (d, *J* = 7.3 Hz, 1H), 7.70–7.66 (m, 1H), 7.42–7.15 (m, 7H), 2.46 (s, 3H), 1.73 (s, 3H), 1.16 (s, 12H).

^{13}C-NMR (100 MHz, $CDCl_3$) δ (ppm). 153.5 (C), 147.8 (C), 145.1 (C), 141.1 (C), 137.8 (C), 137.1 (C), 129.3 (2 x CH), 127.5 (2 x CH), 126.6 (CH), 126.2 (CH), 124.6 (CH), 123.8 (CH), 122.1 (CH), 83.0 (2 x C), 26.4 (CH_3), 26.2 (CH_3), 24.6 (4 x CH_3). Csp^2–B signal is not observed.

^{11}B-NMR (128 MHz, $CDCl_3$) δ 30.5.

HRMS (ESI-TOF) m/z: $[M+H]^+$ Calcd for $C_{24}H_{28}BO_2$ 359.2177. Found 359.2186.

2-Phenyl-1-(propan-2-ylidene)-3-(4,4,5,5-tetraethyl-1,3,2-dioxaborolan-2-yl)-1*H*-indene (3a')

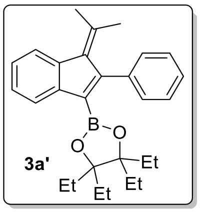

Obtained as yellow oil (132 mg, 0.32 mmol, 79%) from **1a** (93 mg, 0.4 mmol). R_f = 0.50 (Hexane/EtOAc 30:1).

^{1}H-NMR (400 MHz, $CDCl_3$) δ (ppm) 7.89 (ddd, *J* = 7.7, 1.5, 0.5 Hz, 1H), 7.76 (d, *J* = 7.7 Hz, 1H), 7.35–7.27 (m, 6H), 7.23 (td, *J* = 7.6, 1.5 Hz, 1H), 2.48 (s, 3H), 1.70 (s, 3H), 1.67–1.53 (m, 8H), 0.82 (t, *J* = 7.5 Hz, 12H).

^{13}C-NMR (100 MHz, $CDCl_3$) δ (ppm). 154.6 (C), 148.2 (C), 145.4 (C), 141.3 (C), 138.1 (C), 137.0 (C), 129.3 (2 x CH), 127.4 (2 x CH), 126.4 (CH), 126.3 (CH), 124.4 (CH), 123.7 (CH), 122.5 (CH), 87.9 (2 x C), 26.3 (CH_3) 26.2 (4 x CH_2), 26.1 (CH_3), 8.8 (4 x CH_3). Csp^2–B signal is not observed.

^{11}B-NMR (128 MHz, $CDCl_3$) δ 29.9.

HRMS (ESI-TOF) m/z: $[M+H]^+$ Calcd for $C_{28}H_{36}BO_2$ 415.2803. Found 415.2809.

2-(4-Methylphenyl)-1-(propan-2-ylidene)-3-(4,4,5,5-tetramethyl-1,3,2-dioxaborolan-2-yl)-1*H*-indene (3b)

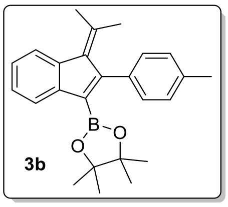

Obtained as orange solid (121 mg, 0.32 mmol, 81%) from **1b** (99 mg, 0.4 mmol). R_f = 0.33 (Hexane/EtOAc 30:1). M.p.: 75−77 ºC.

^{1}H-NMR (400 MHz, $CDCl_3$) δ (ppm) 7.62 (d, *J* = 7.6 Hz, 1H), 7.60–7.52 (m, 1H), 7.14 (td, *J* = 7.4, 1.2 Hz, 1H), 7.11–7.01 (m, 5H), 2.35 (s, 3H), 2.30 (s, 3H), 1.65 (s, 3H), 1.07 (s, 12H).

^{13}C-NMR (75 MHz, $CDCl_3$) δ (ppm). 153.5 (C), 147.6 (C), 145.2 (C), 138.0 (C), 137.9 (C), 137.1 (C), 136.0 (C), 129.2 (2 x CH), 128.2 (2 x CH), 126.2 (CH), 124.4 (CH), 123.7 (CH), 122.0 (CH), 82.9 (2 x C), 26.4 (CH_3), 26.1 (CH_3), 24.7 (4 x CH_3), 21.3 (CH_3). Csp^2−B signal is not observed.

^{11}B-NMR (128 MHz, $CDCl_3$) δ 30.3.

HRMS (ESI-TOF) m/z: $[M+H]^+$ Calcd for $C_{25}H_{30}BO_2$ 373.2333. Found 373.2349.

2-(4-Methoxyphenyl)-1-(propan-2-ylidene)-3-(4,4,5,5-tetramethyl-1,3,2-dioxaborolan-2-yl)-1*H*-indene (3c)

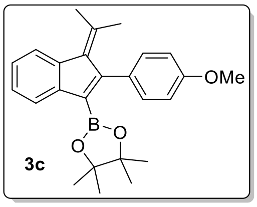

Obtained as orange solid (132 mg, 0.34 mmol, 85%) from **1c** (105 mg, 0.4 mmol). R_f = 0.15 (Hexane/EtOAc 40:1). M.p.: 90−93 ºC.

^{1}H-NMR (400 MHz, $CDCl_3$) δ (ppm) 7.71 (d, *J* = 7.7 Hz, 1H), 7.66 (ddt, *J* = 7.1, 1.6, 0.8 Hz, 1H), 7.28–7.12 (m, 5H), 6.87 (d, *J* = 8.8 Hz, 2H), 3.85 (s, 3H), 2.45 (s, 3H), 1.74 (s, 3H), 1.17 (s, 12H).

^{13}C-NMR (100 MHz, $CDCl_3$) δ (ppm). 158.6 (C), 153.2 (C), 147. 6 (C), 145.2 (C), 137.9 (C), 137.1 (C), 133.4 (C), 130.4 (2 x CH), 126.2 (CH), 124.4 (CH), 123.7 (CH), 122.0 (CH), 113.0 (2 x CH), 82.9 (2 x C), 55.30 (CH_3), 26.3 (CH_3), 26.2 (CH_3), 24.7 (4 x CH_3). Csp^2−B signal is not observed.

^{11}B-NMR (128 MHz, $CDCl_3$) δ 30.9.

HRMS (ESI-TOF) m/z: $[M+H]^+$ Calcd for $C_{25}H_{30}BO_3$ 389.2283. Found 389.2293.

2-(4-Chlorophenyl)-1-(propan-2-ylidene)-3-(4,4,5,5-tetramethyl-1,3,2-dioxaborolan-2-yl)-1*H*-indene (3d)

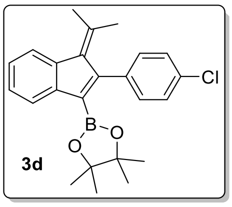

Obtained as orange solid (119 mg, 0.30 mmol, 76%) from **1d** (106 mg, 0.4 mmol). R_f = 0.30 (Hexane/EtOAc 30:1). M.p.: 80−82 ºC.

^{1}H-NMR (400 MHz, $CDCl_3$) δ (ppm) 7.66–7.60 (m, 2H), 7.23 (d, *J* = 8.5 Hz, 2H), 7.09–7.09 (m, 4H), 2.38 (s, 3H), 1.65 (s, 3H), 1.08 (s, 12H).

^{13}C-NMR (100 MHz, $CDCl_3$) δ (ppm). 152.1 (C), 148.0 (C), 144.9 (C), 139.7 (C), 137.7 (C), 137.0 (C), 132.5 (C), 130.8 (2 x CH), 127.6 (2 x CH), 126.3 (CH), 124.7 (CH), 123.8 (CH), 122.3 (CH), 83.0 (2 x C), 26.5 (CH_3), 26.1 (CH_3), 24.7 (4 x CH_3). Csp^2−B signal is not observed.

^{11}B-NMR (128 MHz, $CDCl_3$) δ 30.3.

HRMS (ESI-TOF) m/z: $[M+H]^+$ Calcd for $C_{24}H_{27}BClO_2$ 393.1787. Found 393.1790.

2-(3-Chlorophenyl)-1-(propan-2-ylidene)-3-(4,4,5,5-tetramethyl-1,3,2-dioxaborolan-2-yl)-1*H*-indene (3e)

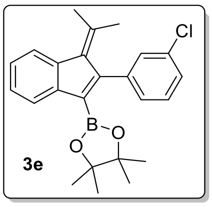

Obtained as yellow oil (122 mg, 0.31 mmol, 78%) from **1e** (106 mg, 0.4 mmol). R_f = 0.41 (Hexane/EtOAc 30:1).

^{1}H-NMR (300 MHz, CDCl$_3$) δ (ppm) 7.76–7.66 (m, 2H), 7.33–7.14 (m, 6H), 2.45 (s, 3H), 1.76 (s, 3H), 1.18 (s, 12H).

^{13}C-NMR (100 MHz, CDCl$_3$) δ (ppm). 151.9 (C), 148.1 (C), 144.9 (C), 143.0 (C), 137.7 (C), 137.0 (C), 133.4 (C), 129.6 (CH), 128.8 (CH), 127.8 (CH), 126.7 (CH), 126.4 (CH), 124.9 (CH), 123.9 (CH), 122.4 (CH), 83.2 (2 x C), 26.8 (CH_3), 26.2 (CH_3), 24.9 (2 x CH_3), 24.7 (2 x CH_3). Csp^2–B signal is not observed.

^{11}B-NMR (128 MHz, CDCl$_3$) δ 30.3.

HRMS (ESI-TOF) m/z: $[M+H]^+$ Calcd for $C_{24}H_{27}BClO_2$ 393.1787. Found 393.1793.

1-(Propan-2-ylidene)-3-(4,4,5,5-tetramethyl-1,3,2-dioxaborolan-2-yl)-2-(thiophen-3-yl)-1*H*-indene (3h)

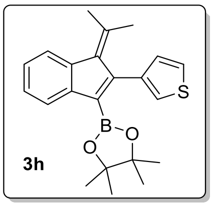

Obtained as brown oil (73 mg, 0.20 mmol, 50%) from **1h** (95 mg, 0.4 mmol). R_f = 0.28 (Hexane/EtOAc 30:1).

^{1}H-NMR (400 MHz, CDCl$_3$) δ (ppm) 7.72 (d, *J* = 7.4 Hz, 1H), 7.66 (ddd, *J* = 7.4, 1.5, 0.7 Hz, 1H), 7.28 (dd, *J* = 4.9, 3.0 Hz, 1H), 7.23 (dd, *J* = 7.3, 1.3 Hz, 1H), 7.19 (td, *J* = 7.4, 1.5 Hz, 1H), 7.11 (dd, *J* = 3.0, 1.2 Hz, 1H), 7.01 (dd, *J* = 4.9, 1.2 Hz, 1H), 2.46 (s, 3H), 1.81 (s, 3H), 1.20 (s, 12H).

^{13}C-NMR (100 MHz, CDCl$_3$) δ (ppm). 147.93 (C), 147.88 (C), 145.0 (C), 140.8 (C), 137.9 (C), 137.2 (C), 129.8 (CH), 126.3 (CH), 124.7 (CH), 124.2 (CH), 123.9 (CH), 122.4 (CH), 122.2 (CH), 83.1 (2 x C), 26.2 (CH_3), 25.7 (CH_3), 24.8 (4 x CH_3). Csp^2–B signal is not observed.

^{11}B-NMR (128 MHz, CDCl$_3$) δ 30.5.

HRMS (ESI-TOF) m/z: $[M+H]^+$ Calcd for $C_{22}H_{26}BO_2S$ 365.1741. Found 365.1746.

2-(Cyclohex-1-en-1-yl)-1-(propan-2-ylidene)-3-(4,4,5,5-tetramethyl-1,3,2-dioxaborolan-2-yl)-1*H*-indene (3i)

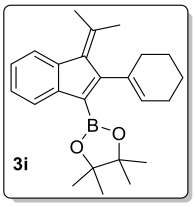

Obtained as orange oil (98 mg, 0.27 mmol, 68%) from **1i** (94 mg, 0.4 mmol). R_f = 0.43 (Hexane/EtOAc 50:1).

^{1}H-NMR (400 MHz, CDCl$_3$) δ (ppm) 7.60–7.52 (m, 2H), 7.10 (td, *J* = 7.4, 1.0 Hz, 1H), 7.03 (td, *J* = 7.5, 1.3 Hz, 1H), 5.47 (m, 1H), 2.36 (s, 3H), 2.27 (s, 3H), 2.21–1.99 (m, 3H), 1.82–1.48 (m, 5H), 1.24 (s, 12H).

^{13}C-NMR (100 MHz, CDCl$_3$) δ (ppm). 156.9 (C), 146.4 (C), 145.3 (C), 138.3 (C), 137.0 (C), 136.9 (C), 126.1 (CH), 124.4 (CH), 124.0 (CH), 123.7 (CH), 121.8 (CH), 82.8 (2 x C), 30.9 (kCH_2), 25.8 (CH_3), 25.7 (CH_3), 25.2 (2 x CH_3), 24.7 (CH_2), 24.6 (2 x CH_3), 23.2 (CH_2), 22.0 (CH_2). Csp^2–B signal is not observed.

^{11}B-NMR (128 MHz, CDCl$_3$) δ 30.4.

HRMS (ESI-TOF) m/z: $[M+H]^+$ Calcd for $C_{24}H_{32}BO_2$ 363.2490. Found 363.2507.

2-(Butyl)-1-(propan-2-ylidene)-3-(4,4,5,5-tetramethyl-1,3,2-dioxaborolan-2-yl)-1*H*-indene (3j)

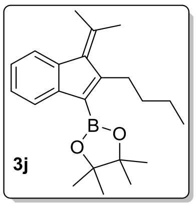

Obtained as yellow oil (101 mg, 0.30 mmol, 75%) from **1j** (95 mg, 0.4 mmol). R_f = 0.43 (Hexane/EtOAc 50:1). Purified with deactivated silica gel to avoid deborylation.

^{1}H-NMR (300 MHz, CDCl$_3$) δ (ppm) 7.80 (d, *J* = 7.4 Hz, 1H), 7.66 (d, *J* = 7.7 Hz, 1H), 7.21 (tt, *J* = 7.5, 1.2 Hz, 1H), 7.12 (td, *J* = 7.4, 1.2 Hz, 1H), 3.10–3.04 (m, 2H), 2.52 (s, 3H), 2.41 (s, 3H), 1.62–1.40 (m, 4H), 1.39 (s, 12H), 0.98 (t, *J* = 7.2 Hz, 3H).

^{13}C-NMR (100 MHz, CDCl$_3$) δ (ppm). 157.5 (C), 145.7 (C), 145.4 (C), 137.9 (C), 137.5 (C), 126.0 (CH), 123.8 (CH), 123.6 (CH), 121.7 (CH), 82.7 (2 x C), 33.9 (CH_2), 30.3 (CH_2), 27.2 (CH_3), 25.2 (CH_3), 25.0 (4 x CH_3), 22.9 (CH_2), 13.9 (CH_3). Csp^2–B signal is not observed.

^{11}B-NMR (128 MHz, CDCl$_3$) δ 30.4.

HRMS (ESI-TOF) m/z: $[M+H]^+$ Calcd for $C_{22}H_{32}BO_2$ 339.2490. Found 339.2491.

2-(Cyclopropyl)-1-(propan-2-ylidene)-3-(4,4,5,5-tetramethyl-1,3,2-dioxaborolan-2-yl)-1*H*-indene (3k)

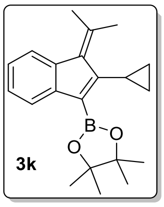

Obtained as orange oil (91 mg, 0.28 mmol, 71%) from **1k** (78 mg, 0.4 mmol). R_f = 0.30 (Hexane/EtOAc 30:1).

^{1}H-NMR (400 MHz, CDCl$_3$) δ (ppm) 7.56 (d, *J* = 7.6 Hz, 1H), 7.50–7.48 (m, 1H), 7.11–7.01 (m, 2H), 2.49 (s, 3H), 2.39 (s, 3H), 1.89 (tt, *J* = 8.4, 5.7 Hz, 1H), 1.33 (s, 12H), 0.91–0.87 (m, 2H), 0.54–0.50 (m, 2H).

^{13}C-NMR (100 MHz, CDCl$_3$) δ (ppm). 155.5 (C), 145.4 (C), 145.1 (C), 138.4 (C), 137.3 (C), 125.9 (CH), 124.1 (CH), 123.6 (CH), 121.3 (CH), 83.3 (2 x C), 26.4 (CH_3), 25.9 (CH_3), 25.0 (4 x CH_3), 14.7 (CH), 9.87 (2 x CH_2). Csp^2–B signal is not observed.

^{11}B-NMR (128 MHz, CDCl$_3$) δ 31.1.

HRMS (ESI-TOF) m/z: $[M+H]^+$ Calcd for $C_{21}H_{28}BO_2$ 323.2177. Found 323.2192.

2-(Methyl)-1-(propan-2-ylidene)-3-(4,4,5,5-tetramethyl-1,3,2-dioxaborolan-2-yl)-1*H*-indene (3l)

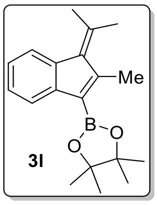

Obtained as yellow oil (97 mg, 0.33 mmol, 82%) from **1l** (68 mg, 0.4 mmol). R_f = 0.38 (Hexane/EtOAc 30:1). Purified with deactivated silica gel to avoid deborylation.

^{1}H-NMR (400 MHz, CDCl$_3$) δ (ppm) 7.80 (d, *J* = 7.5 Hz, 1H), 7.65 (d, *J* = 7.7 Hz, 1H), 7.21 (td, *J* = 7.5, 1.2 Hz, 1H), 7.11 (td, *J* = 7.7, 1.2 Hz, 1H), 2.62 (s, 3H), 2.50 (s, 3H), 2.40 (s, 3H), 1.39 (s, 12H).

^{13}C-NMR (100 MHz, CDCl$_3$) δ (ppm). 152.7 (C), 146.1 (C), 145.9 (C), 139.0 (C), 137.3 (C), 126.2 (CH), 123.7 (CH), 123.6 (CH), 121.7 (CH), 82.9 (2 x C), 27.0 (CH_3), 25.8 (CH_3), 25.1 (4 x CH_3), 19.4 (CH_3). Csp^2–B signal is not observed.

^{11}B-NMR (128 MHz, CDCl$_3$) δ 30.5.

HRMS (ESI-TOF) m/z: $[M+H]^+$ Calcd for $C_{19}H_{26}BO_2$ 297.2020. Found 297.2028.

5-Fluoro-2-phenyl-1-(propan-2-ylidene)-3-(4,4,5,5-tetramethyl-1,3,2-dioxaborolan-2-yl)-1*H*-indene (3m)

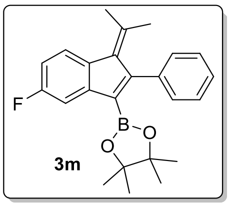

Obtained as yellow solid (125 mg, 0.33 mmol, 83%) from **1m** (100 mg, 0.4 mmol). R_f = 0.43 (Hexane/EtOAc 30:1). M.p.: 114−115 ºC.

^{1}H-NMR (300 MHz, CDCl$_3$) δ (ppm) 7.66 (dd, J = 8.6, 5.1 Hz, 1H), 7.44 (dd, J = 9.3, 2.6 Hz, 1H), 7.37–7.26 (m, 5H), 6.89 (td, J = 8.9, 2.6 Hz, 1H), 2.44 (s, 3H), 1.73 (s, 3H), 1.18 (s, 12H).

^{13}C-NMR (75 MHz, CDCl$_3$) δ (ppm). 162.0 (d, $^{1}J_{C\text{-}F}$ = 242.8 Hz, C), 155.7 (C), 148.1 (d, $^{5}J_{C\text{-}F}$ = 1.9 Hz, C), 147.2 (d, $^{3}J_{C\text{-}F}$ = 9.1 Hz, C), 140.8 (C), 137.0 (C), 133.0 (d, $^{4}J_{C\text{-}F}$ = 2.4 Hz, C), 129.2 (2 x CH), 127.6 (2 x CH), 126.8 (CH), 124.5 (d, $^{3}J_{C\text{-}F}$ = 8.8 Hz, CH), 110.8 (d, $^{2}J_{C\text{-}F}$ = 22.6 Hz, CH), 109.12 (d, $^{2}J_{C\text{-}F}$ = 22.7 Hz, CH), 83.1 (2 x C), 26.3 (CH$_3$), 26.2 (CH$_3$), 24.7 (4 x CH$_3$). Csp^2−B signal is not observed.

^{11}B-NMR (160 MHz, CDCl$_3$) δ 30.2.

HRMS (ESI-TOF) m/z: [M+H]$^+$ Calcd for $C_{24}H_{27}BFO_2$ 377.2083. Found 377.2087.

6-Bromo-2-(butyl)-1-(propan-2-ylidene)-3-(4,4,5,5-tetramethyl-1,3,2-dioxaborolan-2-yl)-1*H*-indene (3o)

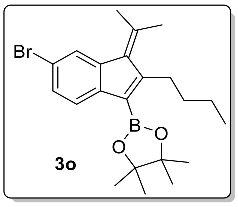

Obtained as yellow oil (138 mg, 0.33 mmol, 83%) from **1o** (116 mg, 0.4 mmol). R_f = 0.18 (Hexane/EtOAc 40:1).

^{1}H-NMR (300 MHz, CDCl$_3$) δ (ppm) 7.76 (d, J = 1.8 Hz, 1H), 7.65 (d, J = 8.1 Hz, 1H), 7.31 (dd, J = 8.1, 1.8 Hz, 1H), 3.11–2.96 (m, 2H), 2.49 (s, 3H), 2.41 (s, 3H), 1.56–1.35 (m, 4H), 1.37 (s, 12H), 0.97 (t, J = 7.2 Hz, 3H).

^{13}C-NMR (100 MHz, CDCl$_3$) δ (ppm). 158.1 (C), 147.6 (C), 144.6 (C), 139.4 (C), 137.4 (C), 128.9 (CH), 126.9 (CH), 123.0 (CH), 117. 7 (C), 83.0 (2 x C), 33.9 (CH$_2$), 30.4 (CH$_2$), 27.4 (CH$_3$), 25.4 (CH$_3$), 25.1 (4 x CH$_3$), 23.0 (CH$_2$), 14.1 (CH$_3$). Csp^2−B signal is not observed.

^{11}B-NMR (160 MHz, CDCl$_3$) δ 29.9.

HRMS (ESI-TOF) m/z: [M+H]$^+$ Calcd for $C_{22}H_{31}BBrO_2$ 417.1595. Found 417.1593.

1-(Benzylidene)-2-(phenyl)-3-(4,4,5,5-tetramethyl-1,3,2-dioxaborolan-2-yl)-1*H*-indene (3p)

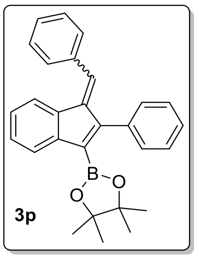

Obtained as orange solid as a 2/1 mixture of *Z*/*E* isomers (136 mg, 0.34 mmol, 84%) from **1p** (112 mg, 0.4 mmol). R_f = 0.21 (Hexane/EtOAc 30:1). M.p.: 92−94 ºC.

^{1}H-NMR (400 MHz, CDCl$_3$) δ (ppm) 7.79 (s, 1H, min), 7.72–7.64 (m, 2H, maj + min), 7.61 (dt, J = 7.4, 1.0 Hz, 1H, min), 7.55–7.48 (m, 4H, maj + min), 7.44–7.35 (m, 10H, maj + min), 7.30–7.16 (m, 2H, maj + min), 7.13 (bs, 1H, maj), 7.09–7.05 (m, 1H, maj + min), 7.05–6.87 (m, 8H, maj + min), 1.24 (s, 12H, maj), 1.23 (s, 12H, min).

^{13}C-NMR (100 MHz, CDCl$_3$) δ (ppm). 155.0 (C, maj), 150.4 (C, min), 146.6 (C, maj), 144.5 (C, min), 142.7 (C, maj), 140.2 (C, min), 138.7 (C, min), 137.7 (C, min), 136.8 (C,

maj), 136.29 (C, maj), 136.26 (CH, maj), 135.4 (C, min), 134.3 (C, maj), 131.9 (CH, min), 130.7 (2 x CH, maj), 130.3 (2 x CH, min), 129.9 (2 x CH, min), 129.4 (2 x CH, maj), 128.4 (2 x CH, maj), 128.33 (2 x CH, min), 128.31 (CH, maj), 127.7 (CH, min), 127.33 (2 x CH, maj), 127.32 (2 x CH, min), 127.2 (CH, min), 127.1 (CH. maj), 126.8 (CH, maj), 126.3 (CH, min), 124.9 (CH, min), 124.6 (CH, maj), 122.9 (CH, maj), 122.2 (CH, maj), 122.0 (CH, min), 118.7 (CH, min), 83.5 (2 x C, min), 83.3 (2 x C, maj), 24.7 (8 x CH_3, maj + min). Csp^2–B signal is not observed.

^{11}B-NMR (128 MHz, $CDCl_3$) δ 30.5.

HRMS (ESI-TOF) m/z: $[M+H]^+$ Calcd for $C_{28}H_{28}BO_2$ 407.2177. Found 407.2189.

1-Diphenylmethylene-2-(phenyl)-3-(4,4,5,5-tetramethyl-1,3,2-dioxaborolan-2-yl)-1*H*-indene (3q)

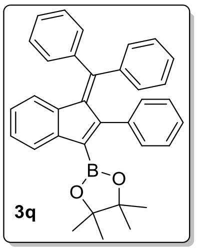

Obtained as orange solid (175 mg, 0.36 mmol, 91%) from **1q** (142 mg, 0.4 mmol) using 4 equiv. of BCl_3 and in the presence of 2,4,6-tri-*tert*-butylpyridine (1 equiv, 0.4 mmol, 99 mg). R_f = 0.25 (Hexane/EtOAc 50:1). M.p.: 205−207 ºC.

^{1}H-NMR (400 MHz, $CDCl_3$) δ (ppm) 7.56 (d, *J* = 7.4 Hz, 1H), 7.51–7.39 (m, 3H), 7.38–7.34 (m, 2H), 7.13 (td, *J* = 7.4, 1.1 Hz, 1H), 7.08–7.01 (m, 2H), 6.94–6.76 (m, 9H), 6.35 (d, *J* = 7.8 Hz, 1H), 1.22 (s, 12H).

^{13}C-NMR (100 MHz, $CDCl_3$) δ (ppm). 152.9 (C), 150.9 (C), 145.7 (C), 143.4 (C), 141.4 (C), 139.8 (C), 138.4 (C), 138.2 (C), 132.5 (2 x CH), 131.1 (2 x CH), 130.3 (2 x CH), 129.0 (CH), 128.7 (2 x CH), 127.9 (CH), 127.2 (CH), 127.0 (2 x CH), 126.7 (2 x CH), 125.7 (CH), 124.6 (CH), 123.4 (CH), 121.8 (CH), 83.5 (2 x C), 24.8 (4 x CH_3). Csp^2–B signal is not observed.

^{11}B-NMR (128 MHz, $CDCl_3$) δ 31.1.

HRMS (ESI-TOF) m/z: $[M+H]^+$ Calcd for $C_{34}H_{32}BO_2$ 483.2490. Found 483.2500.

1-Cyclopentylidene-2-(phenyl)-3-(4,4,5,5-tetramethyl-1,3,2-dioxaborolan-2-yl)-1*H*-indene (3r)

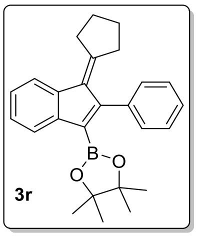

Obtained as orange oil (69 mg, 0.18 mmol, 45%), accompanied with indene **4r** (11%), from **1r** (103 mg, 0.4 mmol). R_f = 0.15 (Hexane/EtOAc 100:1).

^{1}H-NMR (300 MHz, $CDCl_3$) δ (ppm) 7.74–7.65 (m, 1H), 7.65 (d, *J* = 7.1 Hz, 1H), 7.36–7.23 (m, 7H), 3.16–3.02 (m, 2H), 2.19–2.11 (m, 2H), 1.81 (aqui, *J* = 7.0 Hz, 2H), 1.60–1.49 (m, 2H) 1.19 (s, 12H).

^{13}C-NMR (75 MHz, $CDCl_3$) δ (ppm). 159.0 (C), 154.3 (C), 145.3 (C), 140.6 (C), 137.2 (C), 129.4 (2 x CH), 127.6 (2 x CH), 126.8 (CH), 126.2 (CH), 124.6 (CH), 123.2 (CH), 122.3 (CH), 83.0 (2 x C), 35.8 (CH_2), 35.7 (CH_2), 26.9 (CH_2), 26.1 (CH_2), 24.7 (4 x CH_3). Csp^2–B signal is not observed.

^{11}B-NMR (96 MHz, $CDCl_3$) δ 30.2.

HRMS (APCI-TOF) m/z: $[M+H]^+$ Calcd for $C_{26}H_{30}BO_2$ 385.2333. Found 385.2343.

Synthetic procedures and characterization data for the derivatizations of borylated benzofulvene 3a

Synthesis of trifluoroborate salt 6a:[2] To a solution of boronate **3a** (42 mg, 0.12 mmol, 1 equiv.) in MeOH/H_2O (5/1, 1.0 mL / 0.4 mL) was added KHF_2 (46 mg, 0.60 mmol, 5 equiv.). The resulting mixture was stirred under reflux for 3 h. Then, all volatiles were removed under reduced pressure and the residue was extracted with hot acetonitrile (3 x 10 mL) and the solution was concentrated to dryness. The residue was washed with Et_2O to give pure trifluoroborate salt **6a** as a white solid (36 mg, 0.11 mmol, 90%).

Potassium trifluoro(2-phenyl-1-(propan-2-ylidene)-1*H*-inden-3-yl)-borate (6a)

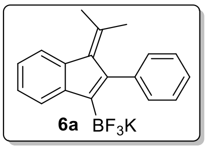

R_f = 0.32 (DCM/MeOH 9:1). M.p.: Decomp. (225 ºC).

^{1}H-NMR (400 MHz, Acetone-d$_6$) δ (ppm) 7.68 (d, J = 7.4 Hz, 1H), 7.50 (d, J = 7.5 Hz, 1H), 7.19–7.11 (m, 4H), 7.08–7.04 (m, 1H), 6.96 (td, J = 7.4, 1.3 Hz, 1H), 6.90 (td, J = 7.5, 1.3 Hz, 1H), 2.23 (s, 3H), 1.39 (s, 3H).

^{13}C-NMR (100 MHz, Acetone-d$_6$) δ (ppm) 149.0 (C), 144.2 (C), 143.7 ($^nJ_{C-F}$ = 3.2 Hz, C), 139.4 (C), 138.2 (C), 138.1 (C), 130.1 (2 x CH), 126.9 (2 x CH), 125.1 (CH), 125.0 (CH), 123.5 ($^4J_{C-F}$ = 2.3 Hz, CH), 122.8 (CH), 122.7 (CH), 25.0 (CH_3), 24.9 (CH_3). Csp^2–B signal is not observed.

^{11}B-NMR (128 MHz, $CDCl_3$) δ 3.1. **^{19}F-NMR** (376 MHz, $CDCl_3$) δ (ppm) –133.93.

HRMS (ESI-TOF) m/z: $[M+H]^+$ Calcd for $C_{18}H_{16}BF_3K$ 339.0934. Found 339.0942.

Suzuki reaction:[3] In a schenk flask under argon atmosphere, 4-iodoanisole (36 mg, 0.08 mmol, 1.5 equiv.), boronic ester **3a** (40 mg, 0.1 mmol, 1 equiv.), Ag_2O (34 mg, 0.08 mmol, 1.5 equiv.), $Pd_2(dba)_3$ (5 mg, 0.005 mmol, 5 mol%), and PPh_3 (26 mg, 0.1 mmol, 1 equiv.) were dissolved in THF (0.06 M, 2 mL) and the reaction mixture was stirred at 66 ºC for 24 h. Water and CH_2Cl_2 were added to the cooled reaction mixture. The separated aqueous phase was extracted three times with CH_2Cl_2. The combined organic layers were dried over anhydrous Na_2SO_4, filtered, and evaporated under reduced pressure. The residue was purified by flash chromatography using a 30:1 mixture of hexane/EtOAc as eluent to obtain **7a**.

3-(4-Methoxyphenyl)-2-phenyl-1-(propan-2-ylidene)-1*H*-indene (7a)

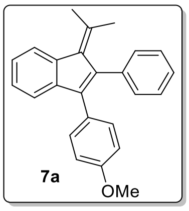

Obtained as a colorless oil (24 mg, 0.07 mmol, 71%) from **3a** (40 mg, 0.10 mmol). R_f = 0.27 (Hexane/EtOAc 30:1).

^{1}H-NMR (400 MHz, $CDCl_3$) δ (ppm) 7.91–7.70 (m, 1H), 7.25–7.08 (m, 8H), 7.02 (d, J = 8.8 Hz, 2H), 6.69 (d, J = 8.8 Hz, 2H), 3.69 (s, 3H), 2.42 (s, 3H), 1.64 (s, 3H).

^{13}C-NMR (100 MHz, $CDCl_3$) δ (ppm) 158.2 (C), 145.4 (C), 142.7 (C), 140.1 (C), 139.4 (C), 138.7 (C), 136.7 (C), 136.2 (C), 130.9 (2 x CH), 130.3 (2 x CH), 128.0 (2 x CH), 127.4 (C), 126.4 (CH), 126.0 (CH), 125.1 (CH), 124.0 (CH), 120.1 (CH), 113.3 (2 x CH), 55.1 (CH_3), 26.3 (CH_3), 26.1 (CH_3).

HRMS (ESI-TOF) m/z: $[M+H]^+$ Calcd for $C_{25}H_{23}O$ 339.1743. Found 339.1744.

[2] Fawcett, A.; Biberger, T.; Aggarwal, V. K. *Nat. Chem.* **2019**, *11*, 117−122.

[3] Imao, D.; Glasspoole, B. W.; Laberge, V. S.; Crudden, C. M. *J. Am. Chem. Soc.* **2009**, *131*, 5024–5025.

Protodeboronation:[2] A solution of TBAF (49 µL, 0.17 mmol, 1.5 equiv.) and boronic ester **3a** (42 mg, 0.11 mmol, 1 equiv.) in THF (1.0 mL) was stirred in a schlenk flask under argon atmosphere at 45 ºC for 3 h. Then, the reaction mixture was quenched with water and extracted with Et_2O. The combined organic phases were dried (Na_2SO_4), filtered, and concentrated under reduced pressure. The crude residue was purified by flash column chromatography using a 40:1 mixture of hexane and CH_2Cl_2 as eluent to give **8a** (65% yield). The spectroscopic data were consistent with previously reported.[4]

Oxidation:[2] A 2:1 degassed mixture of NaOH (3 M in H_2O, 1.5 mL) and H_2O_2 (30% in H_2O, 0.5 mL) was added drop-wise at 0 ºC to a solution of boronic ester **3a** (40 mg, 0.1 mmol, 1 equiv.) in THF (0.1 M, 1 mL). The resulting mixture was subsequently warmed to ambient temperature and allowed to react for 3 h. Then, it was quenched with NH_4Cl (sat) and extracted three times with dichloromethane. The combined organic layers were dried over Na_2SO_4, filtered, and concentrated under reduced pressure. The crude residue was purified by flash chromatography using a 30:1 mixture of hexane/EtOAc as eluent to obtain **9a**. The spectroscopic data were consistent with previously reported.[5]

Synthesis of Sulindac:

Synthesis of substrate 1s:

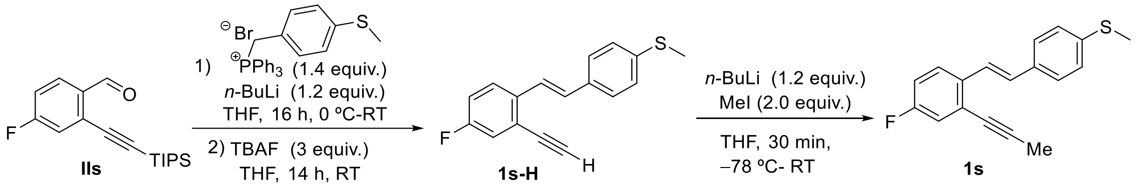

Step 1: *n*-BuLi (3.40 mL, 5.45 mmol, 1.2 equiv., 1.6 M in hexanes) was added to a solution of (4-(methylthio)benzyl)triphenylphosphonium bromide (3.05 g, 6.36 mmol, 1.4 equiv.) in THF (2 M) at 0 ºC and the resulting mixture was stirred for 30 min at RT. A solution of **IIs** (1.38 g, 4.54 mmol, 1 equiv.) in THF (1M) was added at 0 ºC and the reaction stirred at RT until **IIs** was consumed as determined by TLC. The crude mixture was partitioned between water and DCM and the solvents were removed. The residue was then dissolved in THF (0.25 M), TBAF (13.62 mL, 13.62 mmol, 3 equiv., 1.0 M in THF) was added and the reaction was stirred at RT until consumption of the substrate as monitored by TLC. The crude mixture was partitioned between water and DCM and the solvents were removed. The crude was purified by flash chromatography on silica gel using Hexane/EtOAc 95:5 as eluent to obtain *o*-(alkynyl)styrene **1s-H**.

4-Fluoro-2-((triisopropylsilyl)ethynyl)benzaldehyde (IIs)

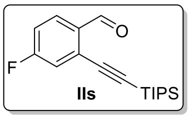

Obtained as brown oil (1.38 g, 4.53 mmol, 76%) from **Is** (1.22 g, 6 mmol) following step 1 of method A. R_f = 0.67 (Hexane/EtOAc 95:5).

^{1}H-NMR (400 MHz, $CDCl_3$) δ (ppm) 10.52 (d, J = 0.9 Hz, 1H), 7.94 (dd, J = 8.7, 5.9 Hz, 1H), 7.25 (dd, J = 8.7, 2.6 Hz, 1H), 7.12 (m, 1H), 1.23–1.05 (m, 21H).

^{13}C-NMR (100 MHz, $CDCl_3$) δ (ppm) 190.2 (C), 165.8 (C, d, $^1J_{C\text{-}F}$ = 257.0 Hz), 133.1 (C, d, $^4J_{C\text{-}F}$ = 2.9 Hz), 129.9 (CH, d, $^3J_{C\text{-}F}$ = 10.2 Hz), 129.6 (C, d, $^3J_{C\text{-}F}$ = 10.9 Hz), 120.5

[4] Akhmetov, V.; Feofanov, M.; Sharapa, D. I.; Amsharov, K. *J. Am. Chem. Soc.* **2021**, *143*, 15420–15426.
[5] Miao, M.; Ren, H.; Xu, H.; Luo, Y.; Jin, M.; Chen, Z.; Xu, J. *Synthesis* **2018**, *50*, 349–360.

(CH, d, $^{2}J_{C\text{-}F}$ = 23.3 Hz), 116.8 (CH, d, $^{2}J_{C\text{-}F}$ = 22.2 Hz), 101.0 (C), 100.8 (C, d, $^{4}J_{C\text{-}F}$ = 2.7 Hz), 18.8 (6 x CH_3), 11.3 (3 x CH).

^{19}F-NMR (376 MHz, CDCl$_3$) δ (ppm) –103.44.

HRMS (ESI-TOF) m/z: $[M+H]^+$ Calcd for $C_{18}H_{26}FOSi$ 305.1731. Found 305.1735.

(*E*)-2-Ethynyl-4-fluoro-1-(4-methylsulfanylstyryl)benzene (1s-H)

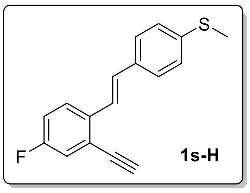

Obtained as white solid an as single diastereomer (d.r. > 20:1) (953 mg, 3.55 mmol, 78%) following method C. R_f = 0.25 (Hexane/DCM 95:5). M.p.: 63−65 ºC.

^{1}H-NMR (400 MHz, CDCl$_3$) δ (ppm) 7.64 (dd, *J* = 8.9, 5.6 Hz, 1H), 7.53 (d, *J* = 16.4 Hz, 1H), 7.46 (d, *J* = 8.4 Hz, 2H), 7.25 (d, *J* = 8.4 Hz, 2H), 7.21 (dd, *J* = 8.9, 2.8 Hz, 1H), 7.10–7.04 (m, 1H), 7.03 (d, *J* = 16.4 Hz, 1H), 3.43 (s, 1H), 2.51 (s, 3H).

^{13}C-NMR (100 MHz, CDCl$_3$) δ (ppm) 161.4 (C, d, $^{1}J_{C\text{-}F}$ = 247.8 Hz), 138.5 (C), 135.95 (C, d, $^{4}J_{C\text{-}F}$ = 3.5 Hz), 134.1 (C), 129.86 (CH, d, $^{5}J_{C\text{-}F}$ = 2.3 Hz), 127.2 (2 x CH), 126.7 (2 x CH), 126.37 (CH, d, $^{3}J_{C\text{-}F}$ = 8.5 Hz), 124.82 (CH, d, $^{6}J_{C\text{-}F}$ = 1.3 Hz), 122.48 (C, d, $^{3}J_{C\text{-}F}$ = 9.4 Hz), 119.6 (CH, d, $^{2}J_{C\text{-}F}$ = 23.1 Hz), 116.9 (CH, d, $^{2}J_{C\text{-}F}$ = 21.8 Hz), 83.2 (CH), 81.1 (C, d, $^{4}J_{C\text{-}F}$ = 3.1 Hz), 15.8 (CH_3).

^{19}F NMR (376 MHz, CDCl$_3$) δ (ppm) –114.59.

HRMS (ESI-TOF) m/z: $[M+H]^+$ Calcd for $C_{17}H_{14}FS$ 269.0795. Found: 269.0790.

Step 2: *n*-BuLi (1.77 mL, 2.83 mmol, 1.2 equiv., 1.6 M in hexanes) was added dropwise at –78 ºC to a solution of **1s-H** (632 mg, 2.36 mmol, 1 equiv.) in dry THF (0.28 M) under Ar. After 30 min, MeI (0.29 mL, 4.7 mmol, 2.0 equiv.) was added dropwise and the mixture was stirred at RT until consumption of starting material. Water was added to the reaction mixture and it was extracted with Et_2O; the combined organic layers were dried over anhydrous Na_2SO_4, filtered and evaporated under reduced pressure. The residue was purified by flash chromatography on silica gel using mixtures of hexane and DCM as eluent to obtain methylated *o*-(alkynyl)styrene **1s** as a white solid an as almost single diastereomer (d.r. > 20:1) (563 mg, 2.0 mmol, 85%).

(*E*)-2-Propynyl-4-fluoro-1-(4-methylsulfanylstyryl)benzene (1s)

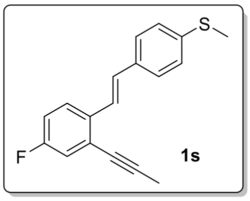

R_f = 0.13 (Hexane/DCM 95:5). M.p.: 97−99 ºC.

^{1}H-NMR (400 MHz, CDCl$_3$) δ (ppm) 7.60 (dd, *J* = 8.8, 5.8 Hz, 1H), 7.51 (d, *J* = 16.4 Hz, 1H), 7.45 (d, *J* = 8.4 Hz, 2H), 7.26 (d, *J* = 8.4 Hz, 2H), 7.11 (dd, *J* = 9.3, 2.7 Hz, 1H), 7.01–6.96 (m, 1H), 7.01 (d, *J* = 16.4 Hz, 1H), 2.51 (s, 3H), 2.15 (s, 3H).

^{13}C-NMR (100 MHz, CDCl$_3$) δ (ppm) 161.6 (C, d, $^{1}J_{C\text{-}F}$ = 247.8 Hz), 138.2 (C), 135.12 (d, $^{4}J_{C\text{-}F}$ = 3.3 Hz), 134.47 (C, d, $^{7}J_{C\text{-}F}$ = 0.6 Hz), 129.13 (CH, d, $^{5}J_{C\text{-}F}$ = 2.3 Hz), 127.2 (2 x CH), 126.8 (2 x CH), 126.27 (CH, d, $^{3}J_{C\text{-}F}$ = 8.6 Hz), 125.54 (CH, d, $^{6}J_{C\text{-}F}$ = 1.2 Hz), 122.64 (C, d, $^{3}J_{C\text{-}F}$ = 9.5 Hz), 119.07 (d, $^{2}J_{C\text{-}F}$ = 22.6 Hz), 115.58 (d, $^{2}J_{C\text{-}F}$ = 21.8 Hz), 92.3 (C), 77.4 (C), 15.9 (CH_3), 4.8 (CH_3).

^{19}F-NMR (376 MHz, CDCl$_3$) δ (ppm) –115.00.

HRMS (ESI-TOF) m/z: $[M+H]^+$ Calcd for $C_{18}H_{16}FS$ 283.0951 Found 283.0956.

Borylative cyclization of substrate 1s: The reaction conditions were optimized from the general procedure to reach full conversion. To a stirred solution of *o*-alkynylstyrene **1s** (0.4 mmol, 113 mg) and 2,4,6-tri-*tert*-butylpyridine (0.4 mmol, 99 mg) in anhydrous 1,2-dichloroethane (0.06 M) under Ar, BCl_3 (1.0 M in heptane, 1.6 mL, 1.6 mmol, 4 equiv.) was added dropwise. Then, the flask was sealed and heated to 70 ºC for 2 h. The remaining intermediate **3s-BCl₂** was treated with a solution of pinacol (61.5 mg, 0.52 mmol, 1.3 equiv.) in Et_3N (0.83 mL, 6 mmol, 15 equiv.) and the mixture was stirred for 2 minutes. The crude was extracted with hexane and the organic layer washed with HCl (1M) and brine, dried over anhydrous Na_2SO_4, filtered and concentrated under reduced pressure. The resulting residue was purified by column chromatography on silica gel using a mixture of hexane/EtOAc 98/2 as eluent to give **3s** (137 mg, 0.34 mmol, 84% yield) as *Z*/*E* (2:1) mixture of isomers. Further treatment of the mixture with HCl (2 M in Et_2O) at room temperature for 14 h increased the amount of the *Z* isomer (*Z*/*E* 95:5).

(*Z*)-5-Fluoro-2-methyl-1-(4-methylsulfanylbenzylidene)-3-(4,4,5,5-tetramethyl-1,3,2-dioxaborolan-2-yl)-1*H*-indene (3s)

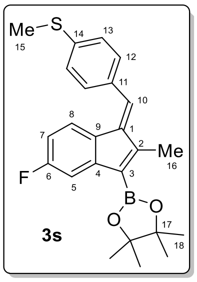

Orange oil. R_f = 0.38 (Hexane/EtOAc 95:5).

^{1}H-NMR (400 MHz, $CDCl_3$) δ (ppm) 7.47 (d, *J* = 8.3 Hz, 2H, H12), 7.42–7.37 (m, 2H, H5/H8), 7.29 (dt, *J* = 8.3 Hz, 2H, H13), 7.23 (s, 1H, H10), 6.54 (td, *J* = 9.0, 2.5 Hz, 1H, H7), 2.55 (s, 3H, H15), 2.47 (s, 3H, H16), 1.37 (s, 12H, H18).

^{13}C-NMR (100 MHz, $CDCl_3$) δ (ppm). 163.2 (d, $^1J_{C-F}$ = 244.5 Hz, C, C6), 155.3 (C, C2), 149.7 (d, $^3J_{C-F}$ = 9.4 Hz, C, C4), 142.0 (C, C1), 139.4 (C, C14), 132.8 (C, C11), 131.5 (d, $^6J_{C-F}$ = 2.1 Hz, CH, C10), 130.1 (d, $^4J_{C-F}$ = 2.8 Hz, C, C9), 129.9 (2 x CH, C12), 125.9 (2 x CH, C13), 123.2 (d, $^3J_{C-F}$ = 9.1 Hz, CH, C8), 109.9 (d, $^2J_{C-F}$ = 22.9 Hz, CH, C7), 109.3 (d, $^2J_{C-F}$ = 23.5 Hz, CH, C5), 83.1 (2 x C, C17), 25.0 (4 x CH_3, C18), 15.4 (CH_3, C15), 13.1 (CH_3, C16). Csp^2–B (C3) signal is not observed.

^{11}B-NMR (128 MHz, $CDCl_3$) δ 30.2.

^{19}F NMR (376 MHz, $CDCl_3$) δ −114.0.

HRMS (ESI-TOF) m/z: $[M+H]^+$ Calcd for $C_{24}H_{27}BFO_2S$ 409.1803. Found 409.1807.

Cross-coupling reaction of 3s with ethyl bromoacetate: To a suspension of $Pd(OAc)_2$ (1.12 mg, 5 μmol, 3 mol %), P(*o*-Tol)$_3$ (4.56 mg, 15 μmol, 9 mol %), and K_3PO_4 (176.7 mg, 0.83 mmol, 5 equiv.) in dry and degassed THF (0.25 M), prepared in a sealable Schlenk flask under Ar, was added the boronate ester **3s** (1.6:1 *Z*/*E* mixture, (68.0 mg, 1.67 mmol, 1 equiv), ethyl bromoacetate (36.8 μL, 0.33 mmol, 2 equiv.) and H_2O (5 μL, 0.33 mmol, 2 equiv.). The resulting reaction mixture was then stirred at 40 ºC for 14 h. The crude was diluted with DCM and extracted with water (3 x 10 mL). The organic phase was dried over anhydrous Na_2SO_4 and concentrated under reduced pressure. The crude residue obtained was purified by flash chromatography using a mixture of hexane/Et_2O (9:1) to yield coupled product **10s** (32.7 mg, 88.75 μmol, 53% yield) as *Z*/*E* (3:1) mixture of isomers.

3-(2-Ethoxy-2-oxoethyl)-5-Fluoro-2-methyl-1-(4-methylsulfanylbenzylidene)-1*H*-indene (10s)

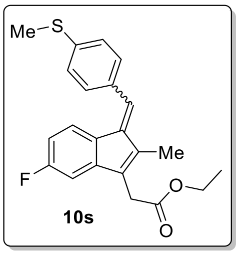

Orange oil. R_f = 0.22 (Hexane/Et_2O 9:1).

^{1}H-NMR (400 MHz, $CDCl_3$) δ (ppm) 7.53 (s, 1H, min), 7.50–7.47 (m, 1H min), 7.45 (d, *J* = 8.2 Hz, 2H maj), 7.39–7.23 (m, 3H maj + 4H min), 7.14 (s, 1H maj), 6.95 (dd, *J* = 9.0, 2.4 Hz, 1H min), 6.90 (dd, *J* = 9.1, 2.5 Hz, 1H maj), 6.84 (td, *J* = 9.0, 2.4 Hz 1H, min), 6.58 (td, *J* = 9.1, 2.5 Hz, 1H maj), 4.20–4.13 (m, 2H maj + 2H min), 3.55 (s, 2H maj), 3.54 (s, 2H min), 2.55 (s, 3H maj), 2.53 (s, 3H min), 2.21 (s, 3H maj), 1.91 (s, 3H min), 1.29–1.23 (m, 3H maj + 3H min).

^{13}C-NMR (100 MHz, $CDCl_3$) δ (ppm). 170.5 (C, maj), 170.4 (C, min), 163.33 (d, $^1J_{C\text{-}F}$ = 244.1 Hz, C min), 163.24 (d, $^1J_{C\text{-}F}$ = 245.5 Hz, C maj), 146.7 (d, $^3J_{C\text{-}F}$ = 8.8 Hz, C maj), 143.8 (d, $^3J_{C\text{-}F}$ = 8.8 Hz, C min), 140.3 (d, $^5J_{C\text{-}F}$ = 0.7 Hz, C maj), 139.6 (d, $^5J_{C\text{-}F}$ = 0.7 Hz, C min), 139.20 (C, maj), 139.0 (C, min), 138.5 (C, maj), 135.7 (C, min), 135.6 (d, $^4J_{C\text{-}F}$ = 2.8 Hz, CH min), 133.5 (d, $^4J_{C\text{-}F}$ = 2.5 Hz, CH min), 133.38 (C + CH maj), 131.3 (d, $^4J_{C\text{-}F}$ = 2.6 Hz, CH maj), 130.3 (2 x CH, min), 130.0 (2 x CH, maj), 130.1 (d, $^5J_{C\text{-}F}$ = 1.9 Hz, C maj), 129.0 (d, $^5J_{C\text{-}F}$ = 1.8 Hz, C min), 128.3 (CH, min), 126.1 (2 x CH, maj), 125.7 (2 x CH, min), 123.8 (d, $^3J_{C\text{-}F}$ = 8.9 Hz, CH maj), 119.5 (d, $^3J_{C\text{-}F}$ = 9.2 Hz, CH min), 111.1 (d, $^2J_{C\text{-}F}$ = 23.3 Hz, CH min), 110.6 (d, $^2J_{C\text{-}F}$ = 22.6 Hz, CH maj), 105.9 (d, $^2J_{C\text{-}F}$ = 23.8 Hz, CH maj), 105.9 (d, $^2J_{C\text{-}F}$ = 23.8 Hz, CH min), 61.2 (2 x CH_2, maj + min), 32.03 (CH_2, maj), 32.01 (CH_2, min), 15.59 (CH_3, min), 15.55 (CH_3, maj), 14.48 (CH_3, min), 14.33 (CH_3, maj), 14.32 (CH_3, min), 10.70 (CH_3, maj).

^{19}F NMR (376 MHz, $CDCl_3$) δ −113.9 (maj), −115.0 (min).

HRMS (ESI-TOF) m/z: $[M+H]^+$ Calcd for $C_{22}H_{22}FO_2S$ 369.1319. Found 369.1320.

Hydrolysis of 10s: In a round bottom flask, **10s** (32.7 mg, 88.75 μmol, 1 equiv.) was dissolved in a 1/1 mixture of AcOH/HCl (0.04 M, 2.4 mL), and heated to reflux for 14 h. The resulting mixture was extracted with DCM and water, the organic phases were combined, dried, and evaporated at low pressure to obtain crude Sulindac sulfide, which was directly employed in the next reaction.

Synthesis of Sulindac by Oxidation of 10s: In a round bottom flask, the sulfide obtained in the previous step was dissolved in 65/35 $CHCl_3$/AcOH (0.17 M, 0.5 mL). To the resulting mixture, a commercial solution of 30% H_2O_2 (18.2 μL, 2 equiv.) was added, and the reaction was heated to 35 ºC until the complete consumption of the sulfide was determined by TLC (4 h). The mixture was then extracted with DCM and water, the organic phases were combined, dried, and evaporated at low pressure. The crude residue was purified through column chromatography with mixtures of DCM/AcOEt as eluent to obtain Sulindac (69% in two steps, 24.7 mg) as the single *Z* stereoisomer. The spectroscopic data were consistent with those previously reported.[6]

[6] Li, Y.; Rizvi, S. A.; Hu, D.; Sun, D.; Gao, A.; Zhou, Y.; Li, J.; Jiang, X. *Angew. Chem., Int. Ed.* **2019**, *58*, 13499–13506.

Copies of ^{1}H, ^{13}C, ^{19}F and ^{11}B NMR spectra for novel compounds and selected GCOSY, TOCSY, NOESY, GHSQC and GHMBC spectra

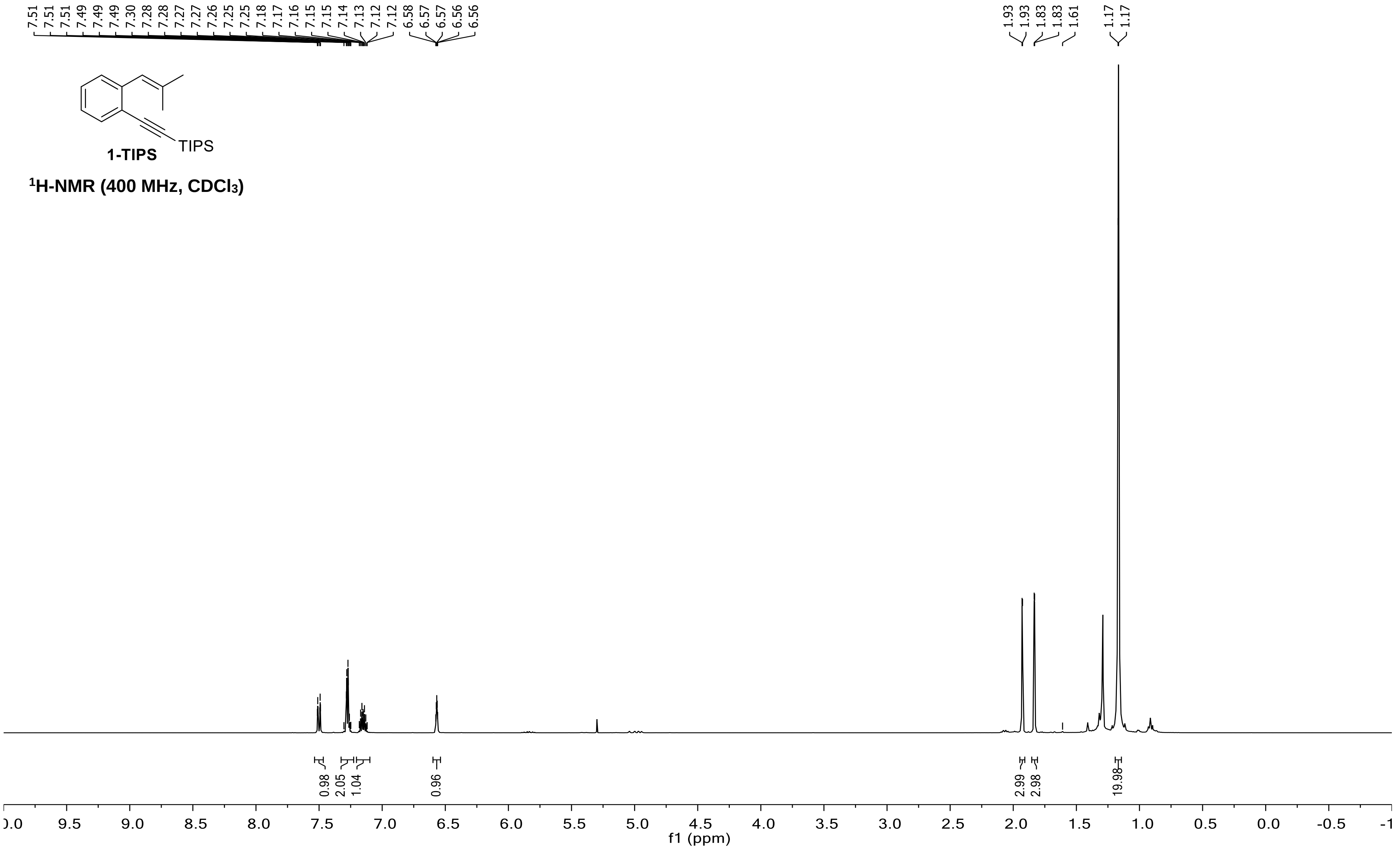
7.51
7.51
7.51
7.49
7.49
7.30
7.28
7.28
7.27
7.27
7.26
7.25
7.25
7.18
7.17
7.16
7.15
7.15
7.14
7.13
7.12
7.12
6.58
6.57
6.57
6.56
6.56
1.93
1.93
1.83
1.83
1.61
1.17
1.17
TIPS
1-TIPS
¹H-NMR (400 MHz, CDCl₃)
0.98
2.05
1.04
0.96
2.99
2.98
19.98
f1 (ppm)

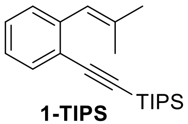

1-TIPS

^{13}C-NMR (100 MHz, CDCl$_3$)

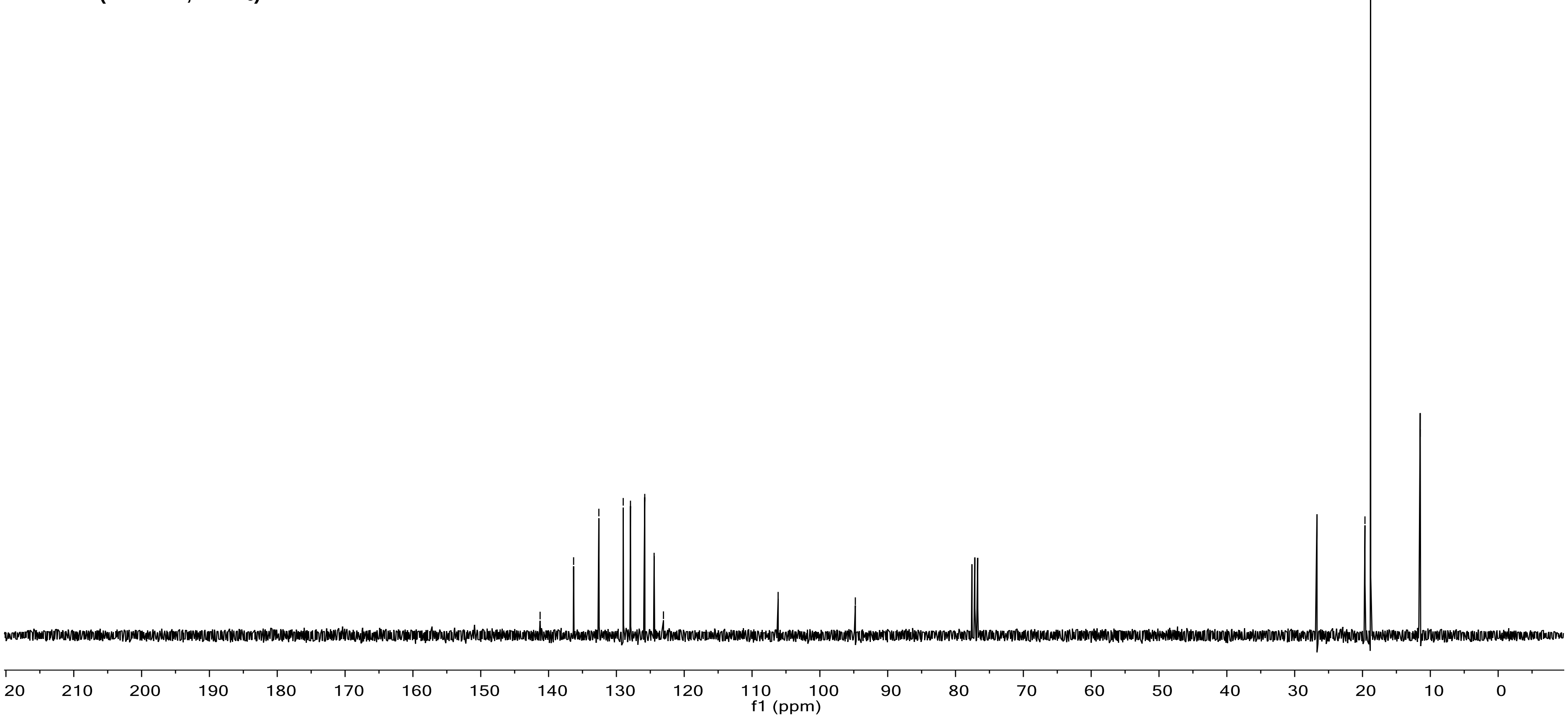

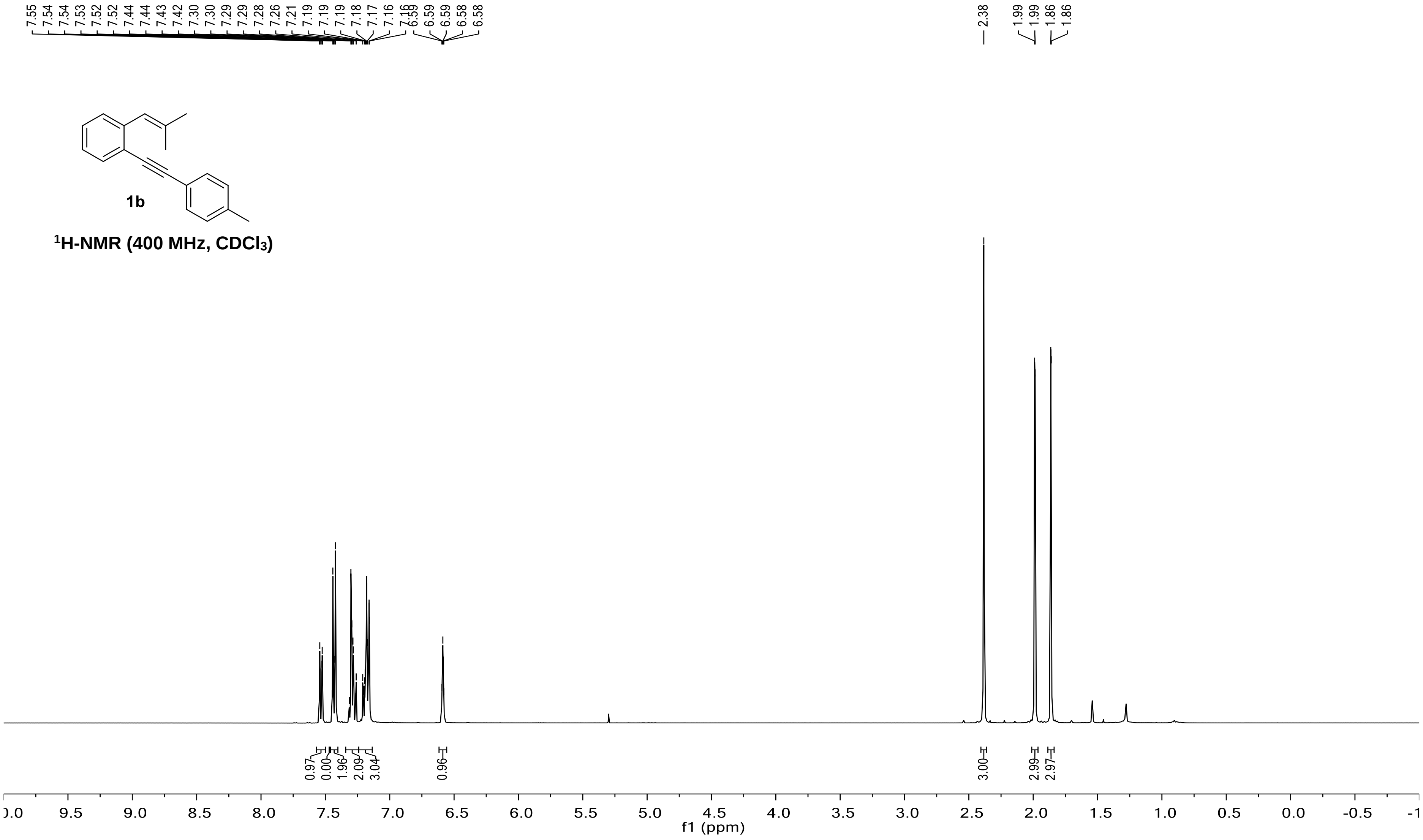

1H-NMR (400 MHz, $CDCl_3$)

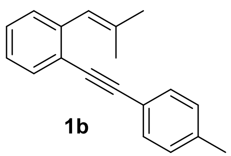

1b

^{13}C-NMR (100 MHz, $CDCl_3$)

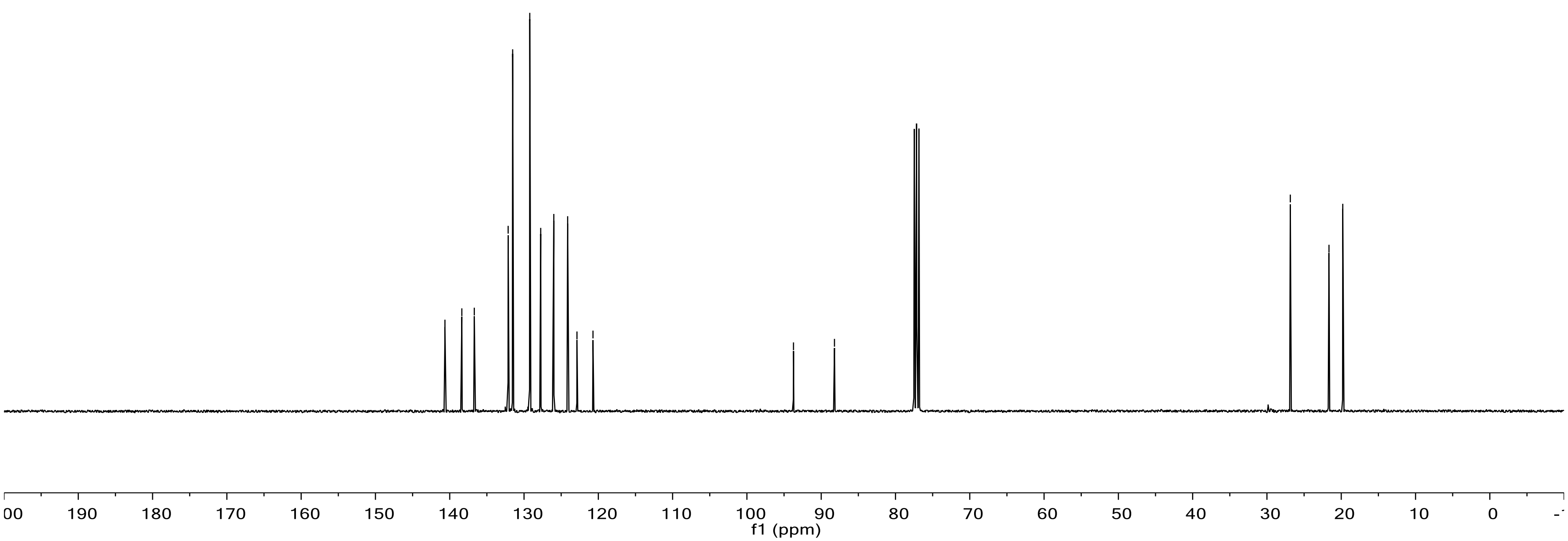

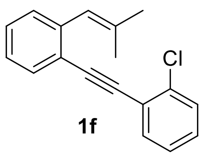

1f

^{1}H-NMR (300 MHz, CDCl$_3$)

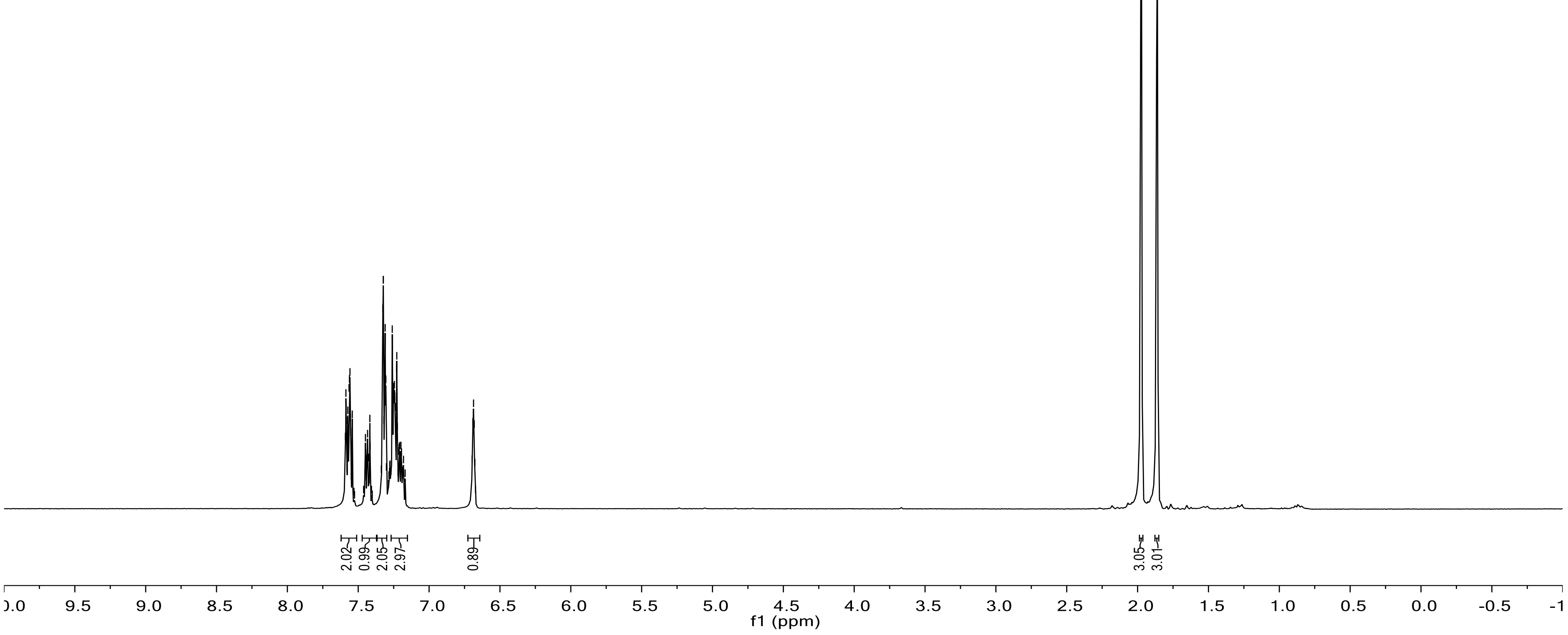

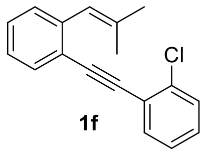

^{13}C-NMR (100 MHz, CDCl$_3$)

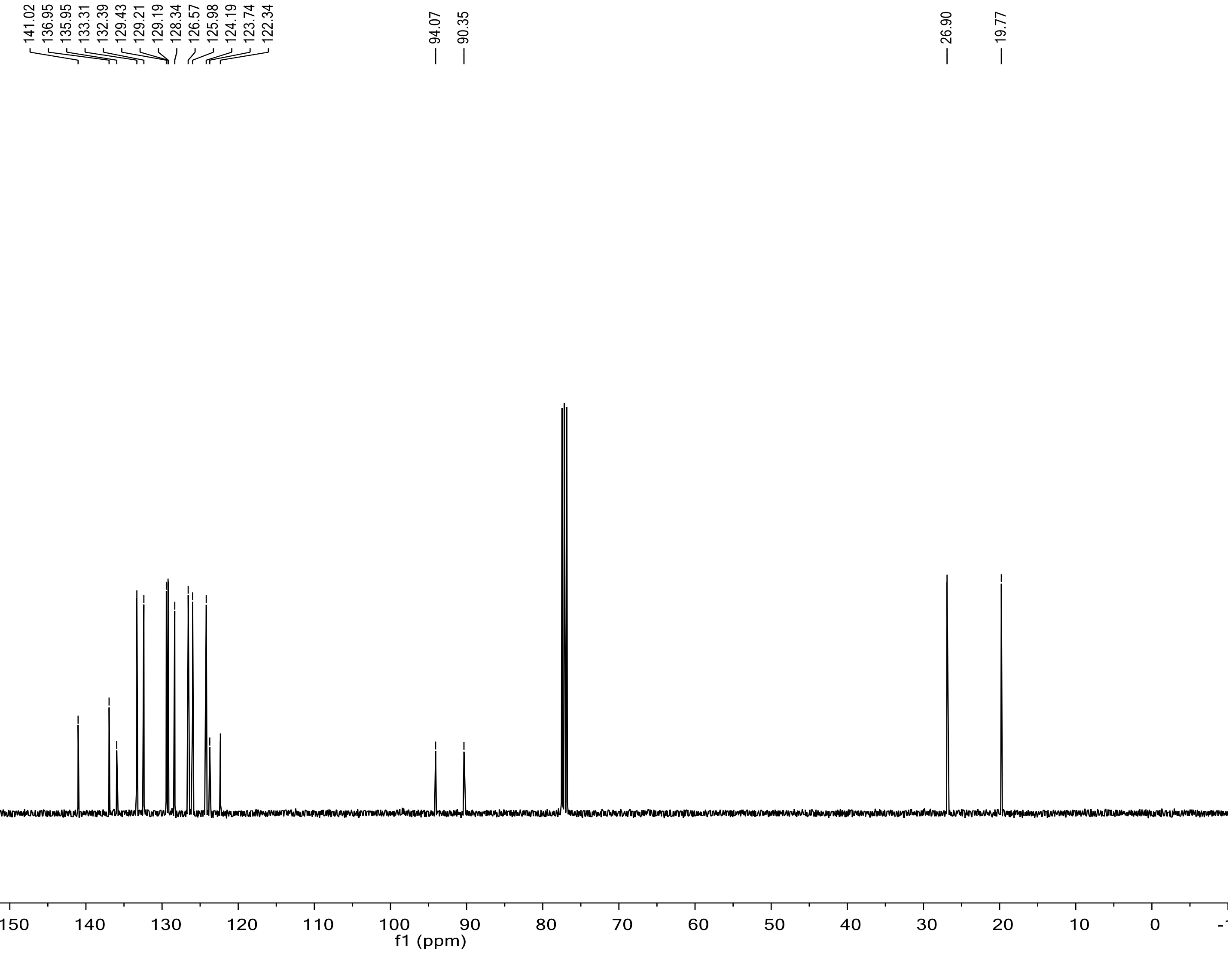

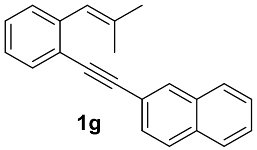

1g

^{1}H-NMR (300 MHz, CDCl$_3$)

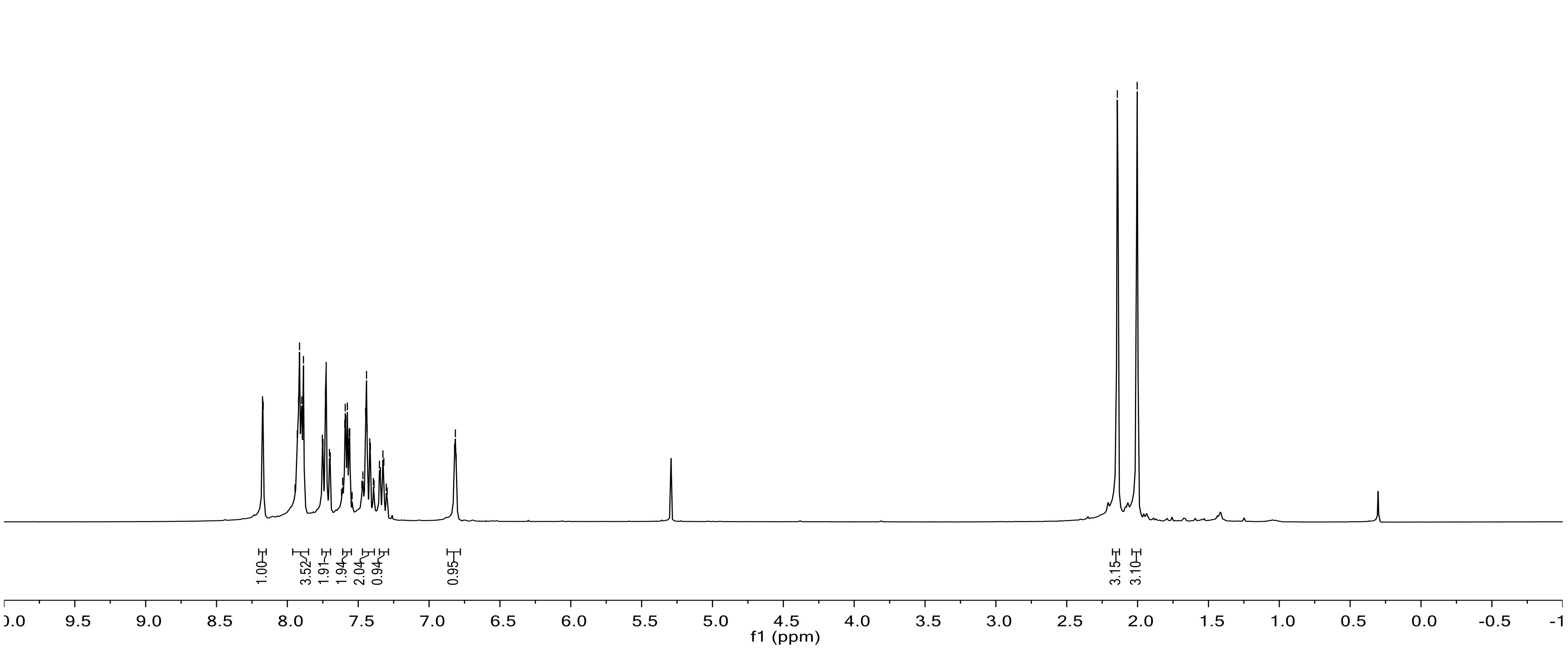

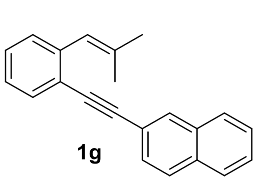

1g

^{13}C-NMR (75 MHz, $CDCl_3$)

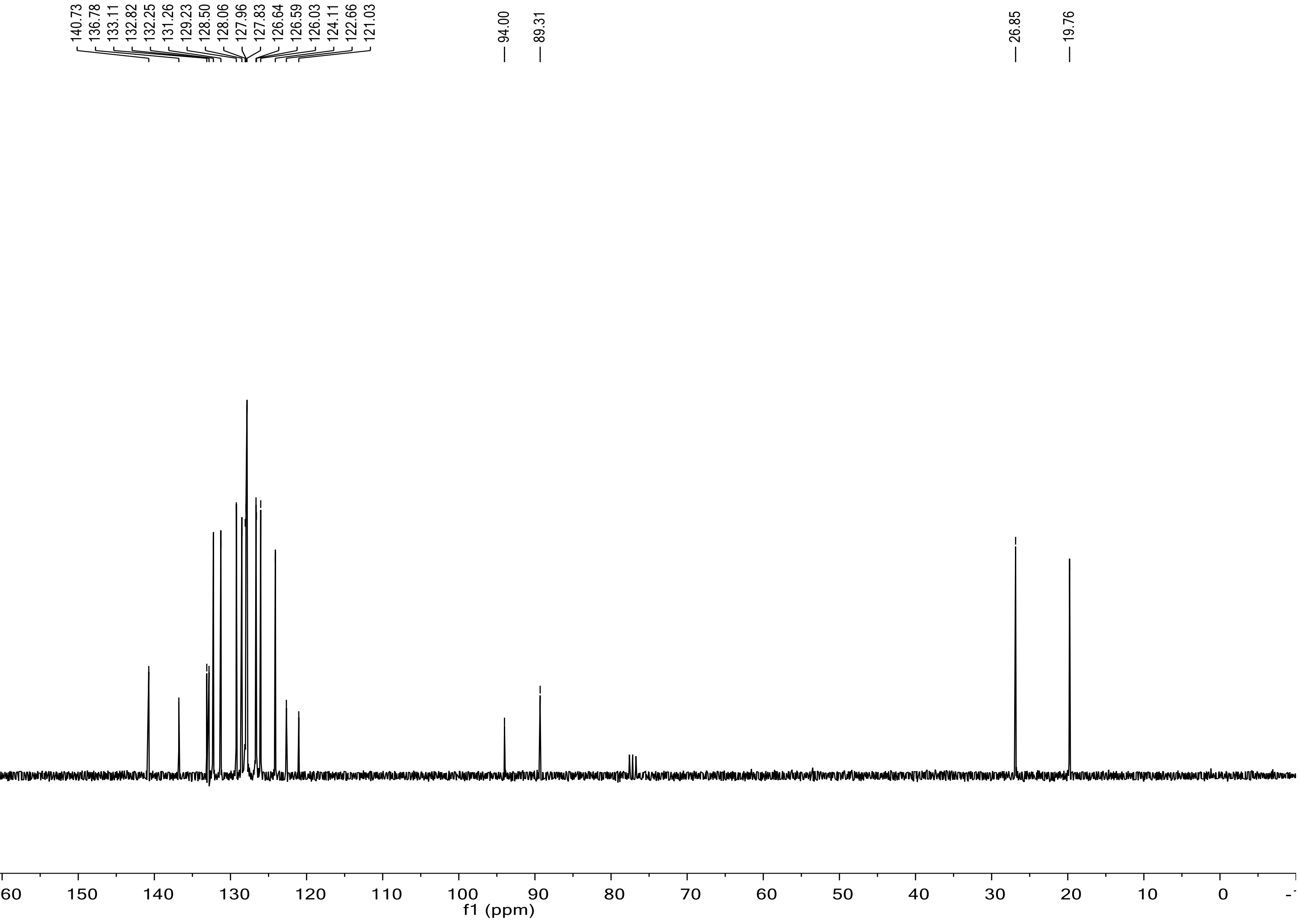

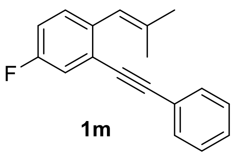

1m

^{1}H-NMR (400 MHz, CDCl$_3$)

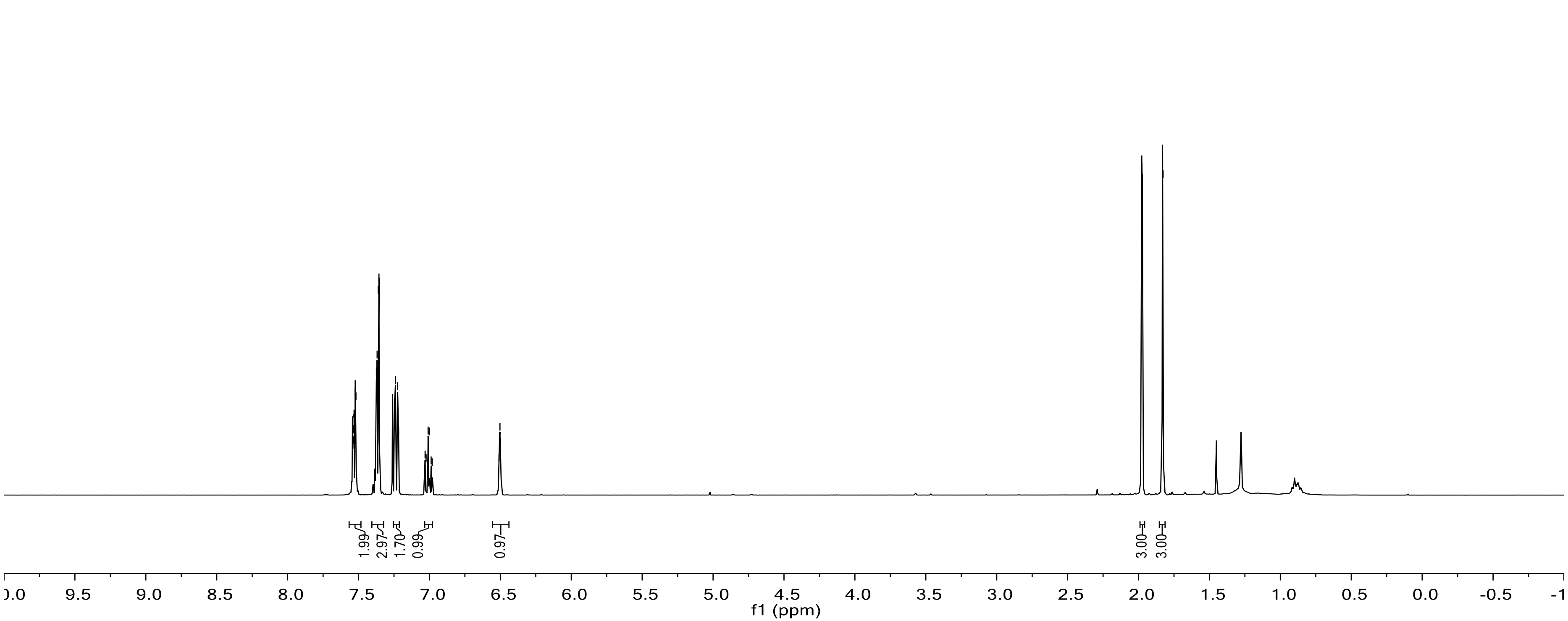

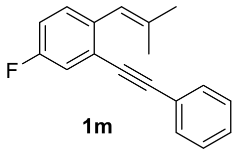

1m

^{13}C-NMR (100 MHz, CDCl$_3$)

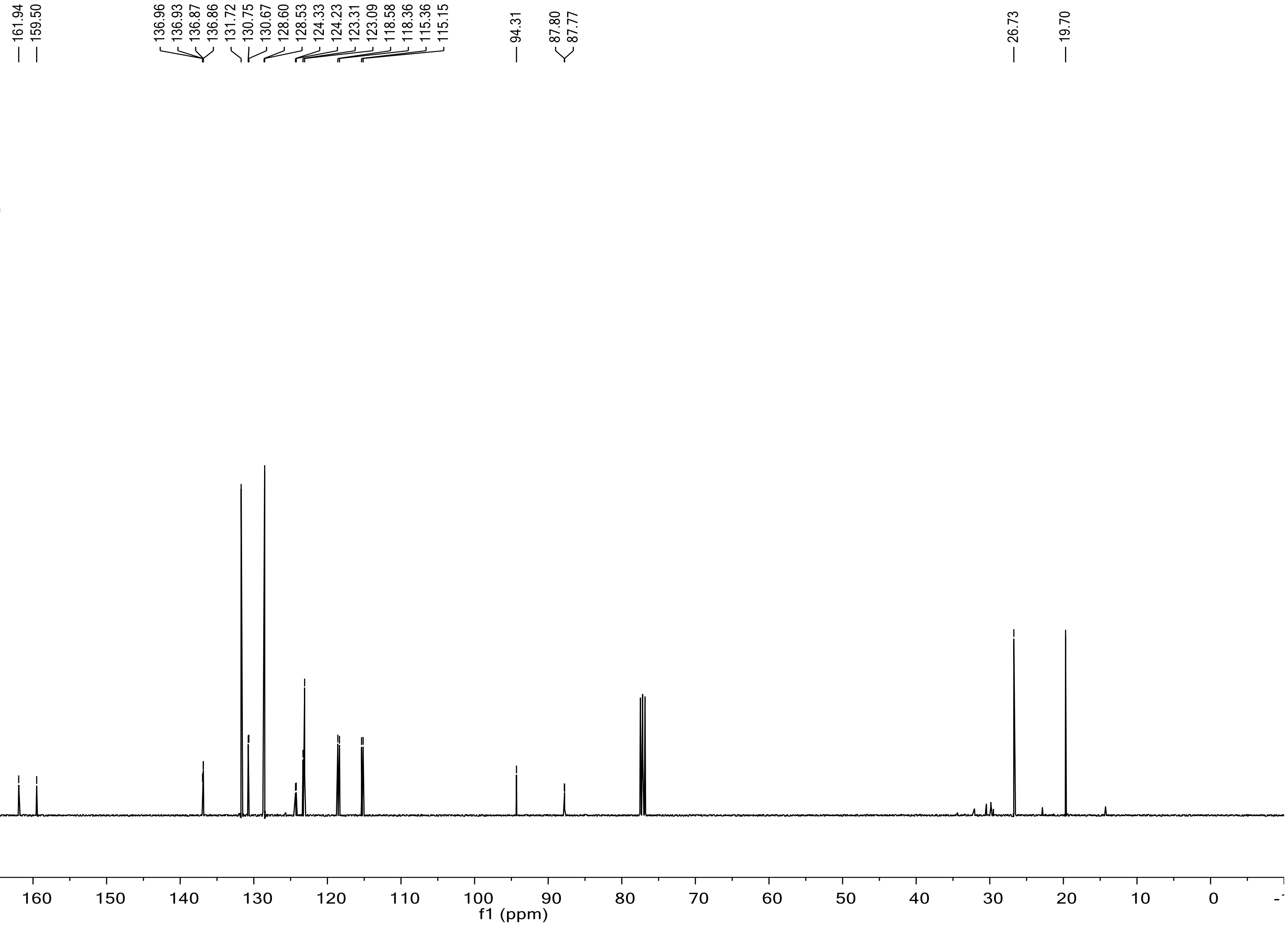

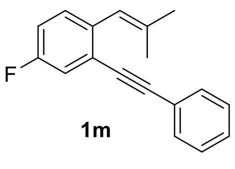

1m

^{19}F-NMR (376 MHz, CDCl$_3$)

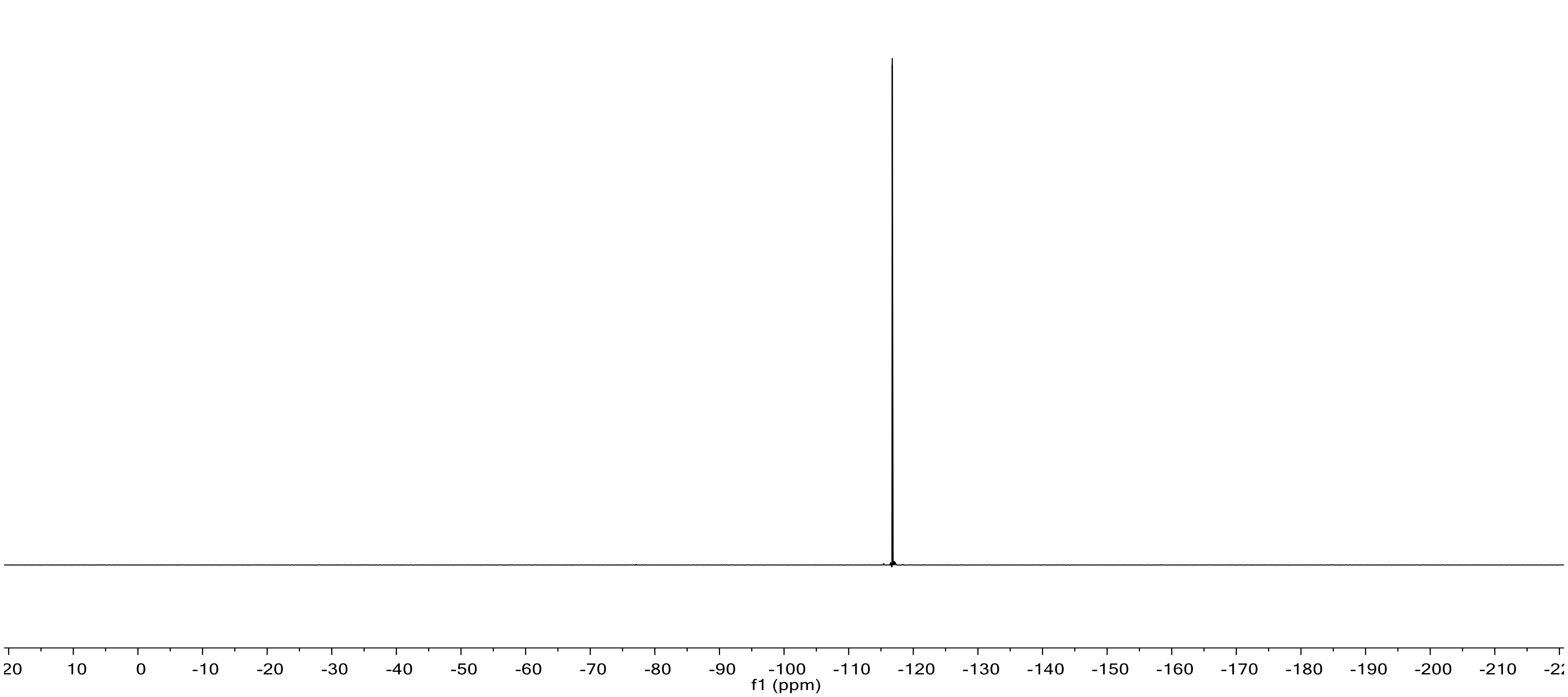

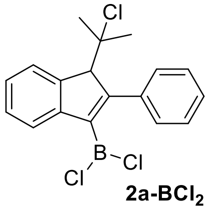

2a-BCl$_2$

^{1}H-NMR (500 MHz, CD_2Cl_2)

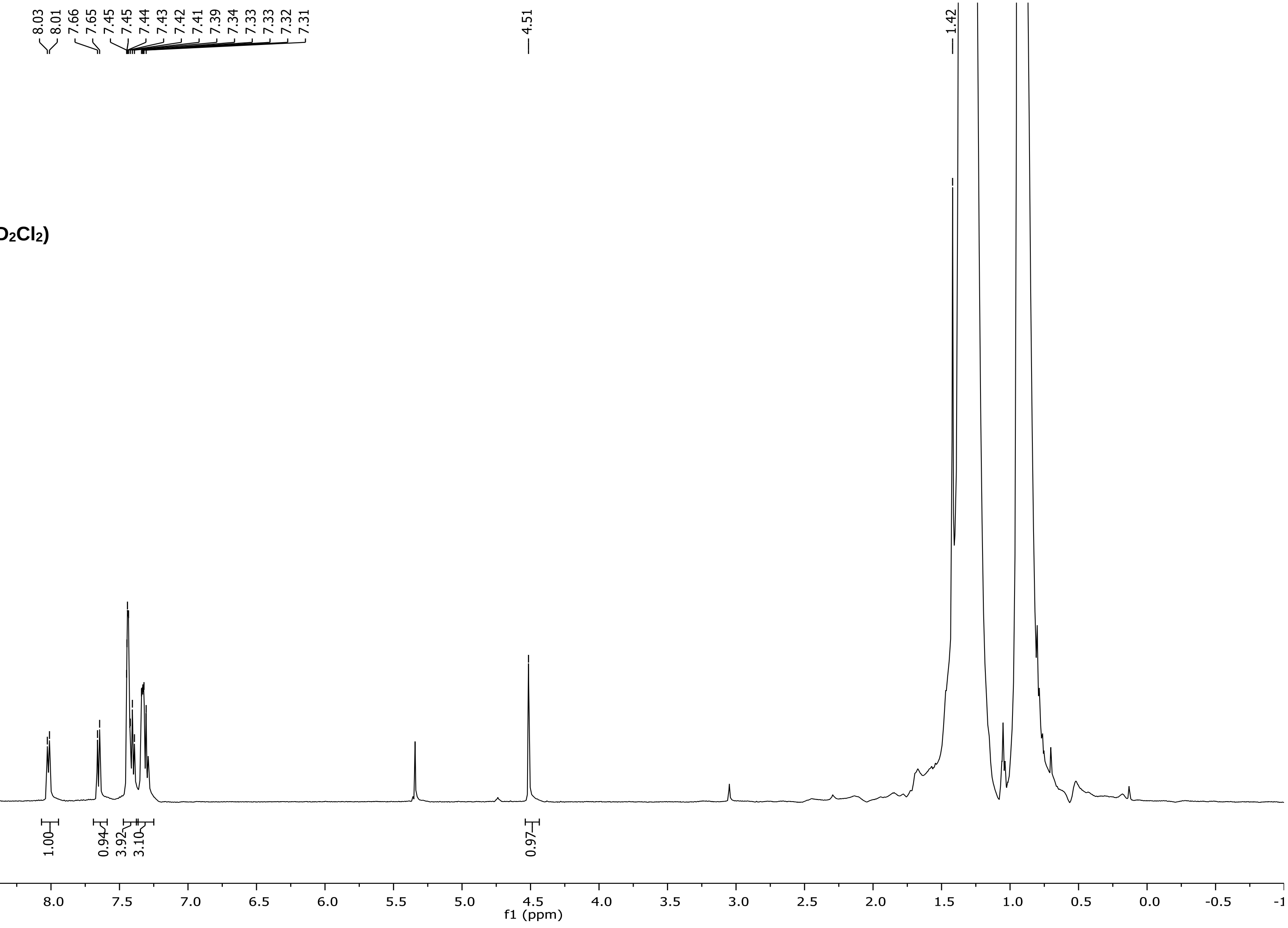

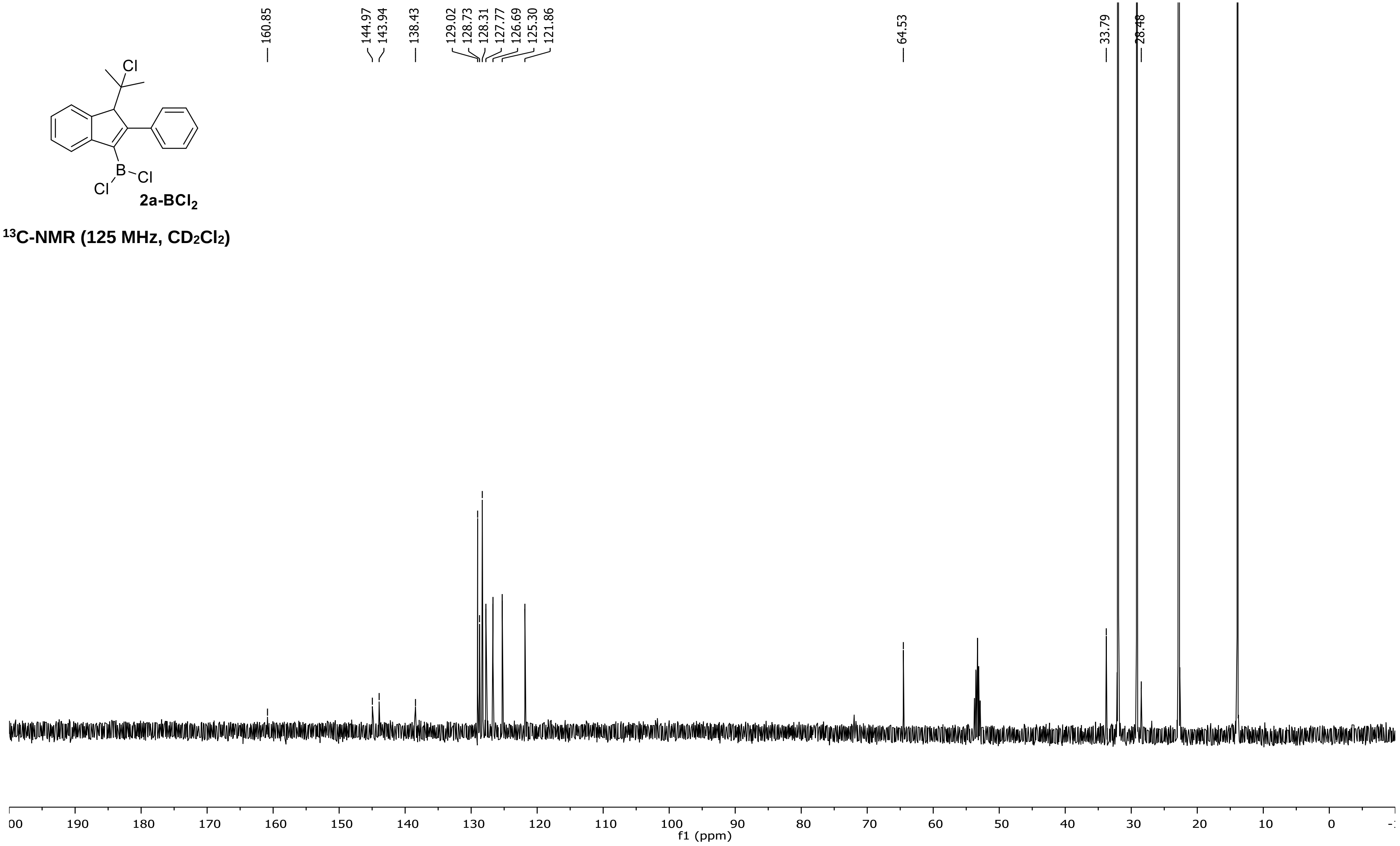

2a-BCl$_2$

^{13}C-NMR (125 MHz, CD_2Cl_2)

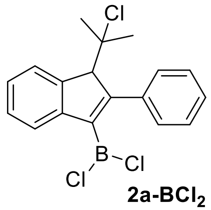

2a-BCl$_2$

^{11}B-NMR (160 MHz, CD_2Cl_2)

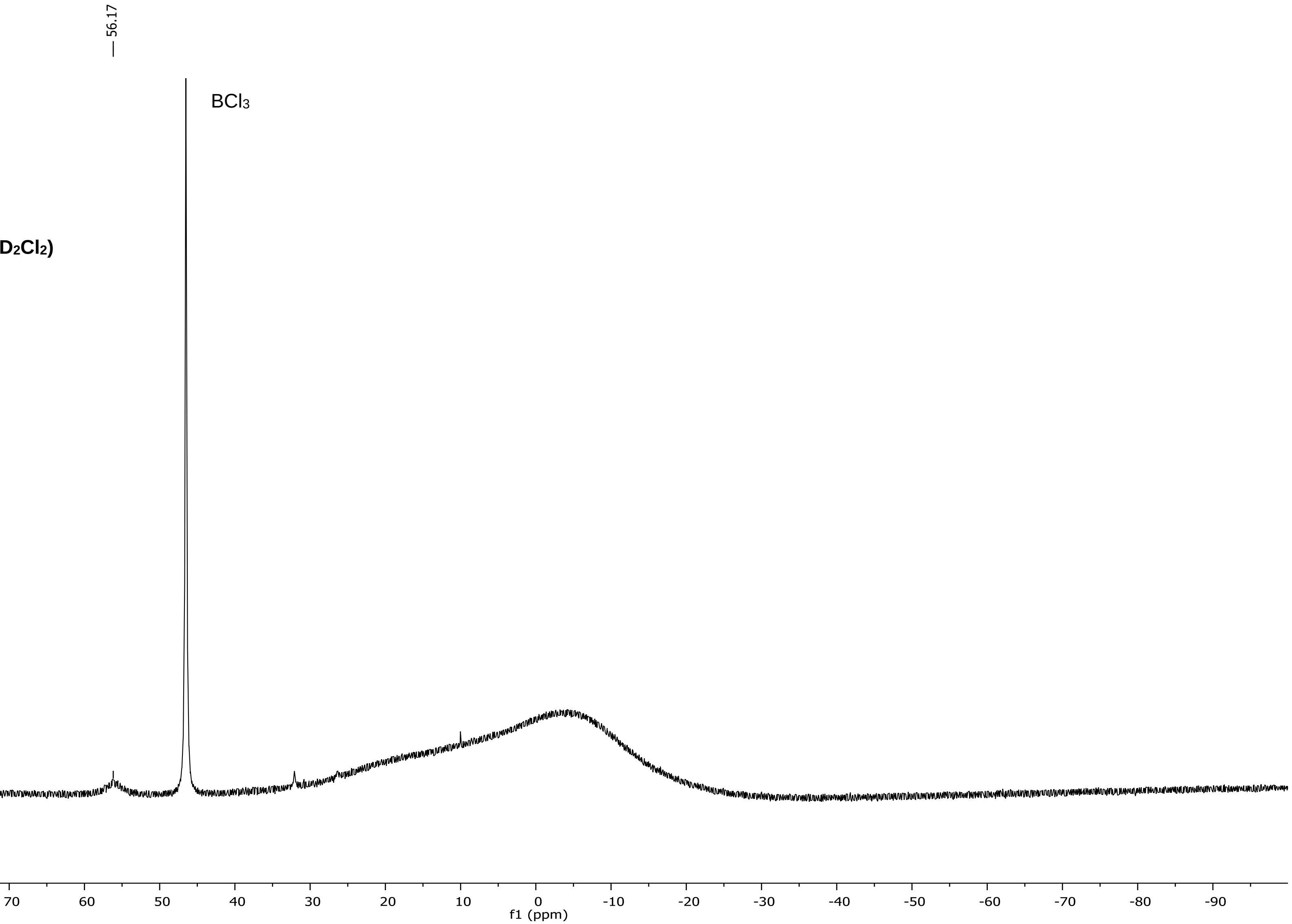

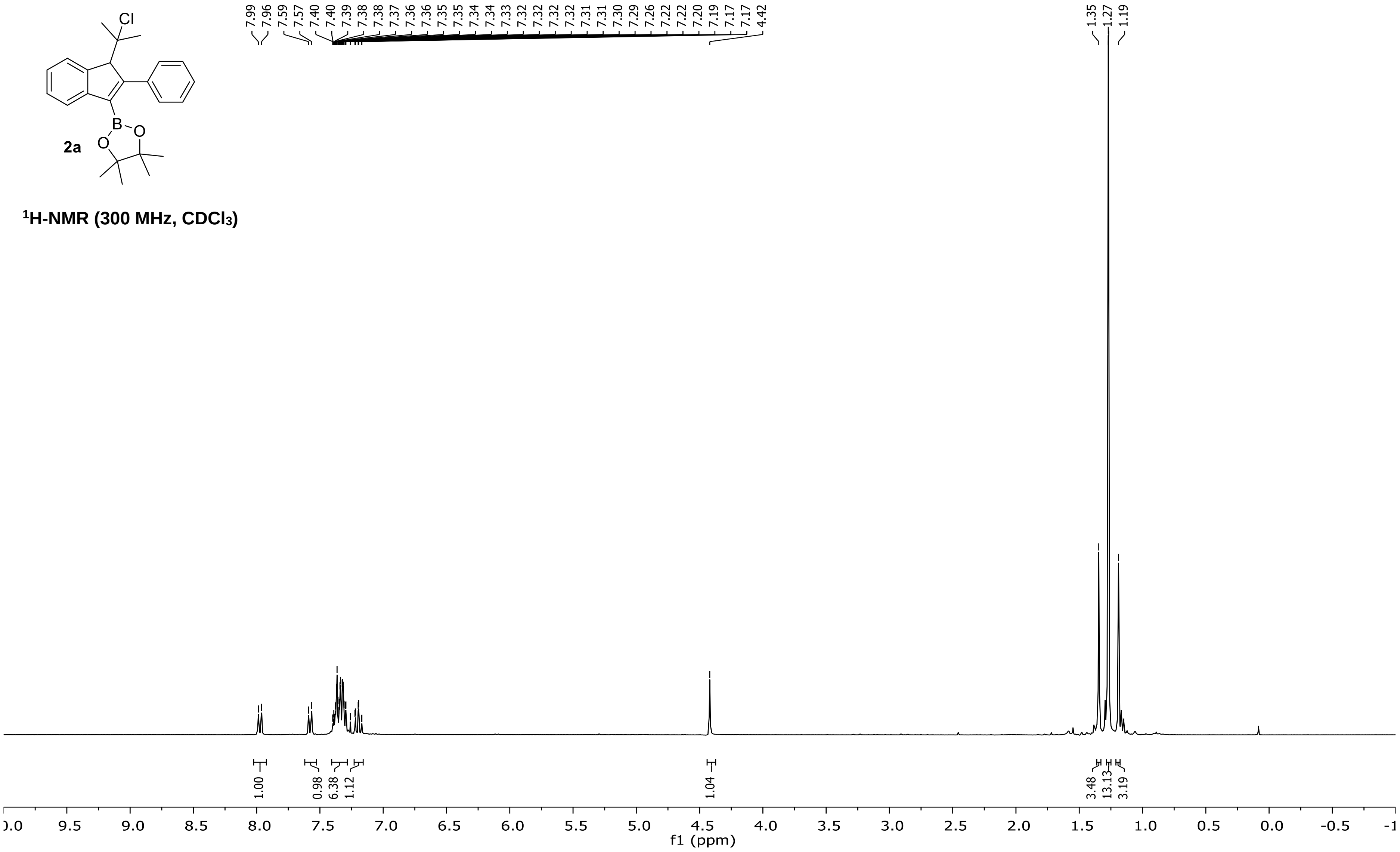
Cl
B
O
O
2a
1H-NMR (300 MHz, CDCl3)
7.99
7.96
7.59
7.57
7.40
7.40
7.39
7.38
7.38
7.37
7.36
7.36
7.35
7.35
7.34
7.34
7.33
7.32
7.32
7.32
7.32
7.31
7.31
7.30
7.29
7.26
7.22
7.22
7.20
7.19
7.17
7.17
4.42
1.35
1.27
1.19
1.00
0.98
6.38
1.12
1.04
3.48
13.13
3.19
f1 (ppm)

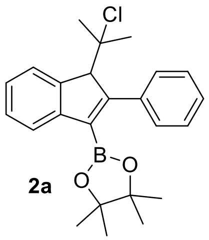

2a

^{13}C-NMR (75 MHz, CDCl$_3$)

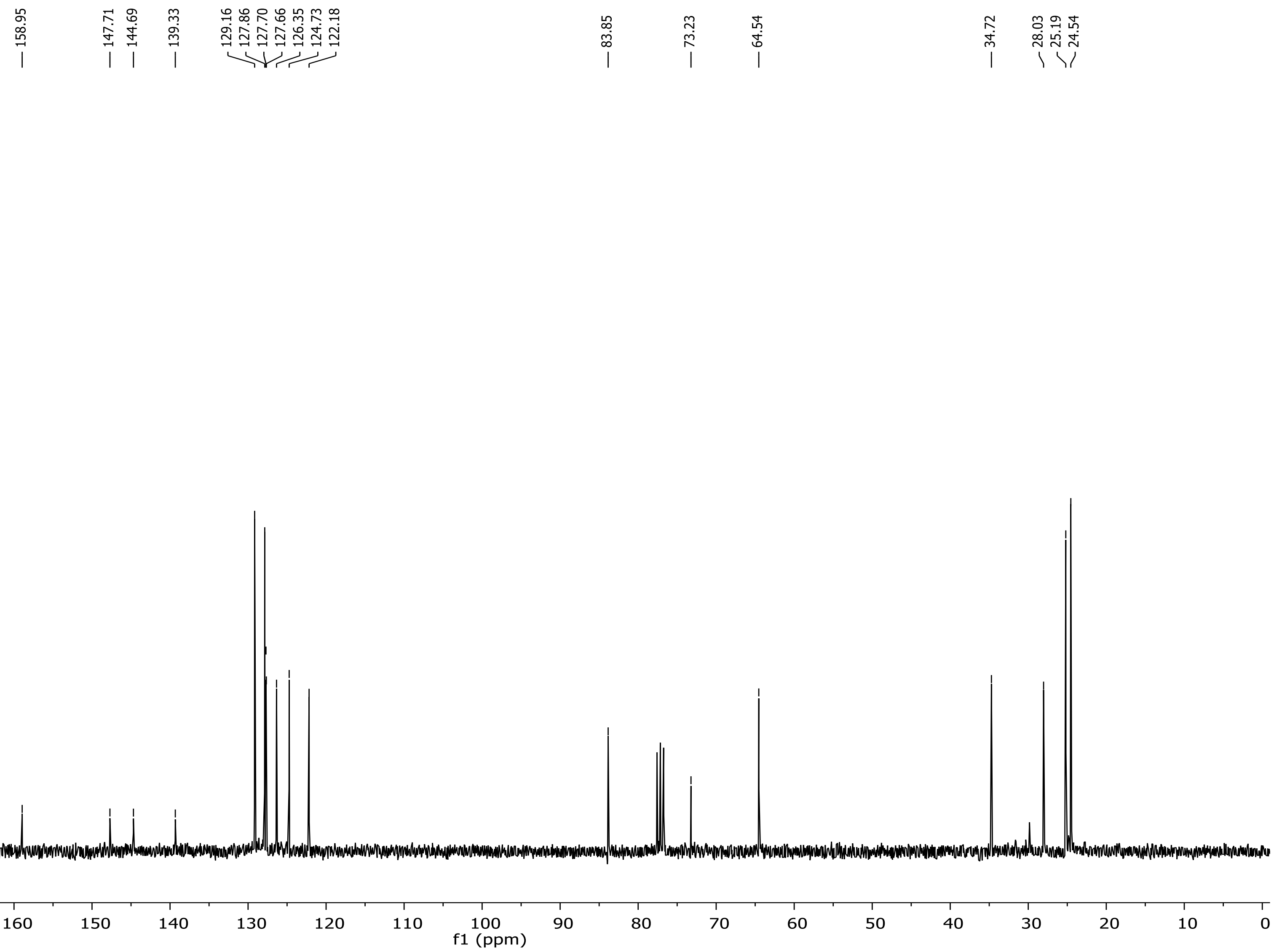

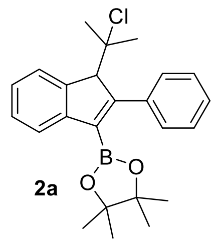

2a

^{11}B-NMR (128 MHz, $CDCl_3$)

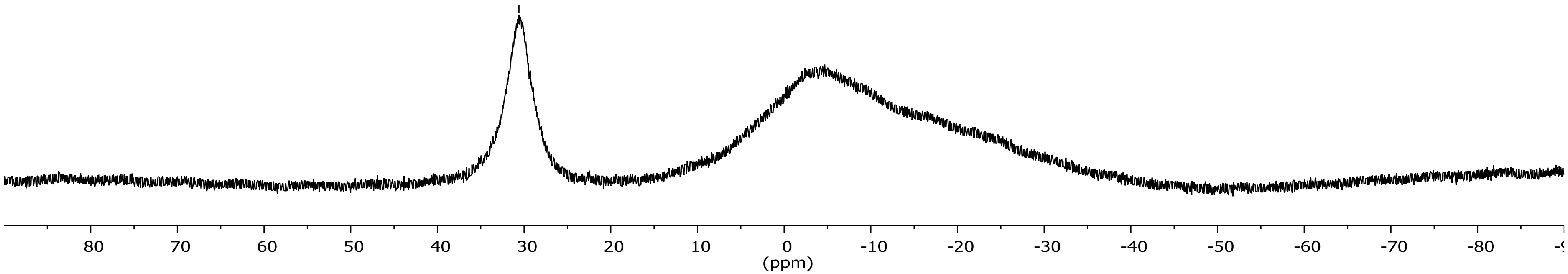

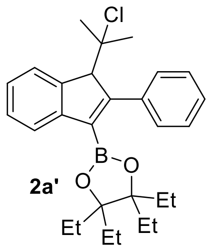

2a'

^{1}H-NMR (400 MHz, $CDCl_3$)

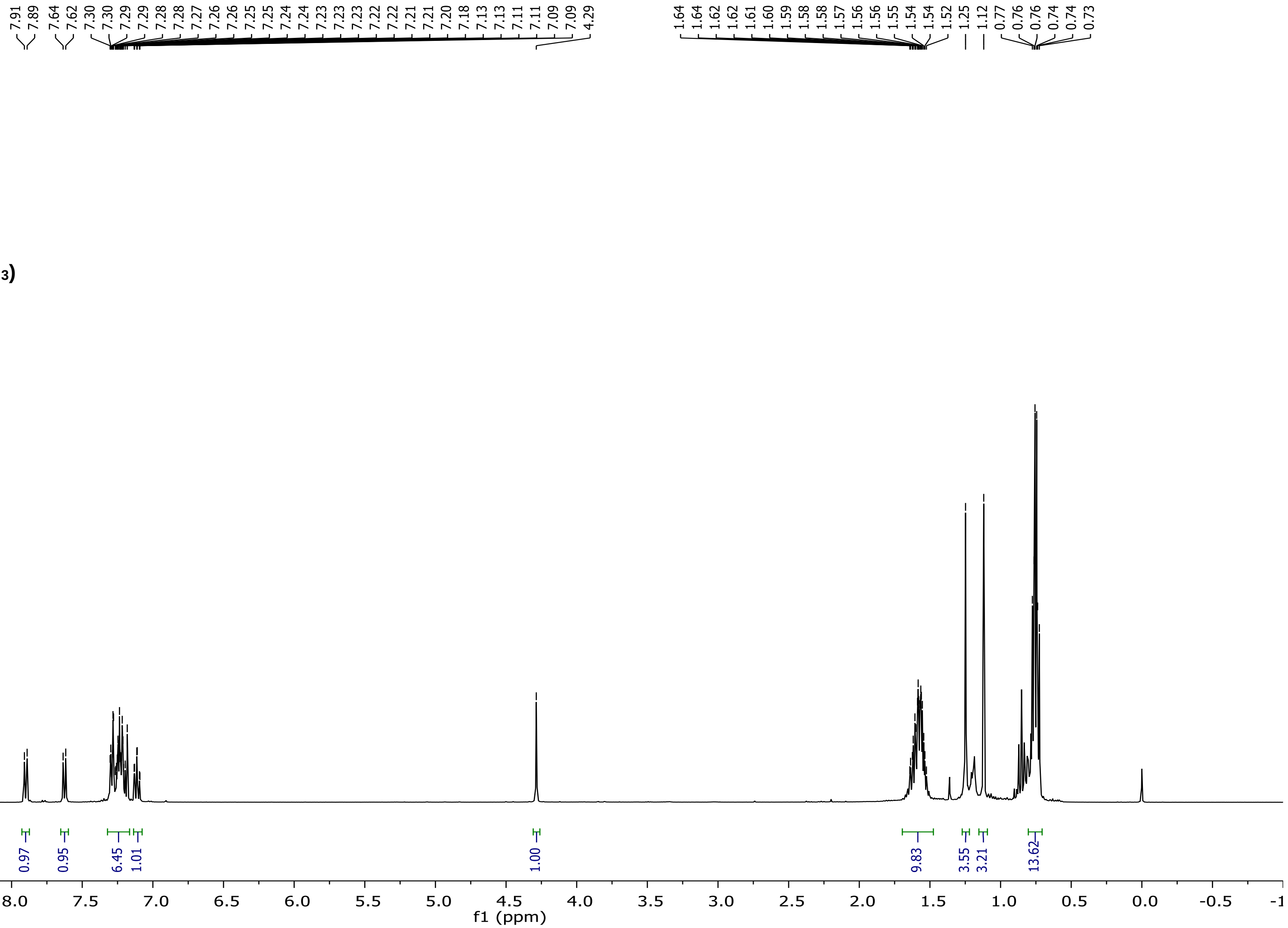

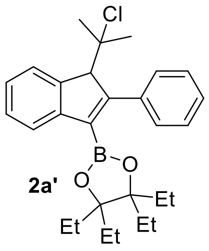

^{13}C-NMR (100 MHz, $CDCl_3$)

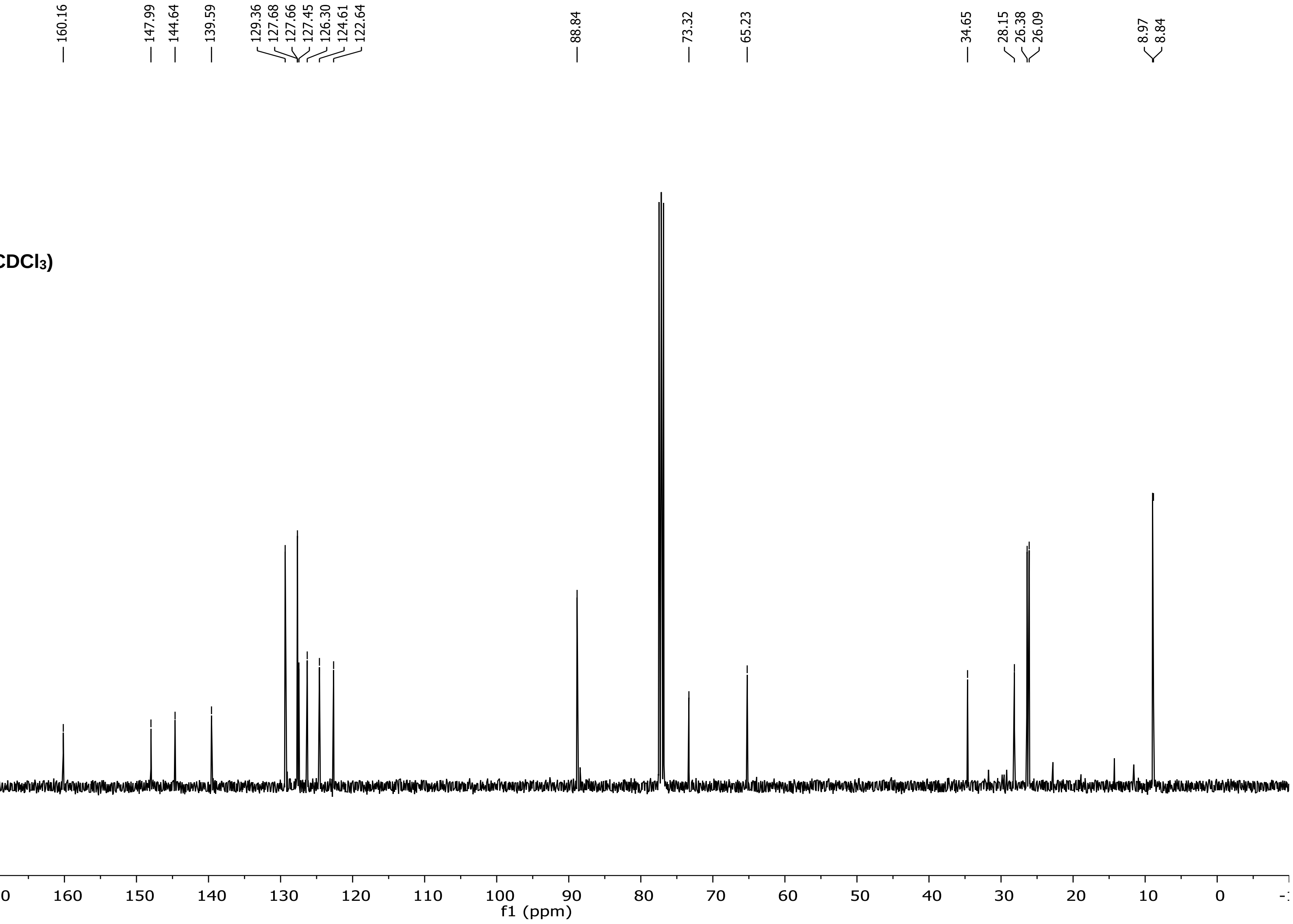

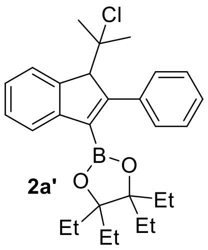

^{11}B-NMR (128 MHz, $CDCl_3$)

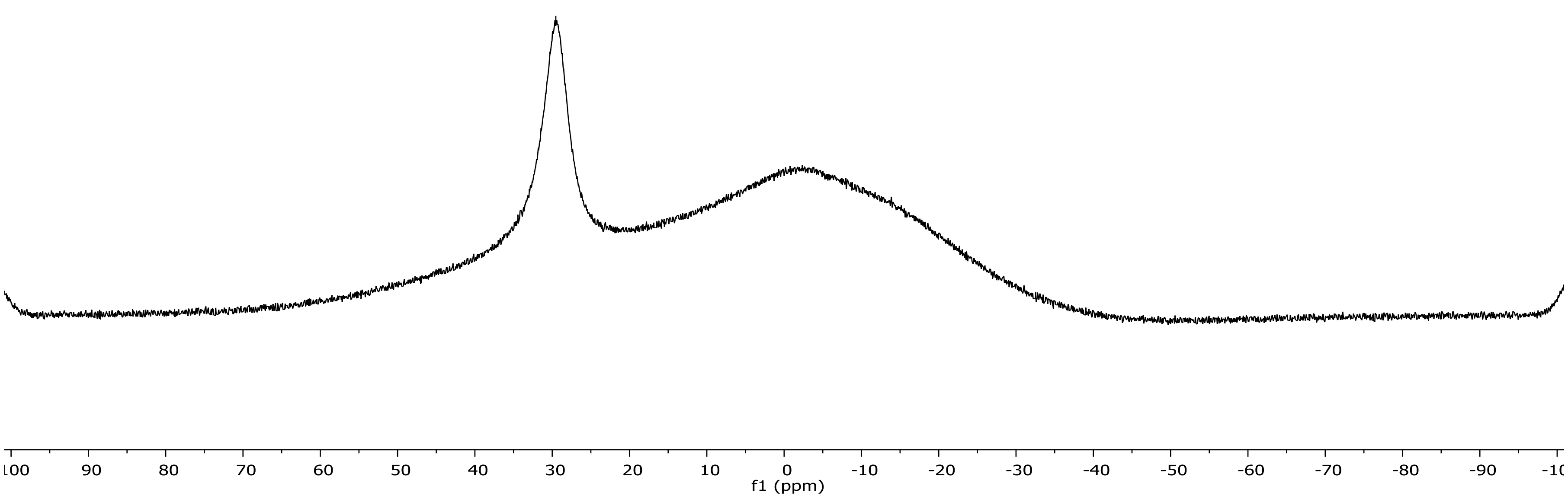

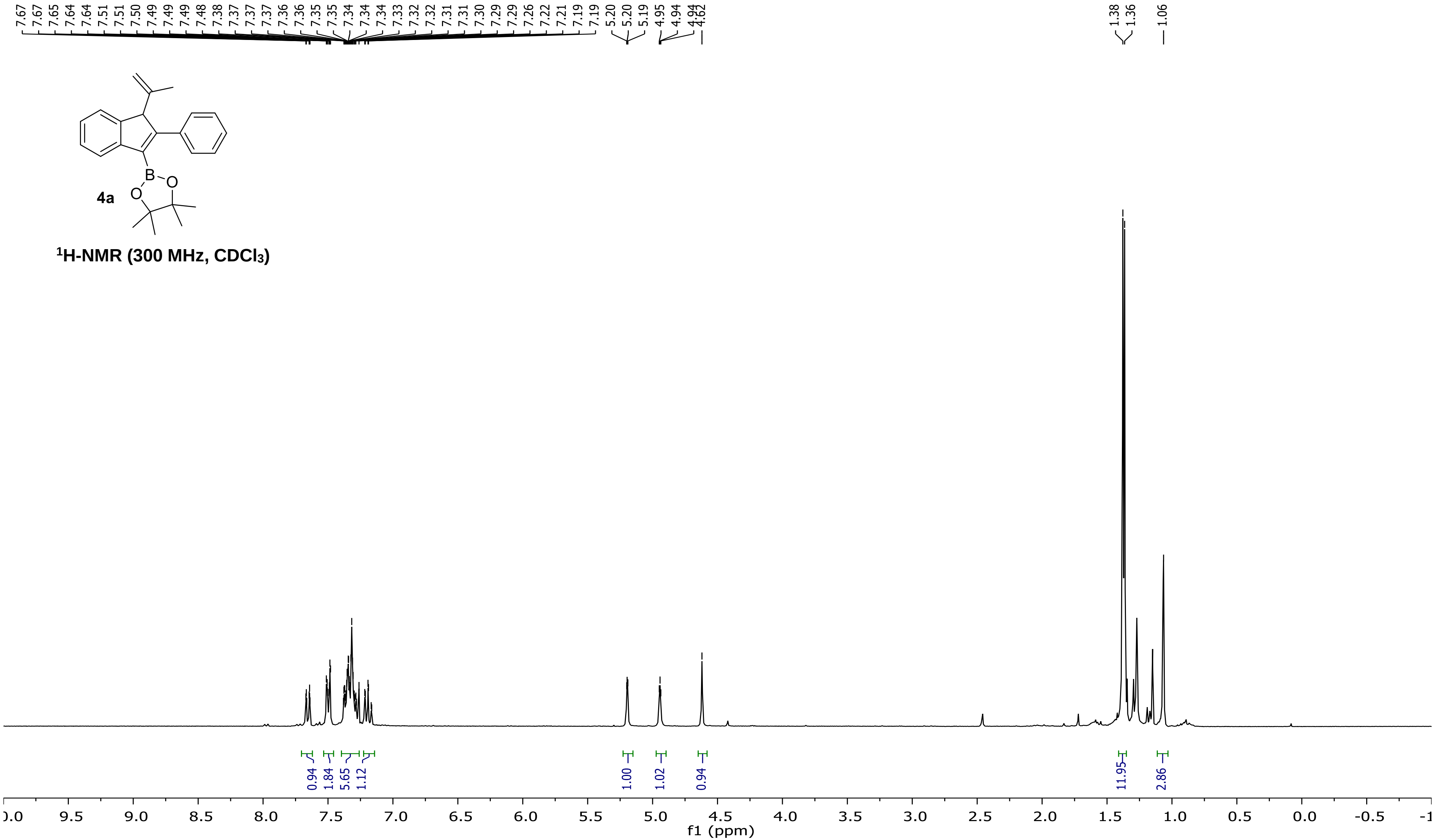

7.67
7.67
7.65
7.64
7.64
7.51
7.51
7.50
7.49
7.49
7.49
7.48
7.38
7.37
7.37
7.37
7.36
7.36
7.35
7.35
7.34
7.34
7.34
7.33
7.32
7.32
7.31
7.30
7.29
7.29
7.26
7.22
7.21
7.19
7.19
5.20
5.20
5.19
4.95
4.94
4.94
4.62
1.38
1.36
1.06
4a
B
O
O
¹H-NMR (300 MHz, CDCl₃)
0.94
1.84
5.65
1.12
1.00
1.02
0.94
11.95
2.86
9.5
9.0
8.5
8.0
7.5
7.0
6.5
6.0
5.5
5.0
4.5
4.0
3.5
3.0
2.5
2.0
1.5
1.0
0.5
0.0
-0.5
f1 (ppm)

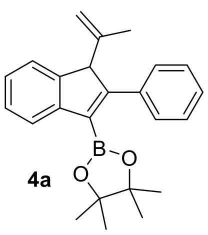

4a

^{13}C-NMR (75 MHz, CDCl3)

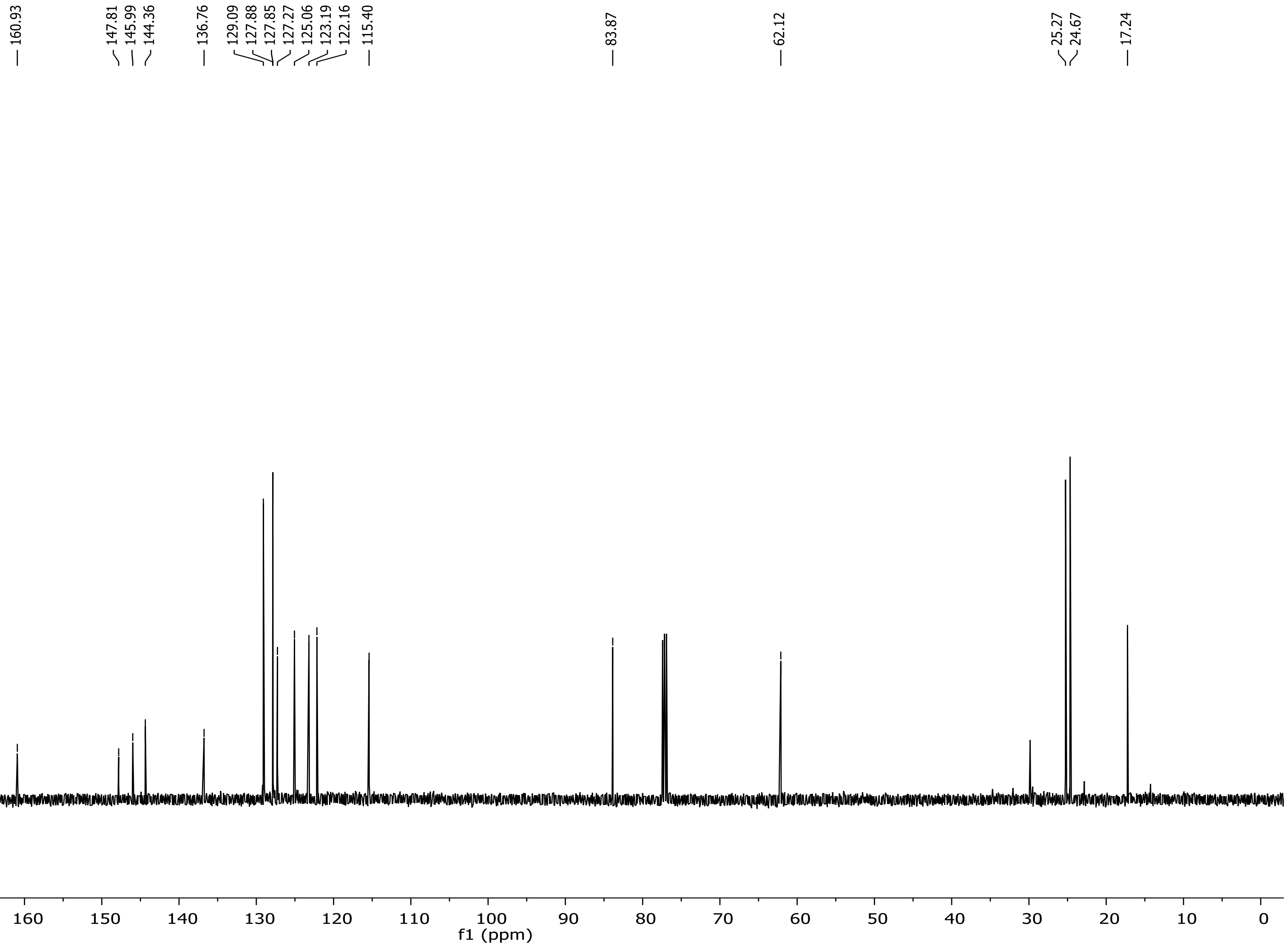

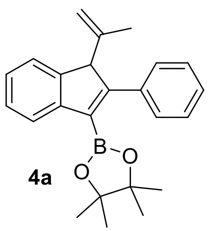

4a

^{11}B-NMR (128 MHz, $CDCl_3$)

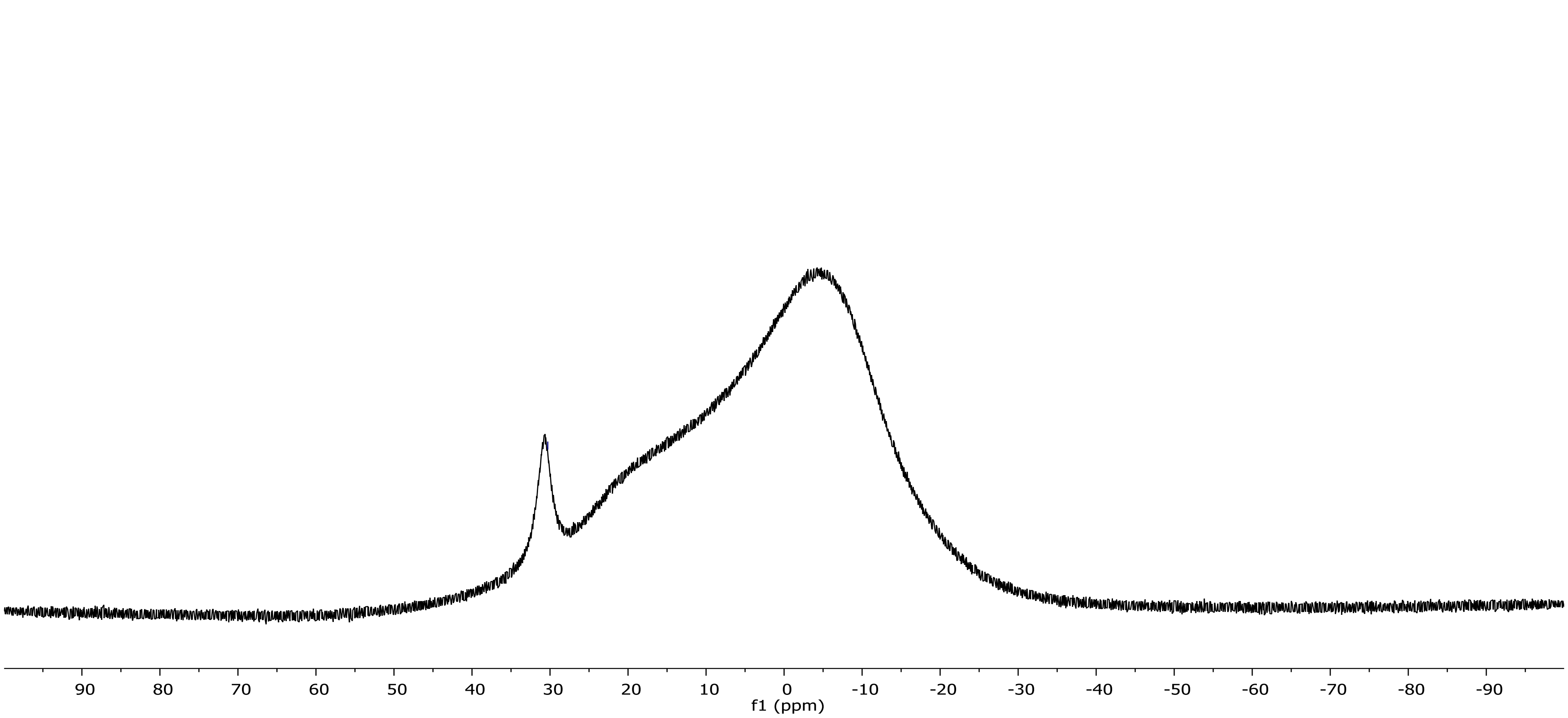

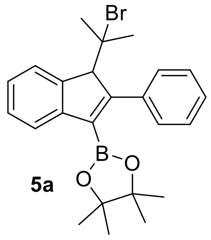

5a

^{1}H-NMR (400 MHz, $CDCl_3$)

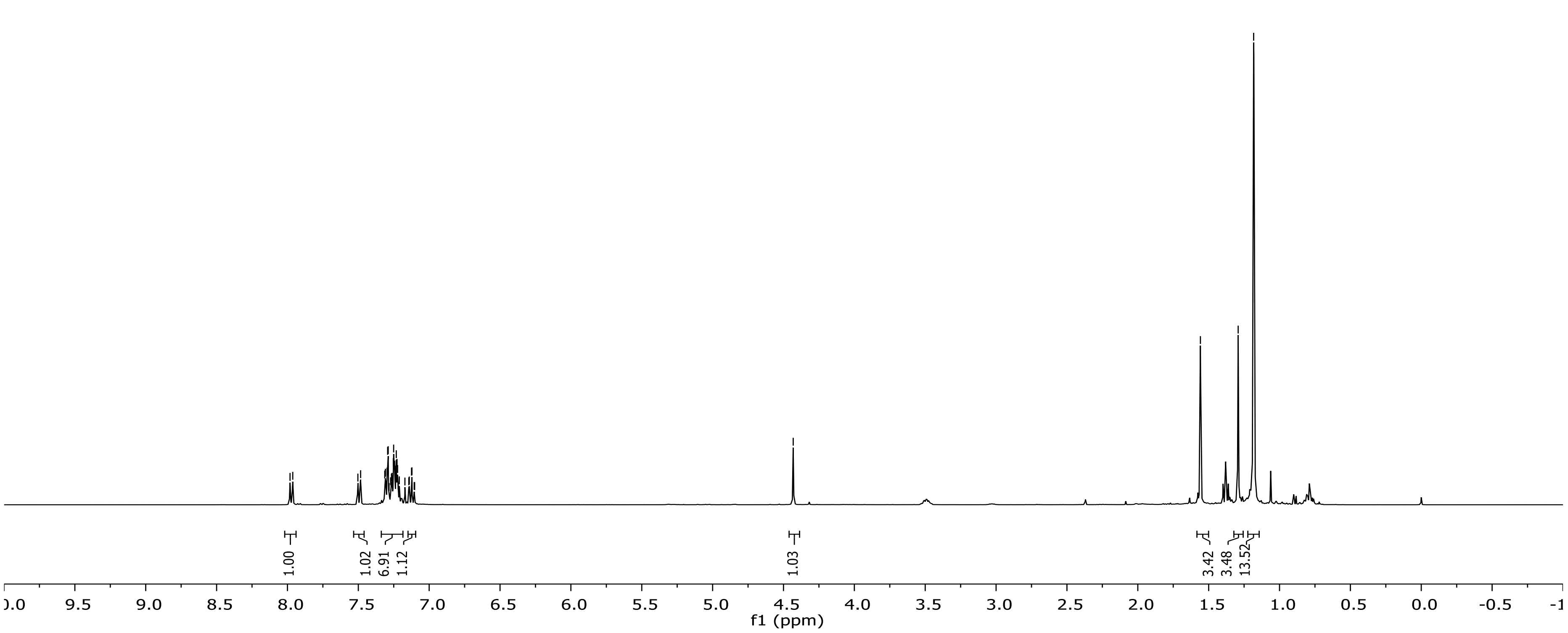

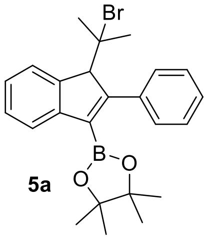

5a

^{13}C-NMR (100 MHz, $CDCl_3$)

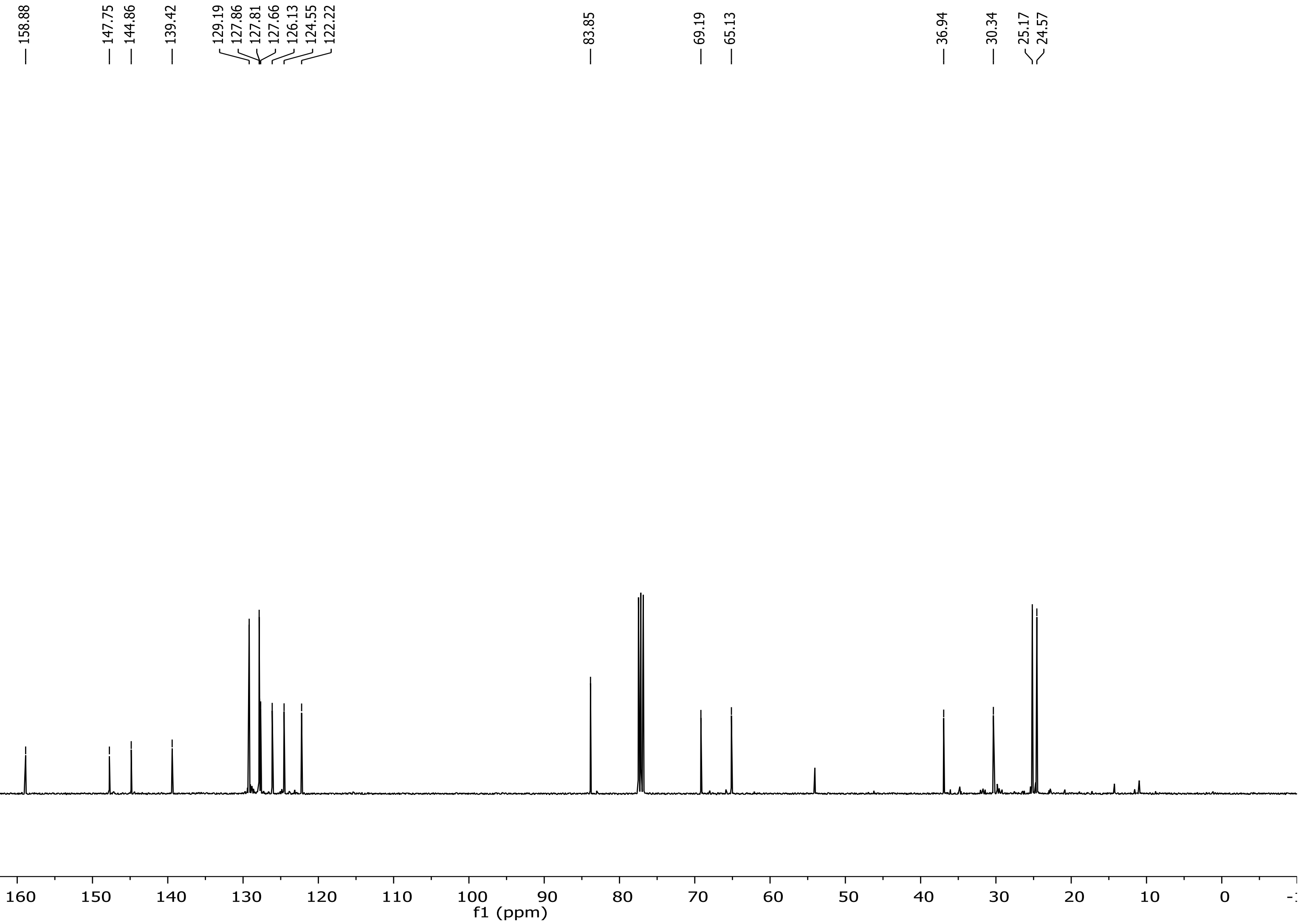

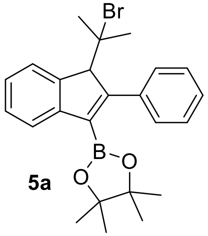

5a

^{11}B-NMR (128 MHz, CDCl$_3$)

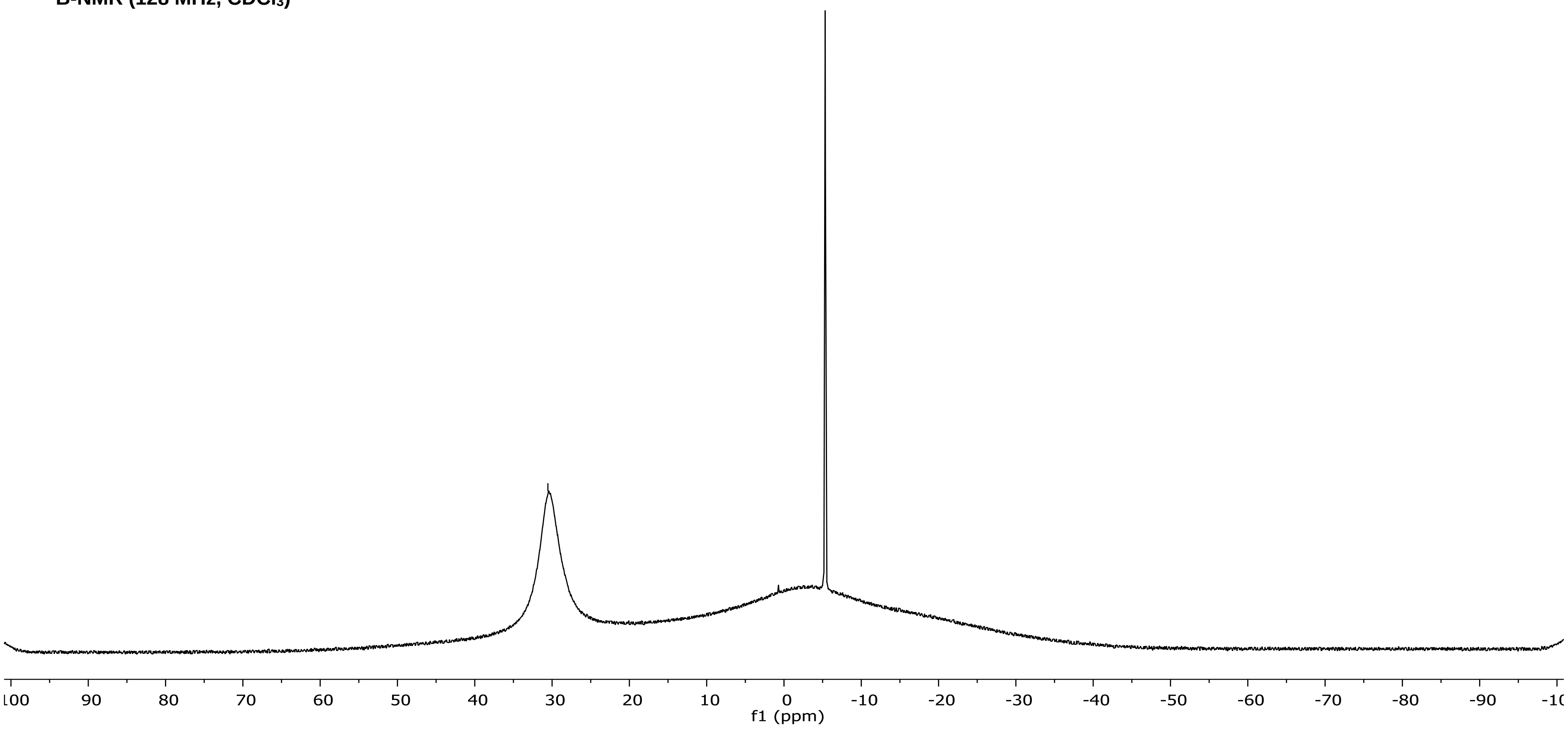

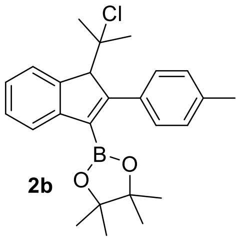

2b

^{1}H-NMR (400 MHz, $CDCl_3$)

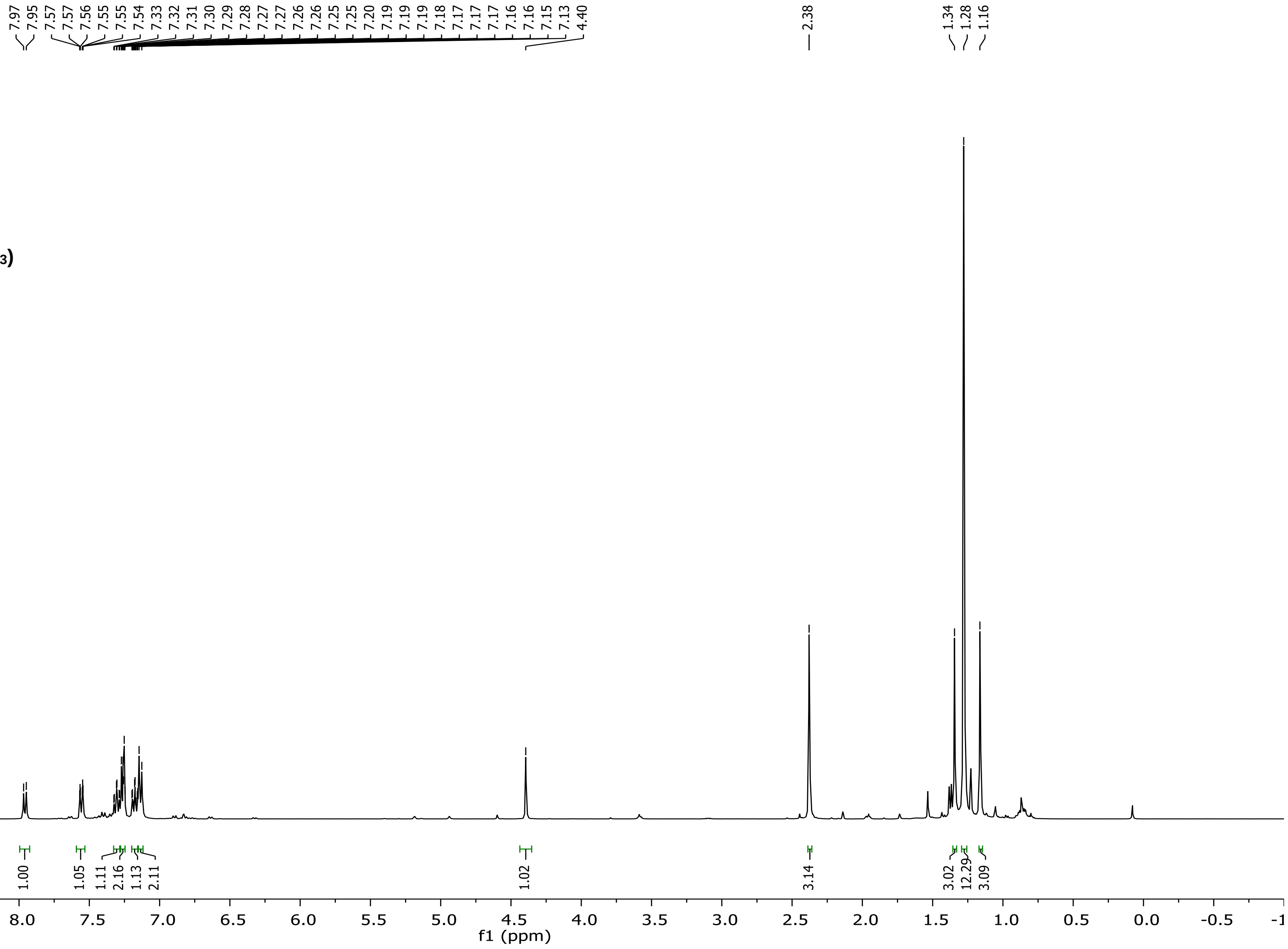

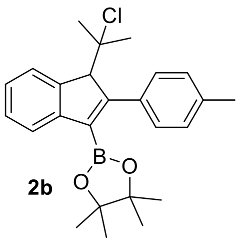

2b

^{13}C-NMR (100 MHz, CDCl$_3$)

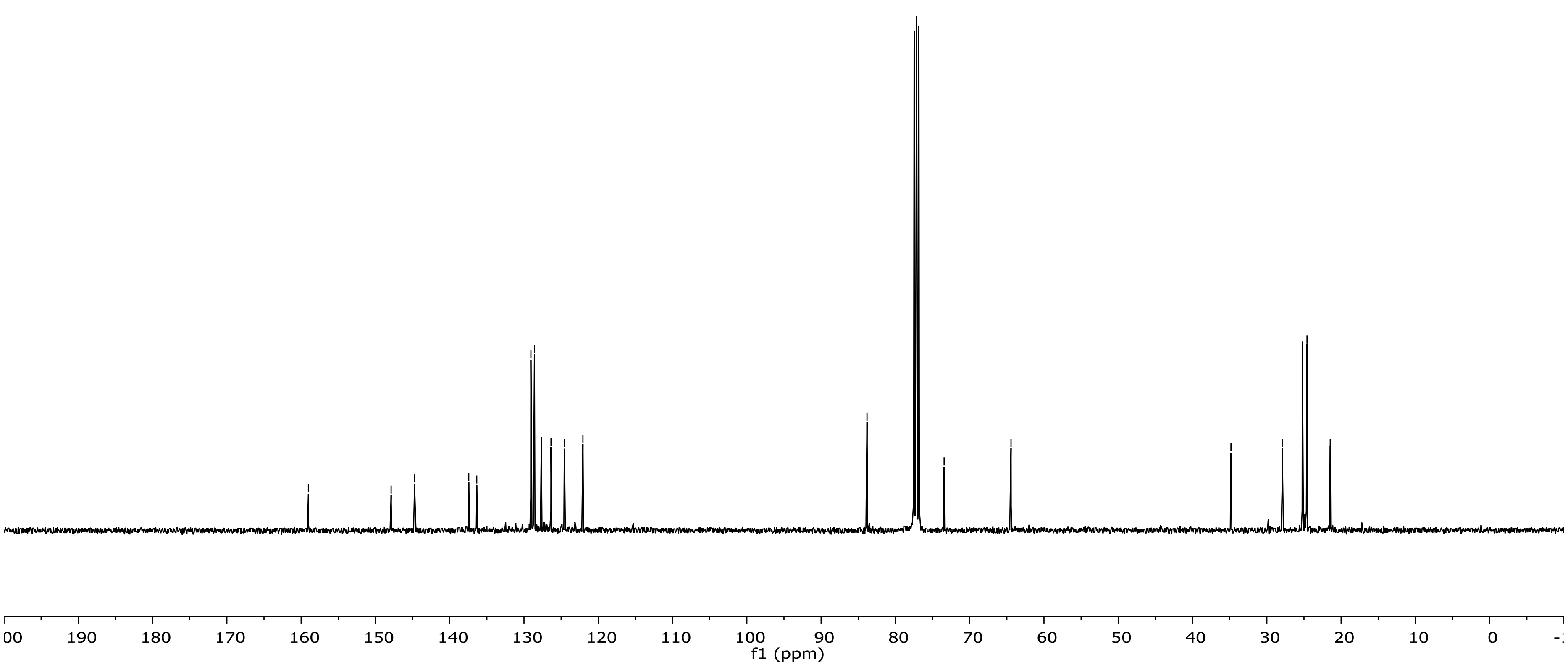

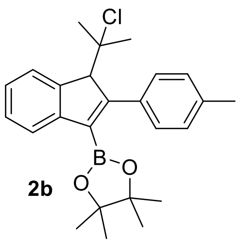

2b

^{11}B-NMR (128 MHz, CDCl$_3$)

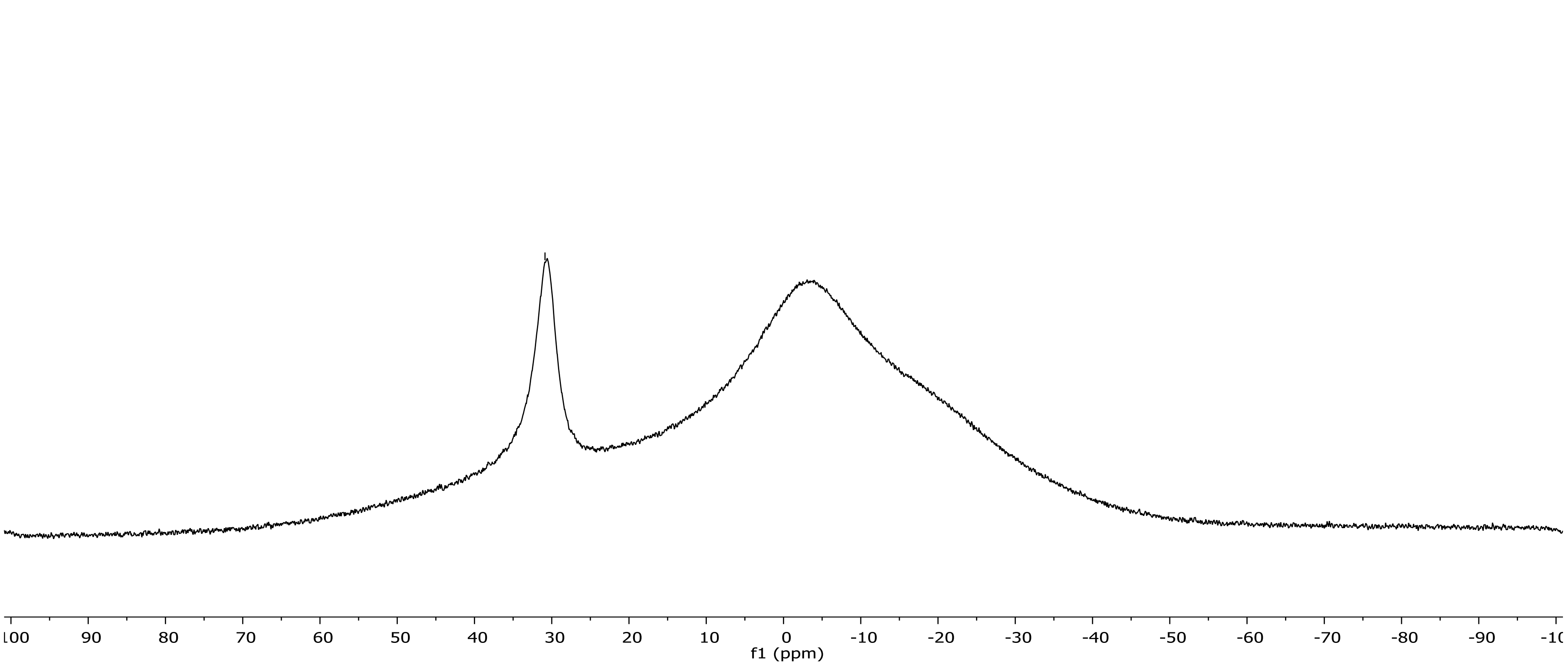

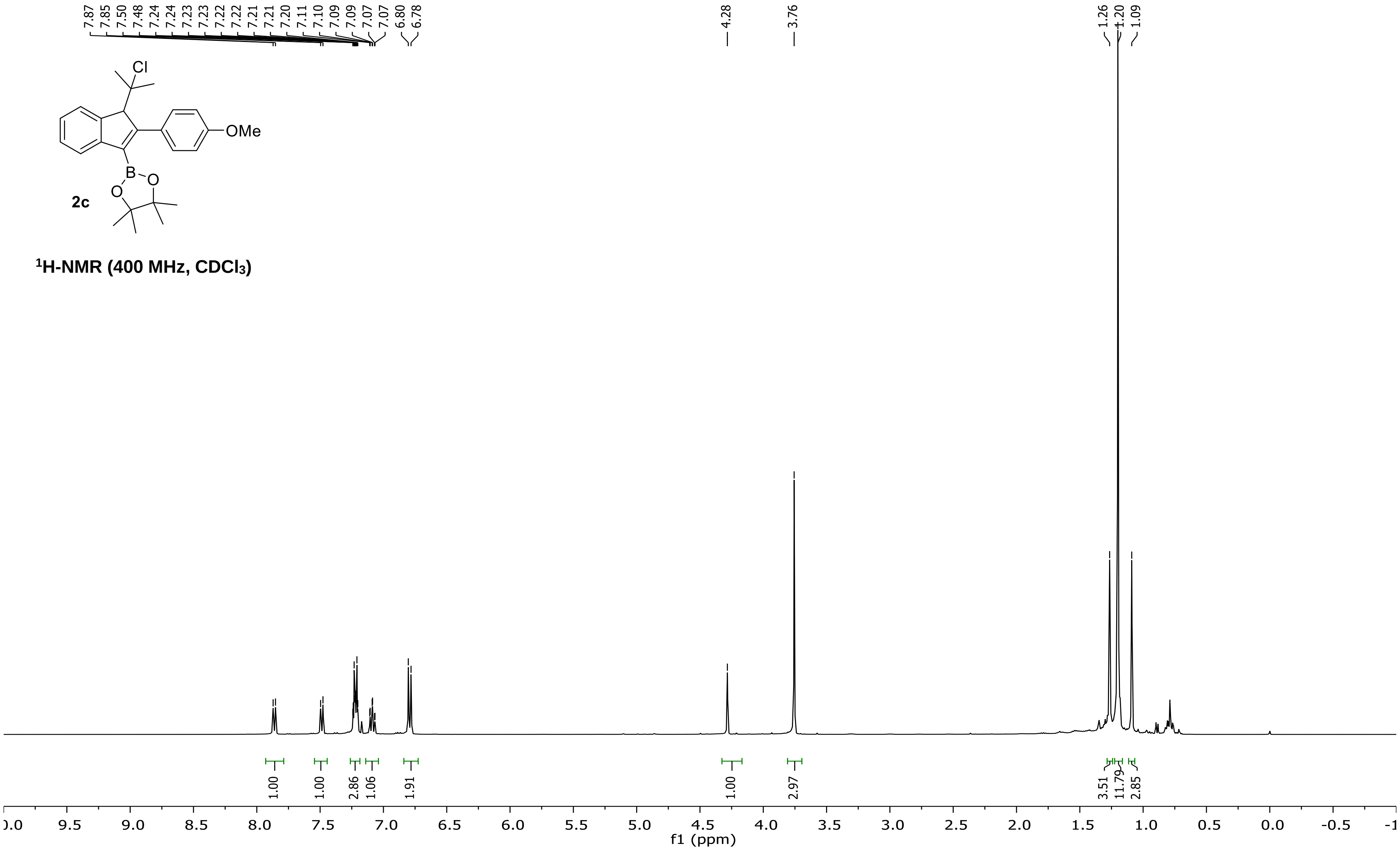

2c

^{1}H-NMR (400 MHz, CDCl$_3$)

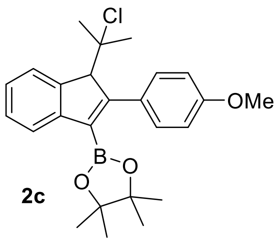

2c

^{13}C-NMR (75 MHz, CDCl$_3$)

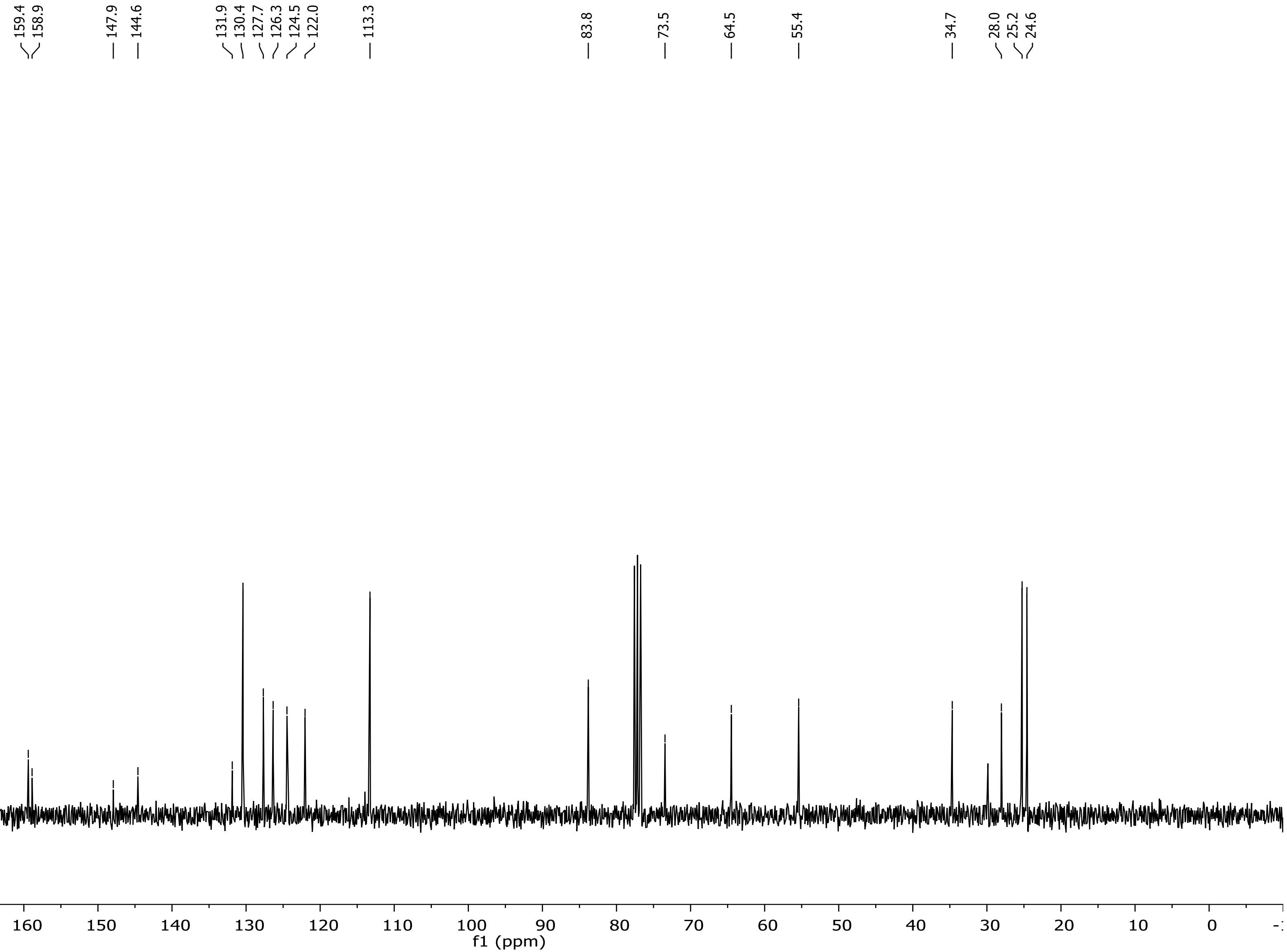

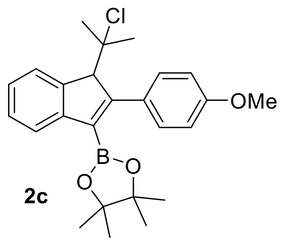

2c

^{11}B-NMR (128 MHz, CDCl$_3$)

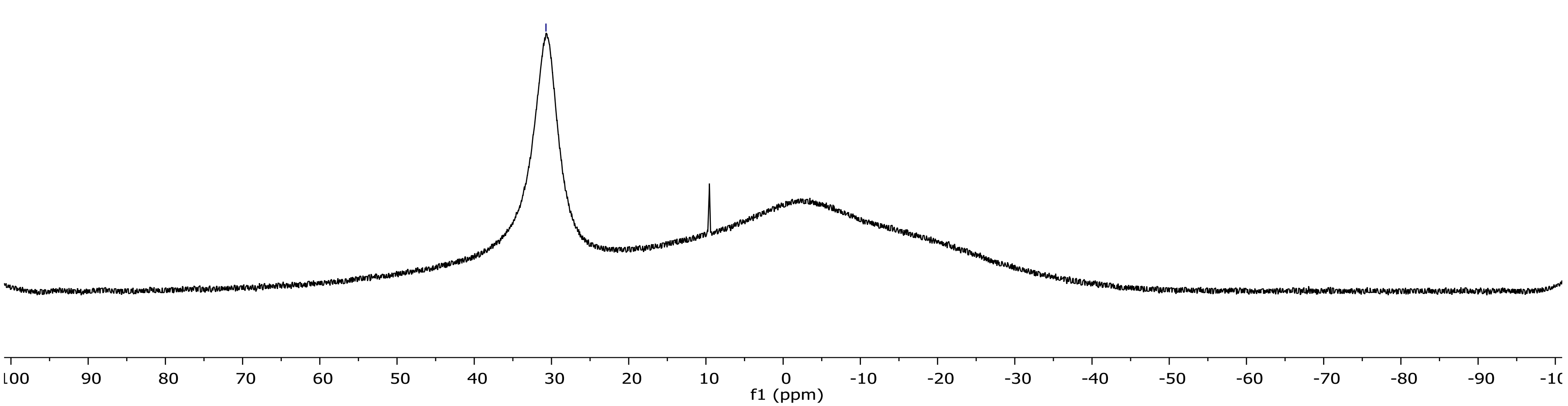

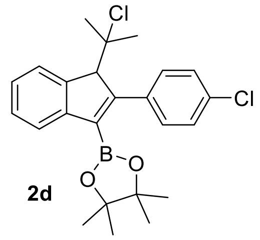

2d

^{1}H-NMR (400 MHz, CDCl$_3$)

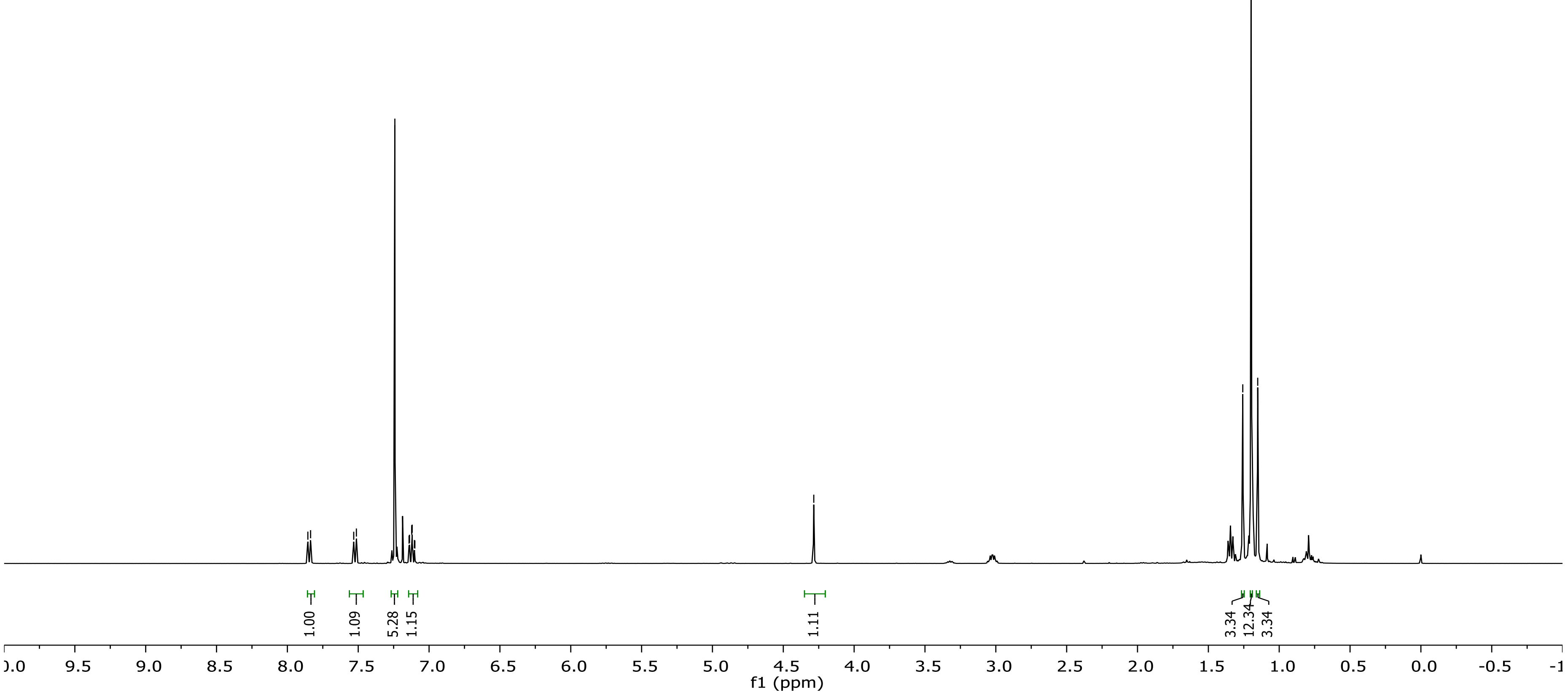

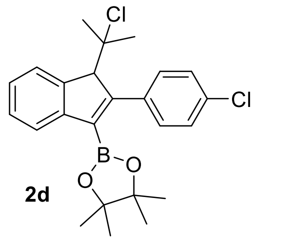

2d

^{13}C-NMR (100 MHz, $CDCl_3$)

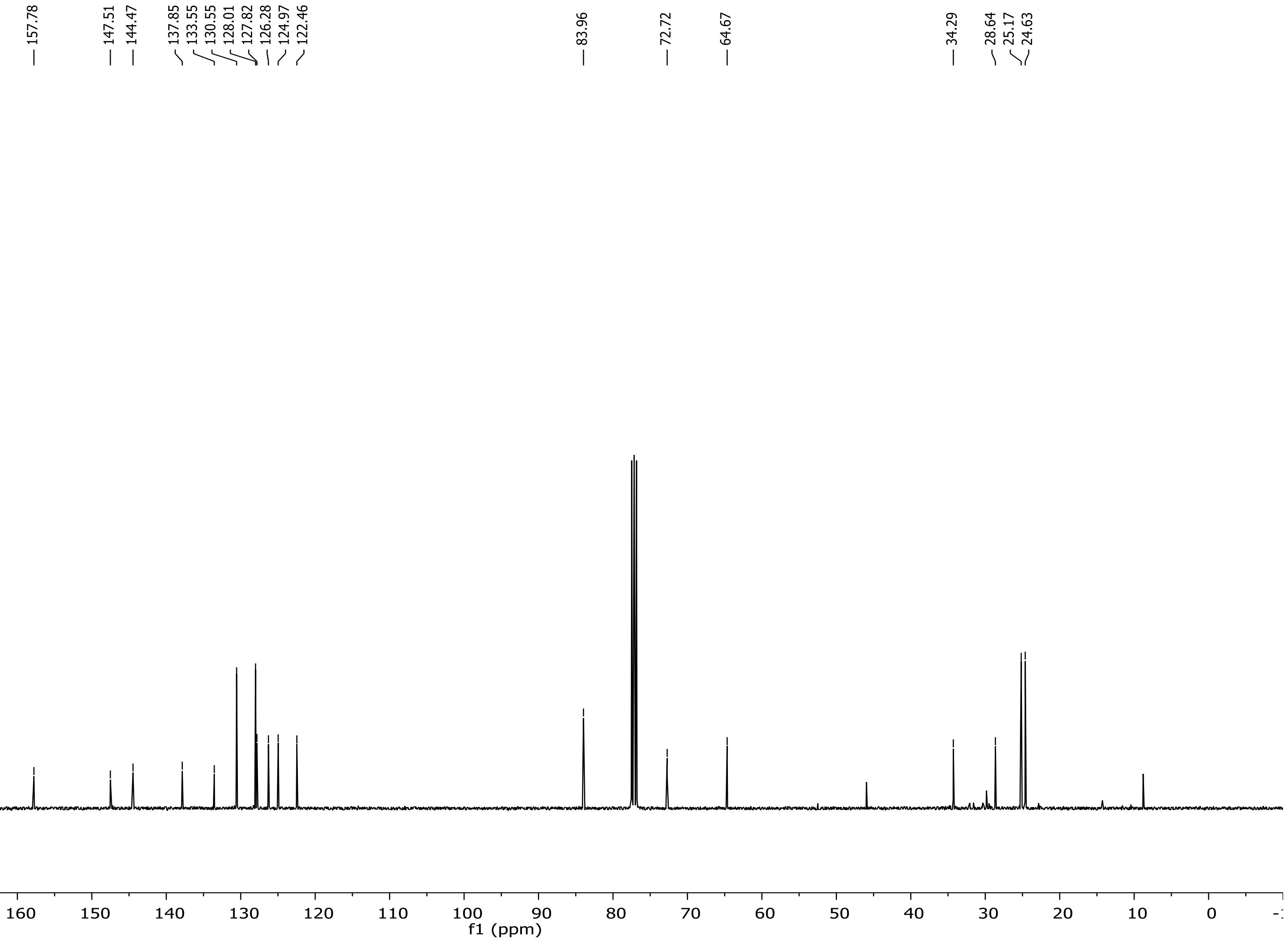

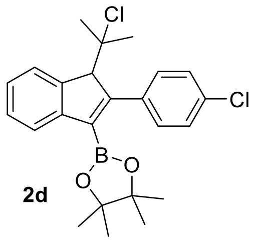

2d

^{11}B-NMR (128 MHz, $CDCl_3$)

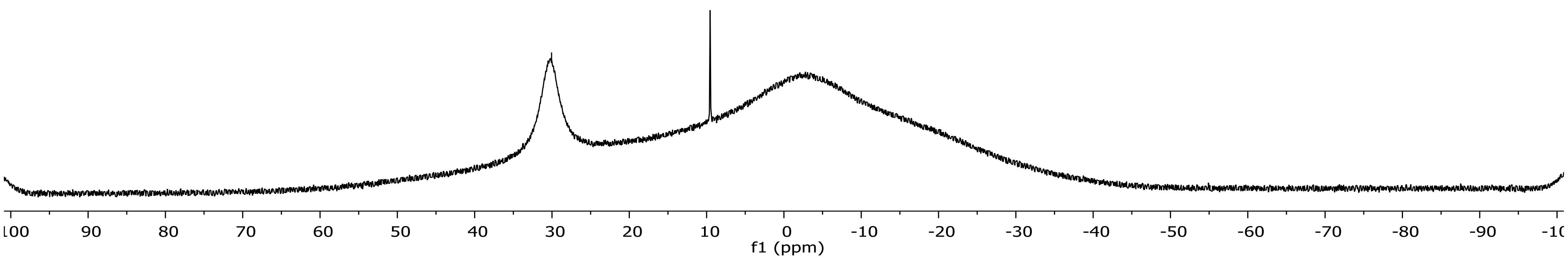

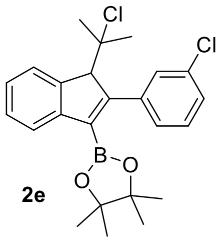

2e

^{1}H-NMR (400 MHz, $CDCl_3$)

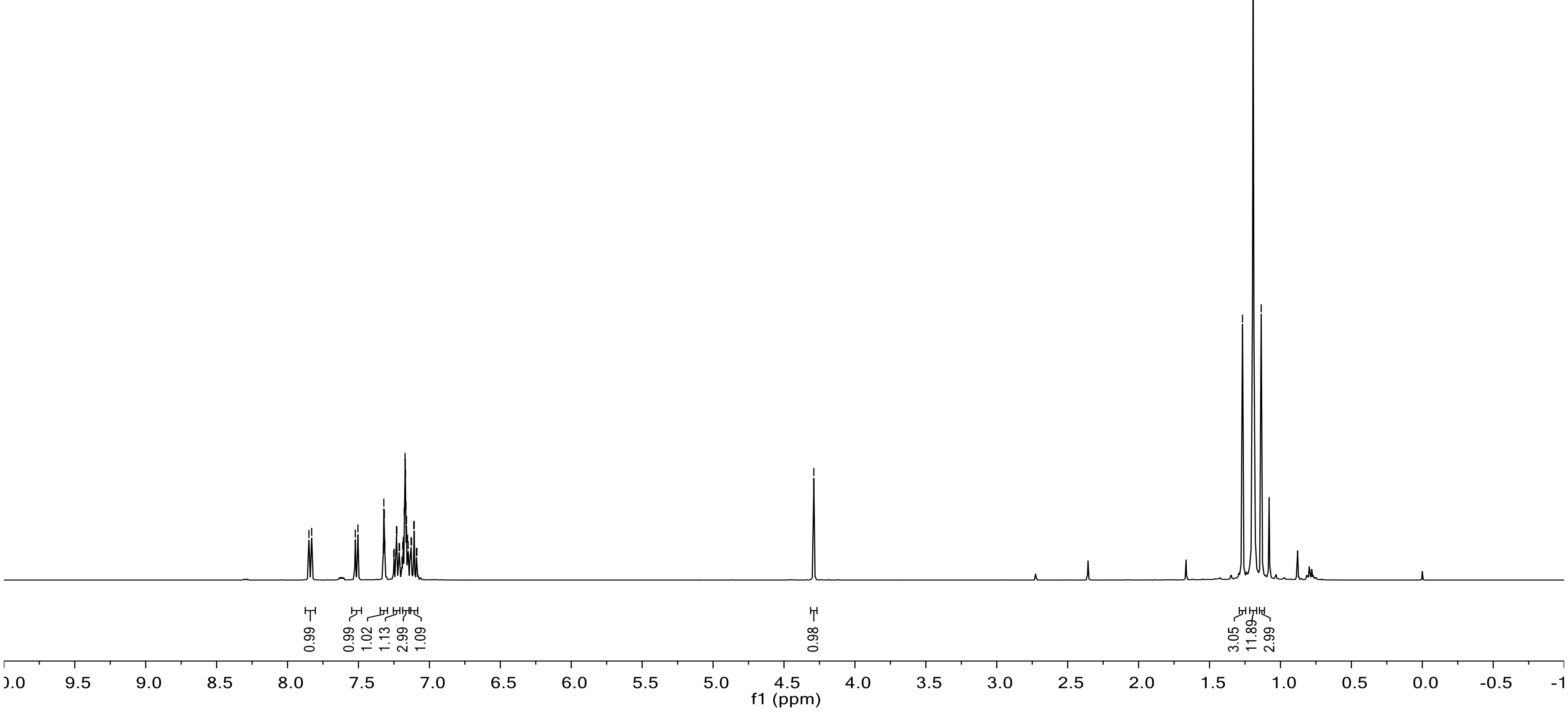

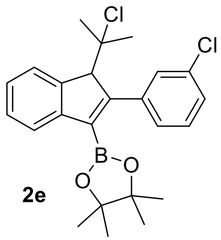

2e

^{13}C-NMR (100 MHz, CDCl$_3$)

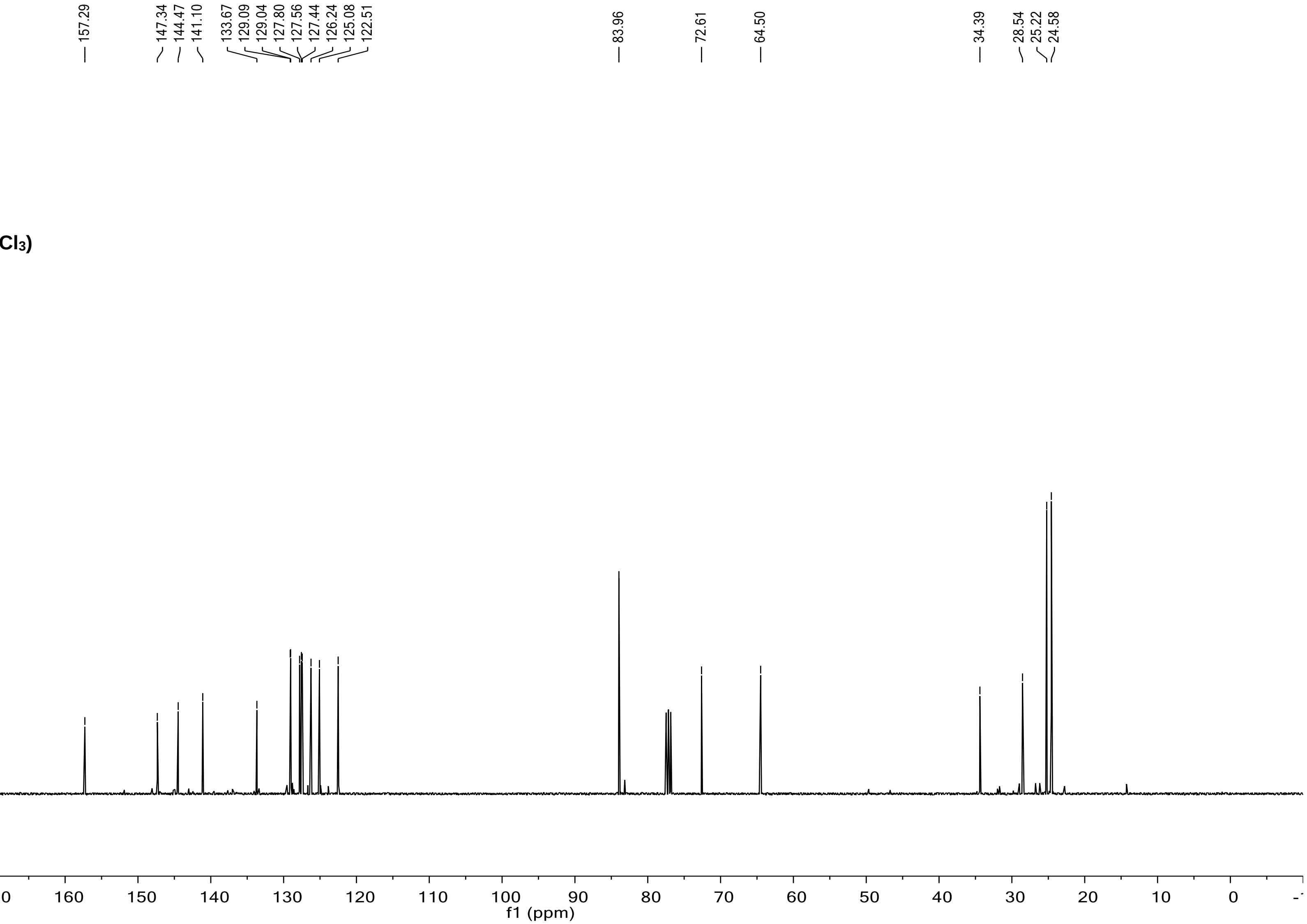

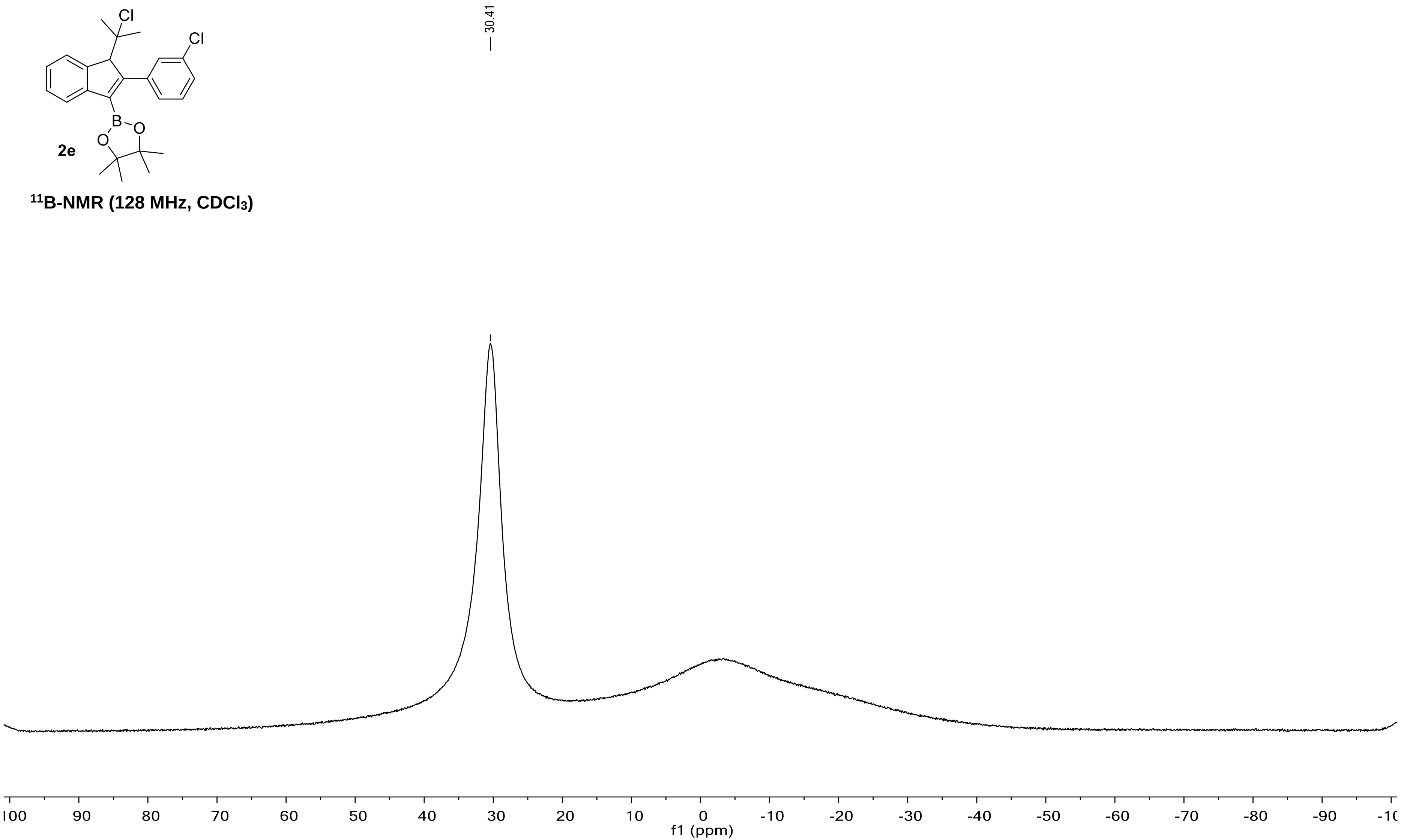

^{11}B-NMR (128 MHz, $CDCl_3$)

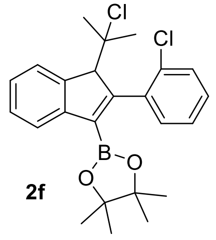

2f

^{1}H-NMR (400 MHz, $CDCl_3$)

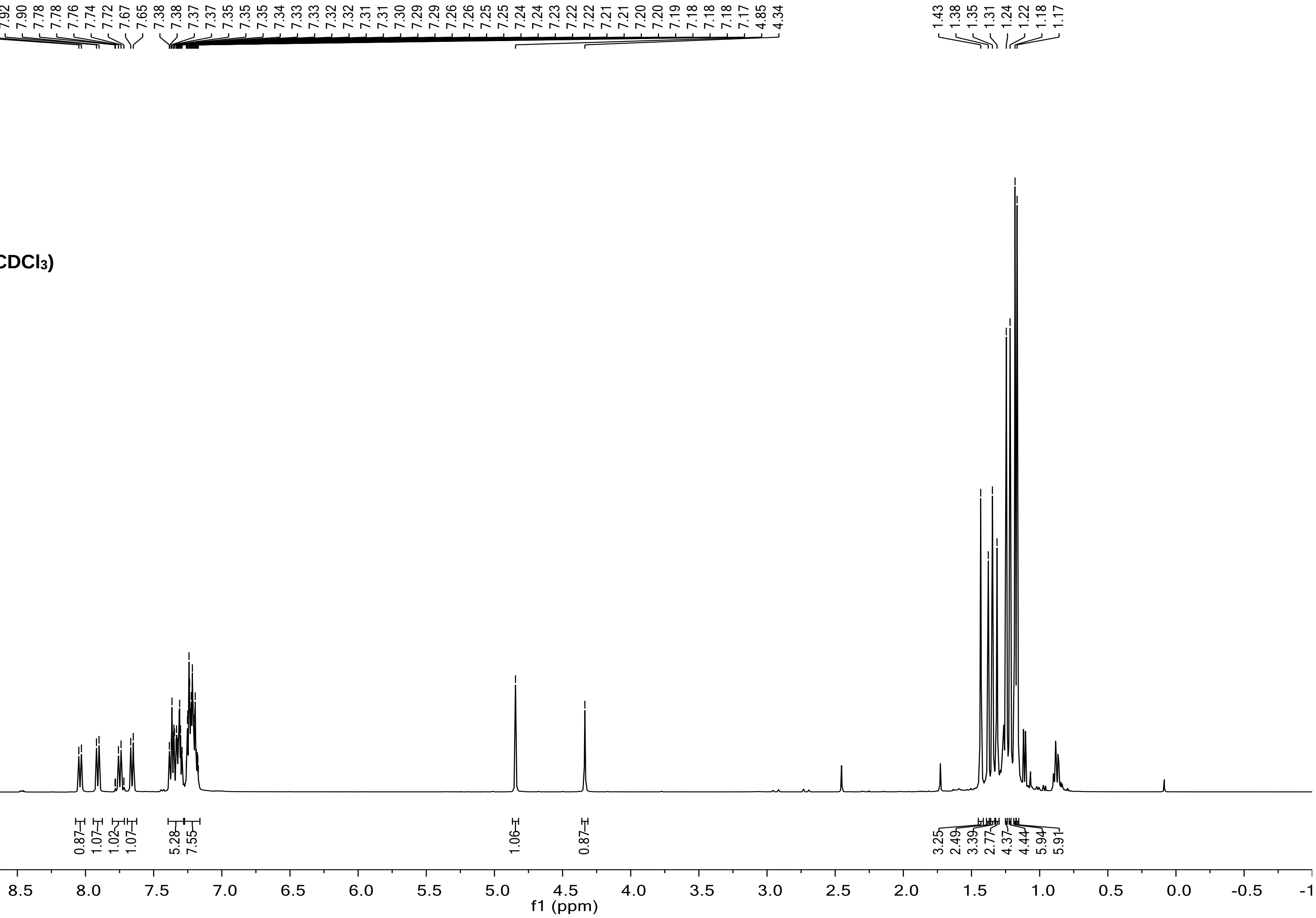

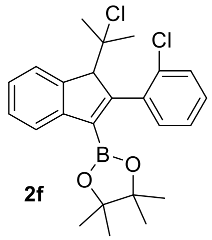

2f

^{13}C-NMR (100 MHz, CDCl$_3$)

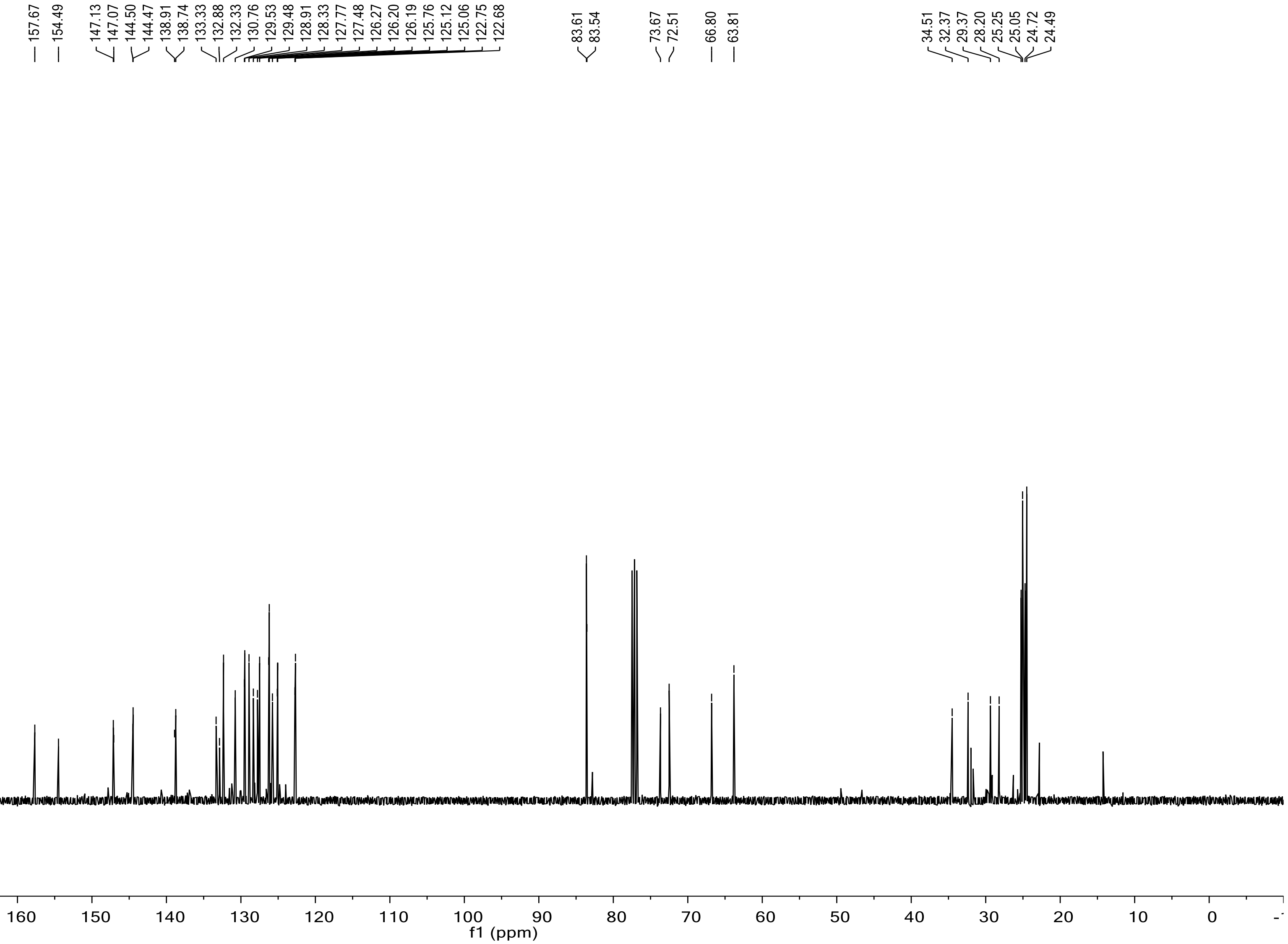

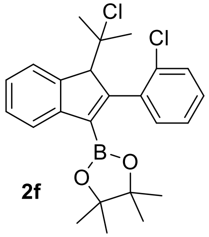

2f

^{11}B-NMR (128 MHz, $CDCl_3$)

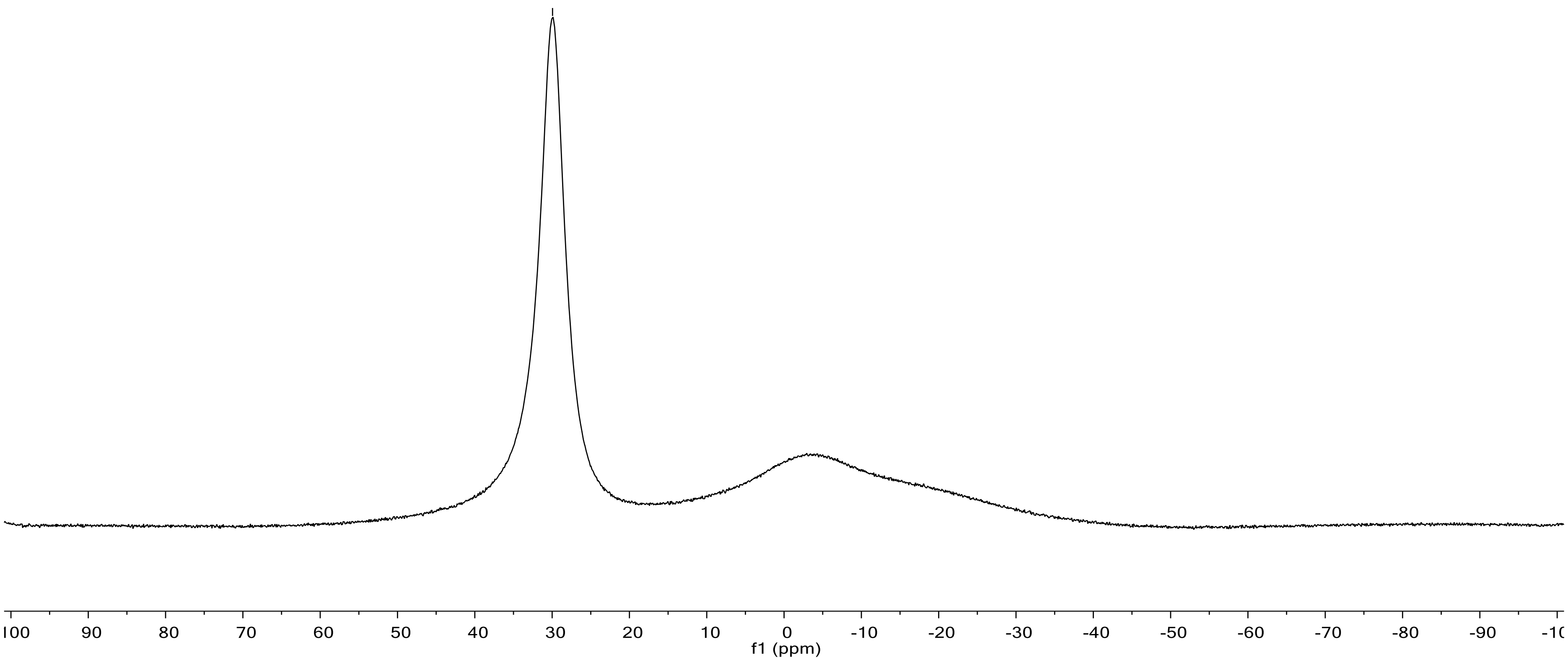

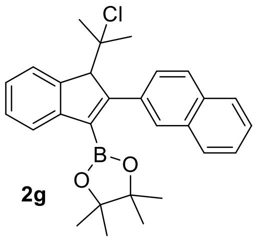

2g

^{1}H-NMR (400 MHz, $CDCl_3$)

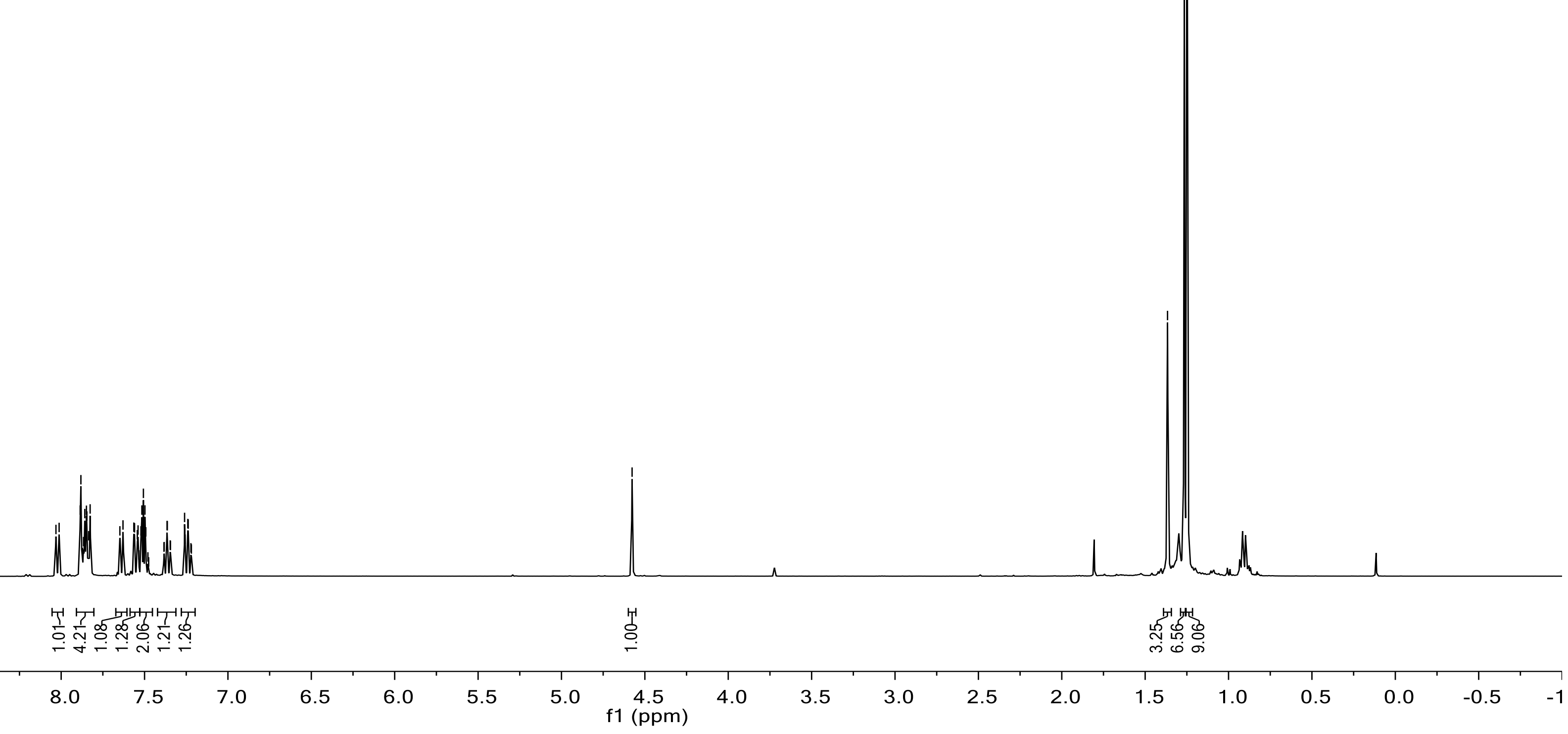

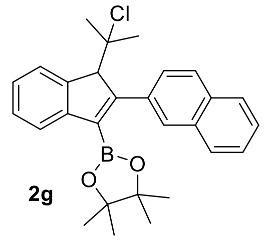

2g

^{13}C-NMR (100 MHz, $CDCl_3$)

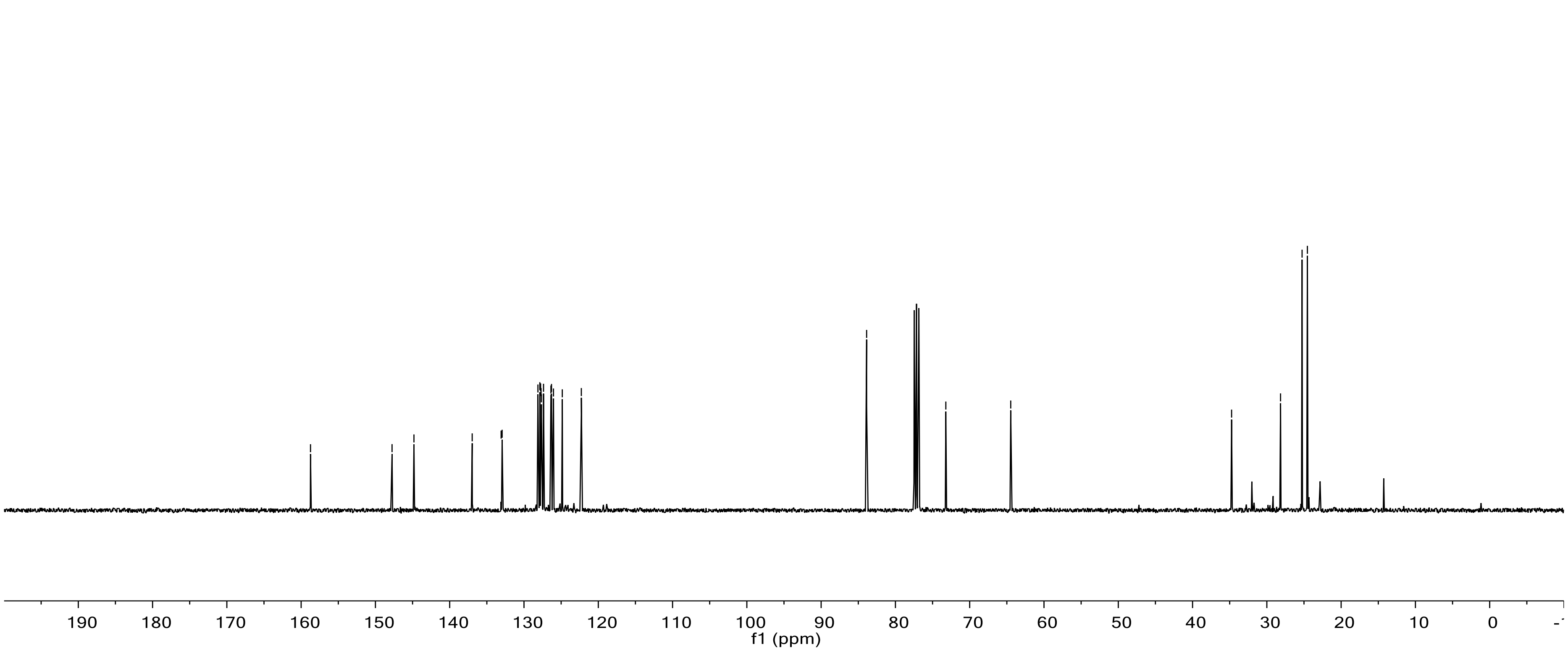

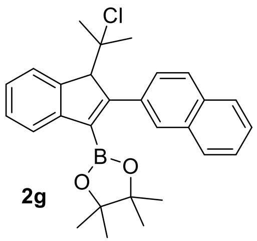

2g

^{11}B-NMR (128 MHz, CDCl$_3$)

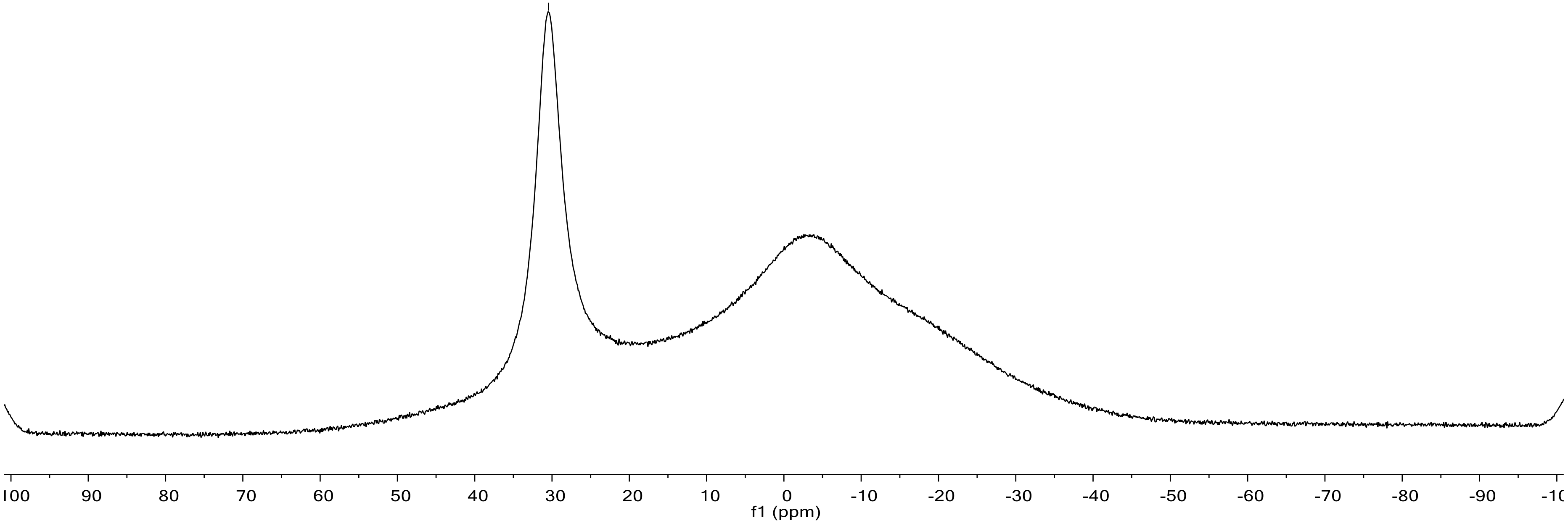

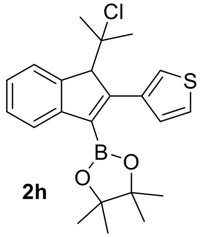

2h

^{1}H-NMR (400 MHz, CDCl$_3$)

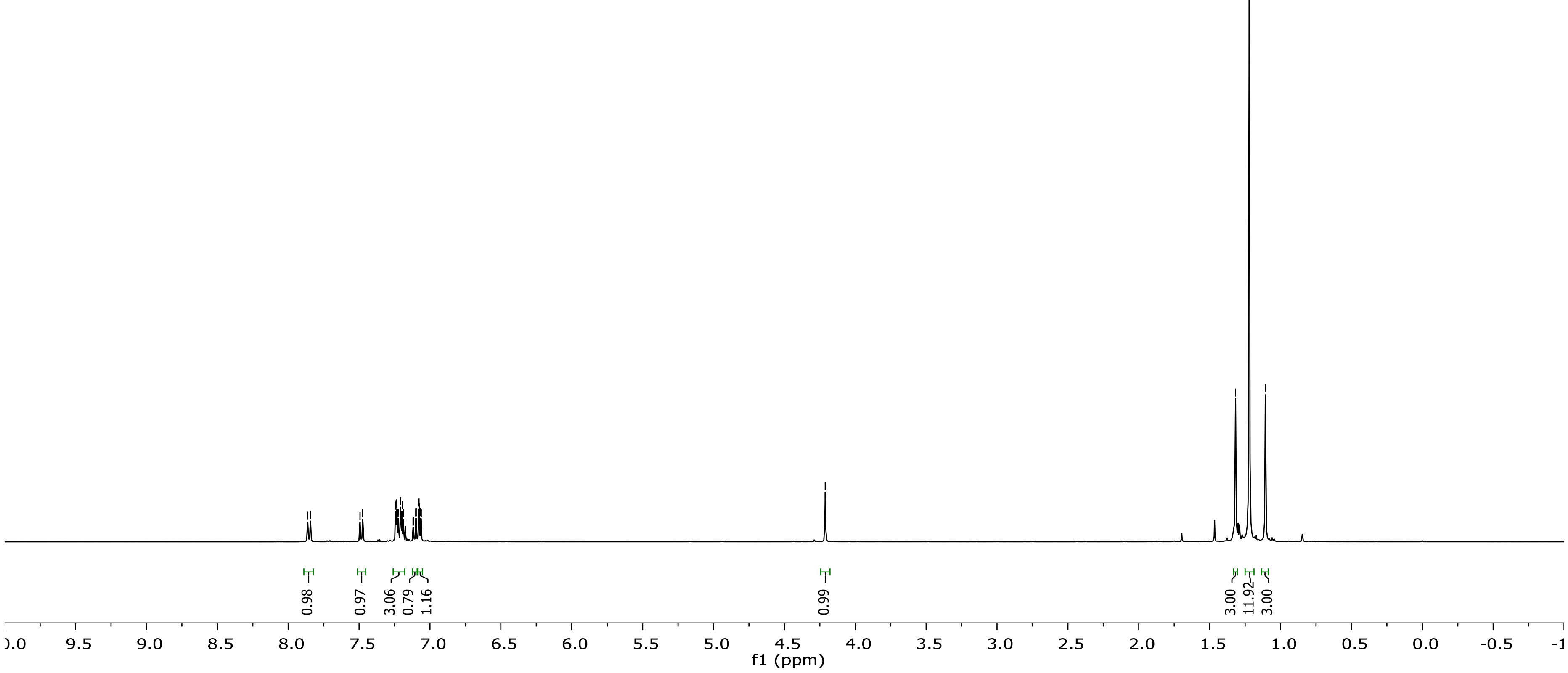

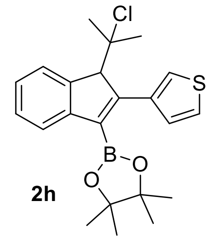

2h

^{13}C-NMR (100 MHz, CDCl$_3$)

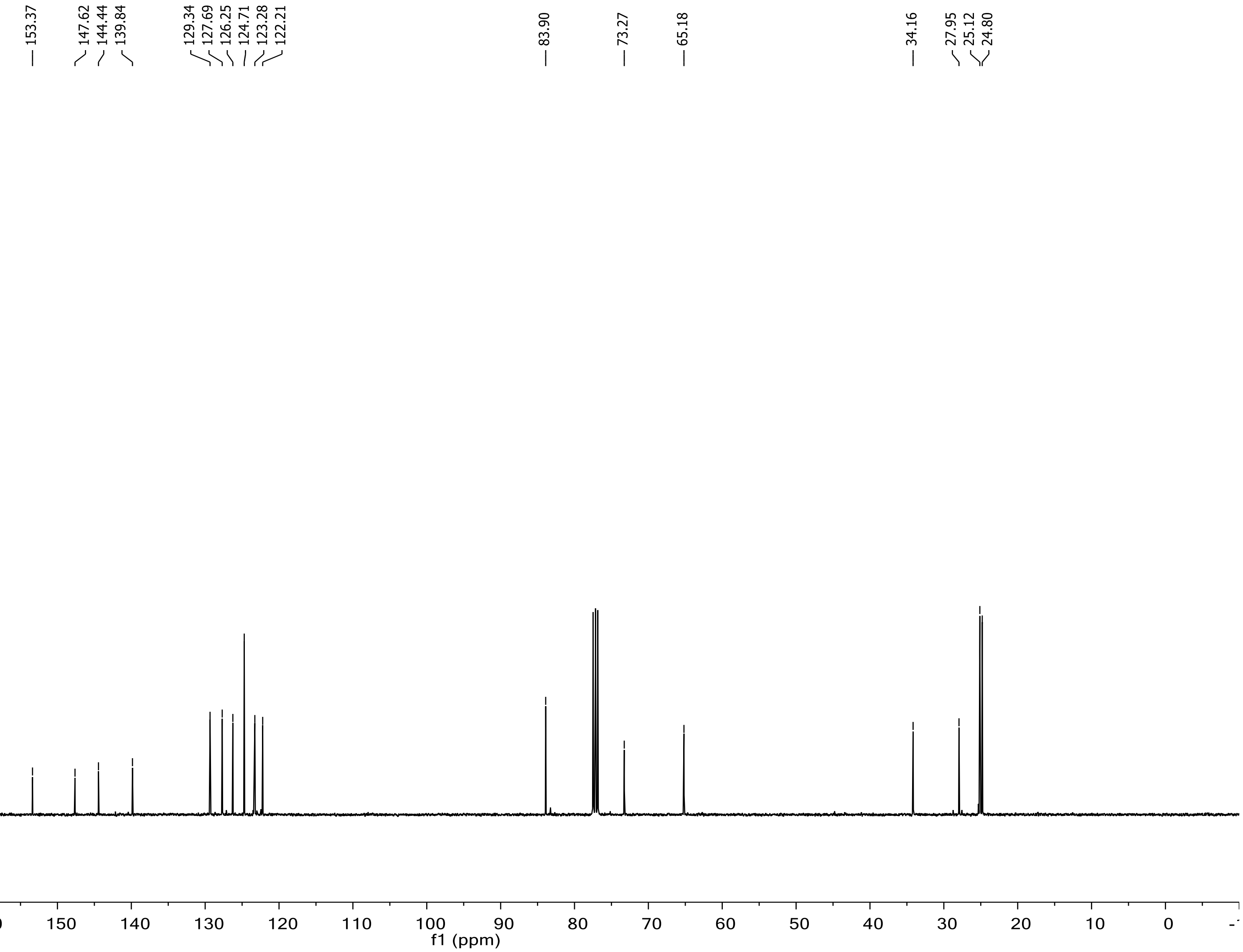

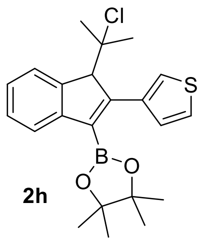

2h

^{11}B-NMR (128 MHz, $CDCl_3$)

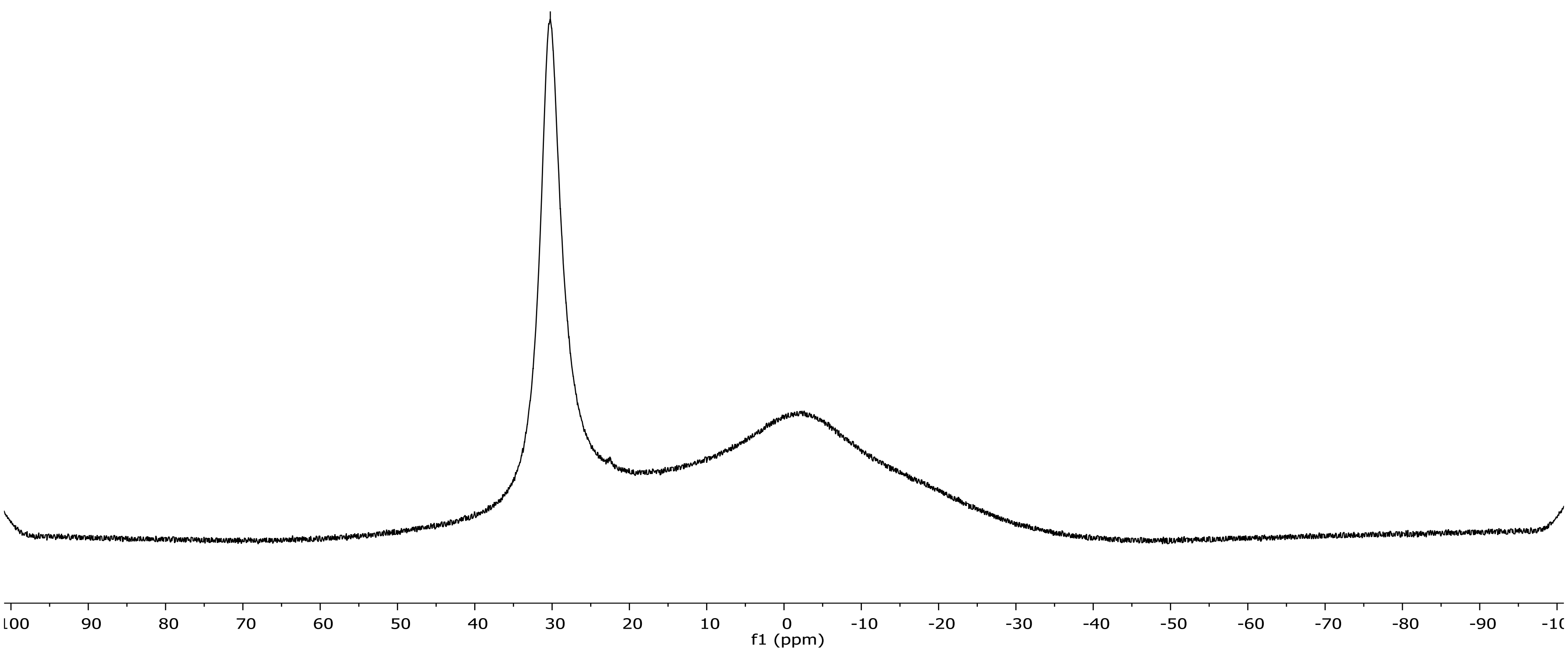

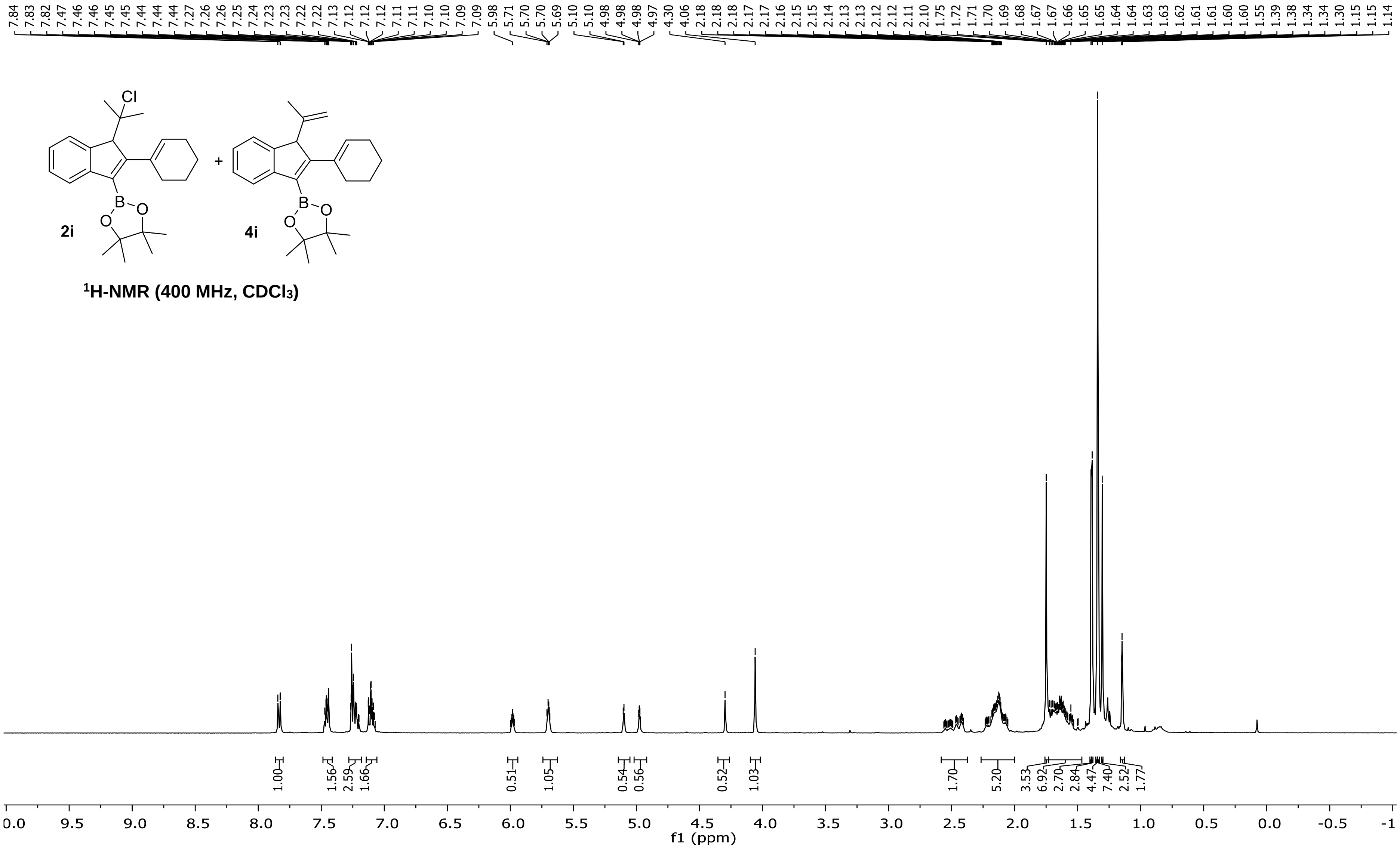
7.84 7.83 7.82 7.47 7.46 7.46 7.45 7.45 7.44 7.44 7.44 7.27 7.26 7.26 7.25 7.24 7.23 7.23 7.22 7.22 7.13 7.12 7.12 7.12 7.12 7.11 7.11 7.10 7.10 7.09 7.09 5.98 5.71 5.70 5.70 5.69 5.10 5.10 4.98 4.98 4.98 4.97 4.30 4.06 2.18 2.18 2.18 2.17 2.17 2.16 2.15 2.15 2.14 2.13 2.13 2.12 2.12 2.11 2.10 1.75 1.72 1.71 1.70 1.69 1.68 1.67 1.67 1.66 1.65 1.65 1.64 1.64 1.63 1.63 1.62 1.61 1.61 1.60 1.60 1.55 1.39 1.38 1.34 1.34 1.30 1.15 1.15 1.14
Cl
+
2i
4i
B
O
O
^{1}H-NMR (400 MHz, CDCl$_3$)
1.00 1.56 2.59 1.66 0.51 1.05 0.54 0.56 0.52 1.03 1.70 5.20 3.53 6.92 2.70 2.84 4.47 7.40 2.52 1.77
0.0 9.5 9.0 8.5 8.0 7.5 7.0 6.5 6.0 5.5 5.0 4.5 4.0 3.5 3.0 2.5 2.0 1.5 1.0 0.5 0.0 -0.5 -1
f1 (ppm)

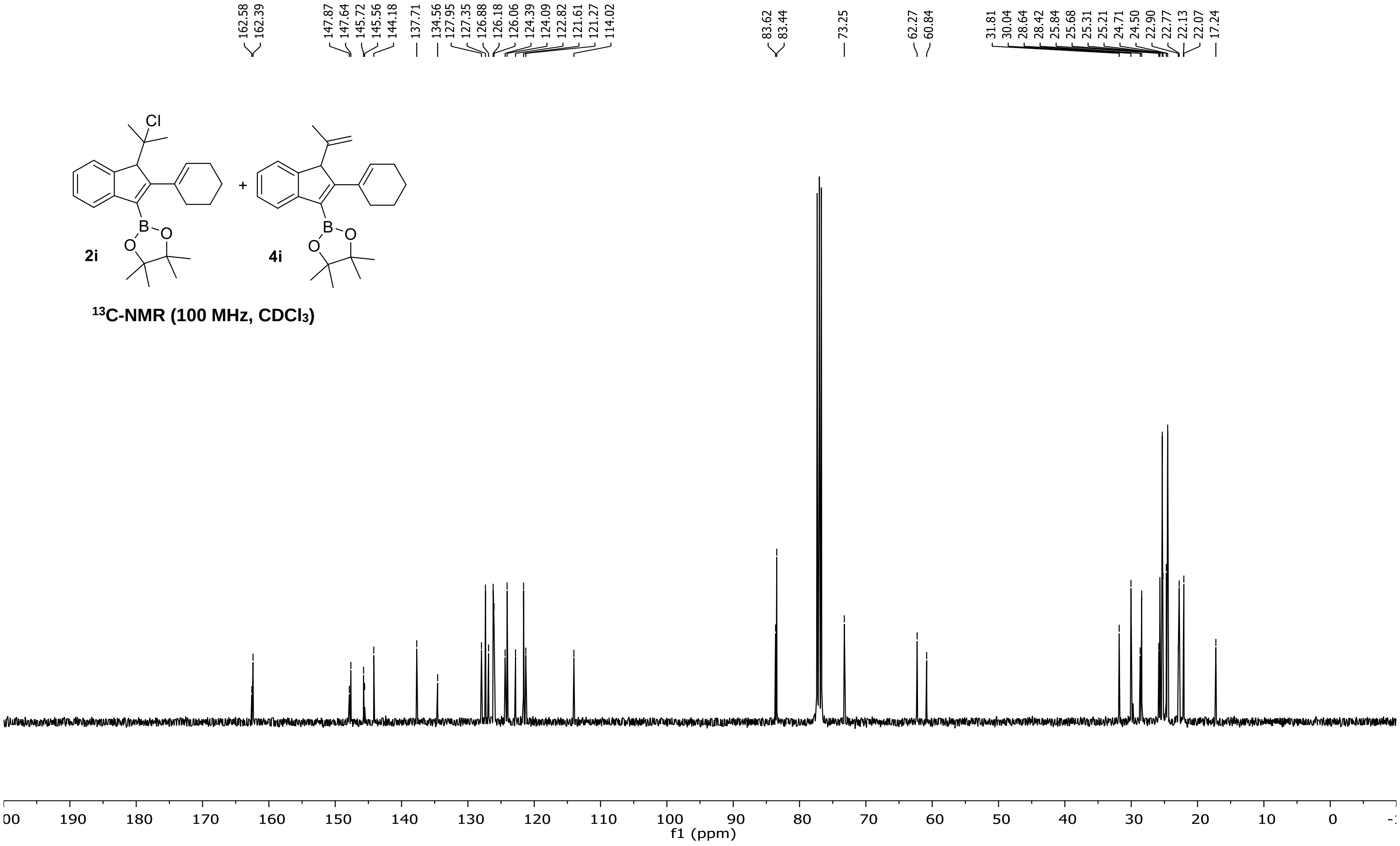
2i
4i
^{13}C-NMR (100 MHz, CDCl$_3$)
162.58
162.39
147.87
147.64
145.72
145.56
144.18
137.71
134.56
127.95
127.35
126.88
126.18
126.06
124.39
124.09
122.82
121.61
121.27
114.02
83.62
83.44
73.25
62.27
60.84
31.81
30.04
28.64
28.42
25.84
25.68
25.31
25.21
24.71
24.50
22.90
22.77
22.13
22.07
17.24
190 180 170 160 150 140 130 120 110 100 90 80 70 60 50 40 30 20 10 0
f1 (ppm)

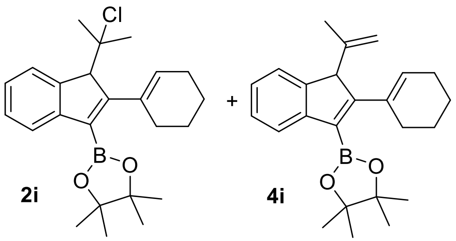

2i + 4i

^{11}B-NMR (128 MHz, CDCl$_3$)

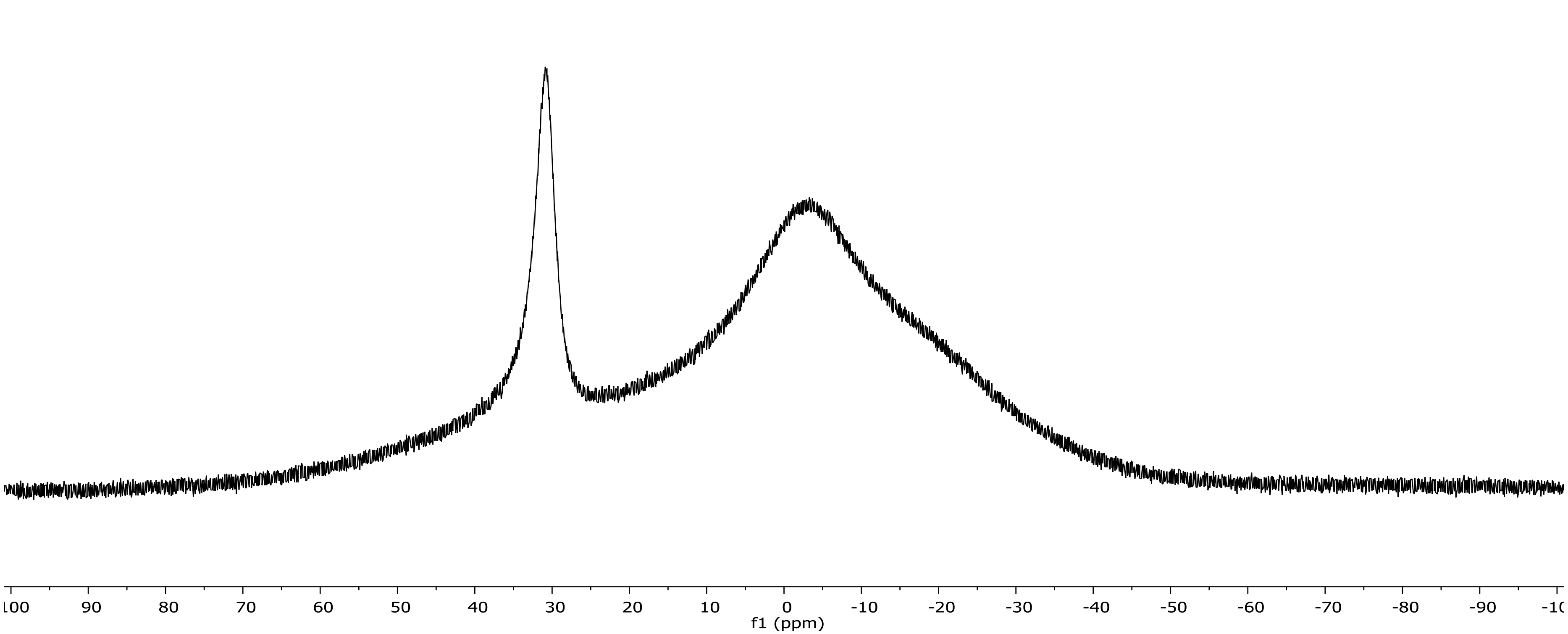

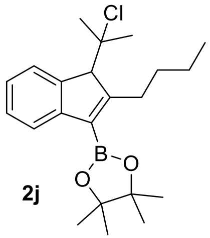

2j

^{1}H-NMR (300 MHz, $CDCl_3$)

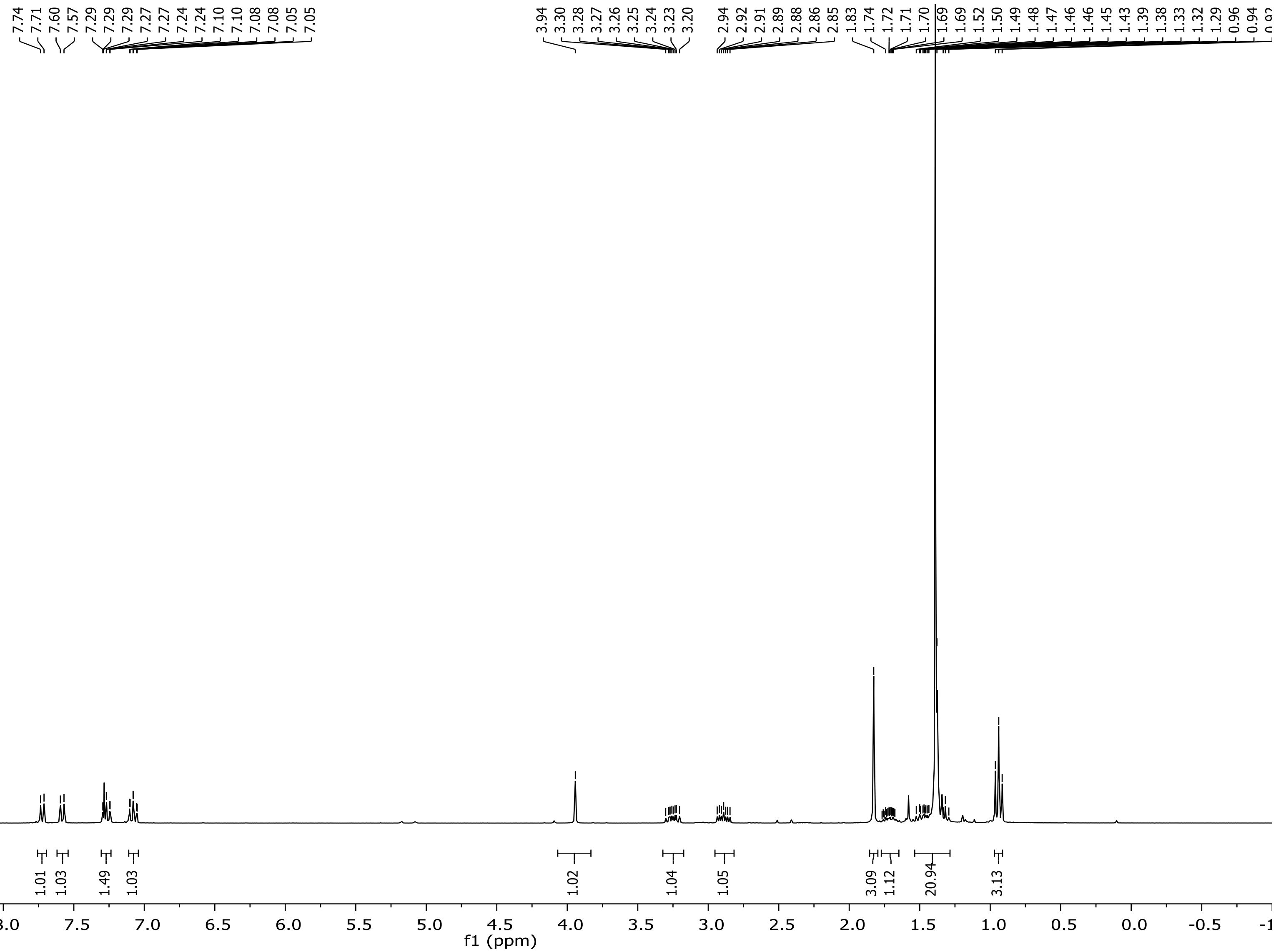

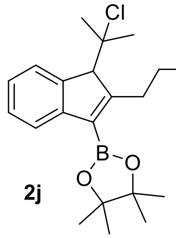

2j

^{13}C-NMR (75 MHz, CDCl$_3$)

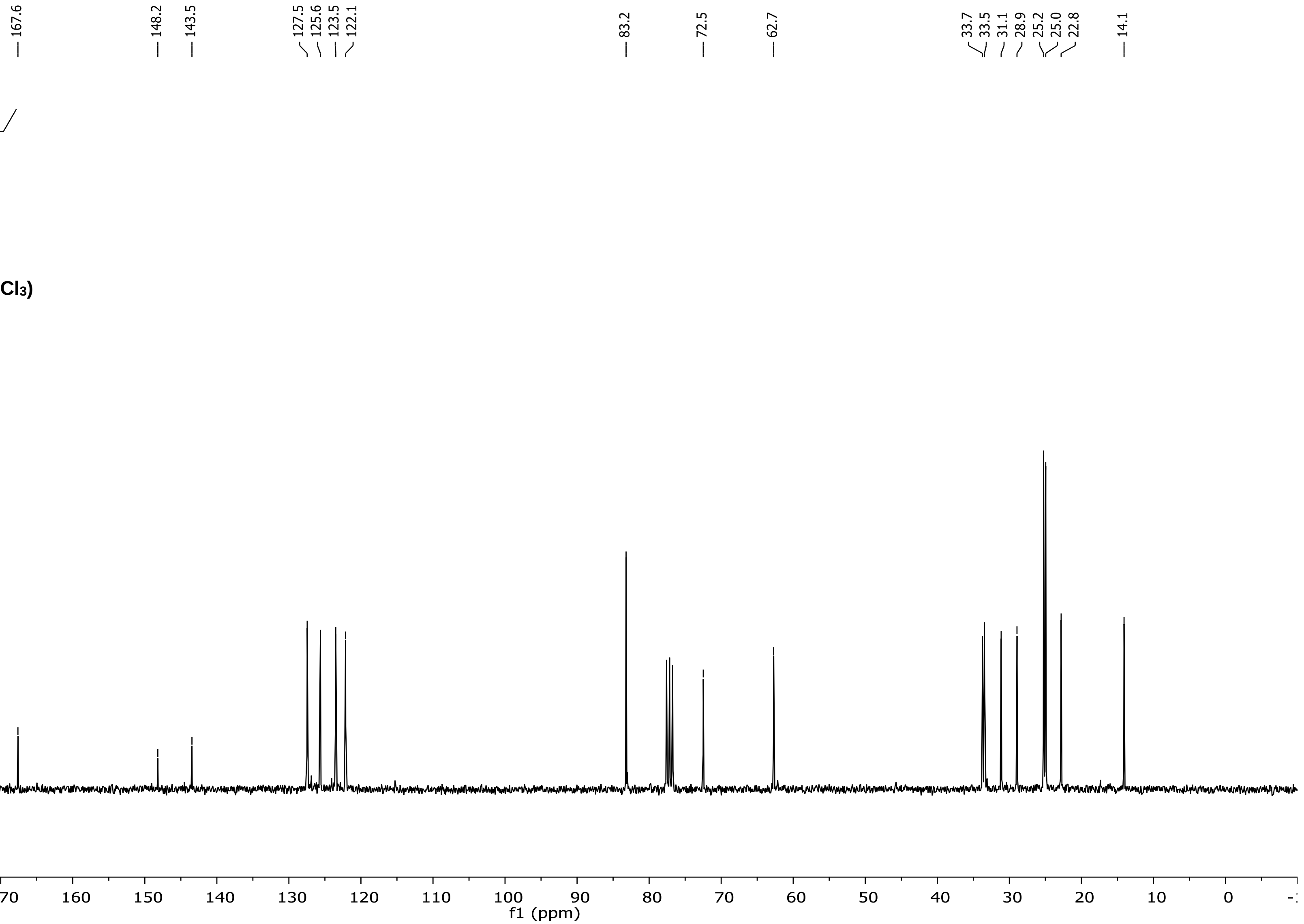

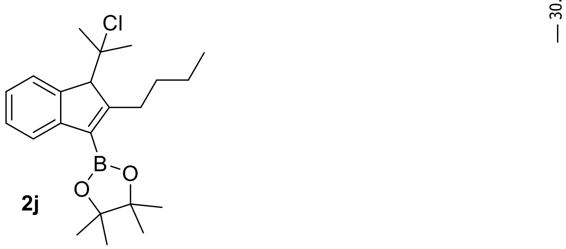

2j

^{11}B-NMR (128 MHz, $CDCl_3$)

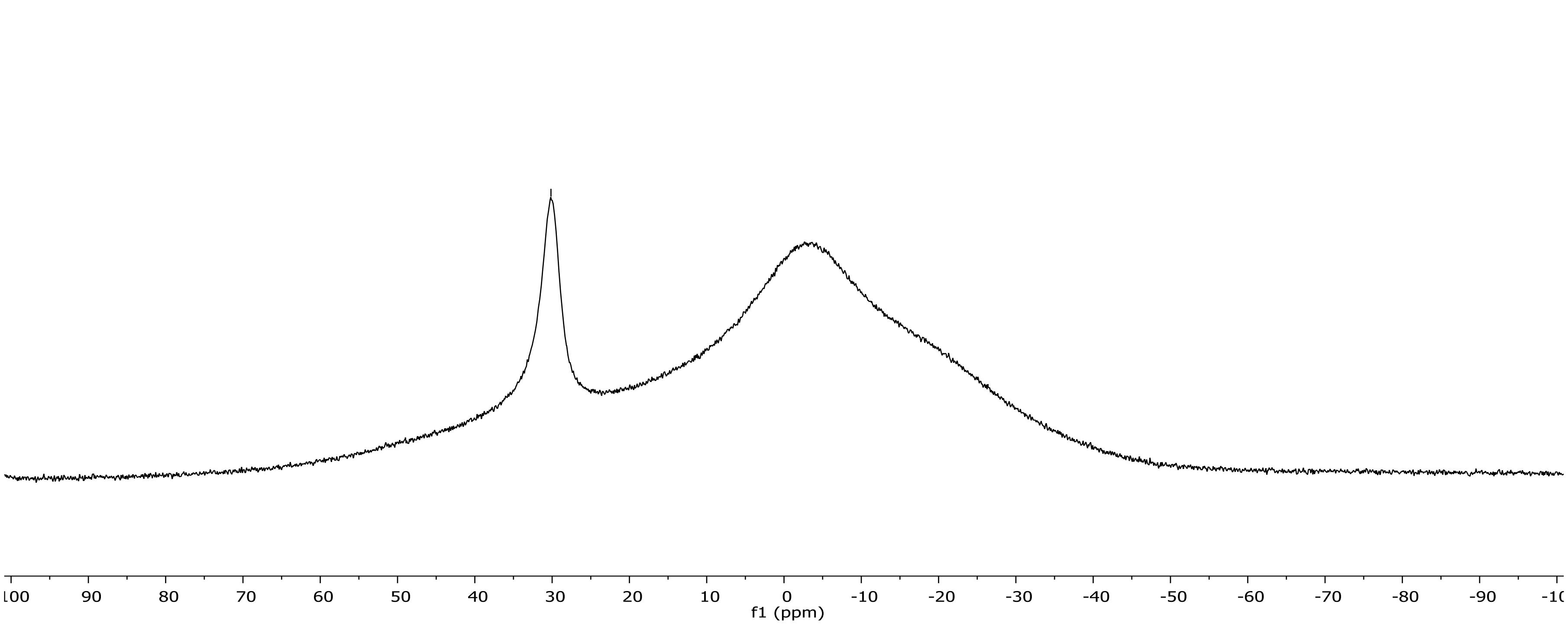

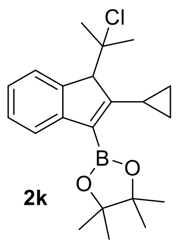

2k

^{1}H-NMR (400 MHz, $CDCl_3$)

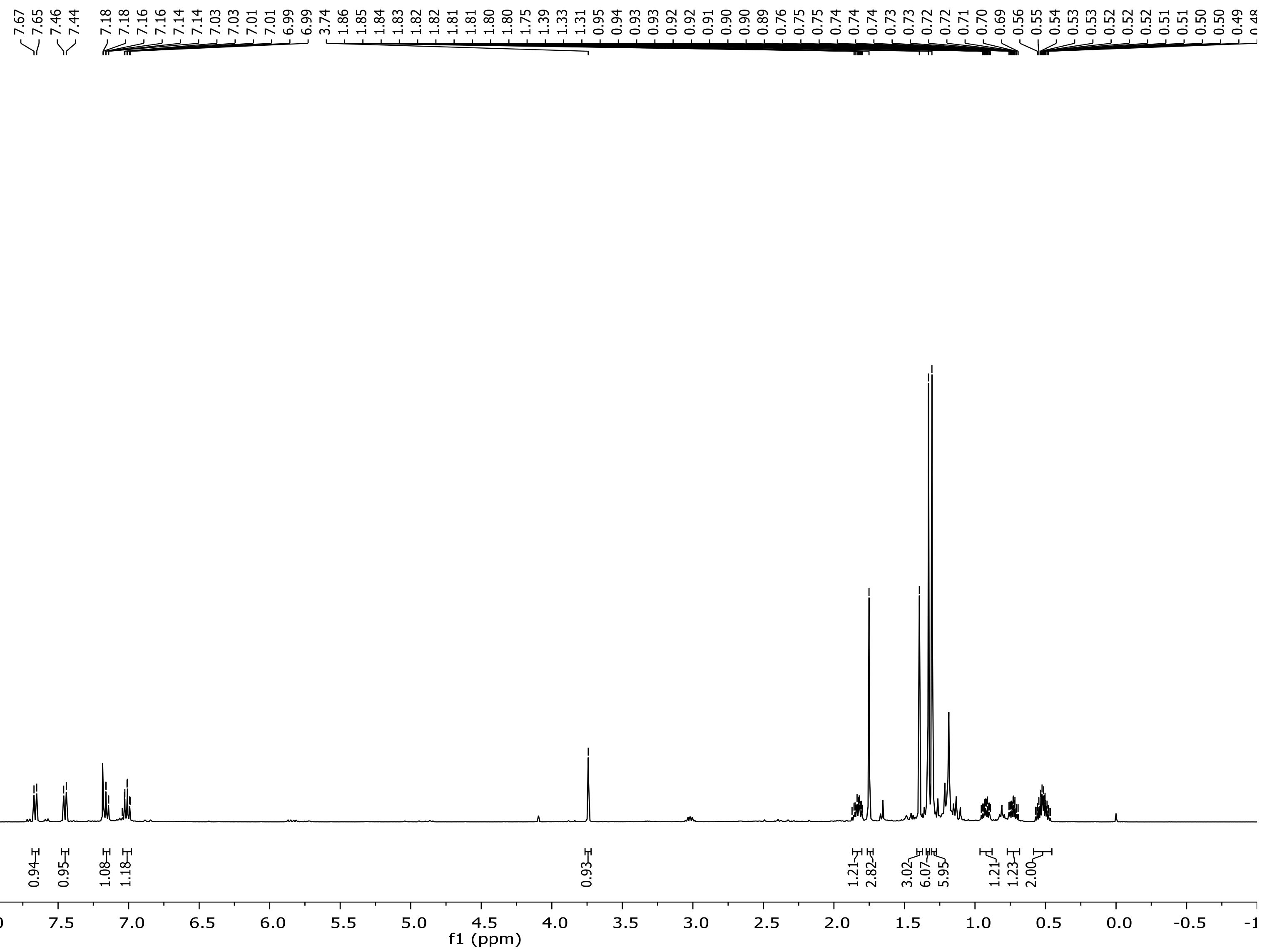

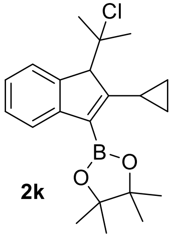

2k

^{13}C-NMR (100 MHz, CDCl$_3$)

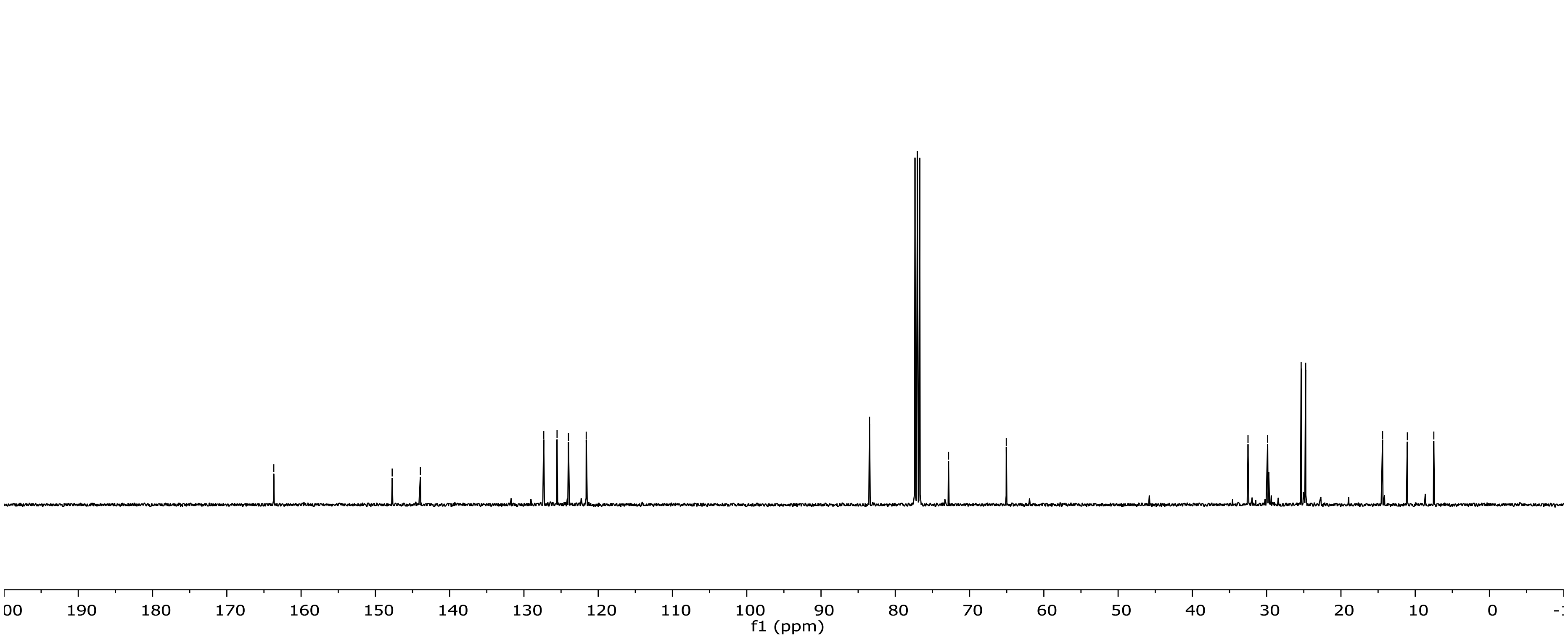

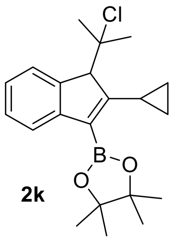

2k

^{11}B-NMR (128 MHz, CDCl3)

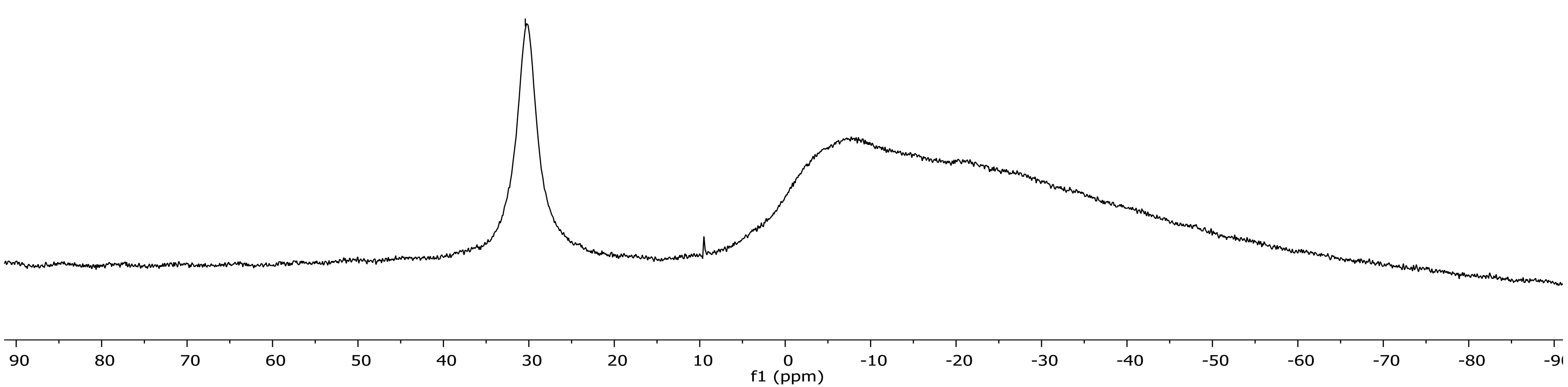

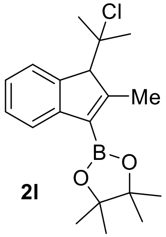

2l

^{1}H-NMR (400 MHz, $CDCl_3$)

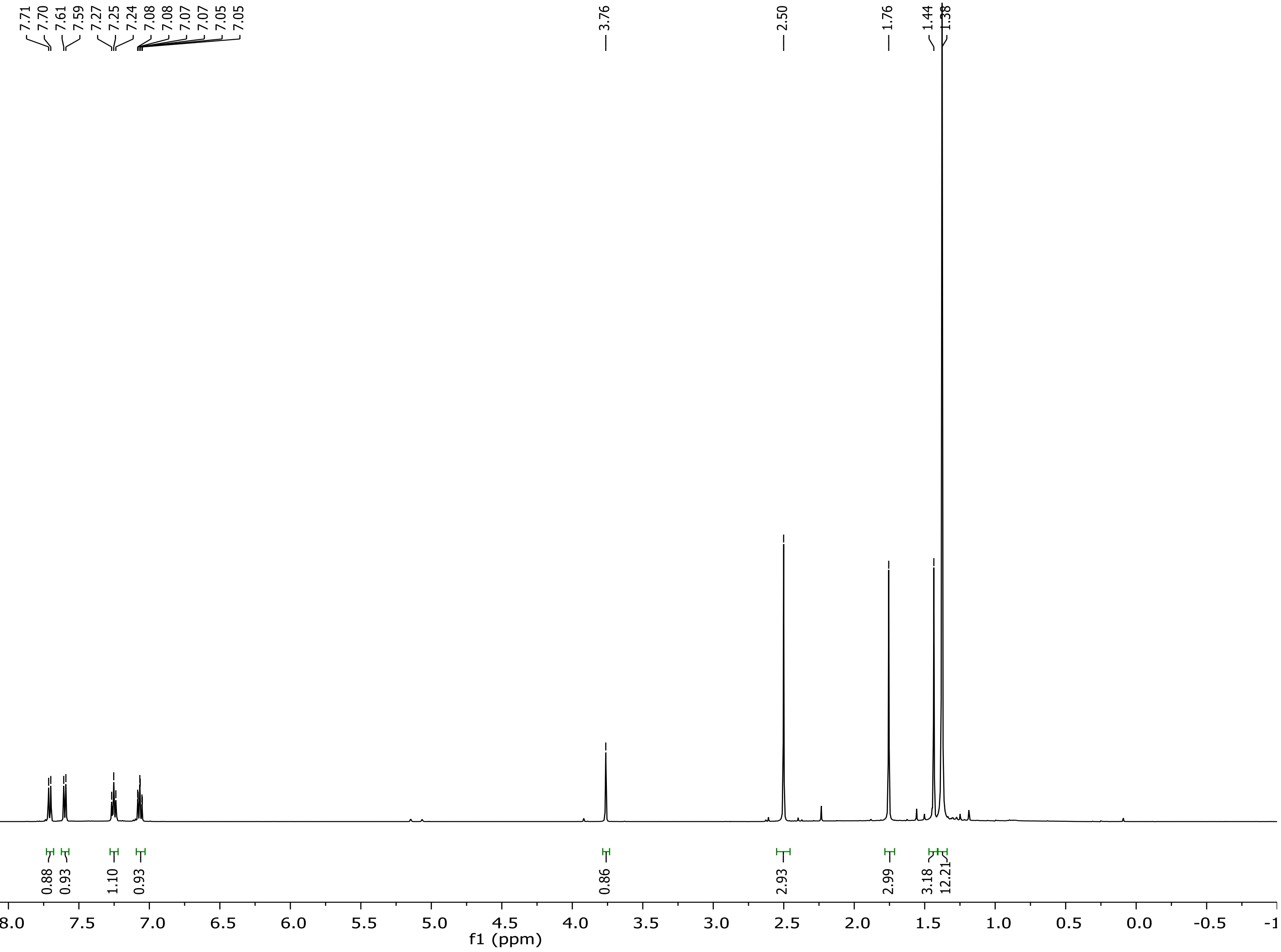

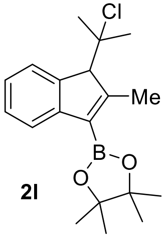

2l

^{13}C-NMR (100 MHz, $CDCl_3$)

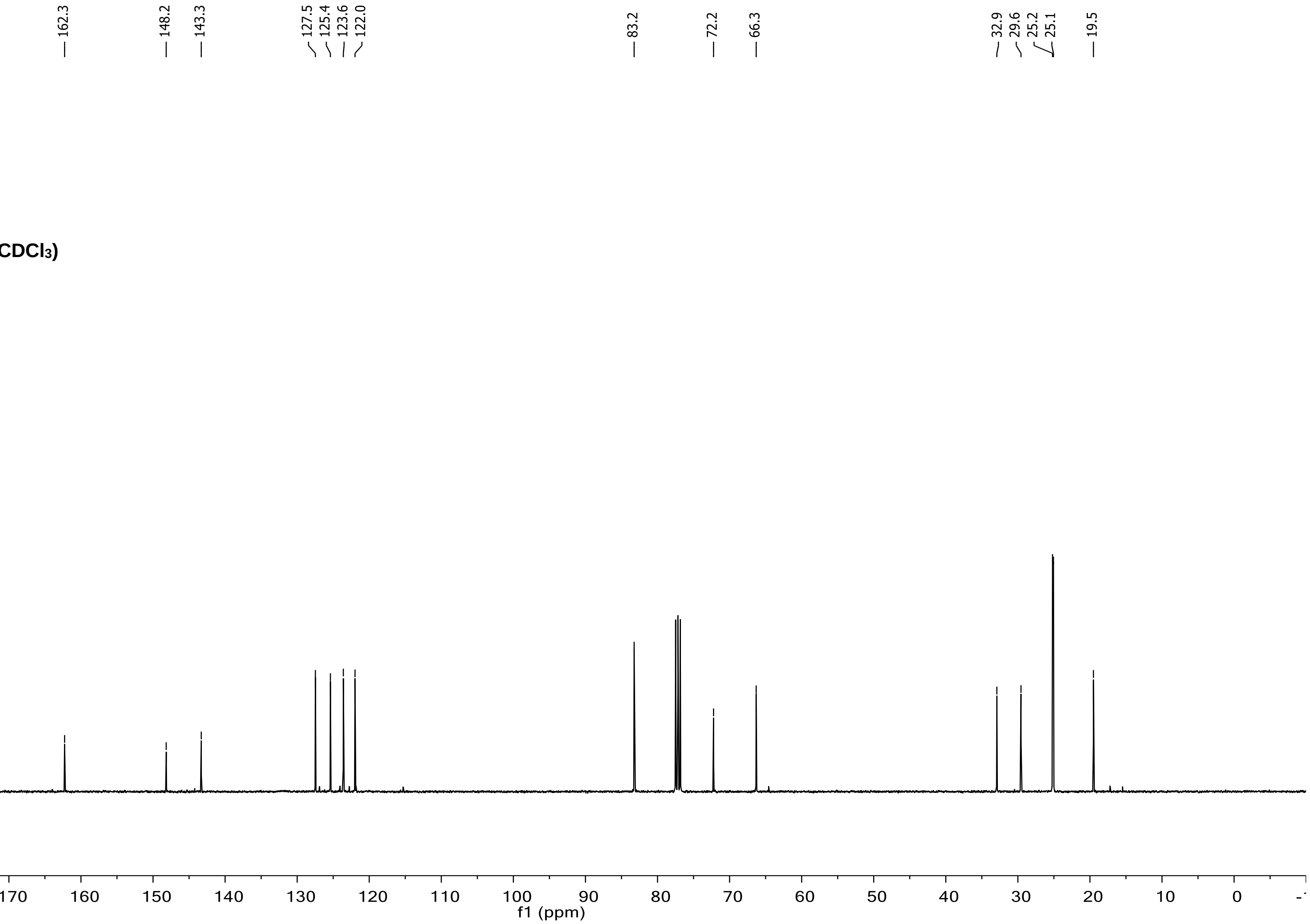

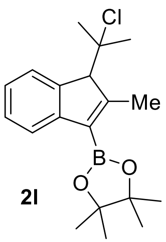

2l

^{11}B-NMR (128 MHz, CDCl$_3$)

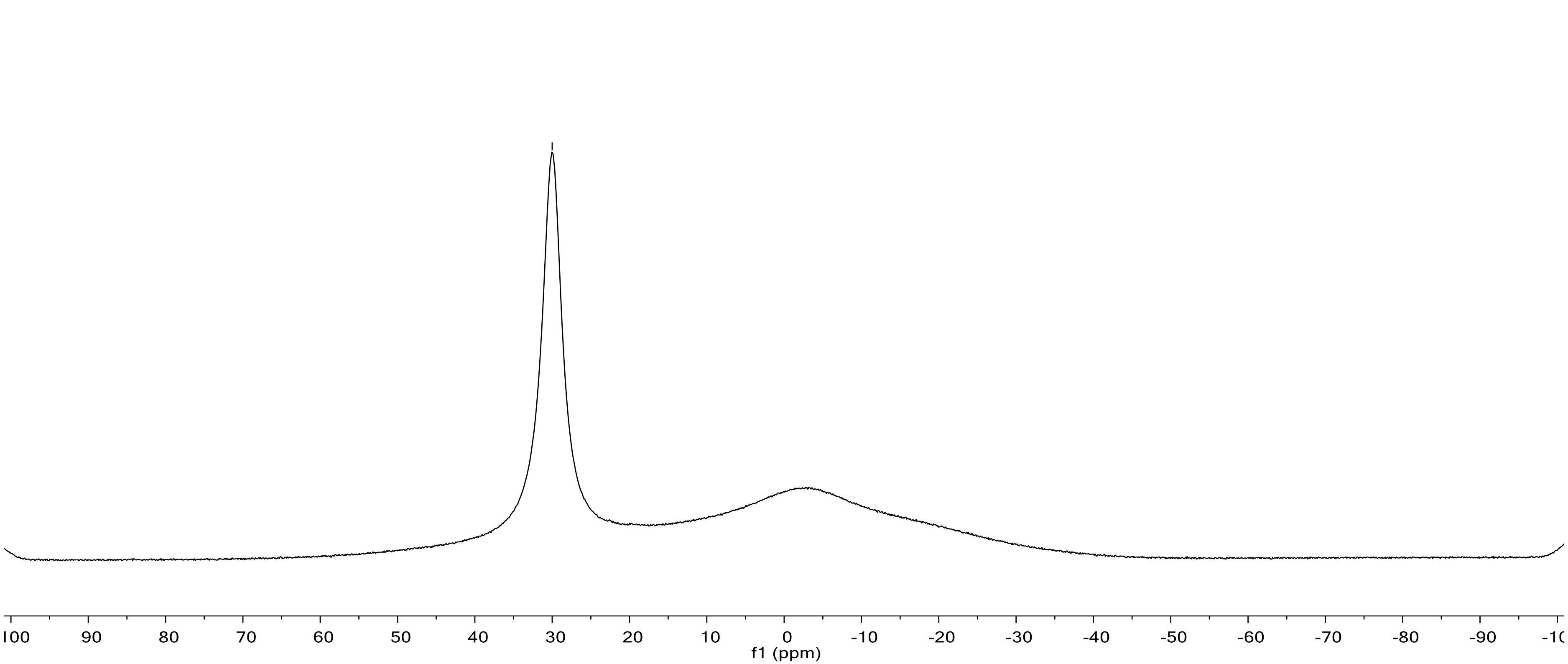

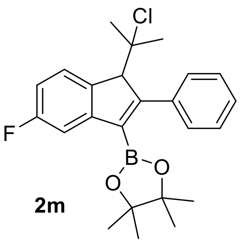

2m

^{1}H-NMR (300 MHz, $CDCl_3$)

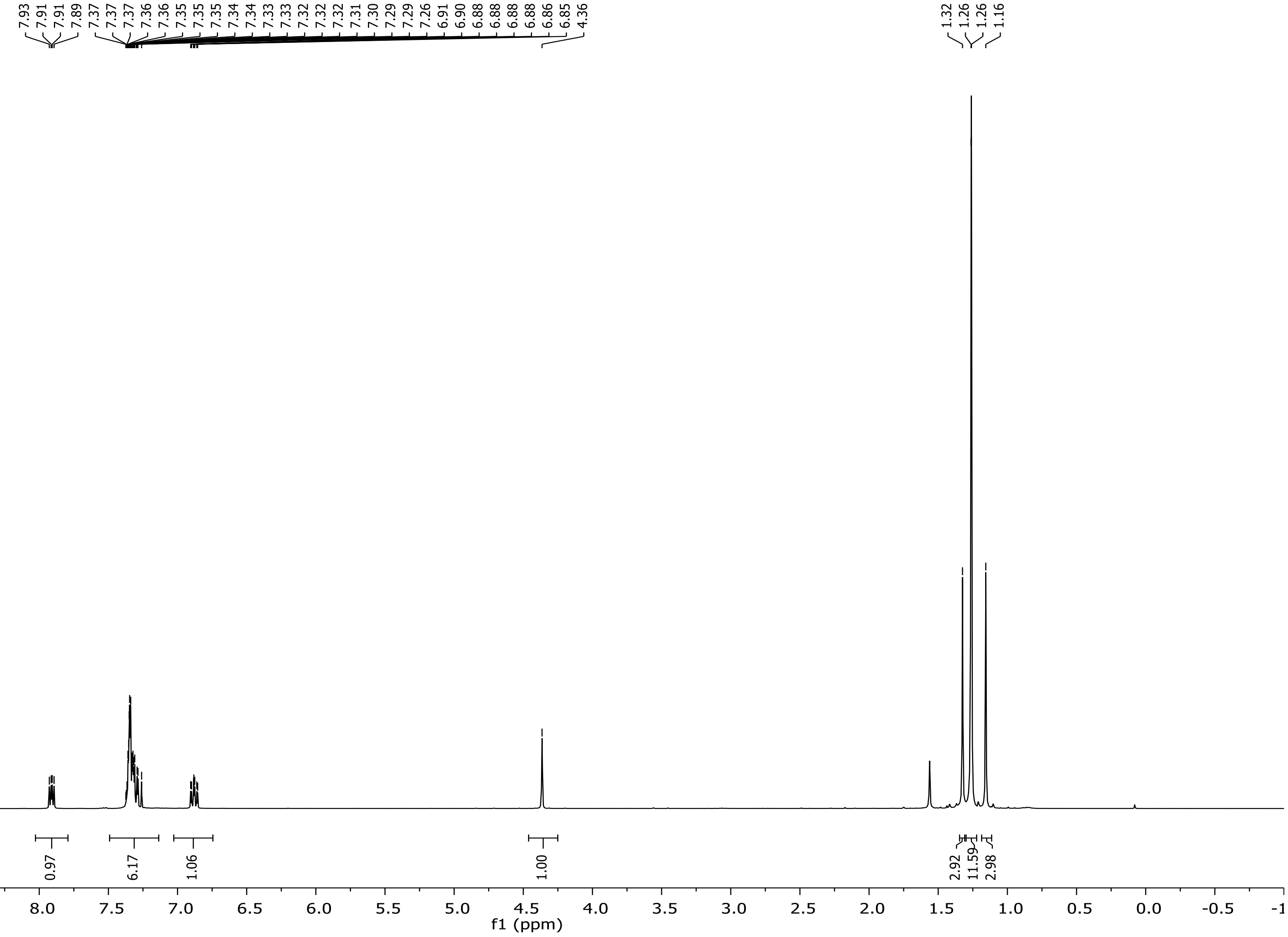

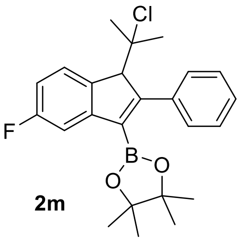

2m

^{13}C-NMR (100 MHz, $CDCl_3$)

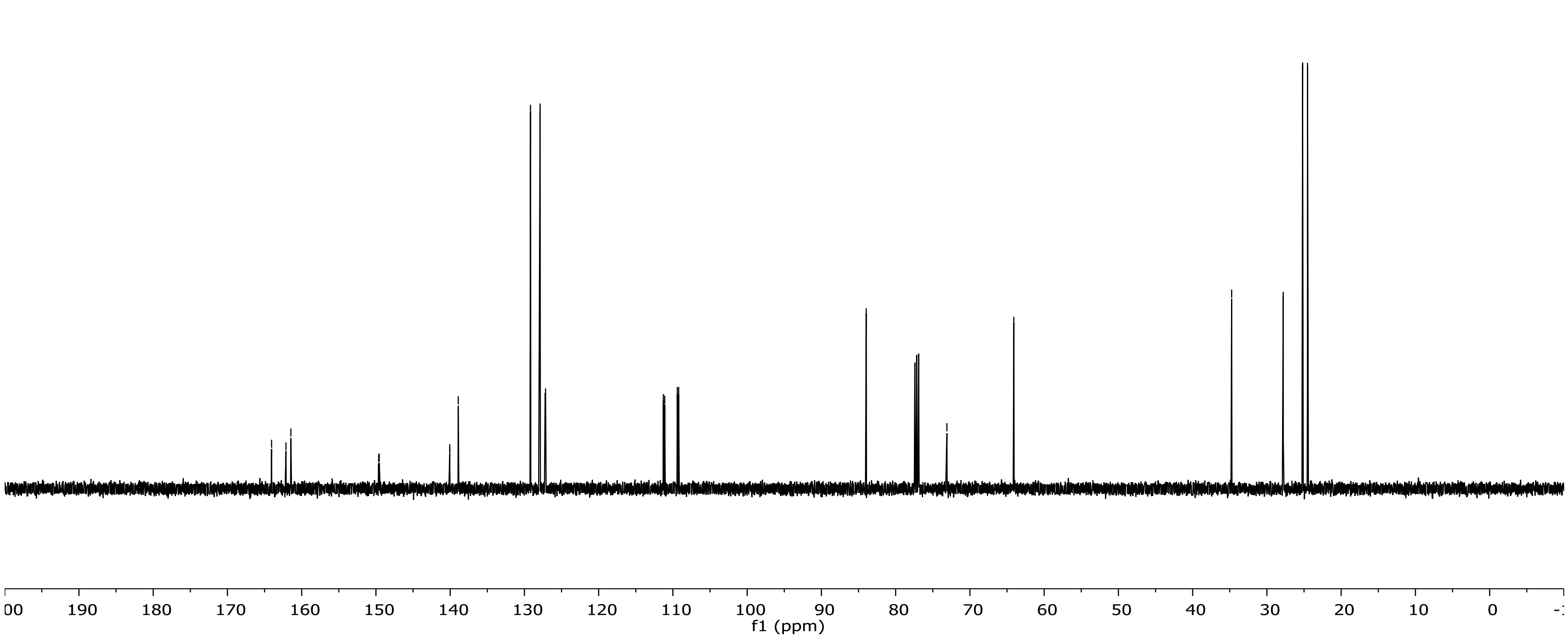

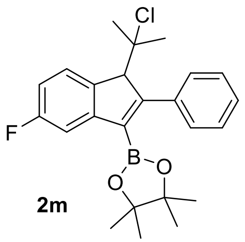

2m

^{19}F-NMR (376 MHz, $CDCl_3$)

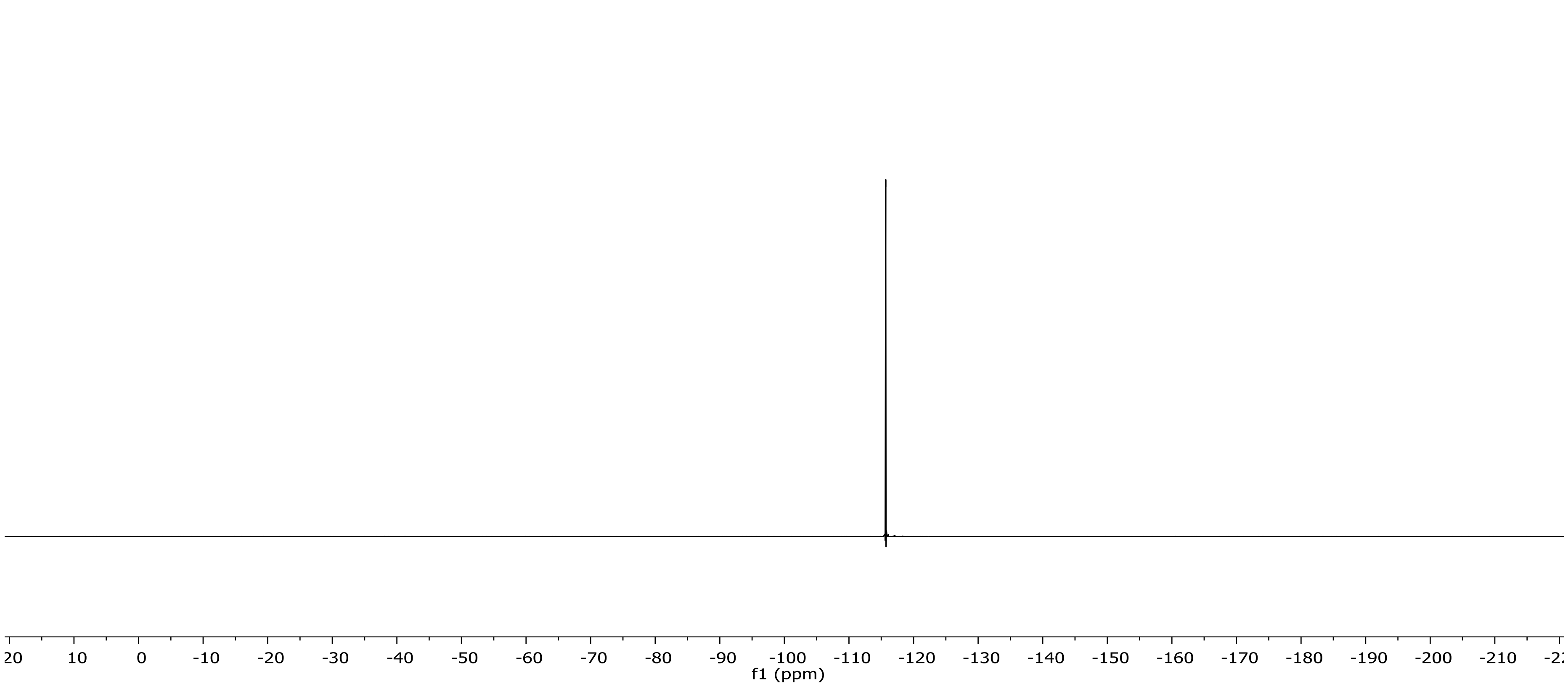

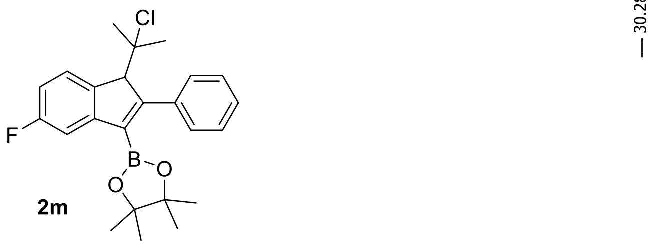

2m

^{11}B-NMR (128 MHz, CDCl$_3$)

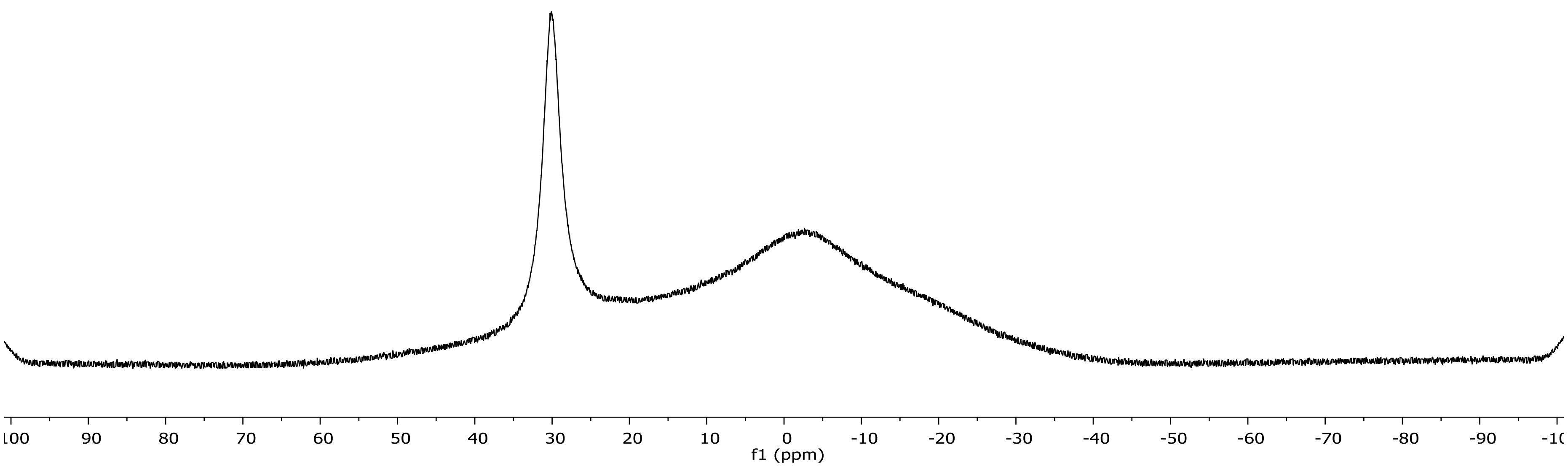

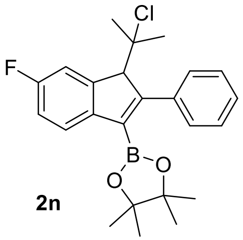

2n

^{1}H-NMR (300 MHz, $CDCl_3$)

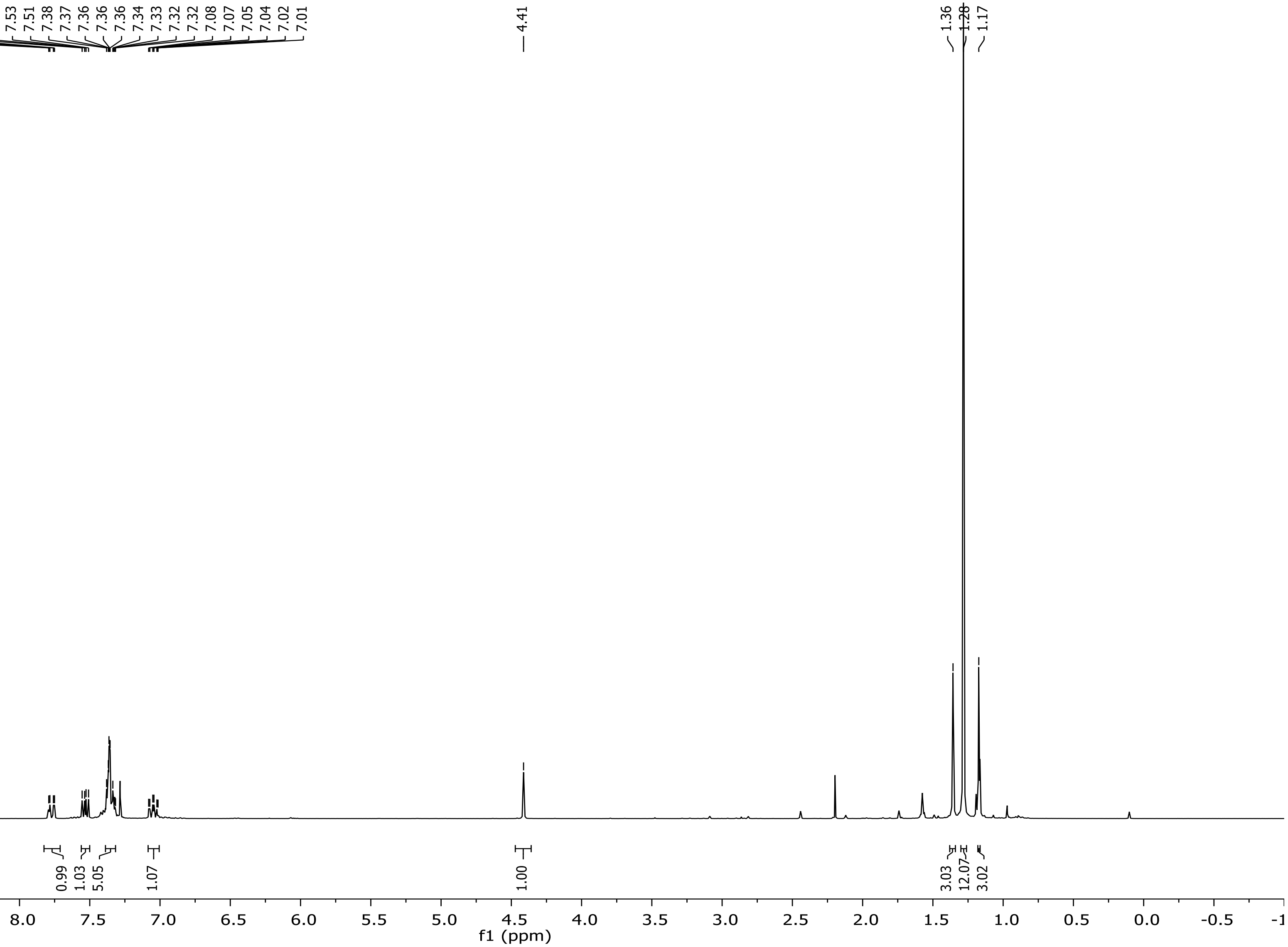

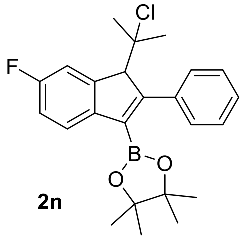

2n

^{13}C-NMR (75 MHz, CDCl$_3$)

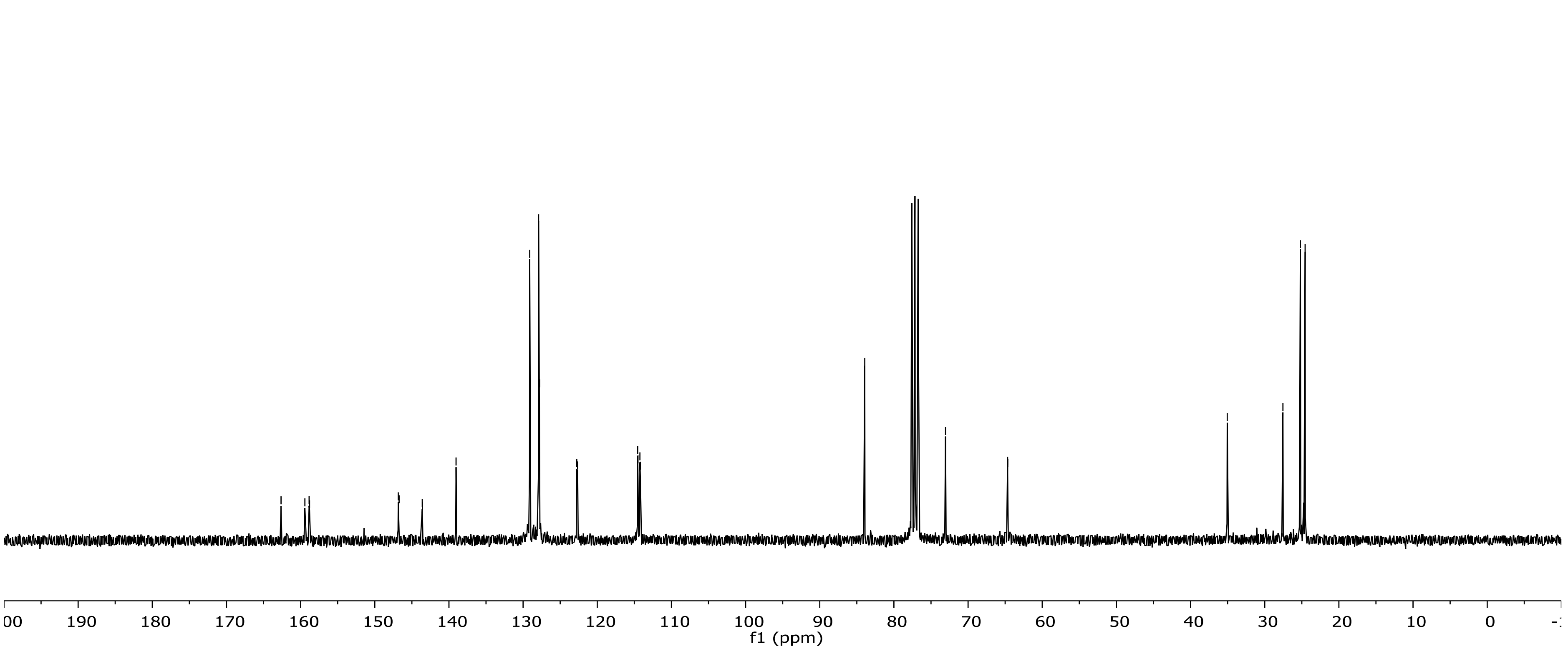

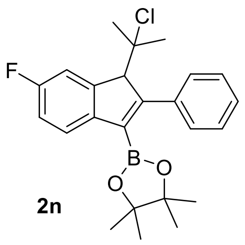

2n

^{19}F-NMR (471 MHz, CDCl$_3$)

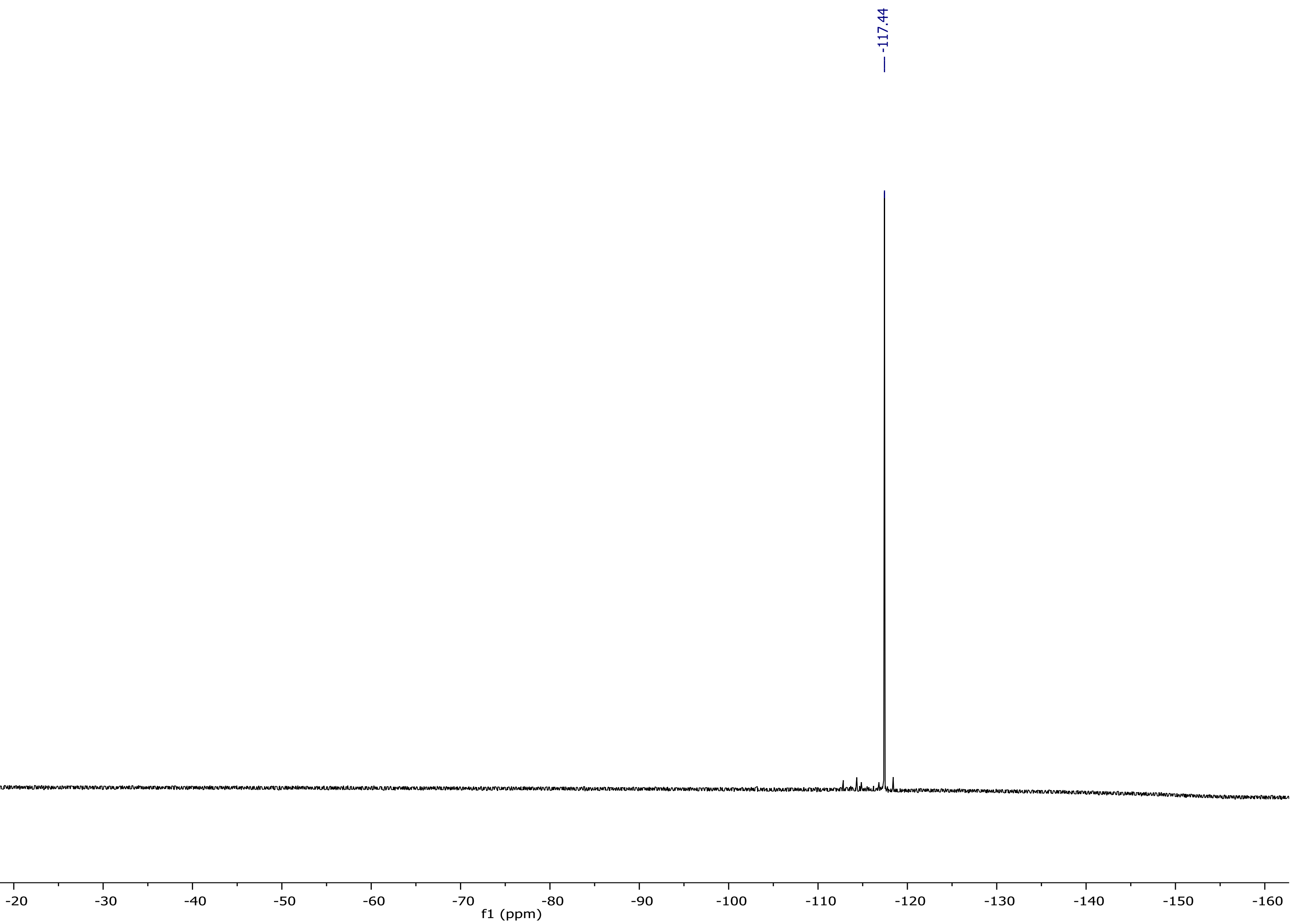

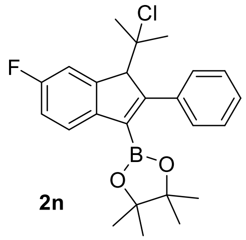

2n

^{11}B-NMR (96 MHz, CDCl₃)

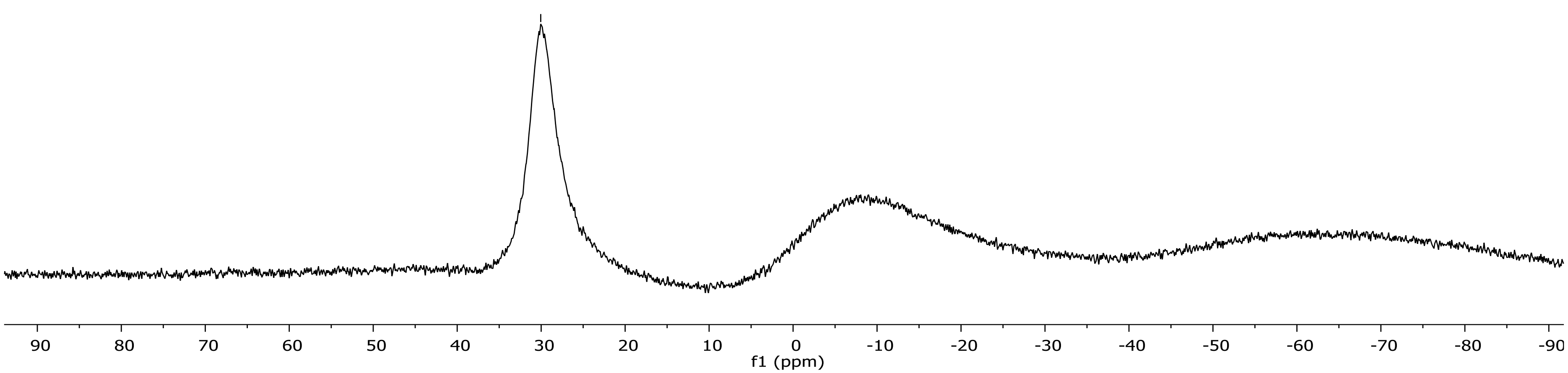

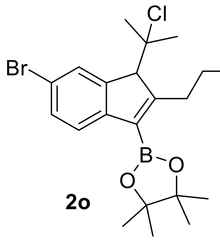

2o

^{1}H-NMR (300 MHz, CDCl₃)

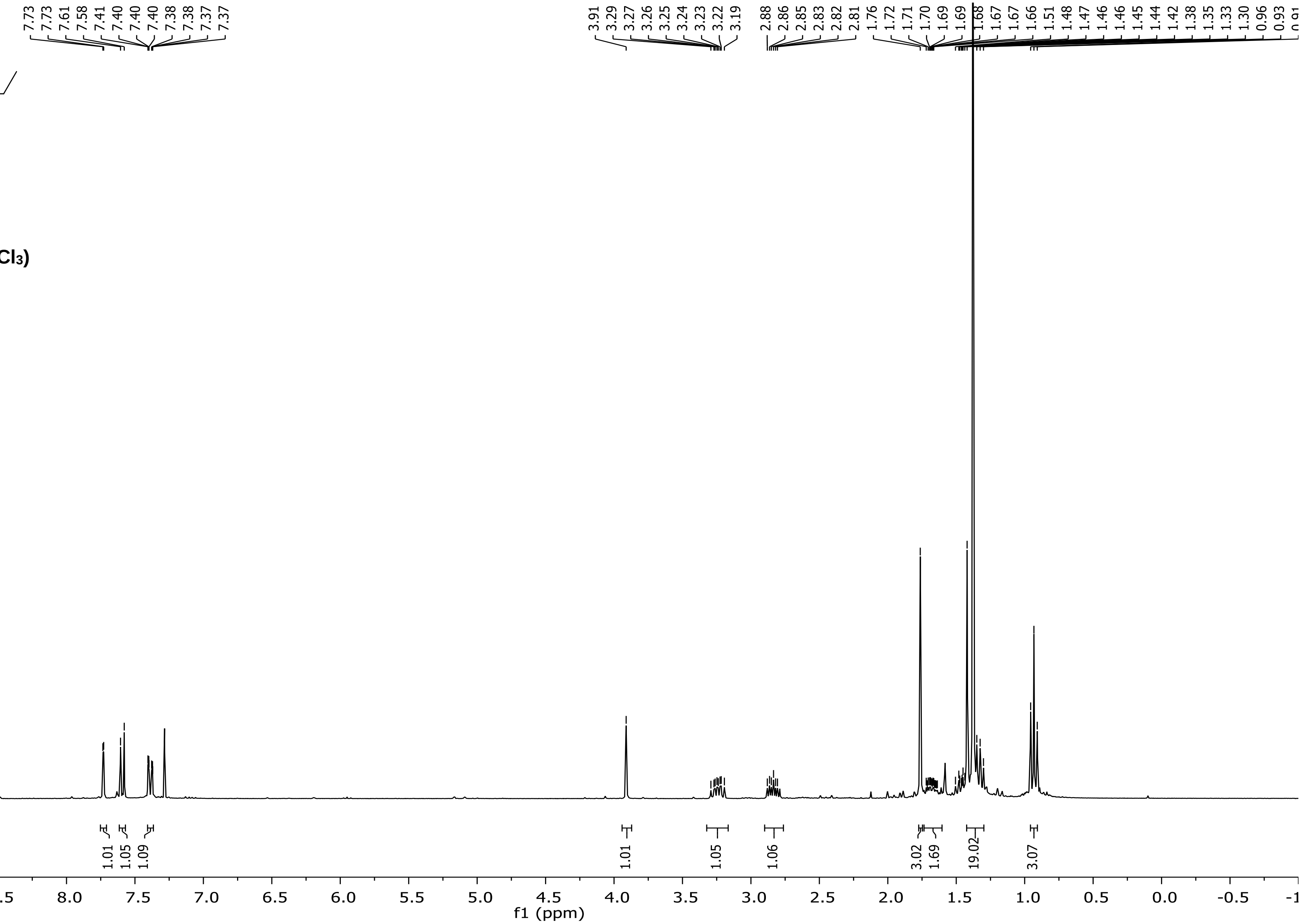

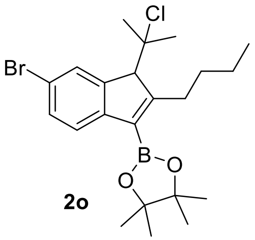

2o

^{13}C-NMR (75 MHz, CDCl$_3$)

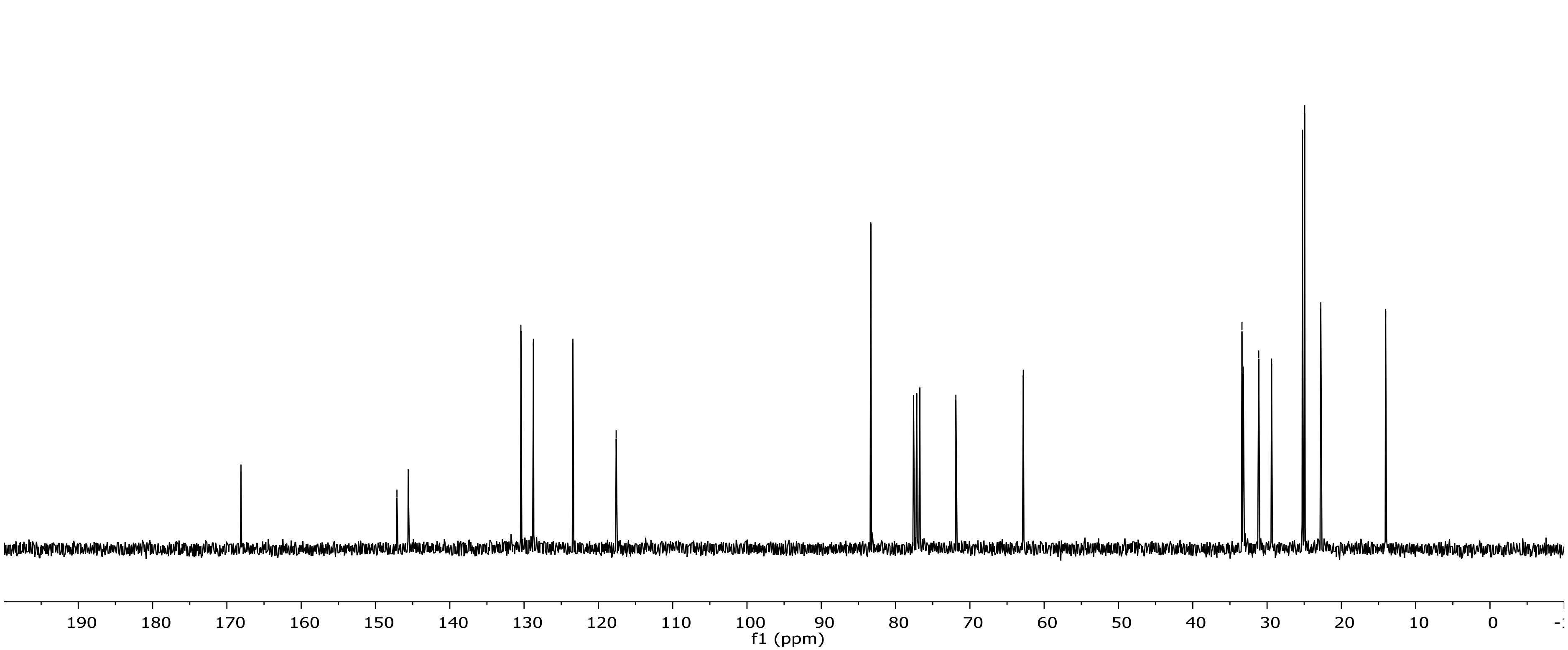

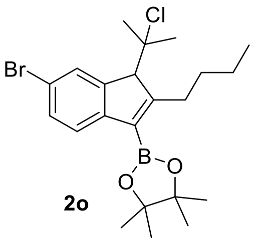

2o

^{11}B-NMR (96 MHz, CDCl$_3$)

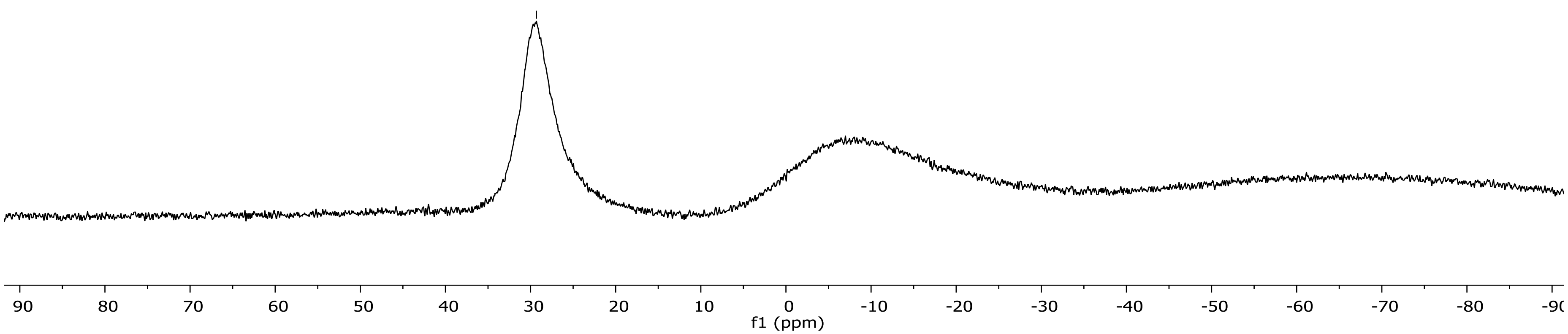

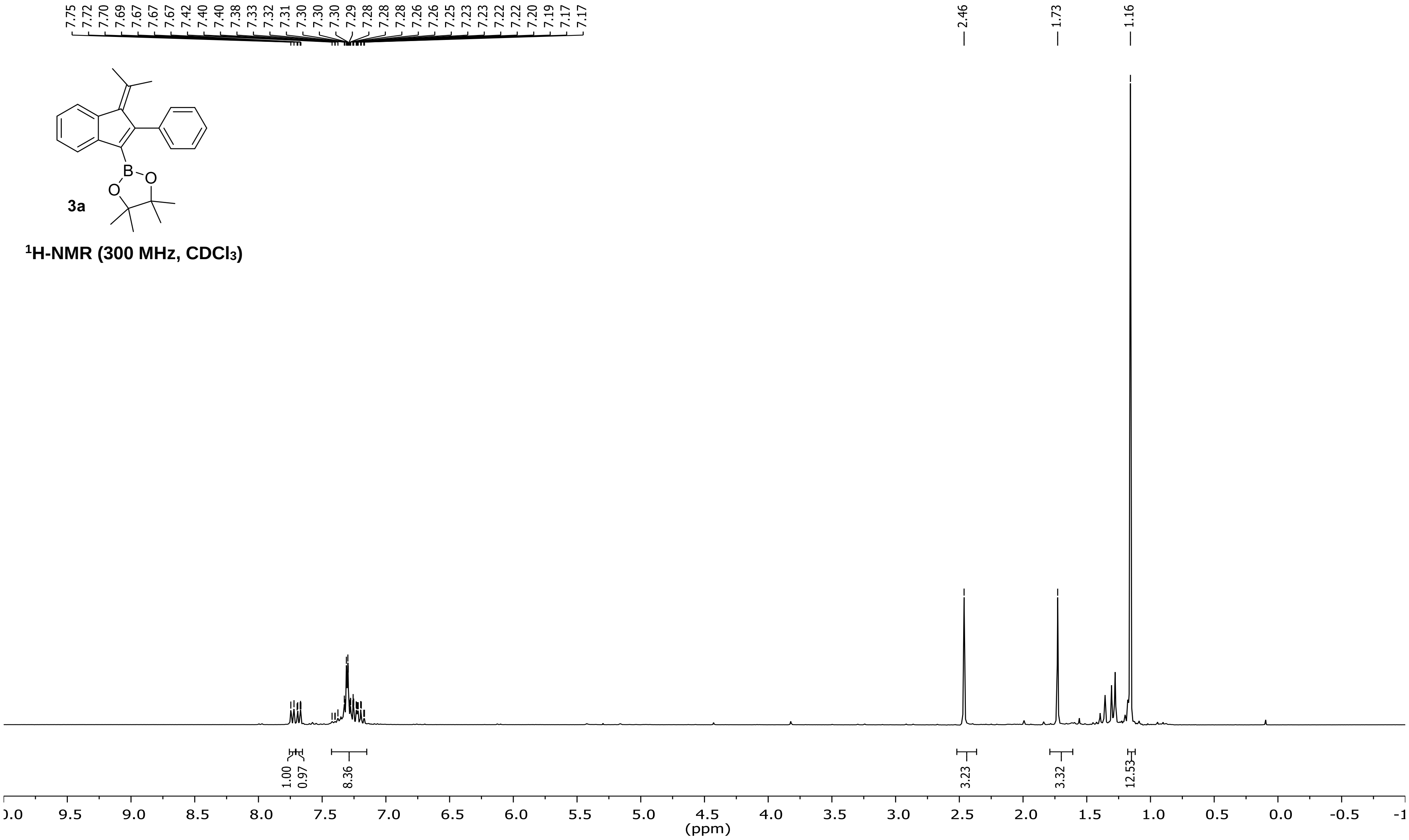
7.75
7.72
7.70
7.69
7.67
7.67
7.67
7.42
7.40
7.40
7.38
7.33
7.32
7.31
7.30
7.30
7.30
7.29
7.28
7.28
7.28
7.26
7.25
7.23
7.23
7.22
7.22
7.20
7.19
7.17
7.17
2.46
1.73
1.16
B
O
O
3a
¹H-NMR (300 MHz, CDCl₃)
1.00
0.97
8.36
3.23
3.32
12.53
9.5
9.0
8.5
8.0
7.5
7.0
6.5
6.0
5.5
5.0
4.5
4.0
3.5
3.0
2.5
2.0
1.5
1.0
0.5
0.0
-0.5
(ppm)

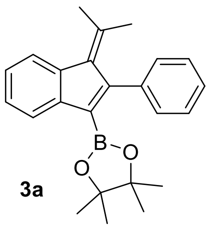

3a

^{13}C-NMR (100 MHz, $CDCl_3$)

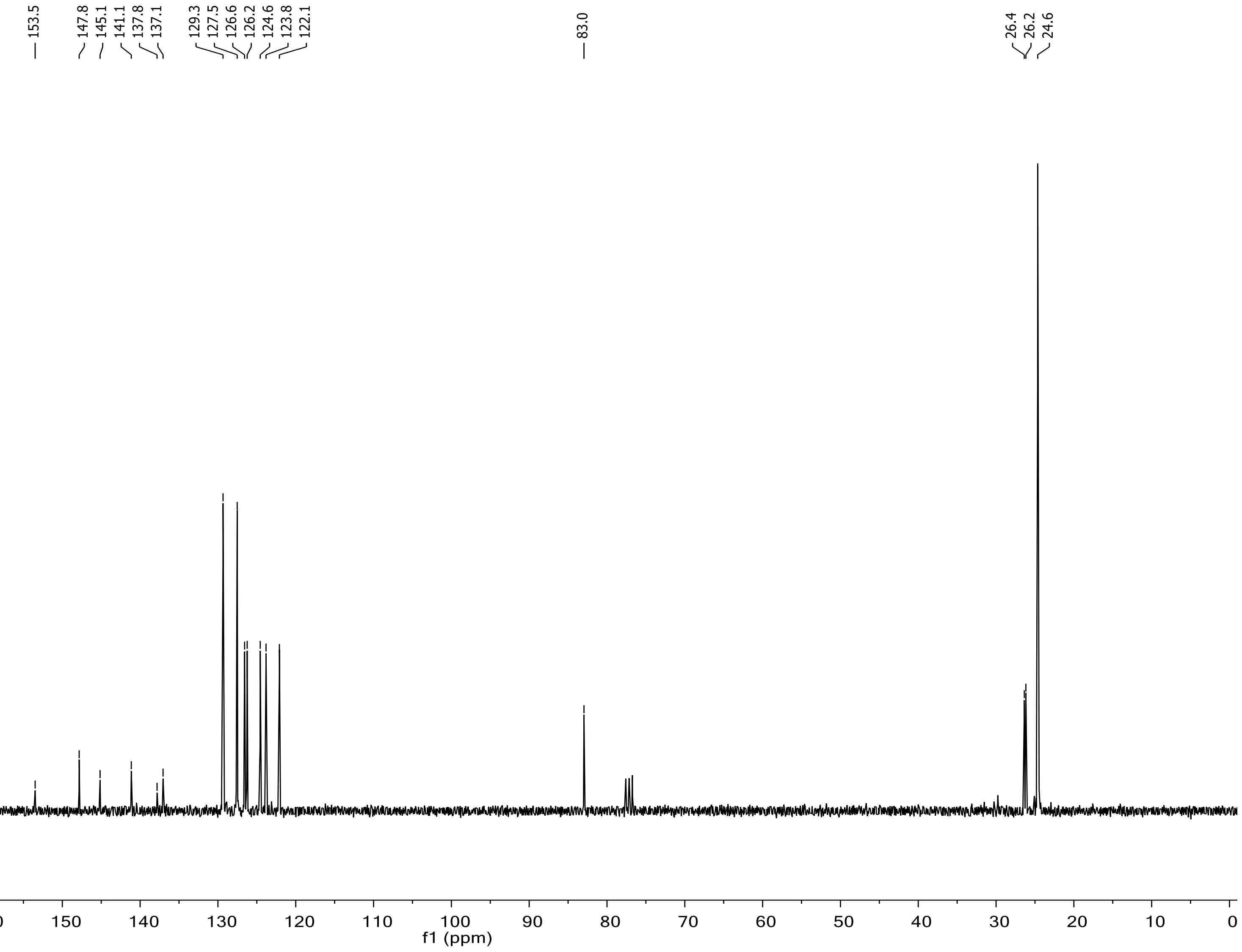

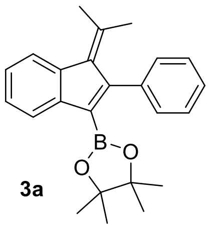

3a

^{11}B-NMR (96 MHz, $CDCl_3$)

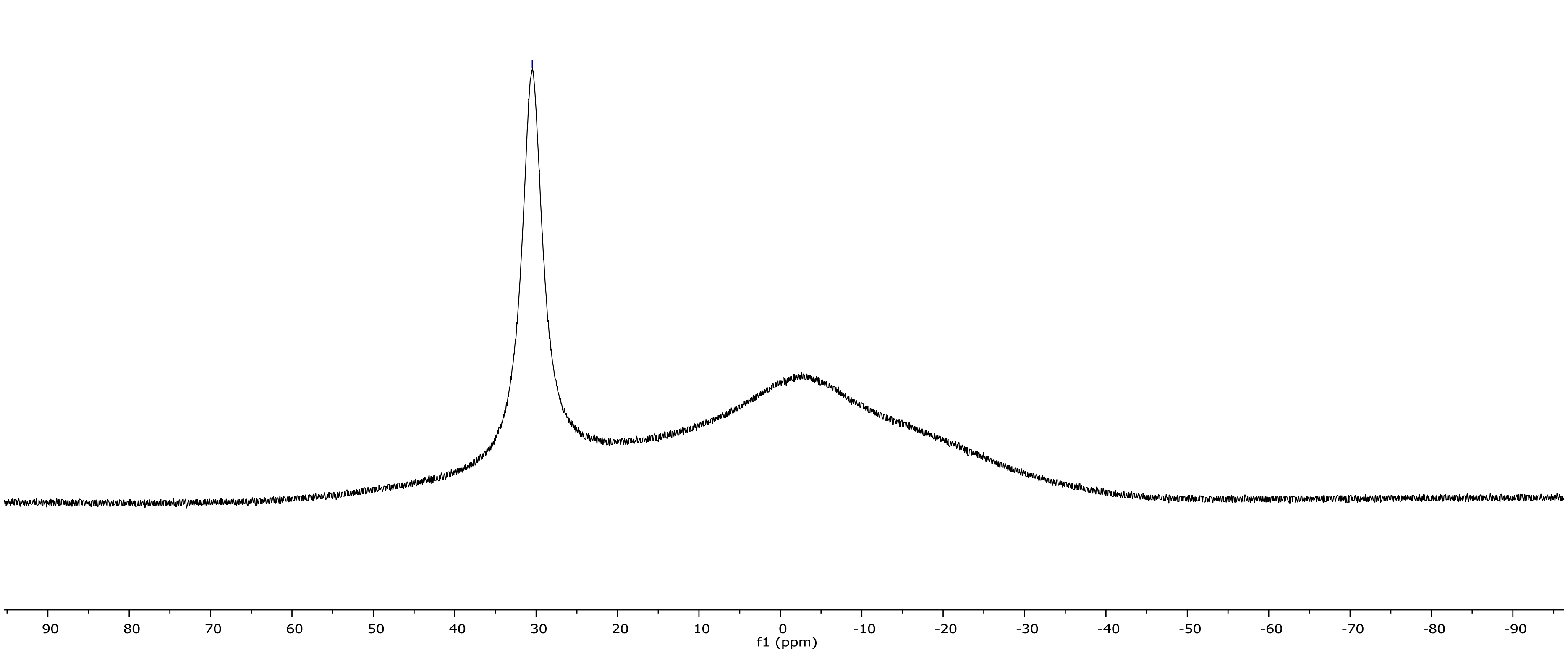

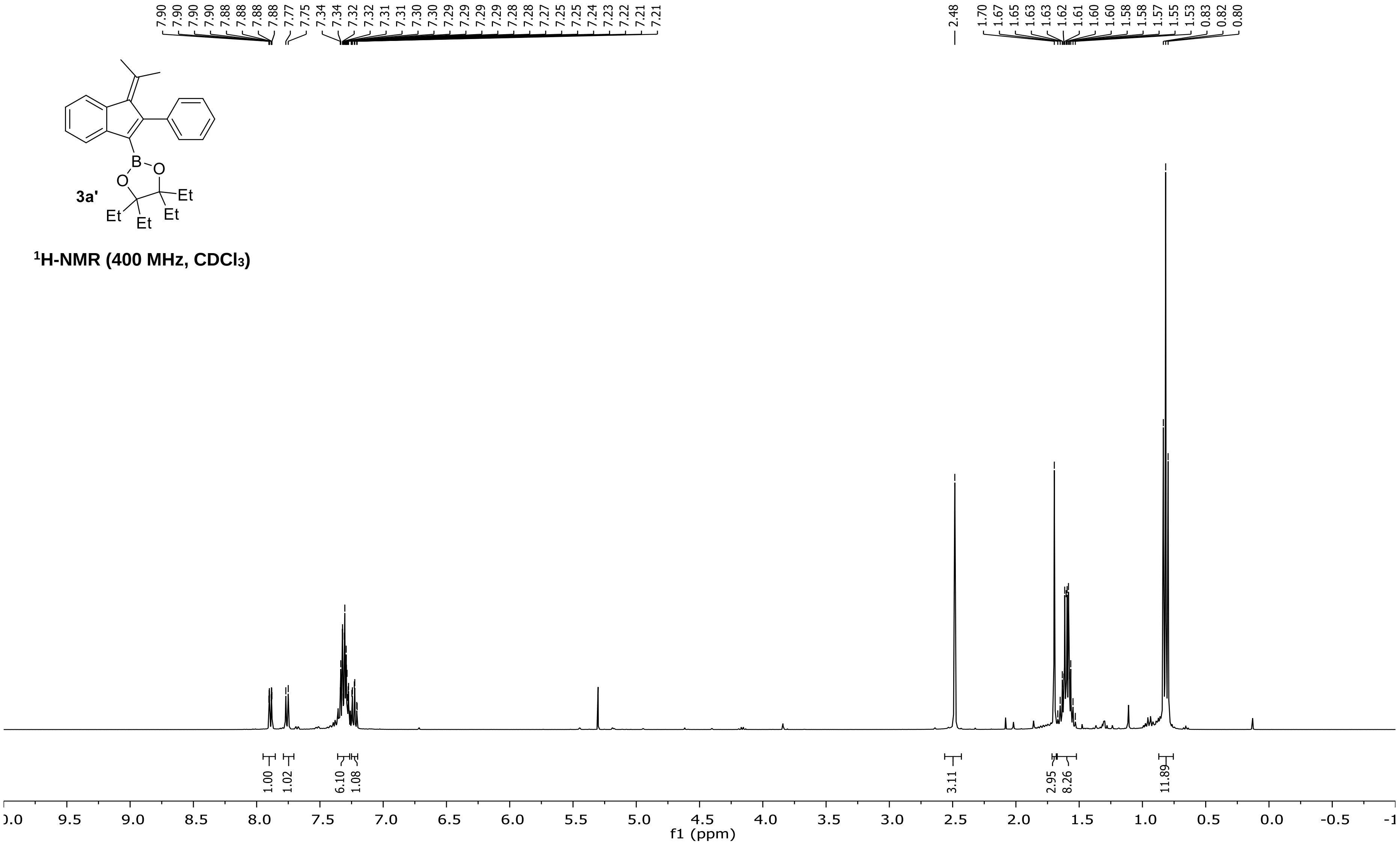

3a'

^{1}H-NMR (400 MHz, CDCl₃)

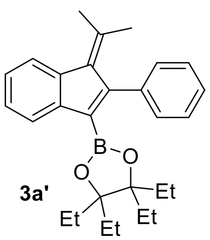

3a'

^{13}C-NMR (100 MHz, $CDCl_3$)

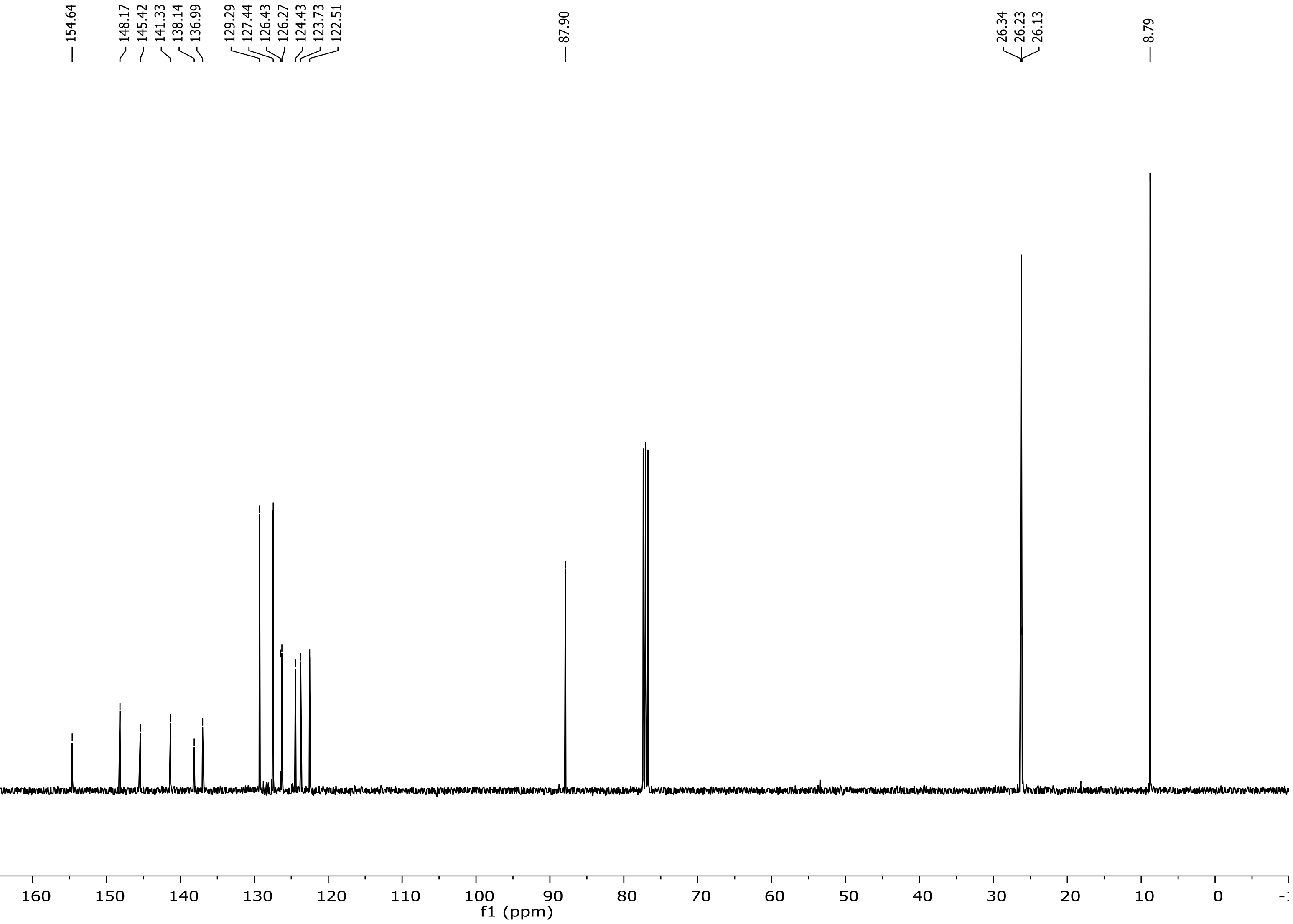

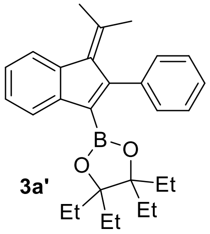

3a'

^{11}B-NMR (128 MHz, CDCl$_3$)

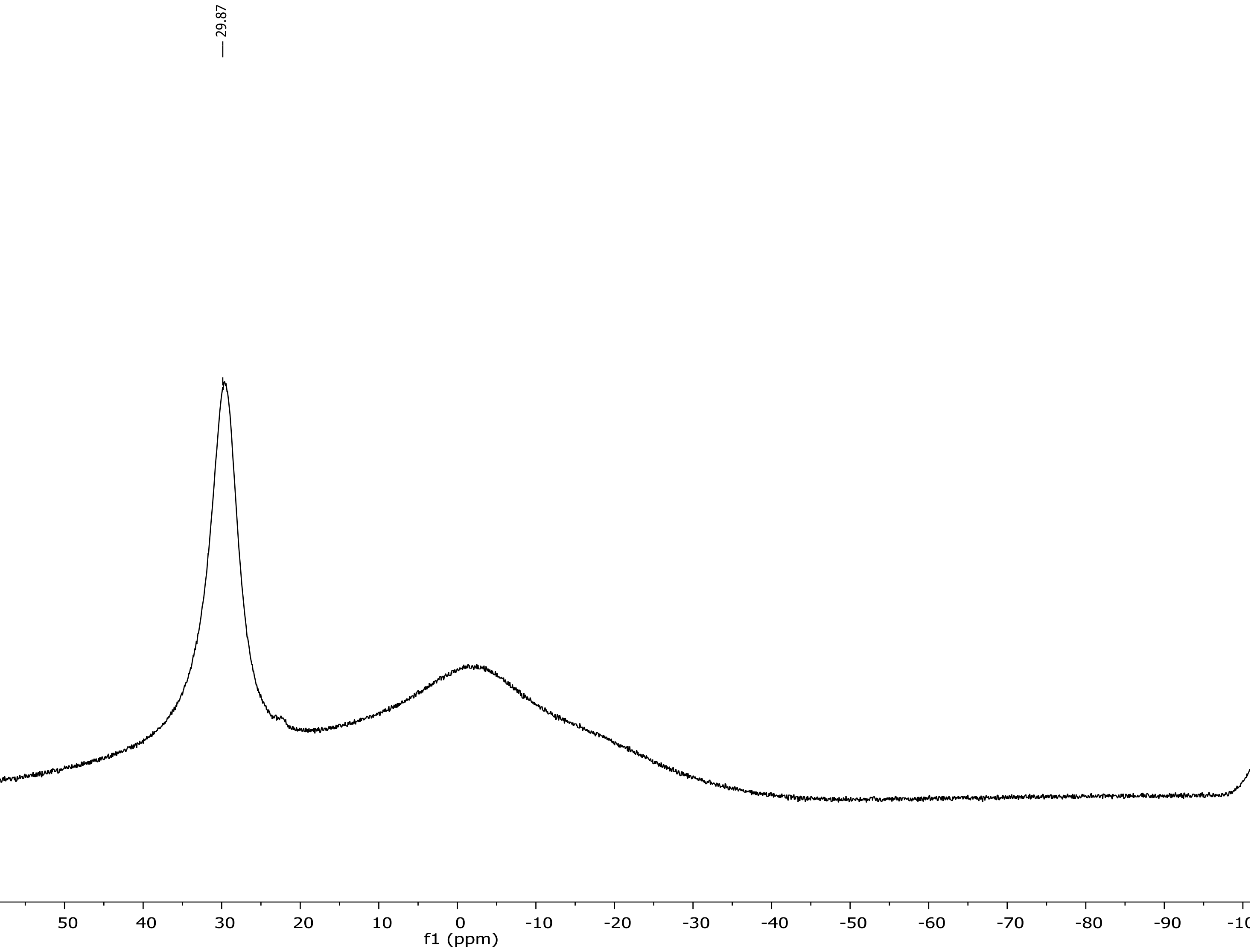

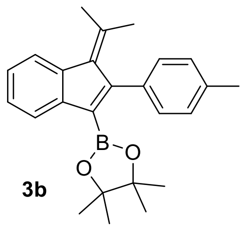

3b

^{1}H-NMR (400 MHz, $CDCl_3$)

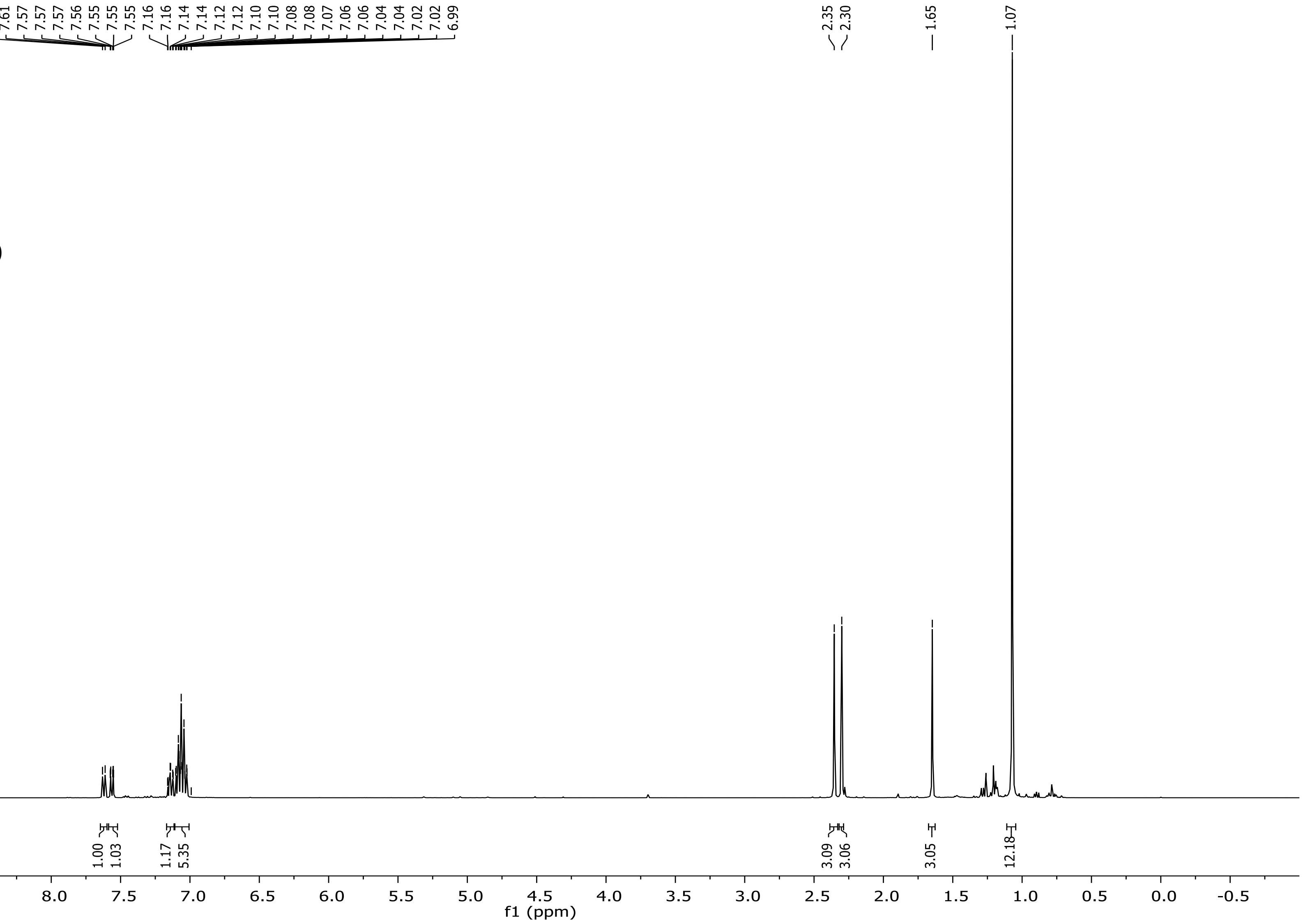

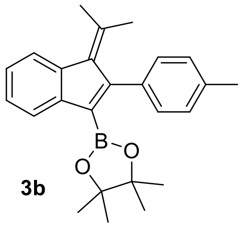

3b

^{13}C-NMR (100 MHz, CDCl$_3$)

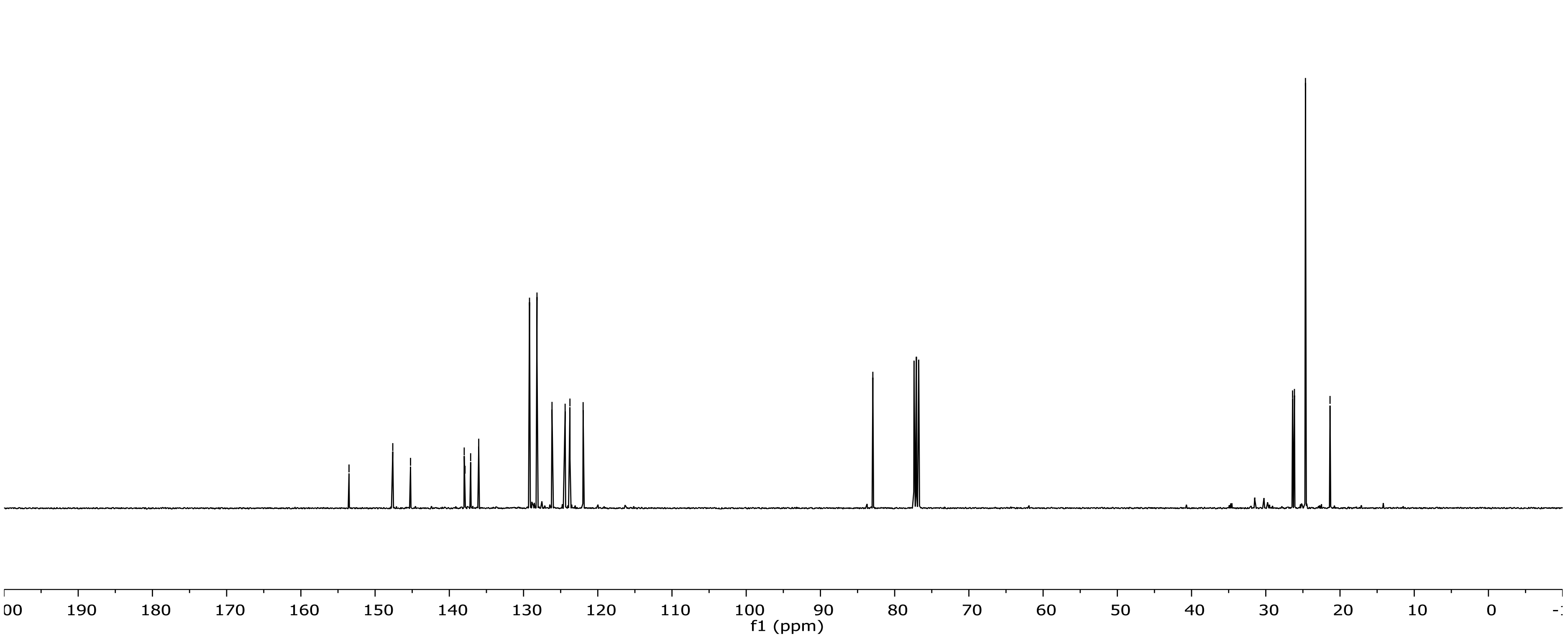

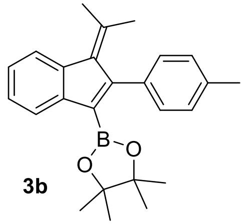

3b

^{11}B-NMR (128 MHz, $CDCl_3$)

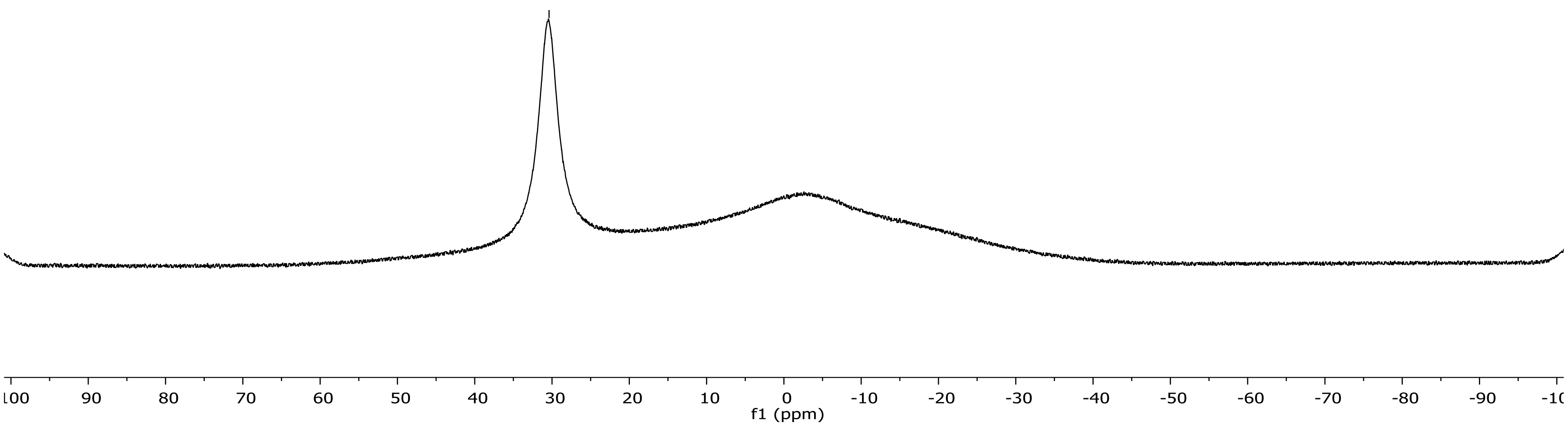

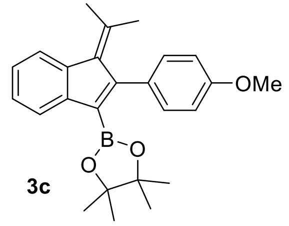

3c

^{1}H-NMR (400 MHz, $CDCl_3$)

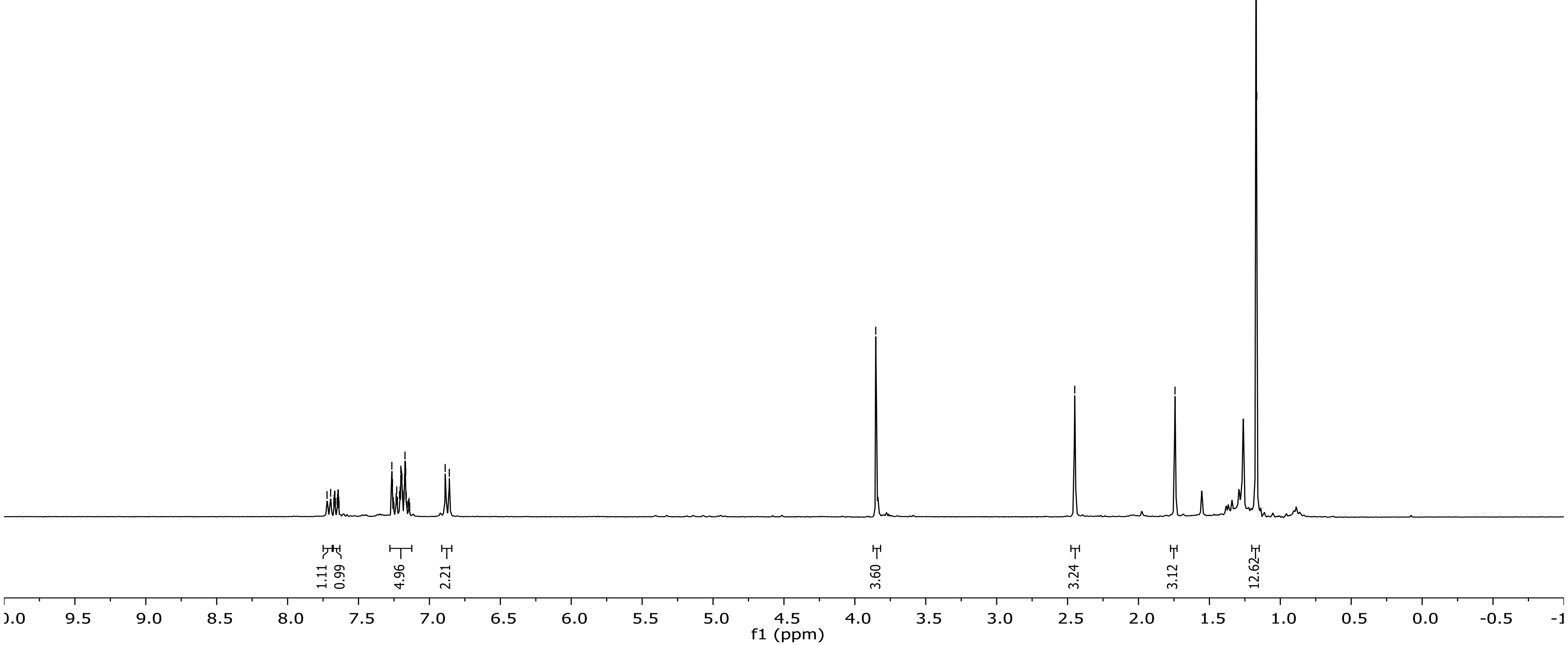

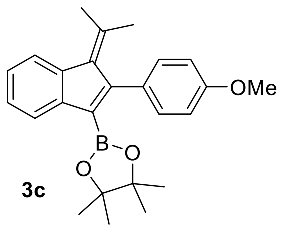

3c

^{13}C-NMR (100 MHz, $CDCl_3$)

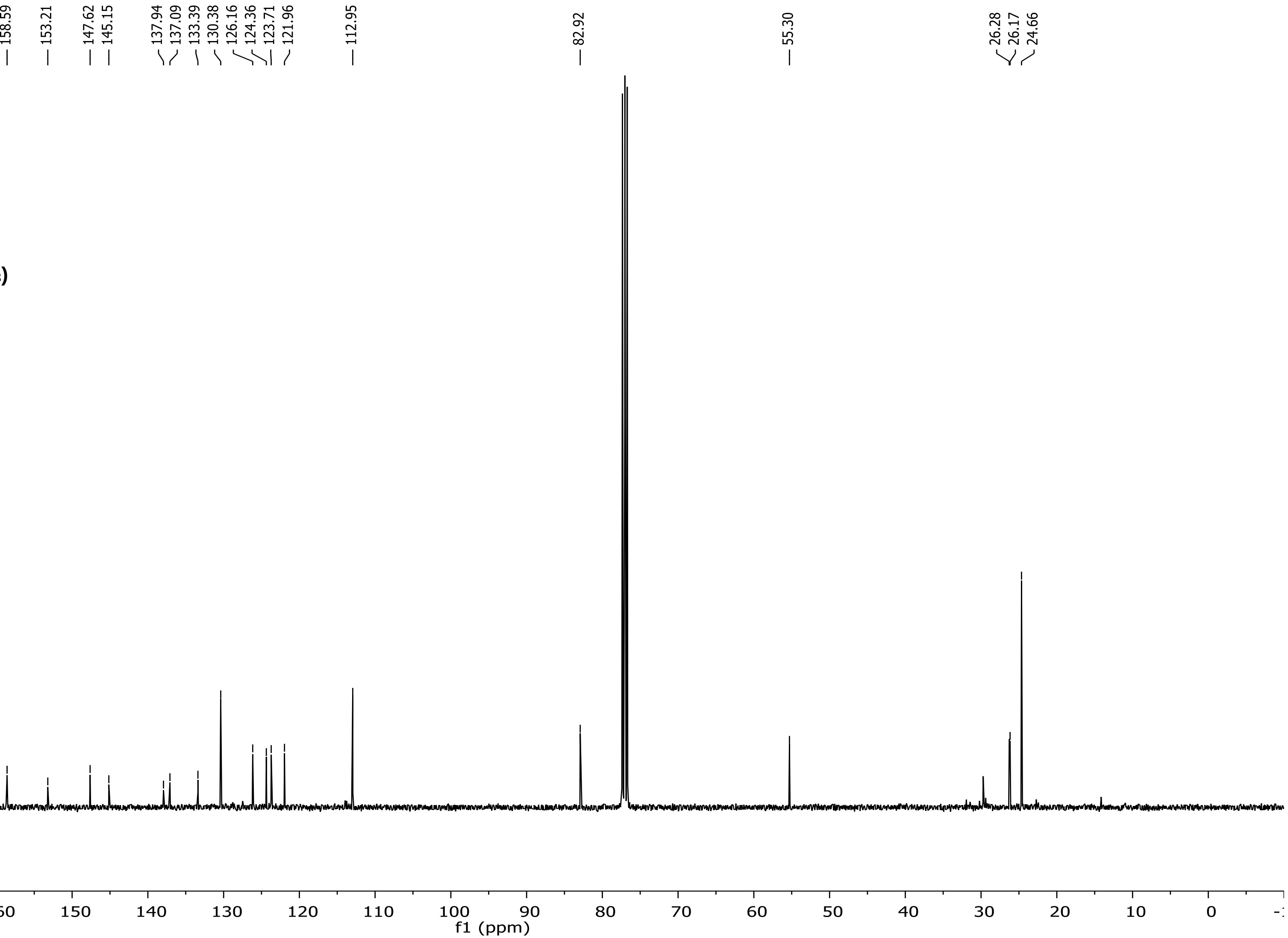

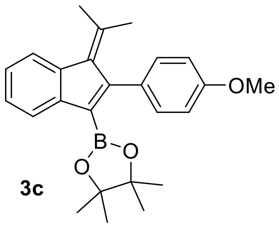

3c

^{11}B-NMR (128 MHz, $CDCl_3$)

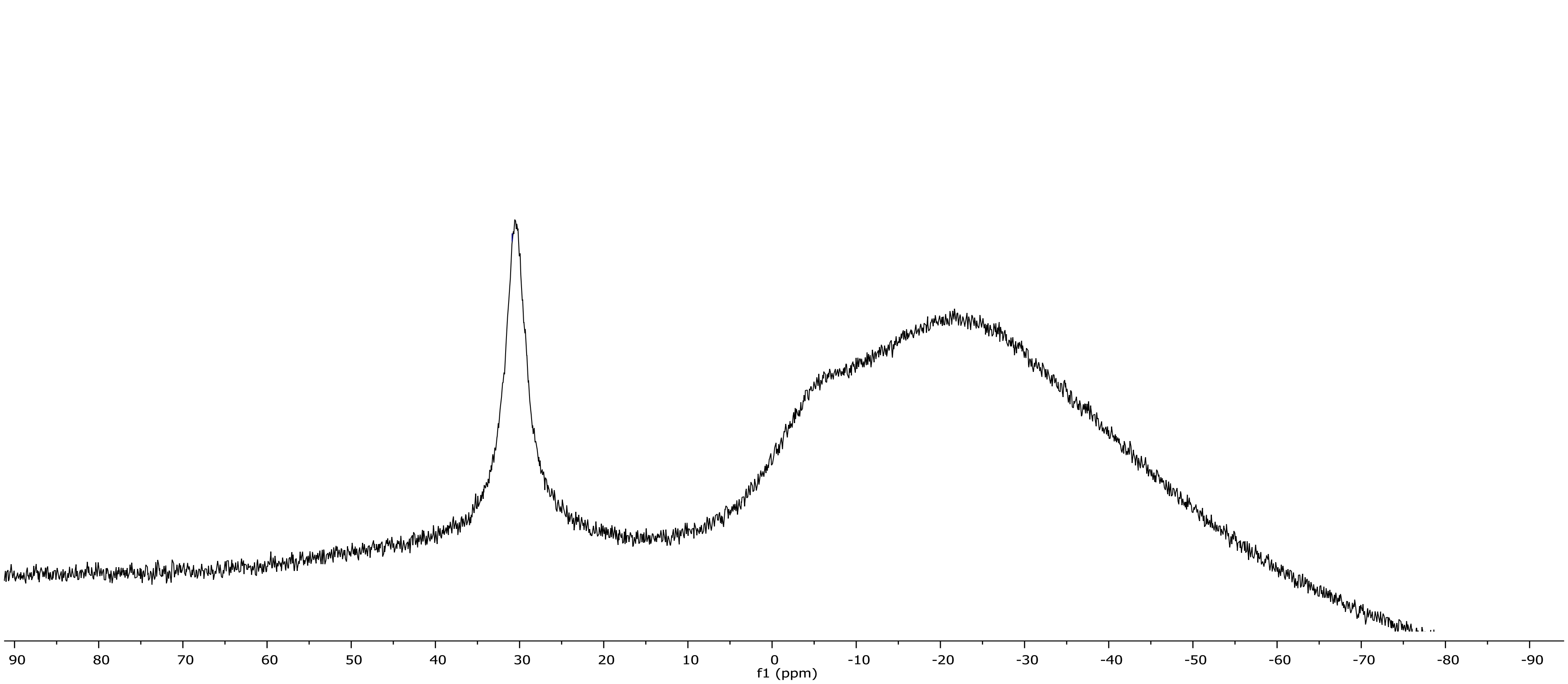

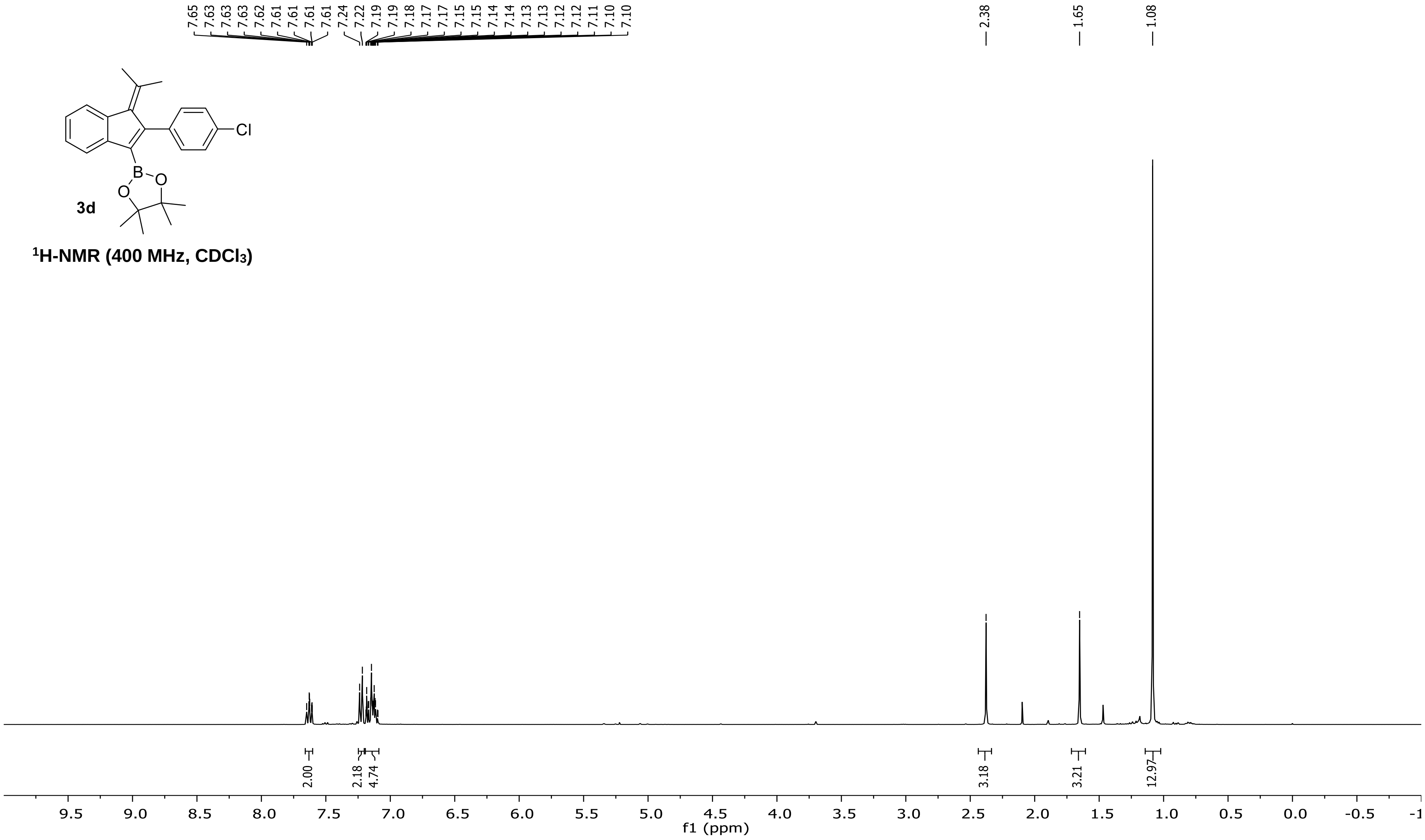

3d

^{1}H-NMR (400 MHz, CDCl$_3$)

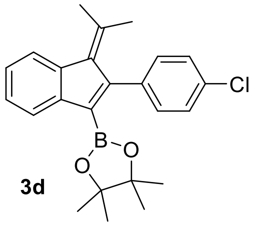

3d

^{13}C-NMR (100 MHz, $CDCl_3$)

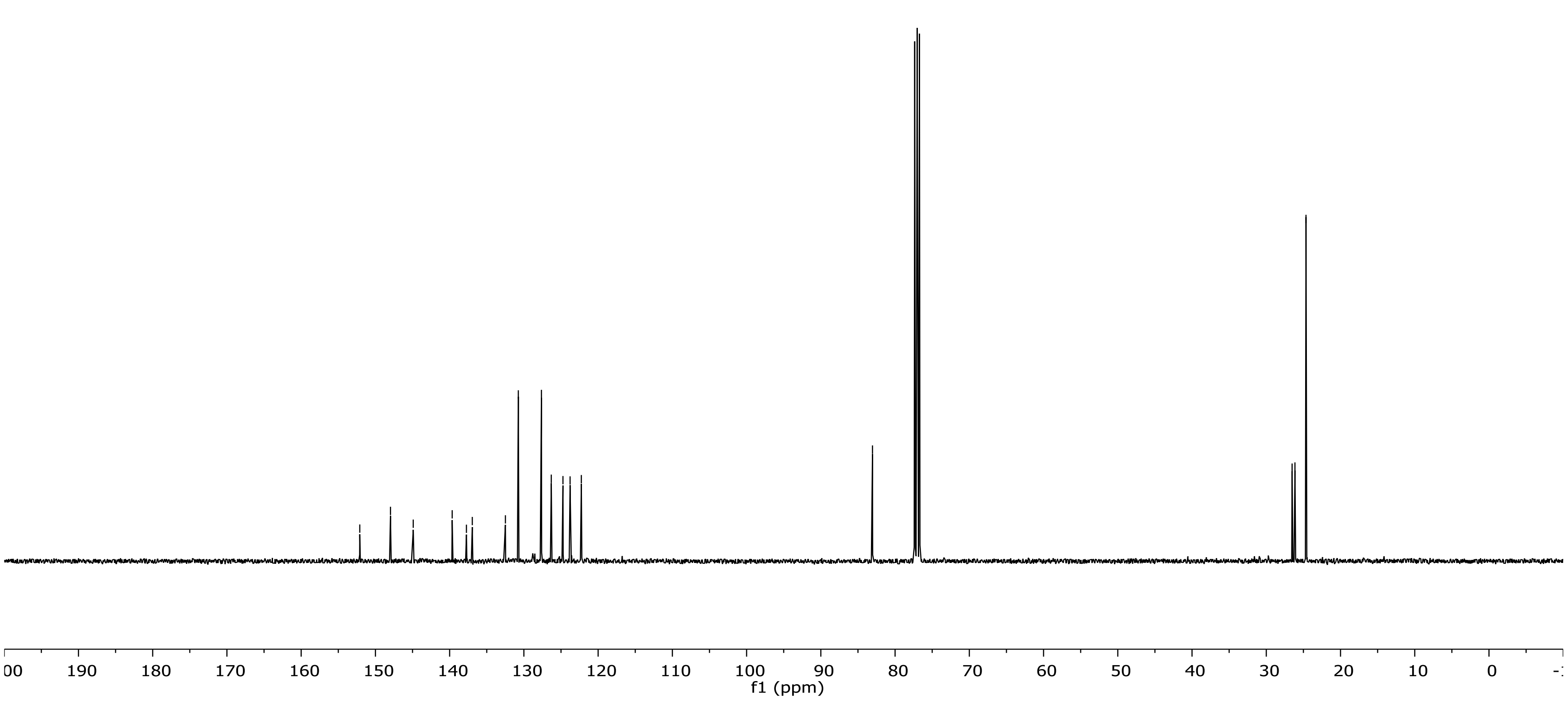

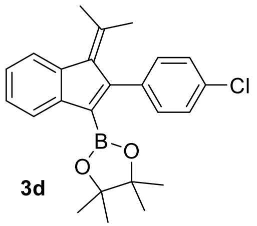

3d

^{11}B-NMR (128 MHz, $CDCl_3$)

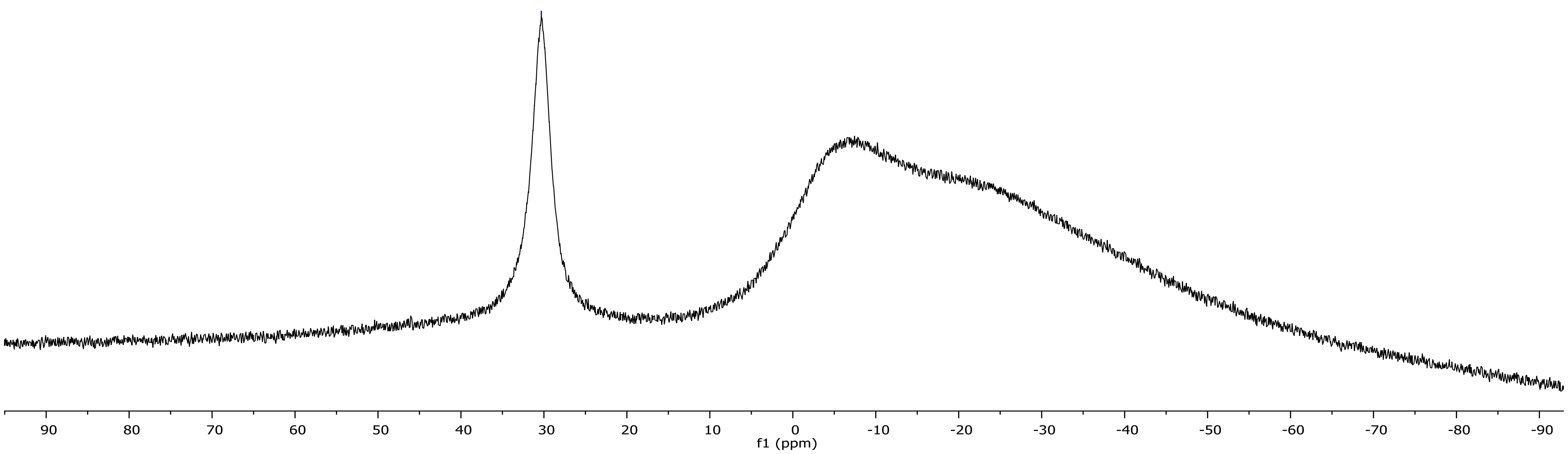

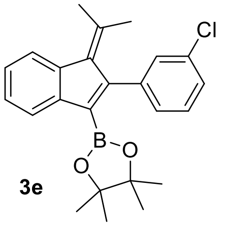

3e

^{1}H-NMR (300 MHz, $CDCl_3$)

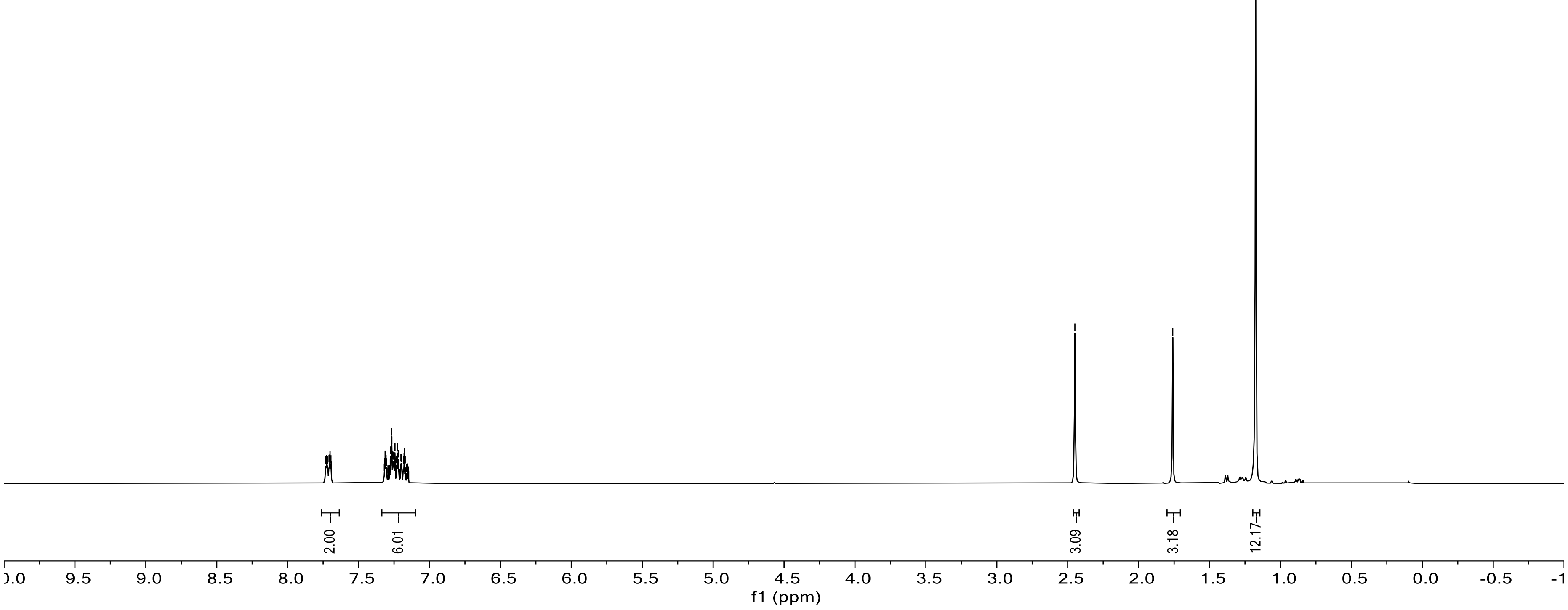

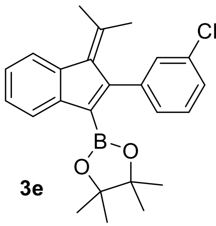

3e

^{13}C-NMR (100 MHz, $CDCl_3$)

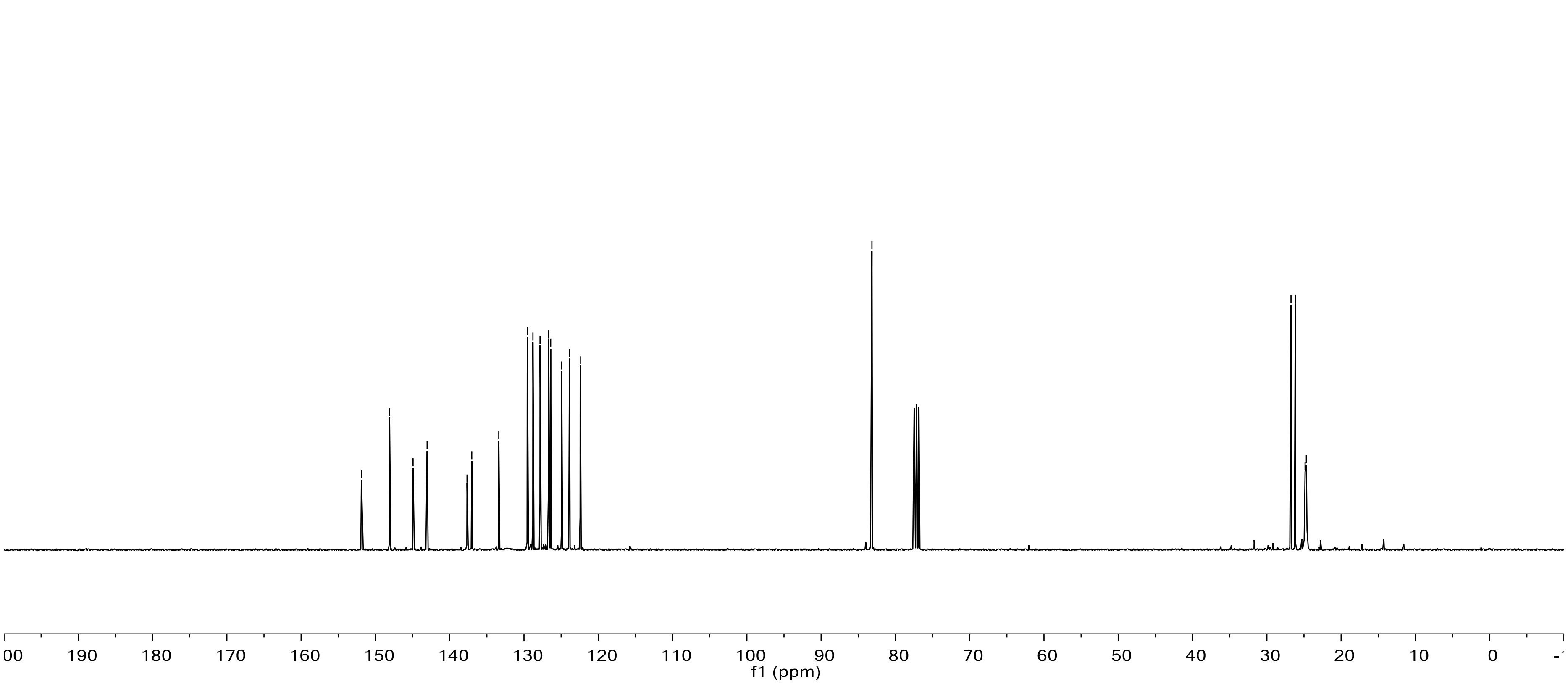

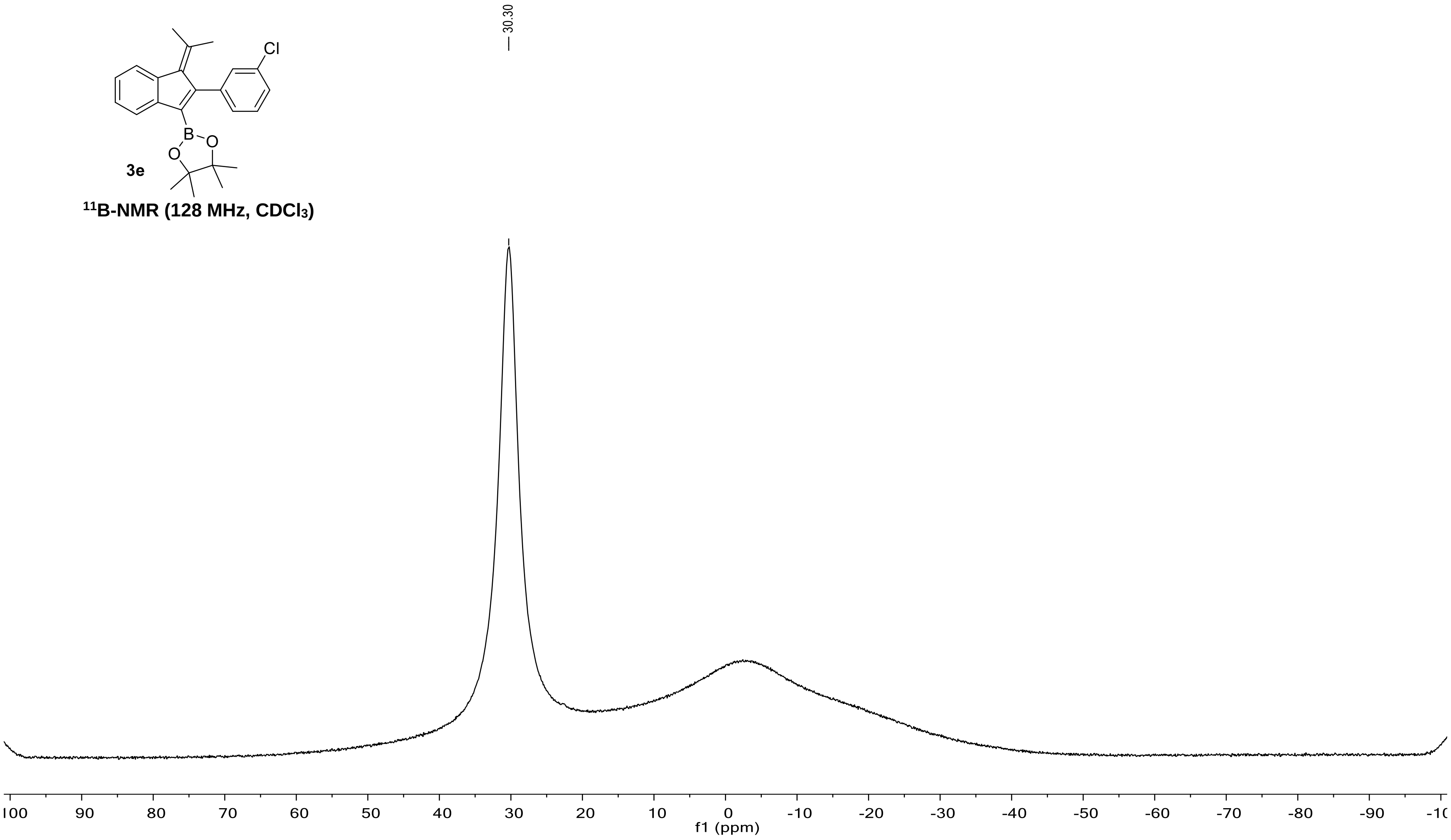

30.30
Cl
B
O
O
3e
11B-NMR (128 MHz, CDCl3)
100
90
80
70
60
50
40
30
20
10
0
-10
-20
-30
-40
-50
-60
-70
-80
-90
f1 (ppm)

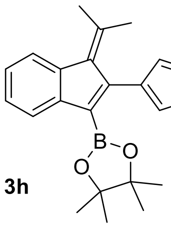

3h

^{1}H-NMR (400 MHz, CDCl₃)

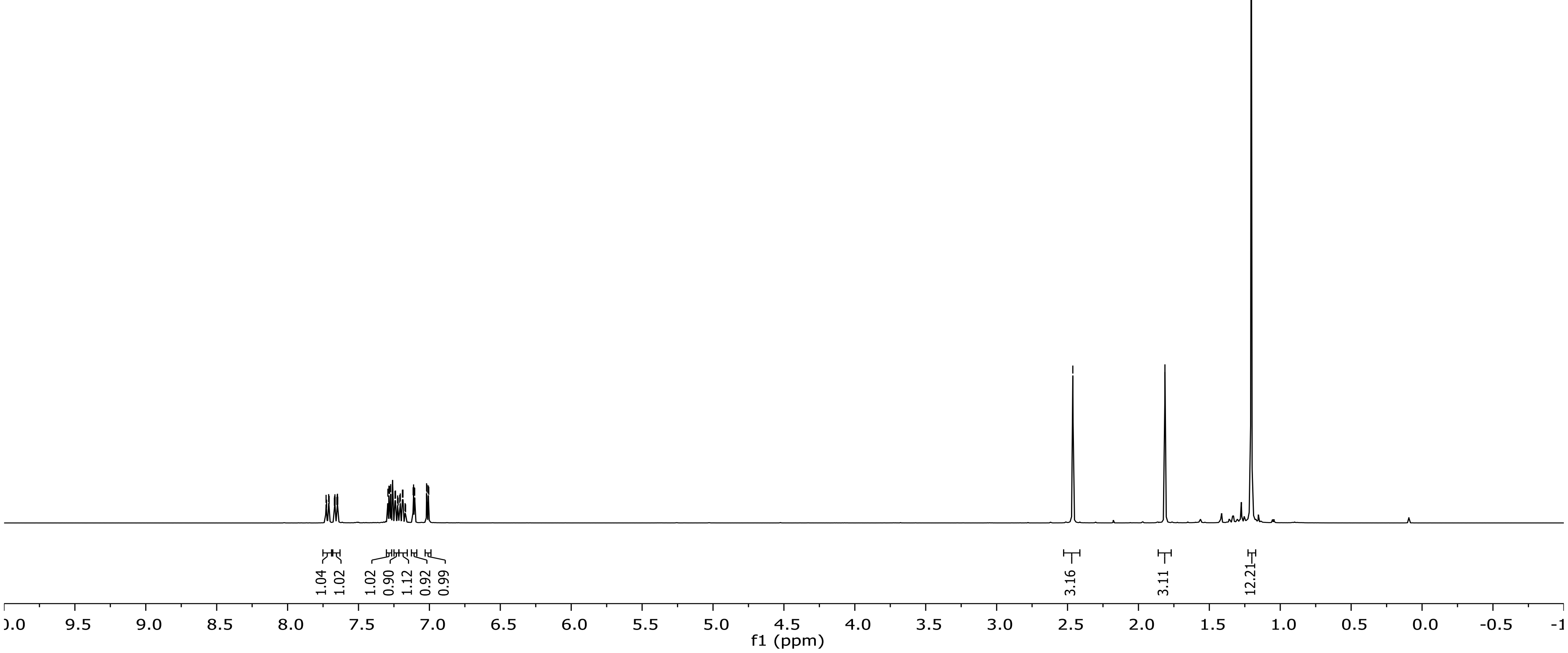

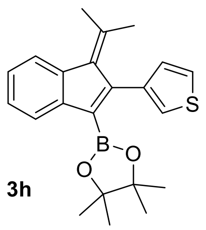

3h

^{13}C-NMR (100 MHz, $CDCl_3$)

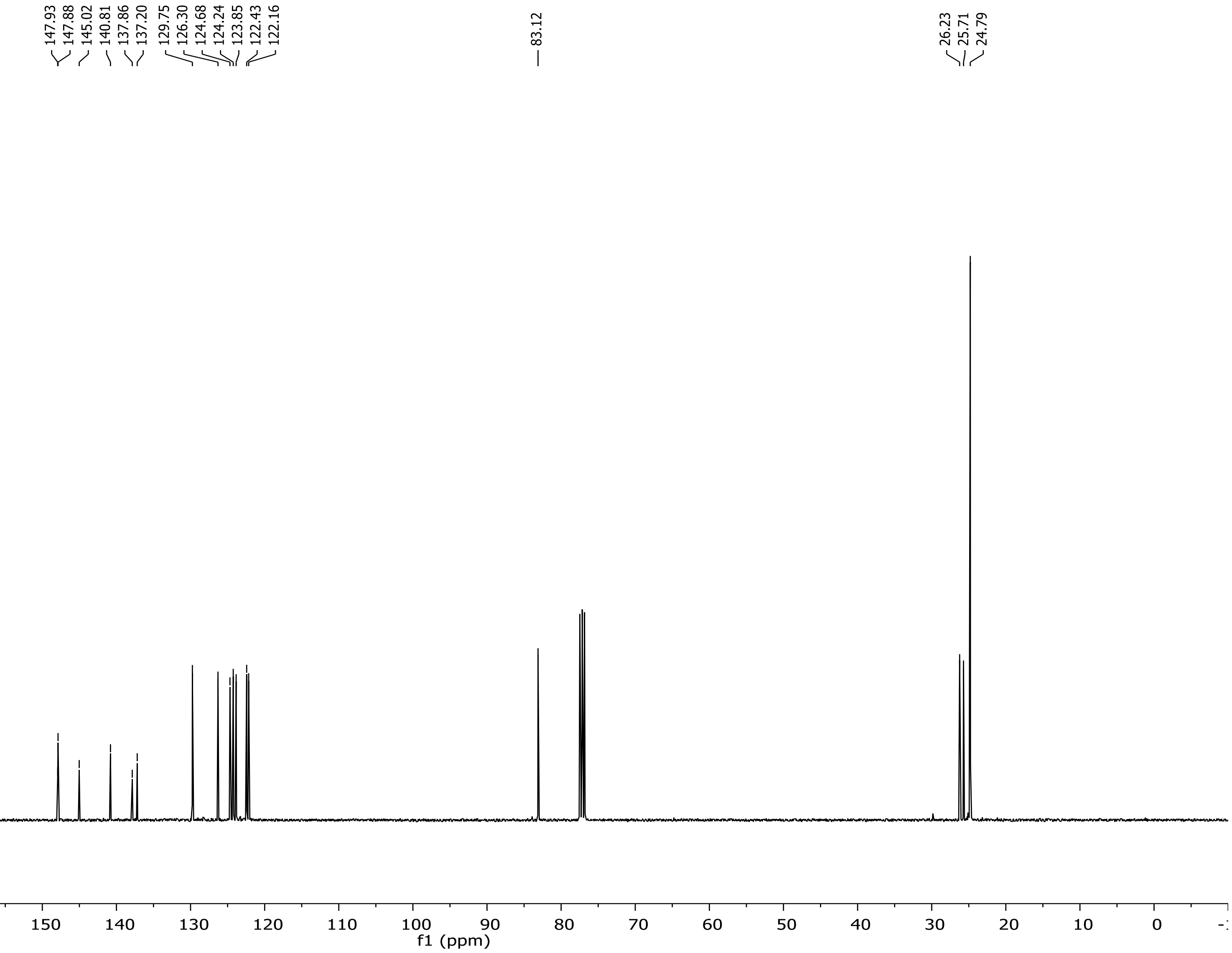

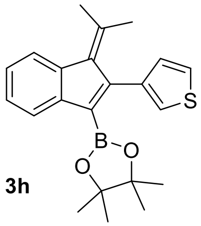

3h

^{11}B-NMR (128 MHz, $CDCl_3$)

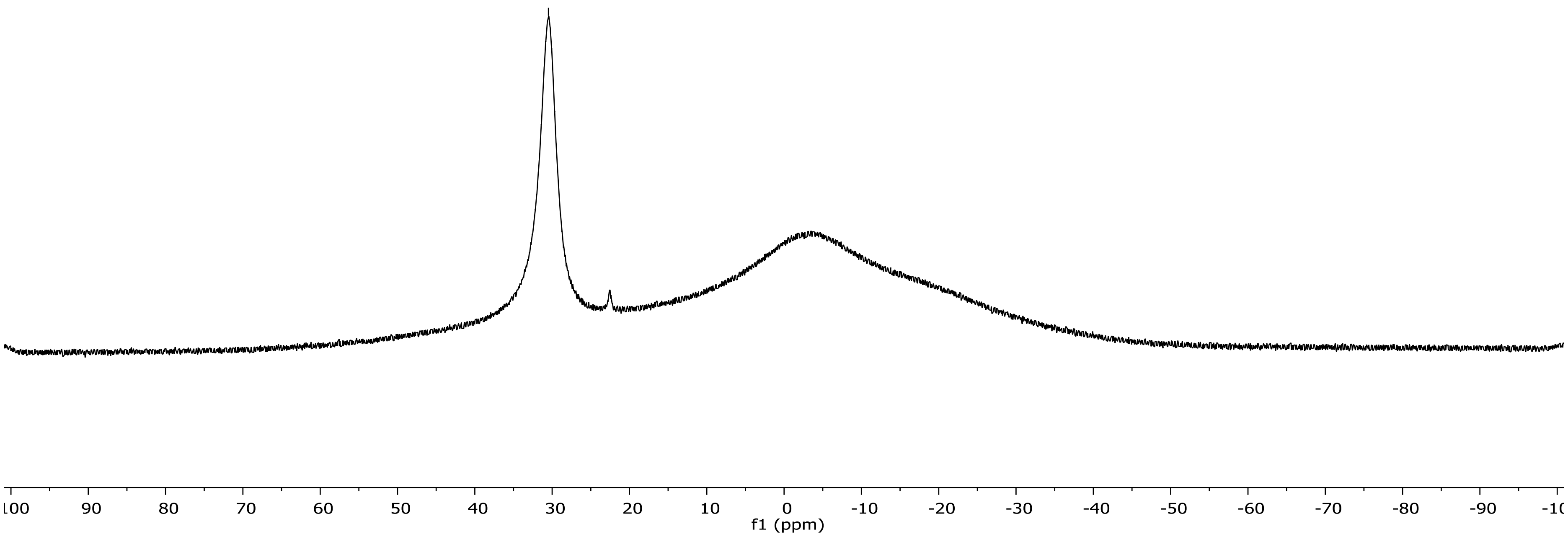

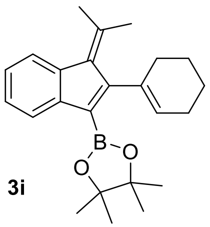

3i

^{1}H-NMR (400 MHz, $CDCl_3$)

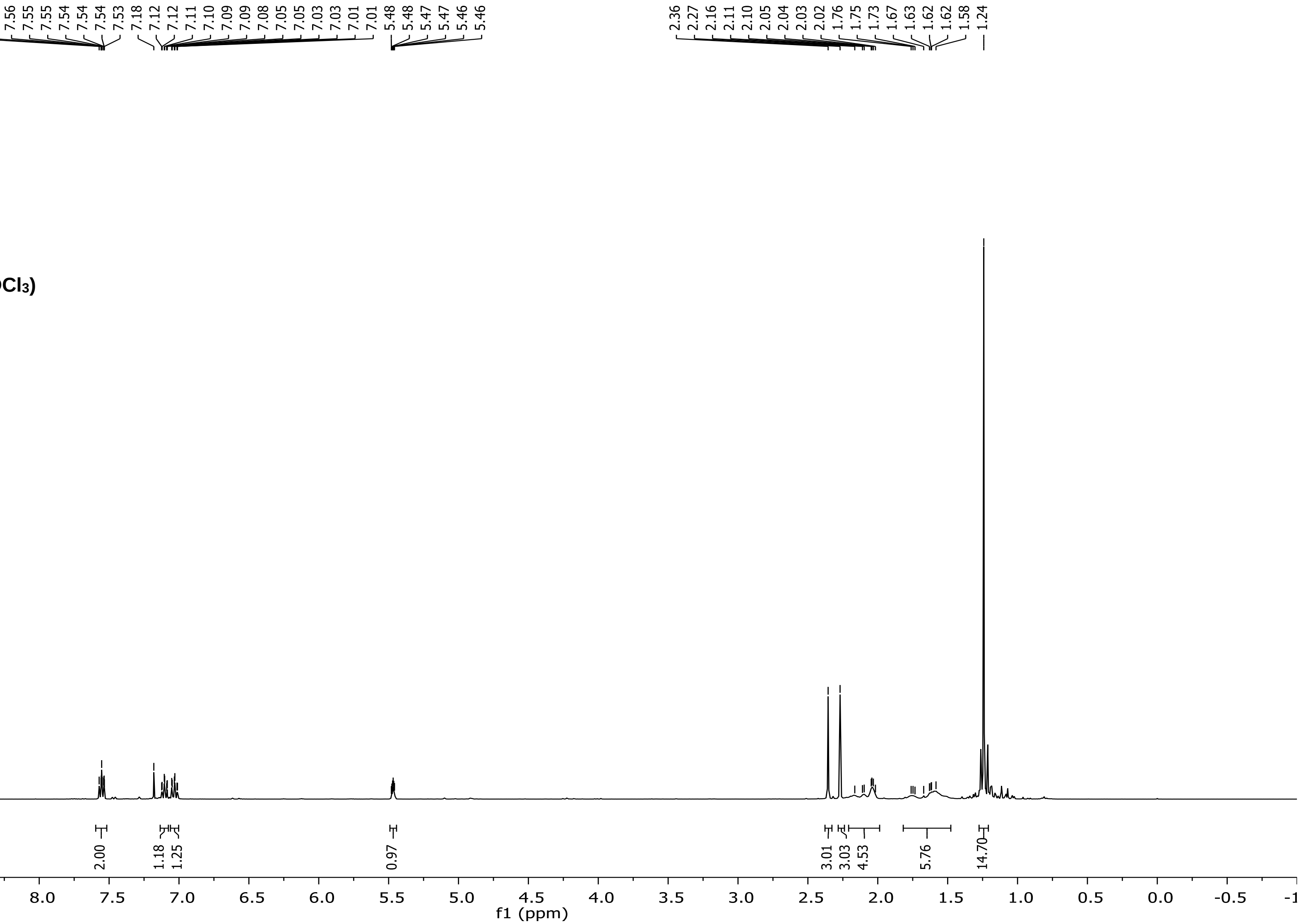

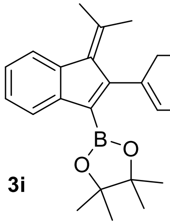

3i

^{13}C-NMR (100 MHz, $CDCl_3$)

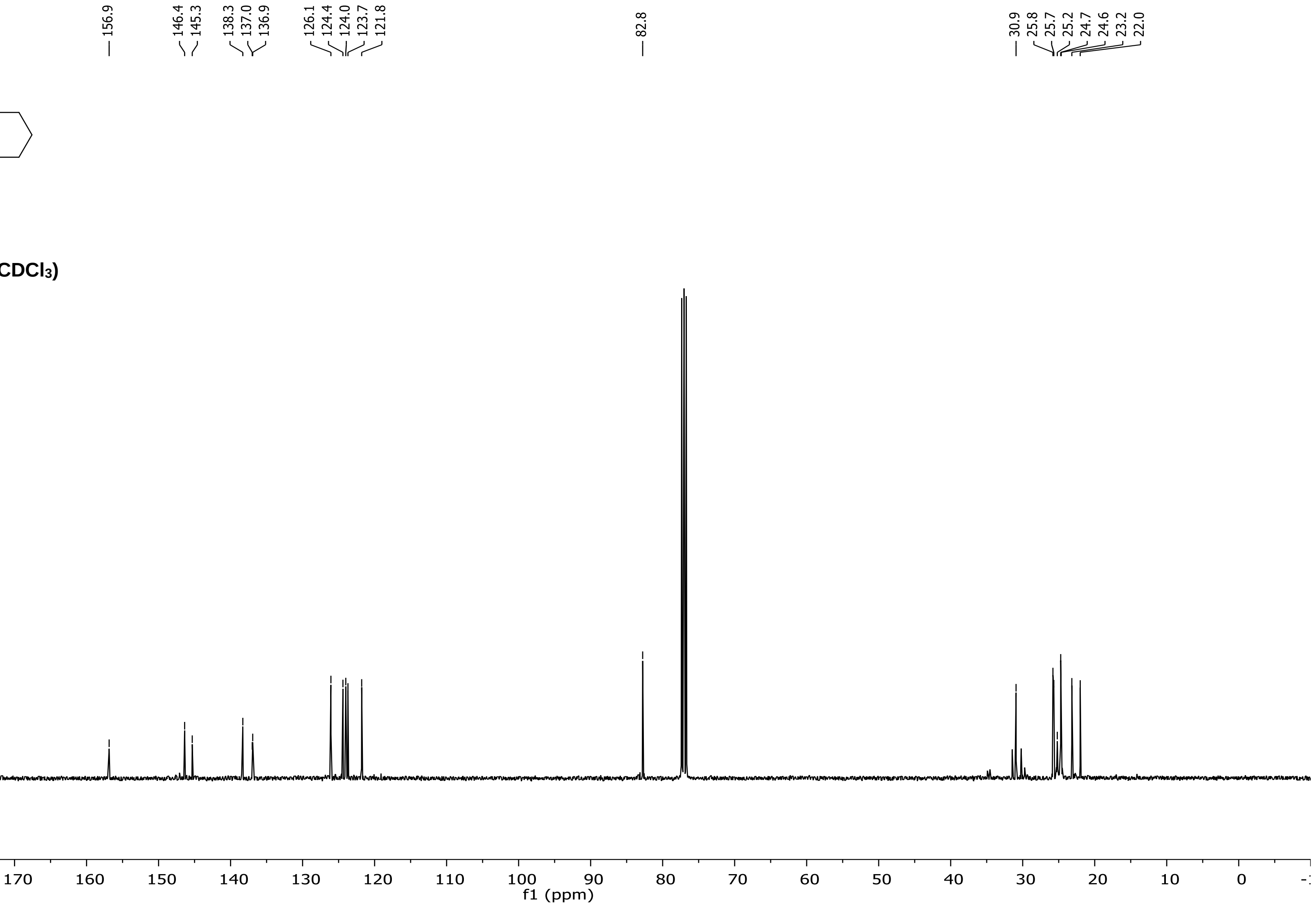

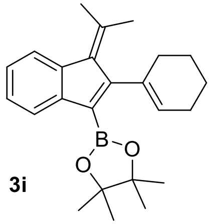

3i

^{11}**B-NMR (128 MHz, CDCl$_3$)**

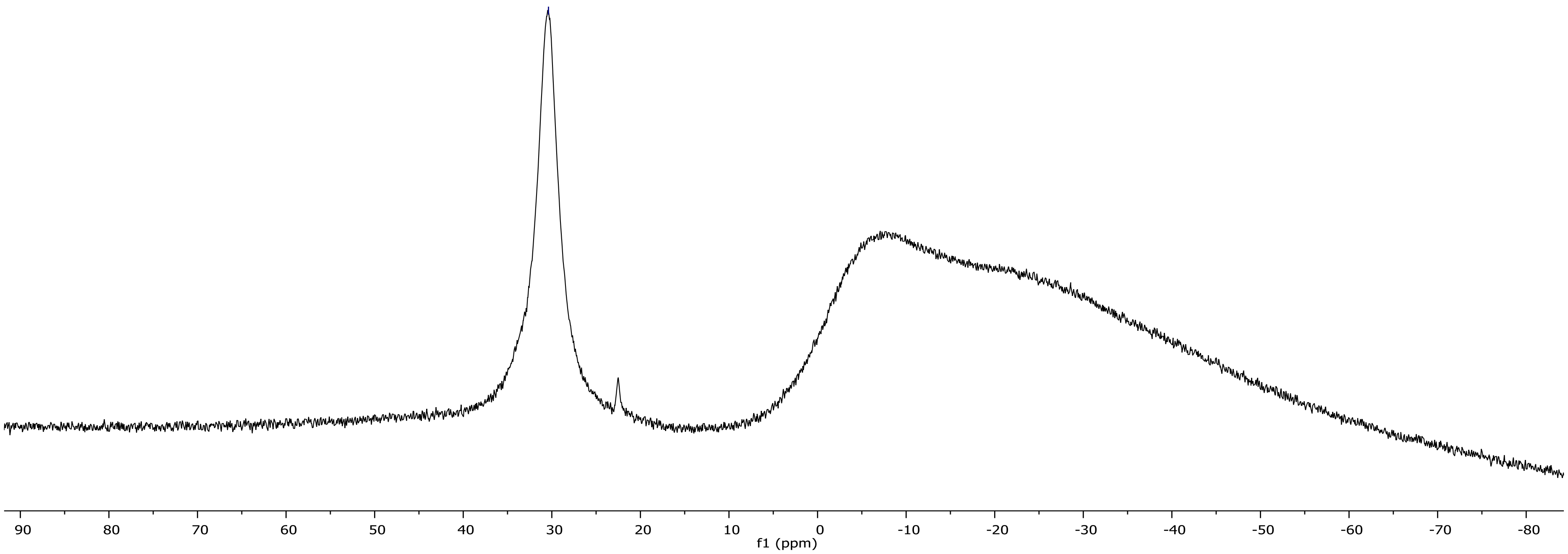

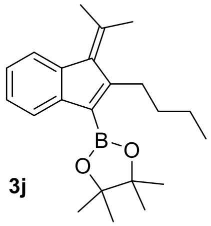

3j

^{1}H-NMR (300 MHz, $CDCl_3$)

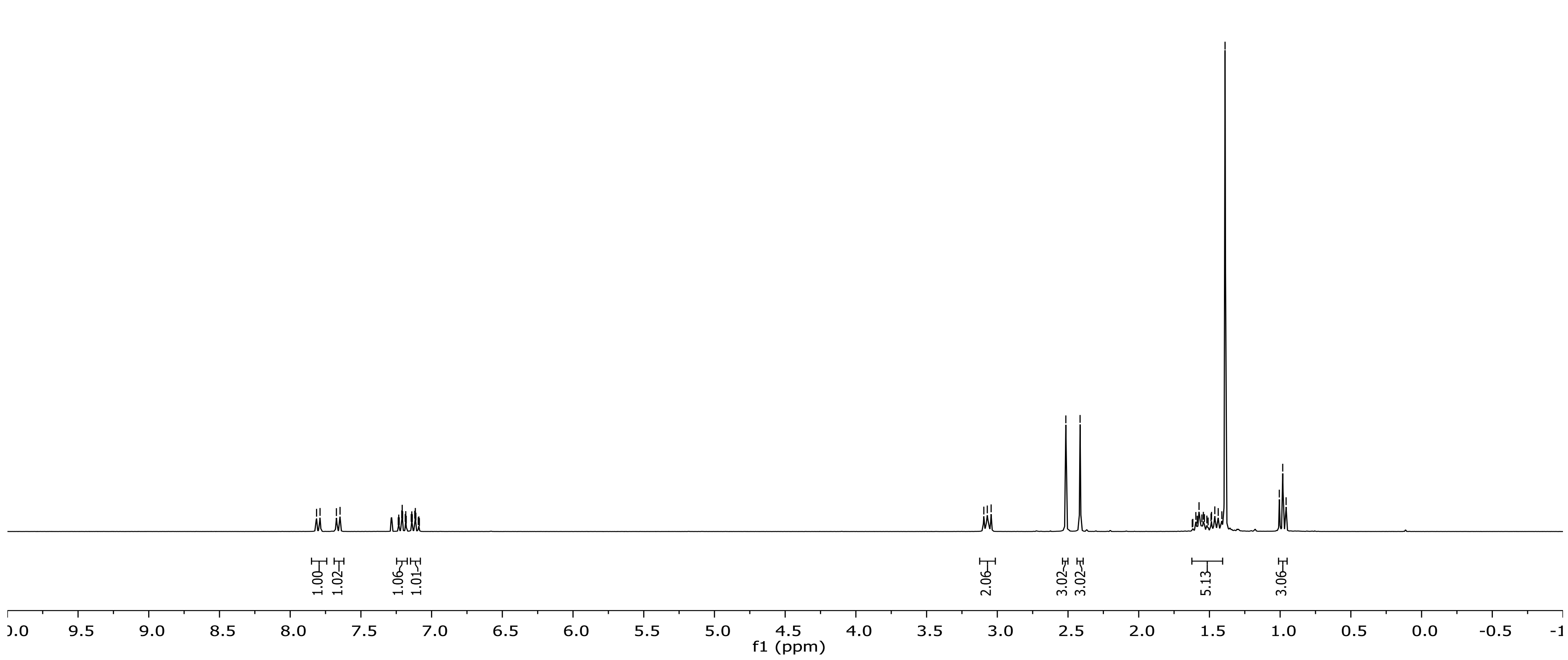

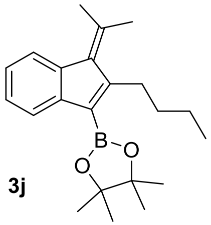

3j

^{13}C-NMR (100 MHz, $CDCl_3$)

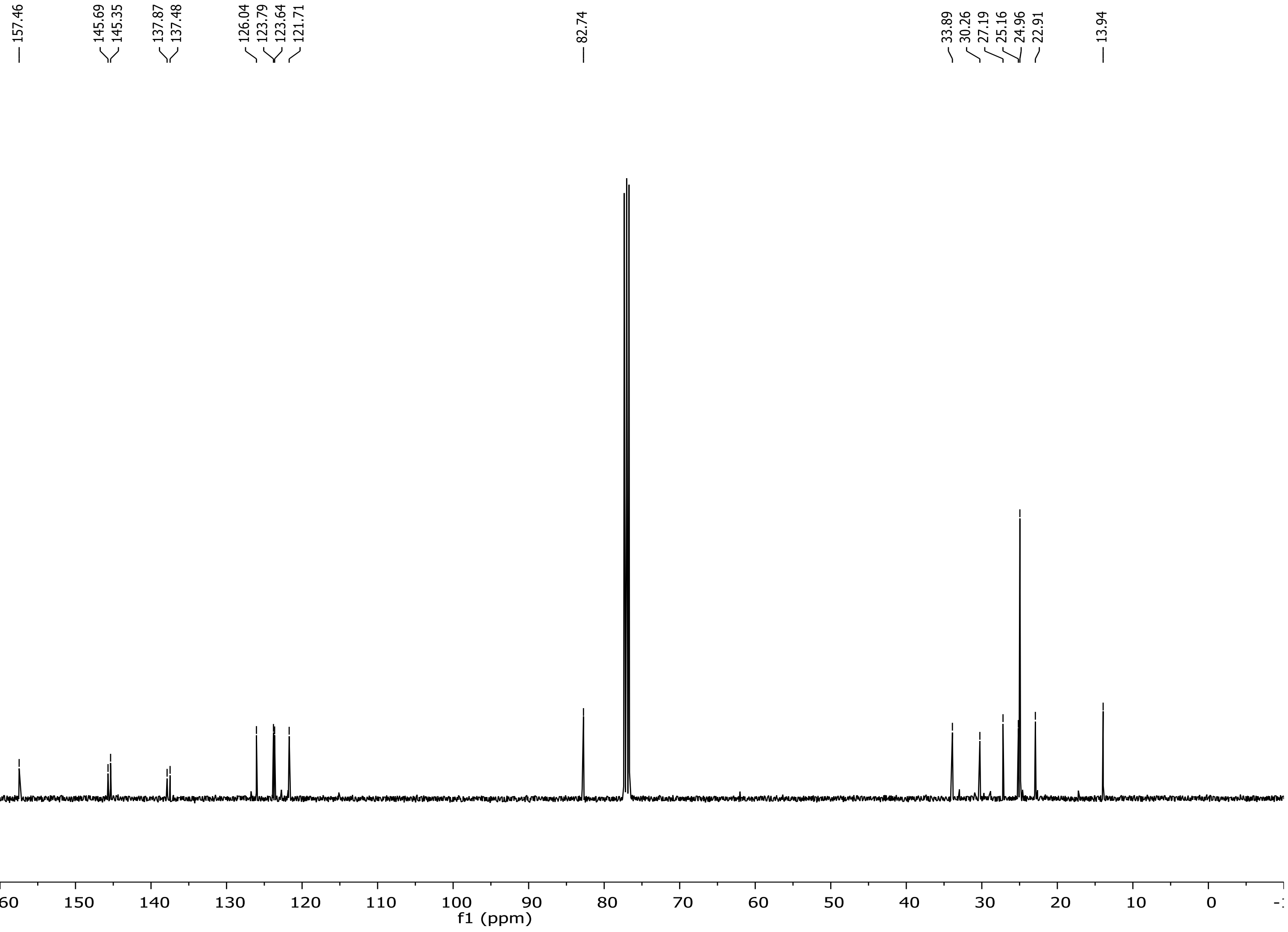

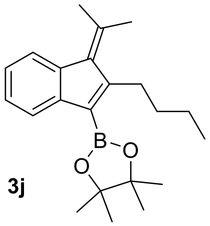

3j

^{11}B-NMR (128 MHz, CDCl₃)

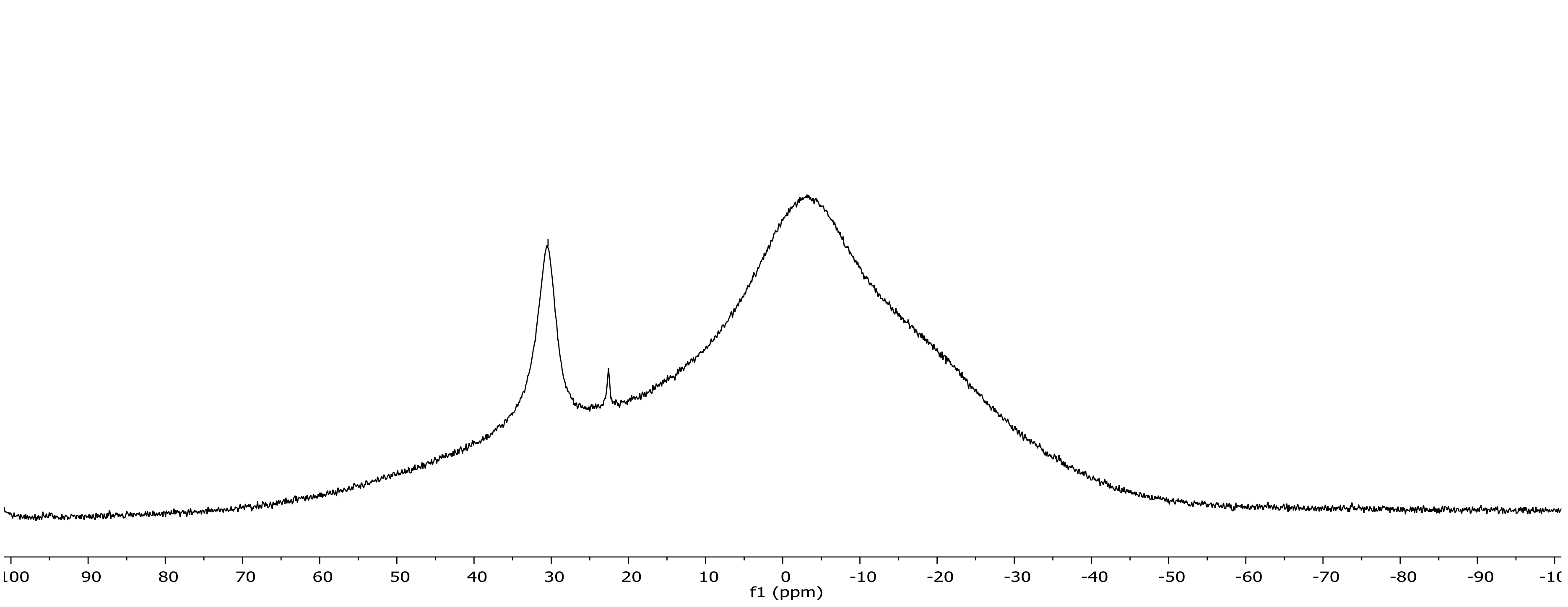

3k

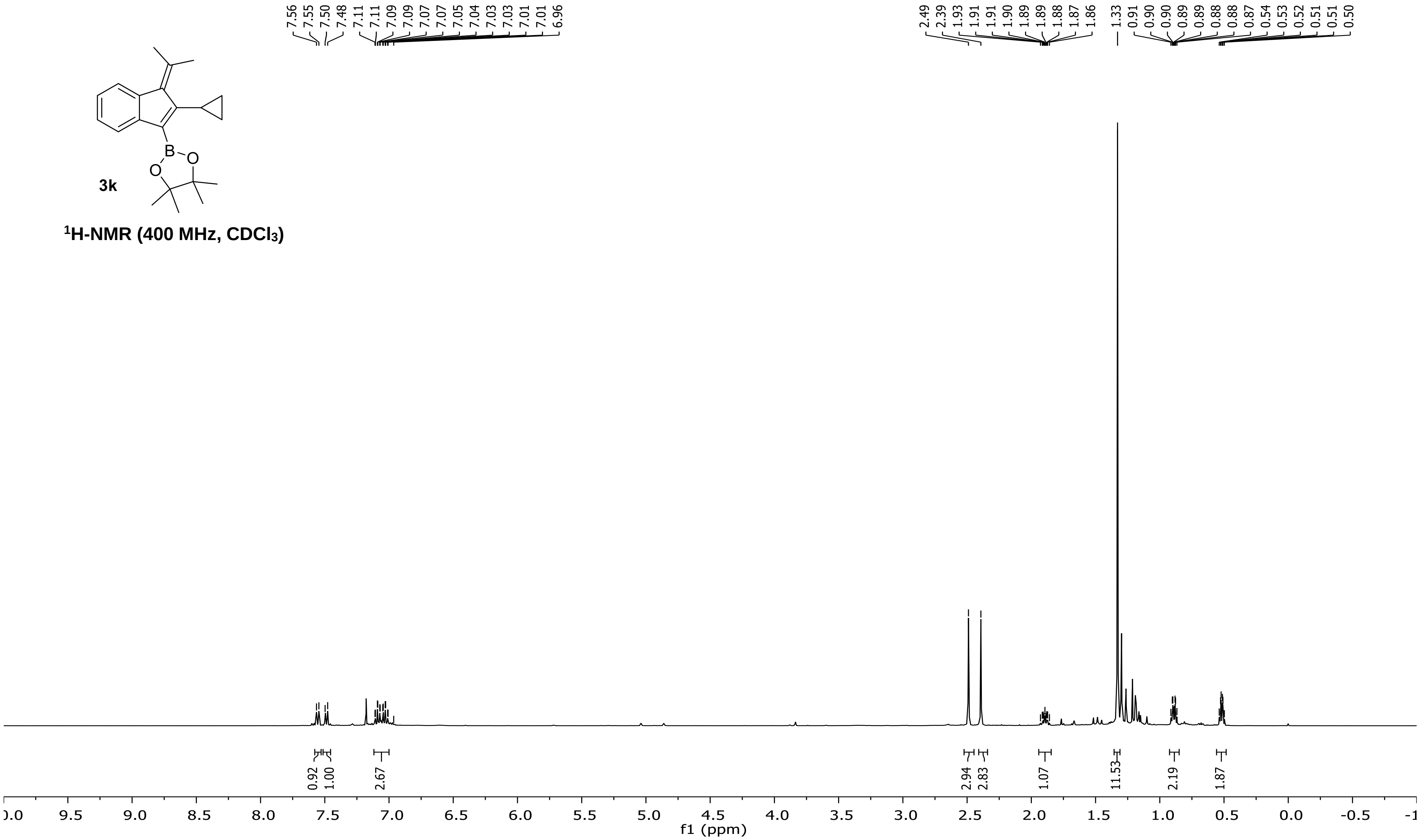

^{1}H-NMR (400 MHz, $CDCl_3$)

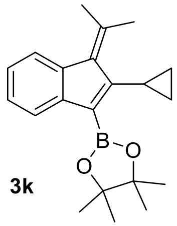

3k

^{13}C-NMR (100 MHz, $CDCl_3$)

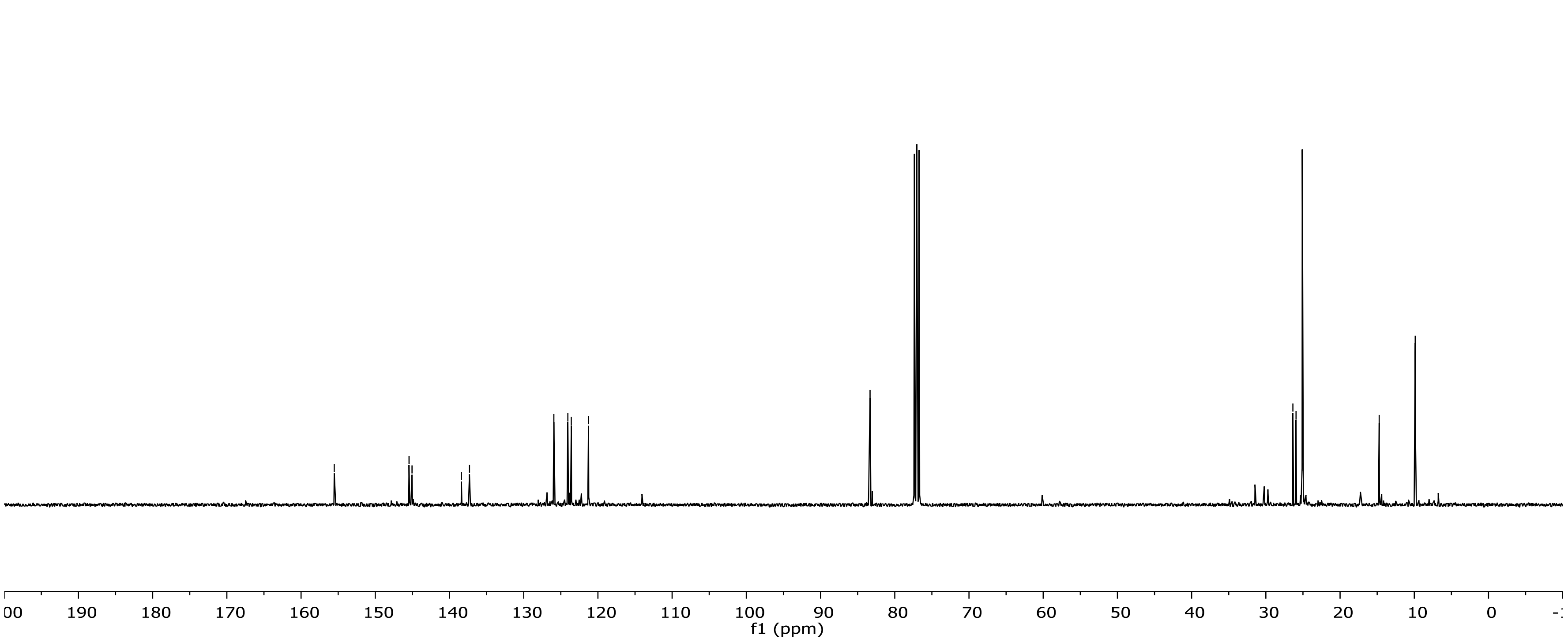

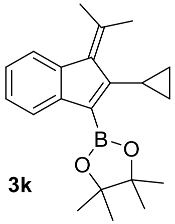

3k

^{11}B-NMR (128 MHz, CDCl$_3$)

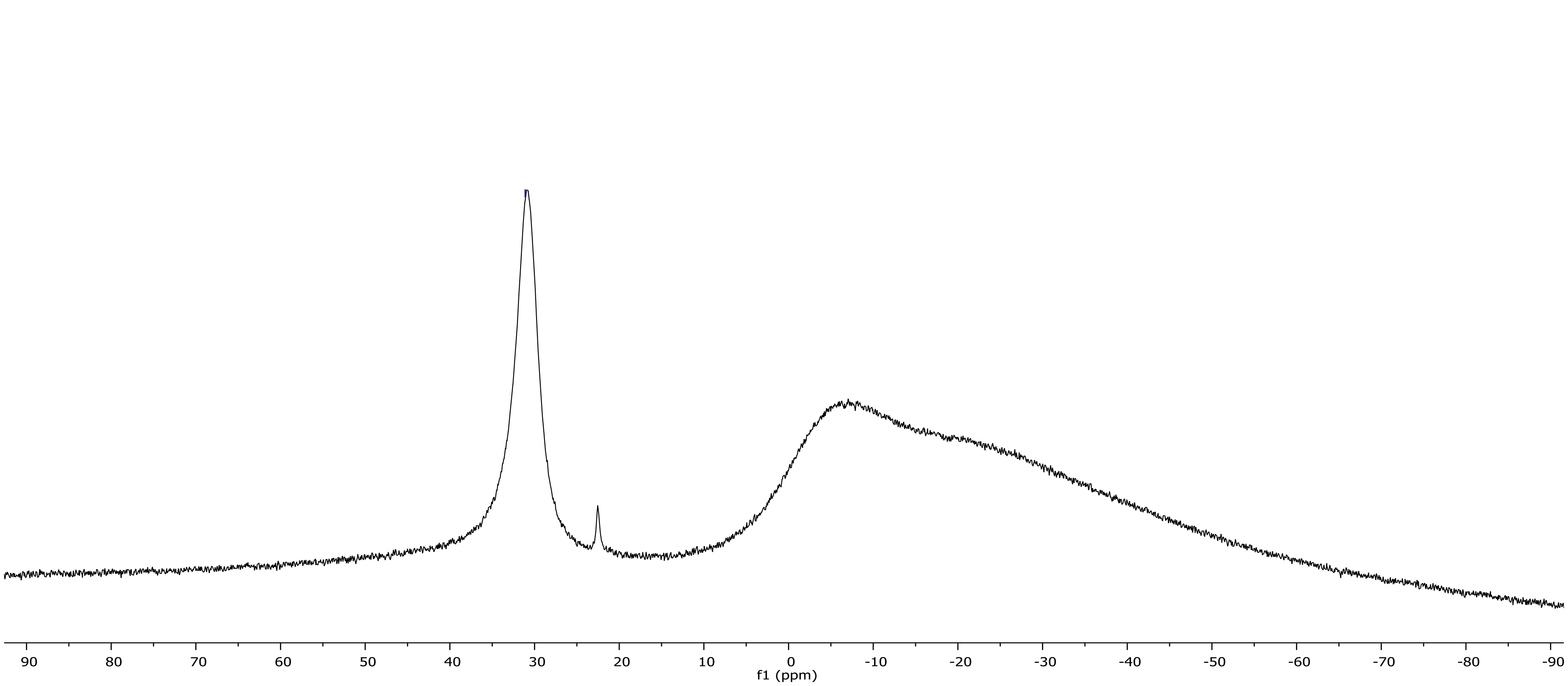

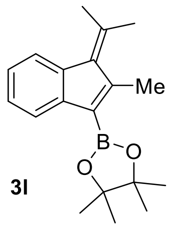

3l

^{1}H-NMR (400 MHz, $CDCl_3$)

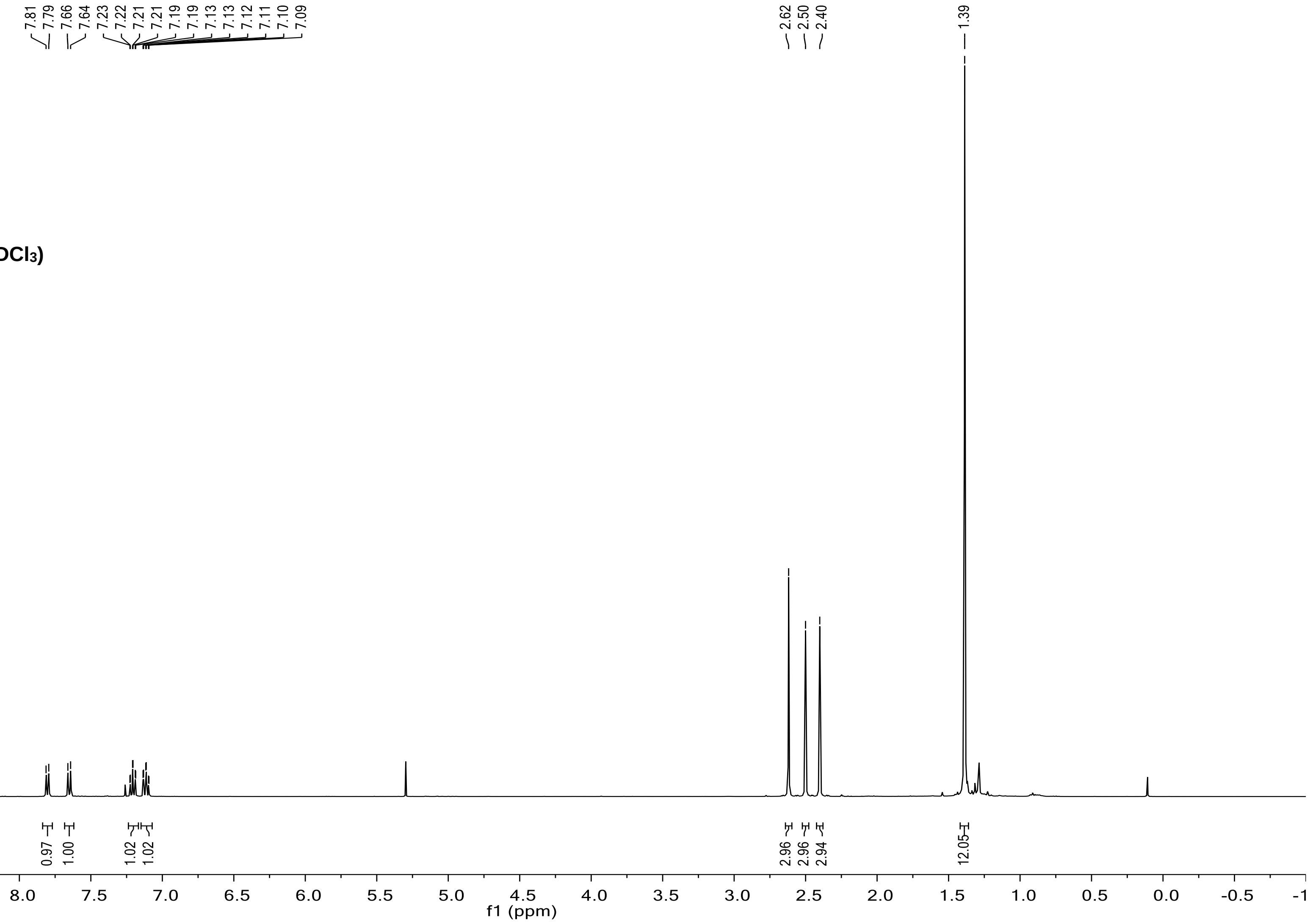

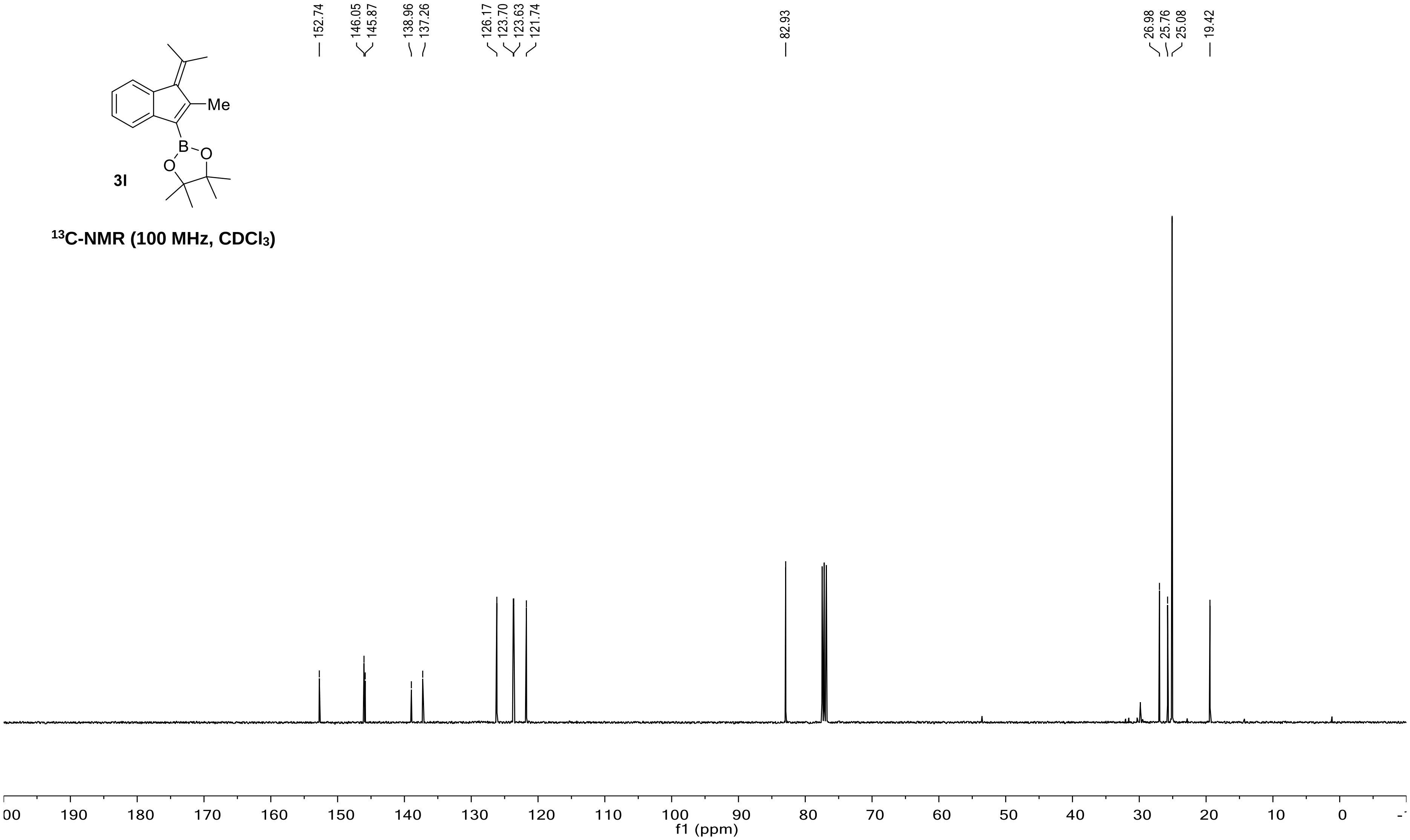
152.74
146.05
145.87
138.96
137.26
126.17
123.70
123.63
121.74
82.93
26.98
25.76
25.08
19.42
Me
B
O
O
3l
^{13}C-NMR (100 MHz, $CDCl_3$)
00
190
180
170
160
150
140
130
120
110
100
90
80
70
60
50
40
30
20
10
0
f1 (ppm)

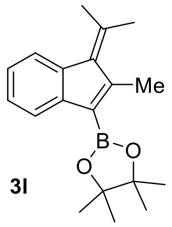

3l

^{11}B-NMR (128 MHz, CDCl$_3$)

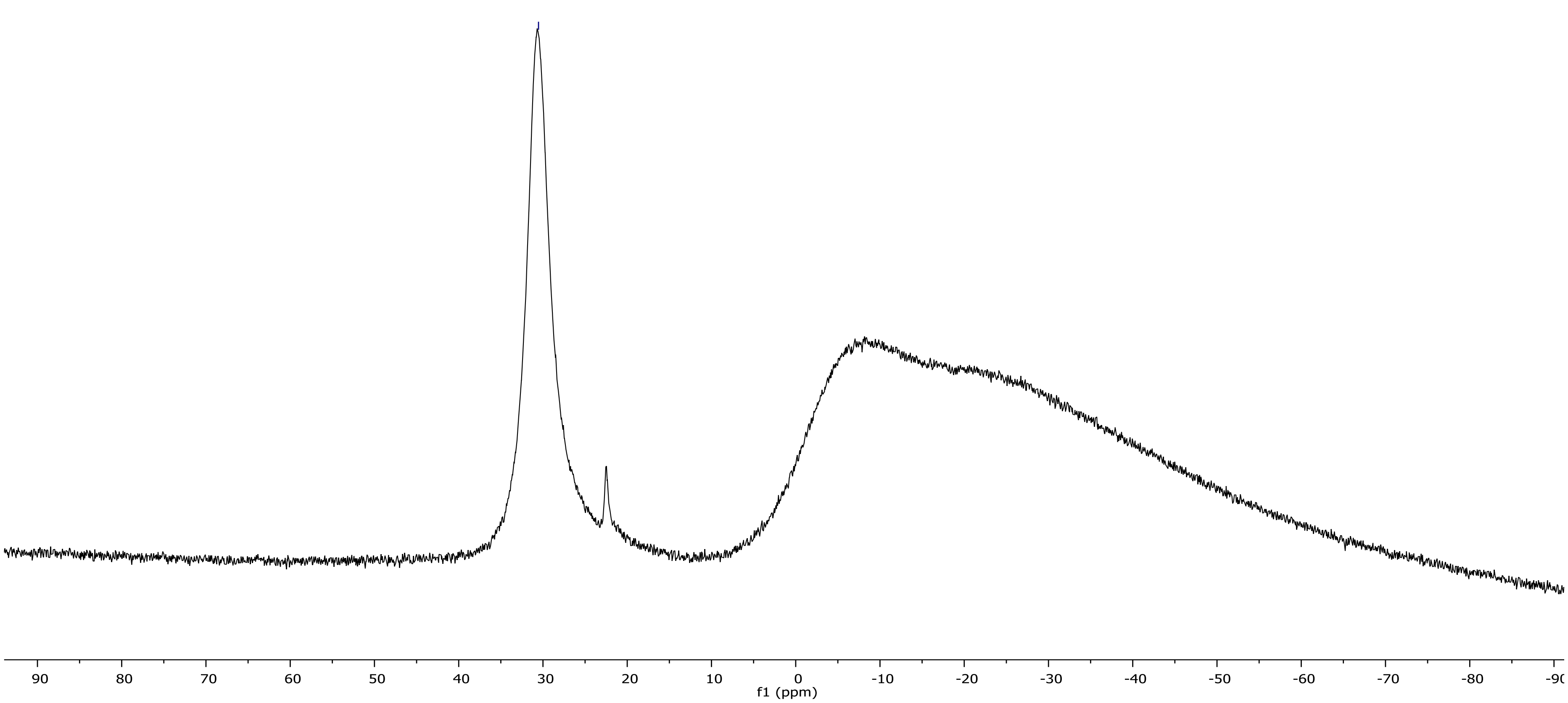

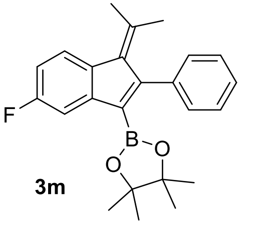

3m

^{1}H-NMR (300 MHz, CDCl$_3$)

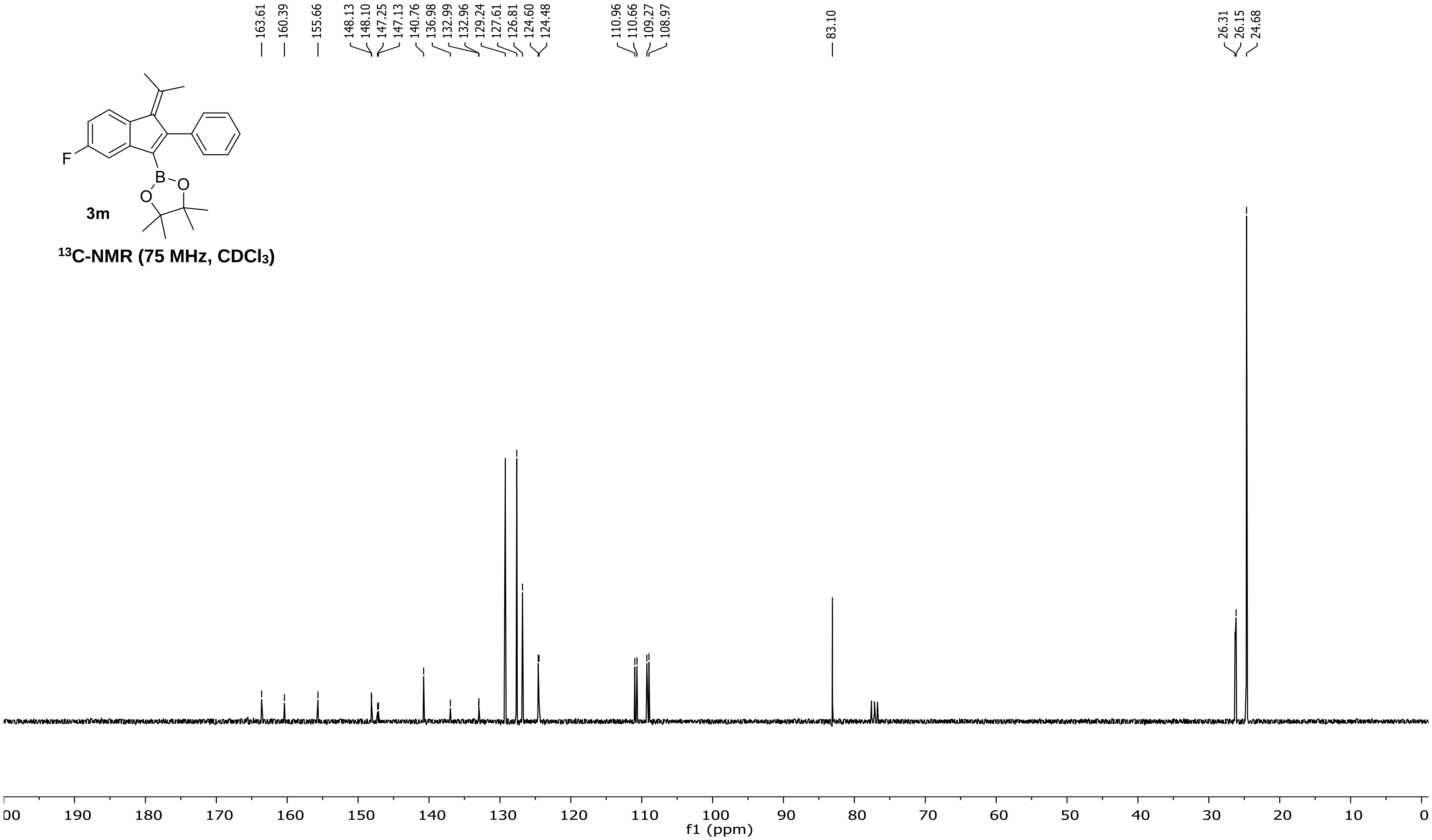

163.61
160.39
155.66
148.13
148.10
147.25
147.13
140.76
136.98
132.99
132.96
129.24
127.61
126.81
124.60
124.48
110.96
110.66
109.27
108.97
83.10
26.31
26.15
24.68
F
B
O
O
3m
13C-NMR (75 MHz, CDCl3)
00
190
180
170
160
150
140
130
120
110
100
90
80
70
60
50
40
30
20
10
0
f1 (ppm)

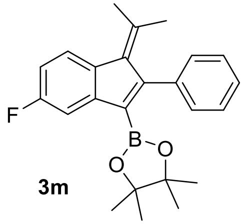

3m

^{11}B-NMR (160 MHz, CDCl3)

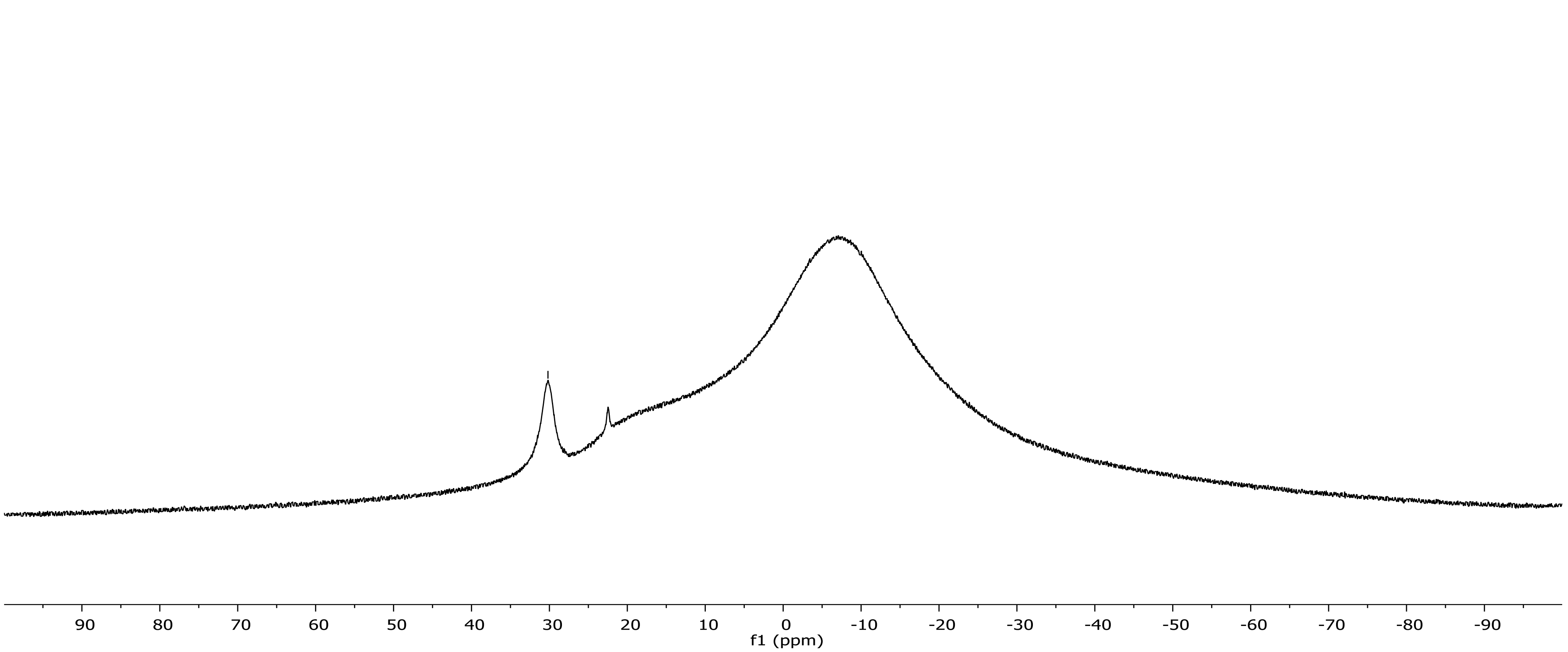

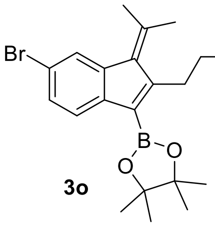

^{1}H-NMR (300 MHz, $CDCl_3$)

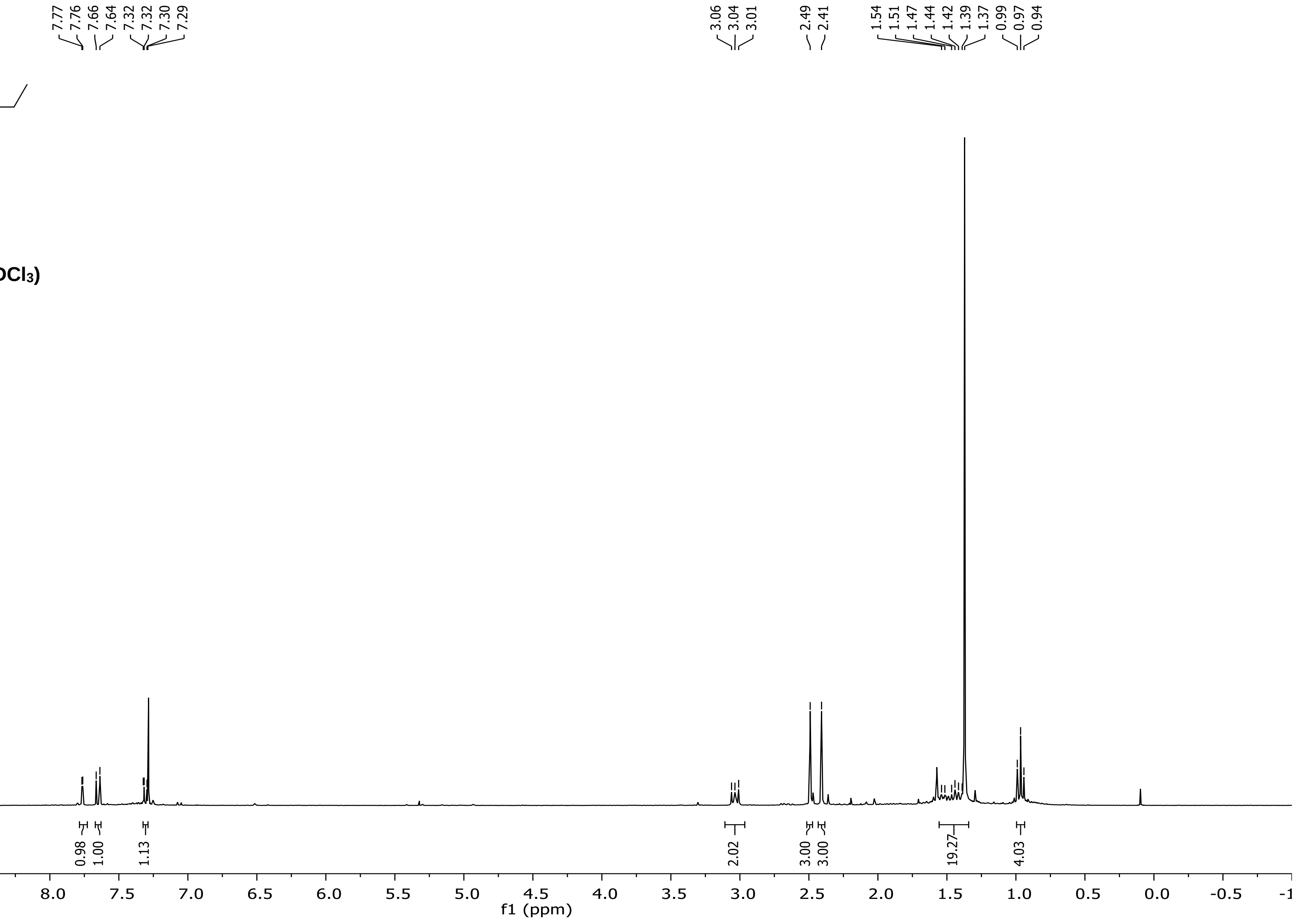

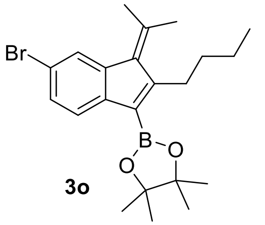

3o

^{13}C-NMR (75 MHz, CDCl$_3$)

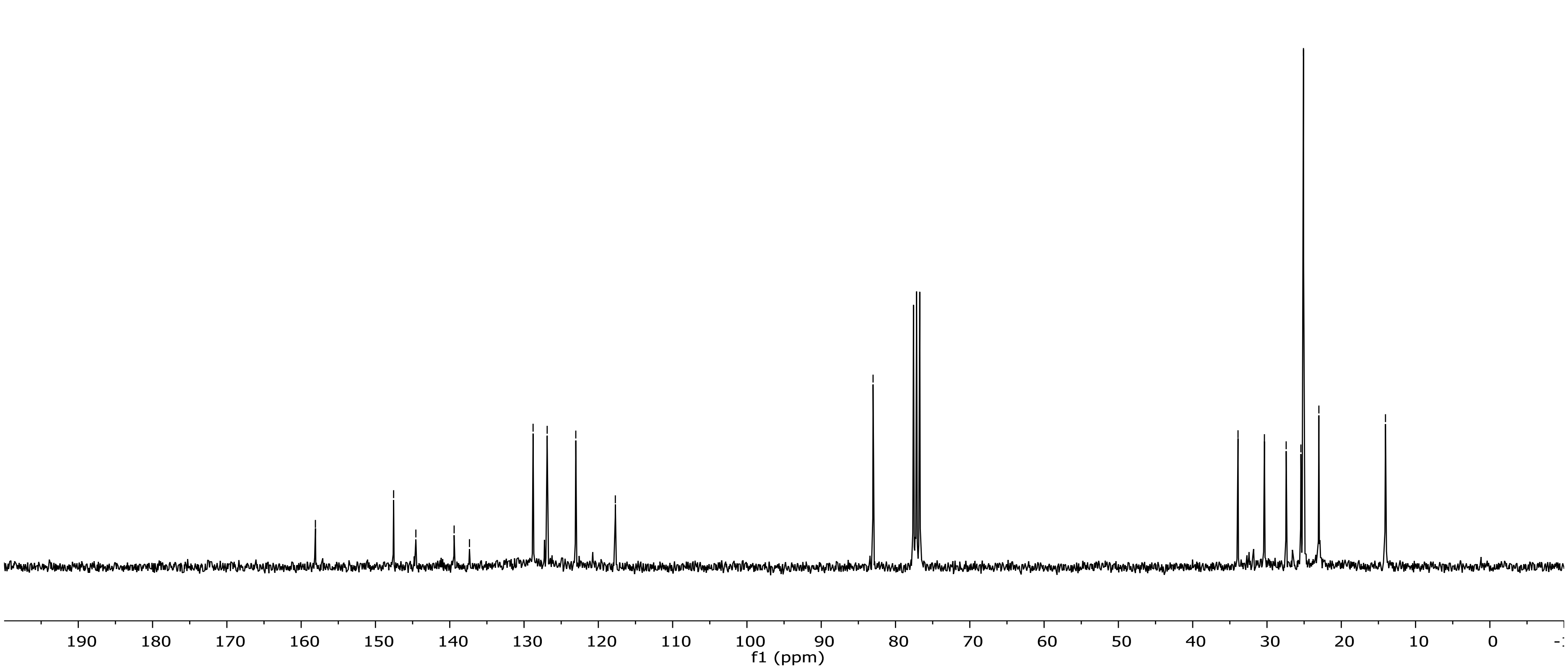

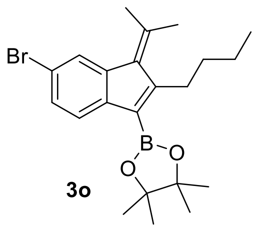

3o

^{11}B-NMR (160 MHz, CDCl$_3$)

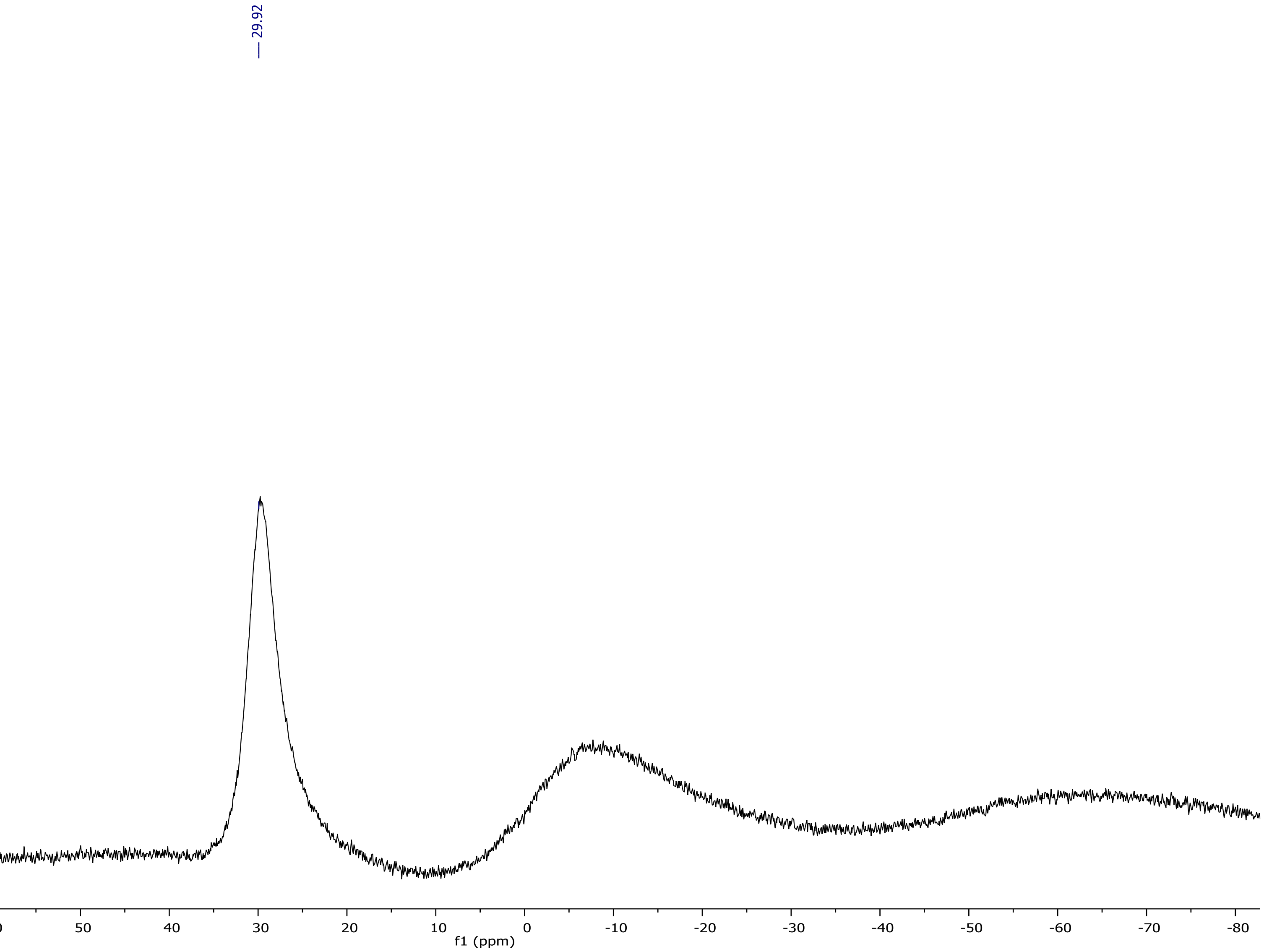

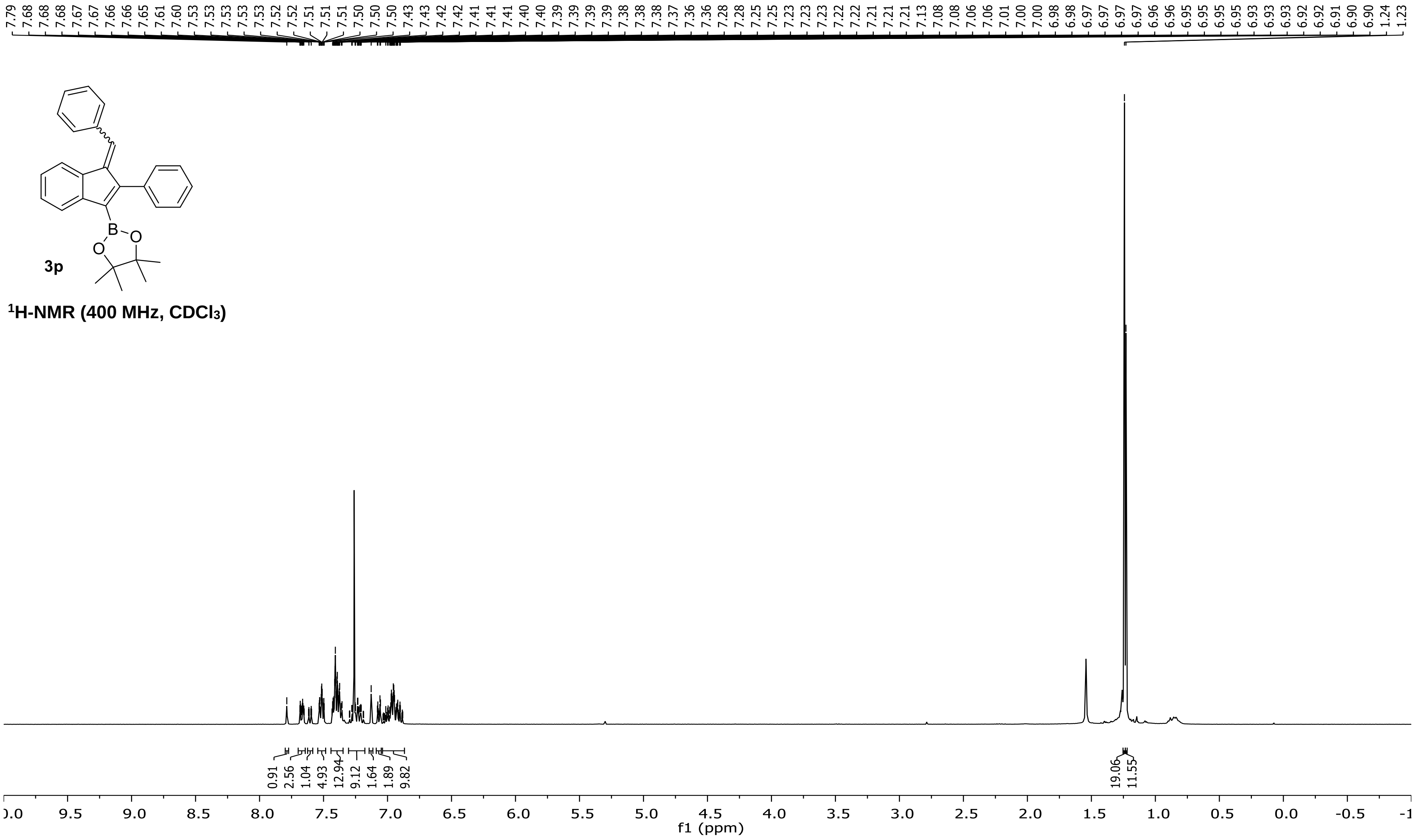
7.79 7.68 7.68 7.67 7.67 7.66 7.66 7.65 7.61 7.60 7.53 7.53 7.53 7.53 7.52 7.51 7.51 7.51 7.50 7.50 7.43 7.43 7.42 7.41 7.41 7.41 7.40 7.39 7.39 7.39 7.39 7.38 7.38 7.38 7.37 7.36 7.36 7.28 7.28 7.25 7.25 7.23 7.23 7.23 7.22 7.22 7.21 7.21 7.21 7.13 7.08 7.06 7.06 7.01 7.00 7.00 6.98 6.98 6.97 6.97 6.97 6.97 6.96 6.96 6.95 6.95 6.95 6.95 6.93 6.93 6.92 6.92 6.91 6.90 1.24 1.23
3p
1H-NMR (400 MHz, CDCl3)
0.91 2.56 1.04 4.93 12.94 9.12 1.64 1.89 9.82
19.06 11.55
f1 (ppm)

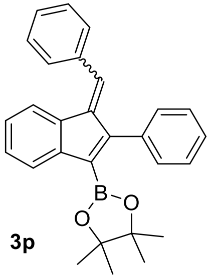

3p

^{13}C-NMR (100 MHz, CDCl$_3$)

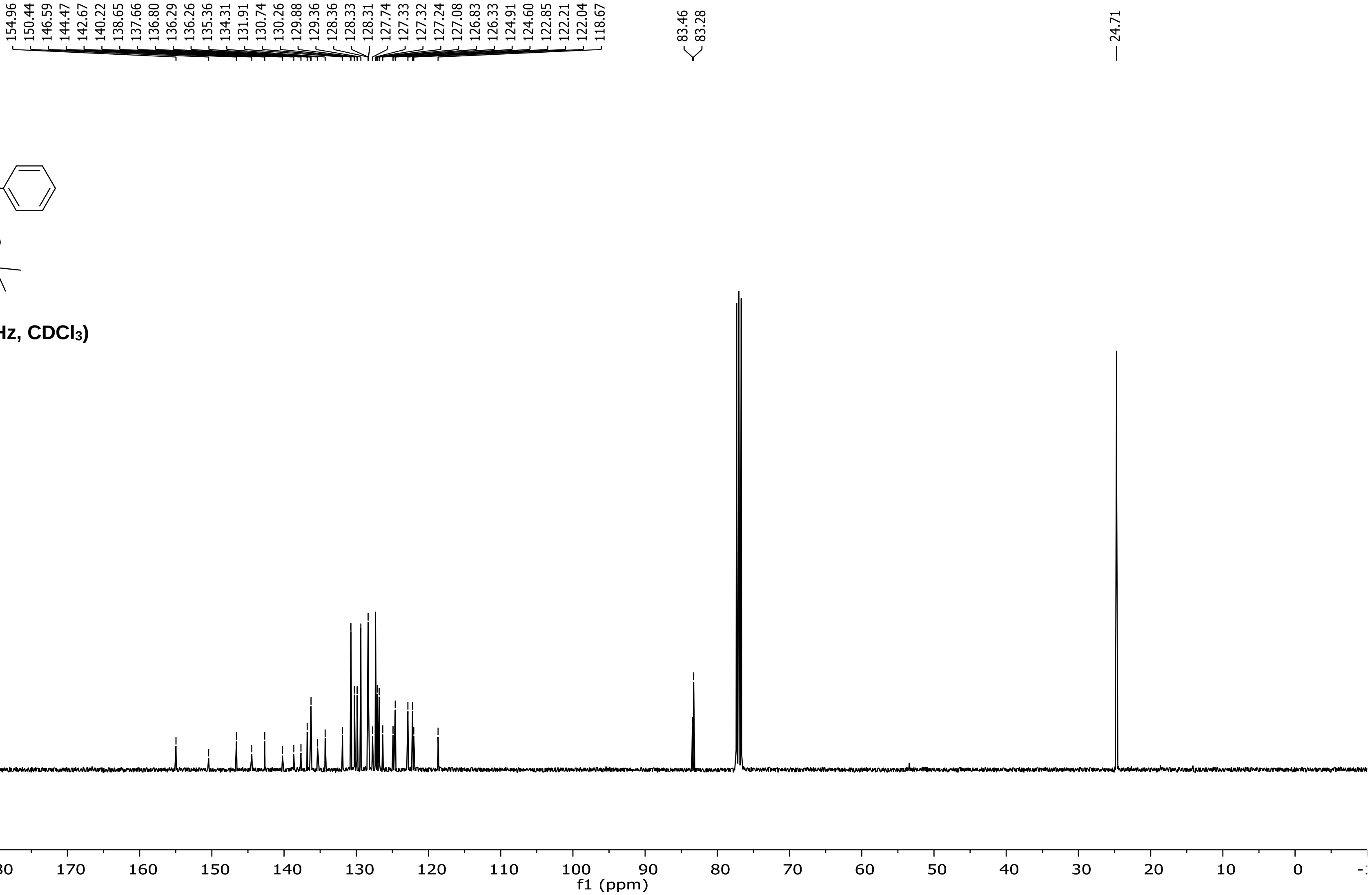

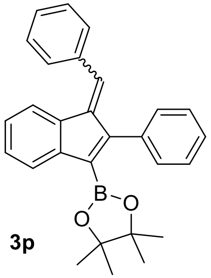

3p

^{11}B-NMR (128 MHz, $CDCl_3$)

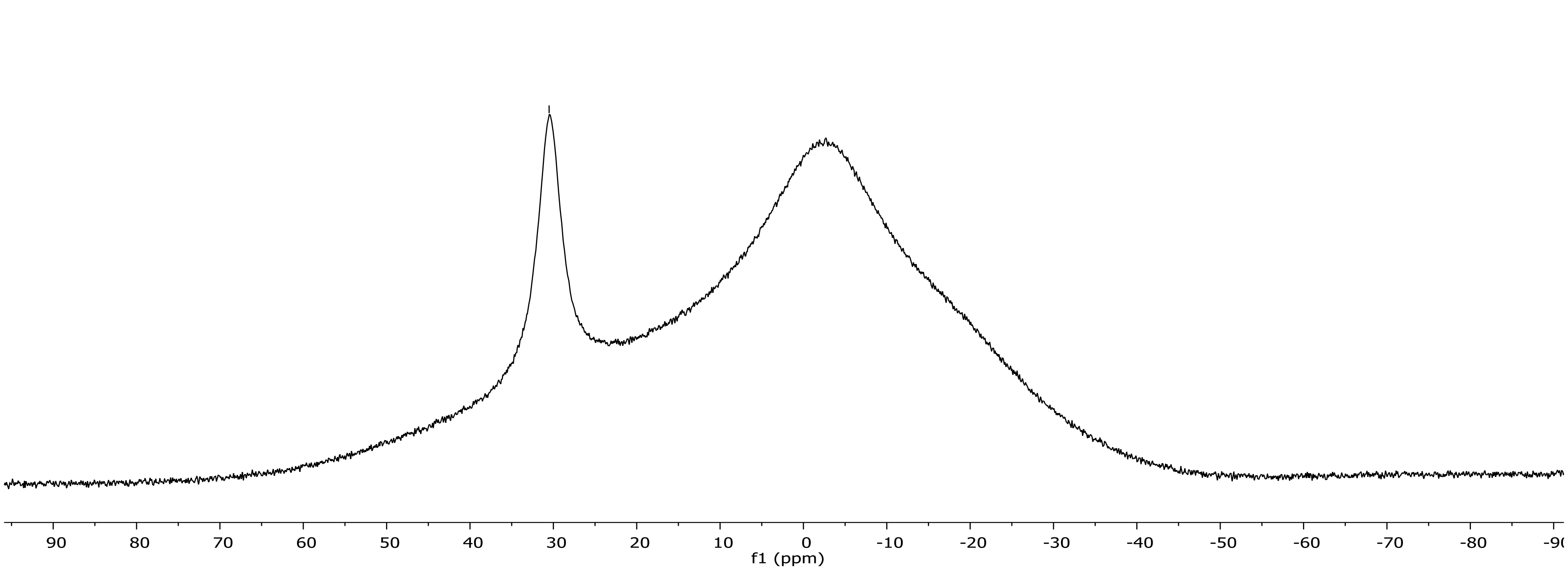

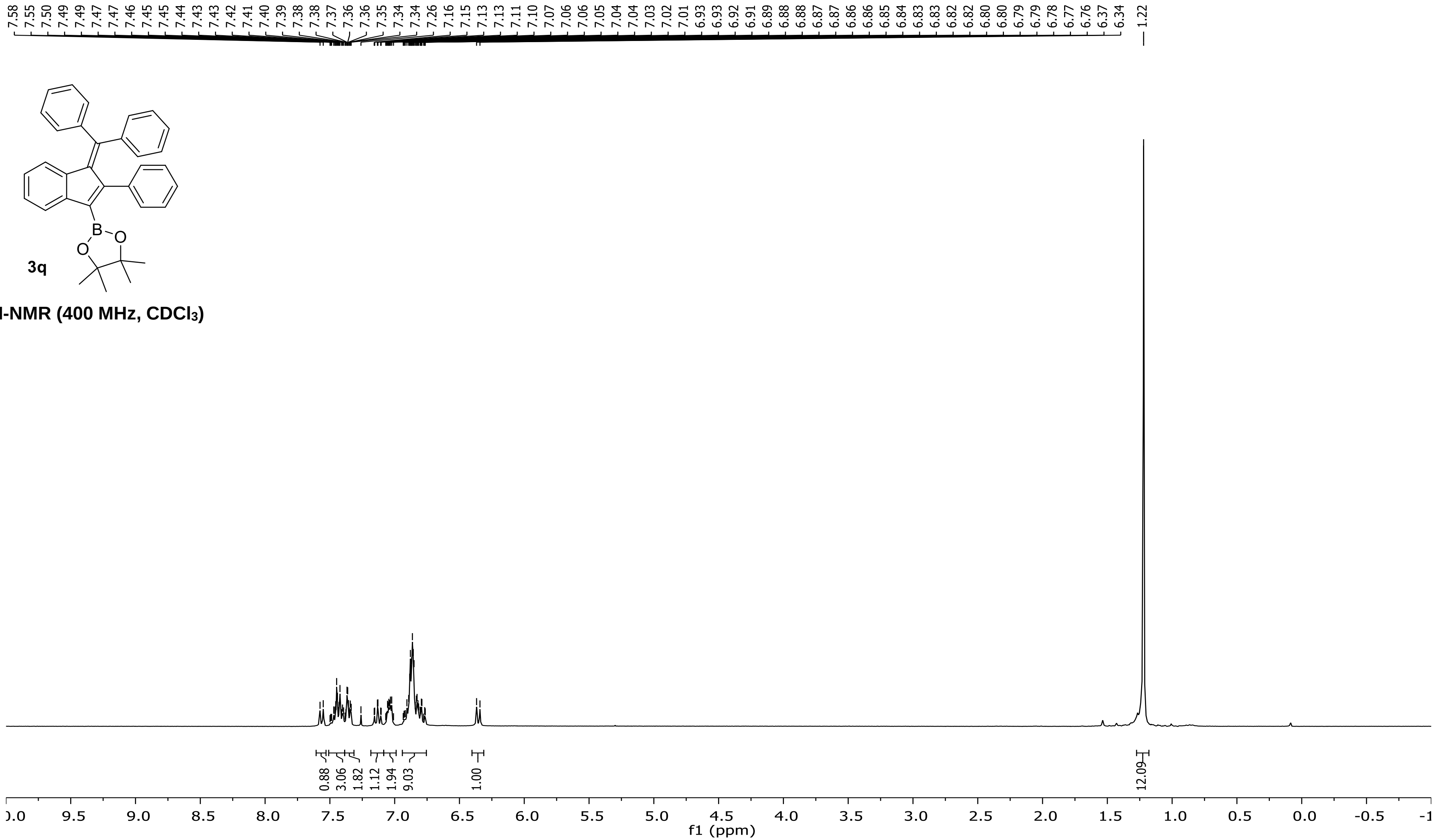

3q

^{1}H-NMR (400 MHz, CDCl$_3$)

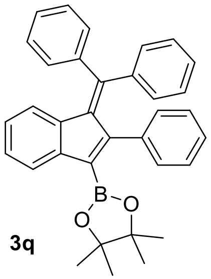

3q

^{13}C-NMR (100 MHz, CDCl$_3$)

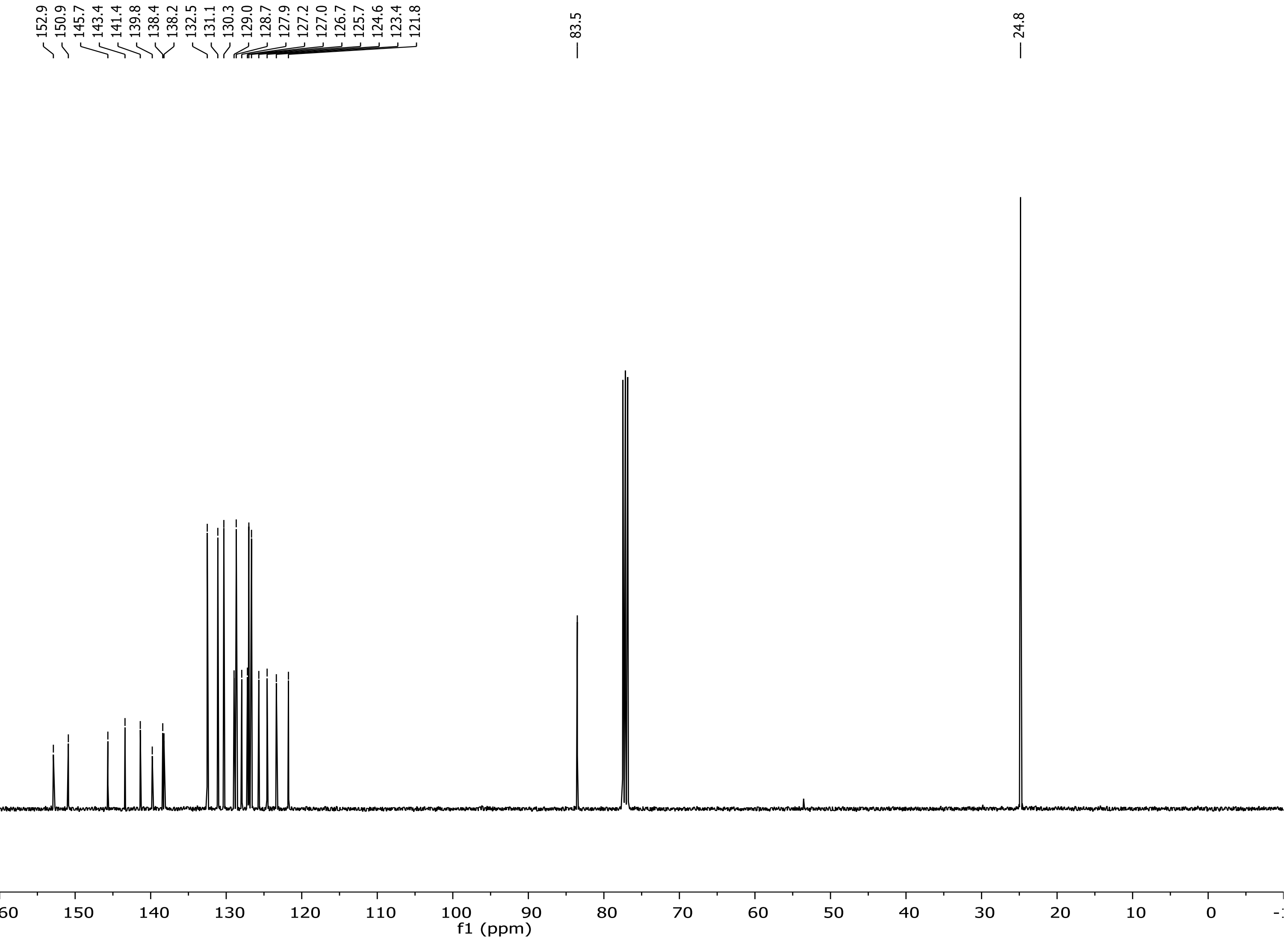

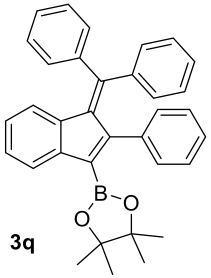

3q

^{11}B-NMR (128 MHz, $CDCl_3$)

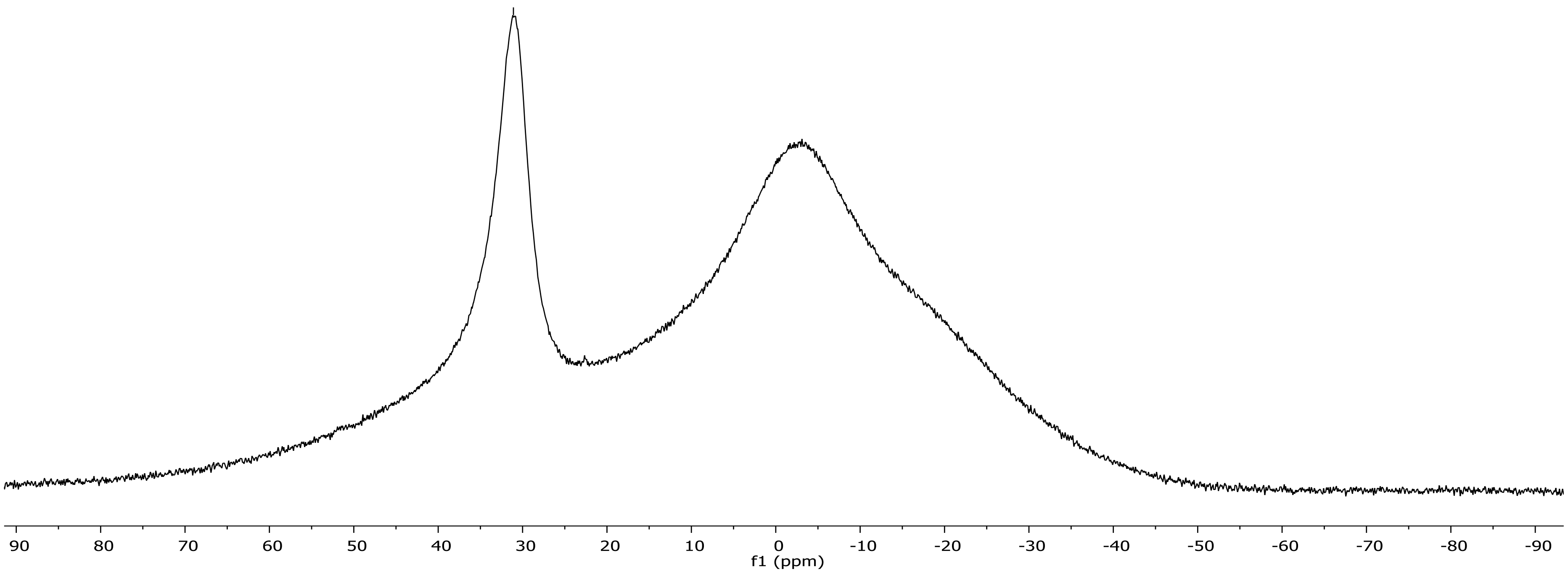

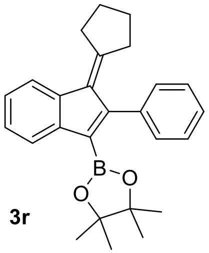

3r

^{1}H-NMR (300 MHz, CDCl$_3$)

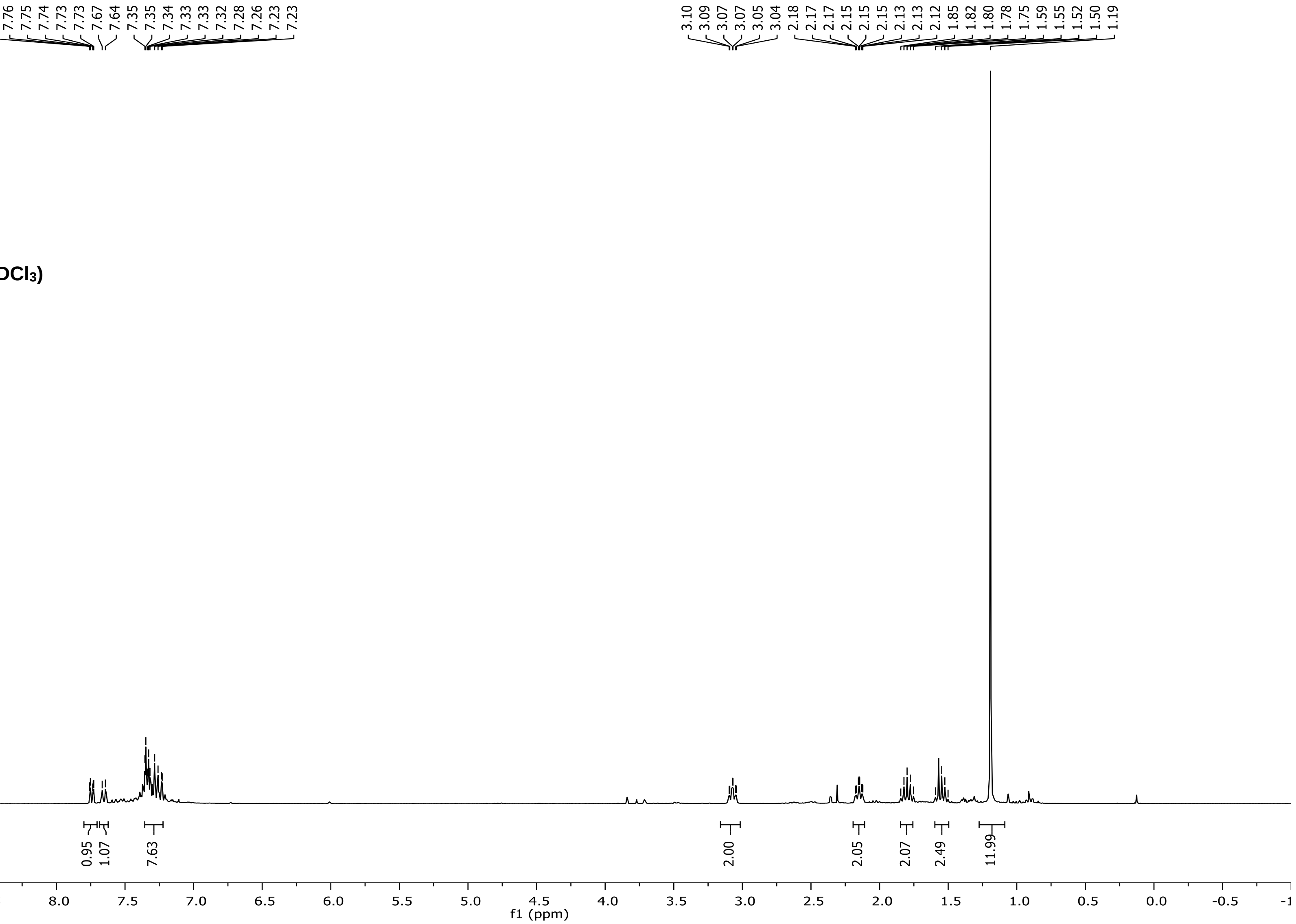

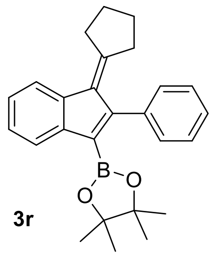

3r

^{13}C-NMR (75 MHz, CDCl$_3$)

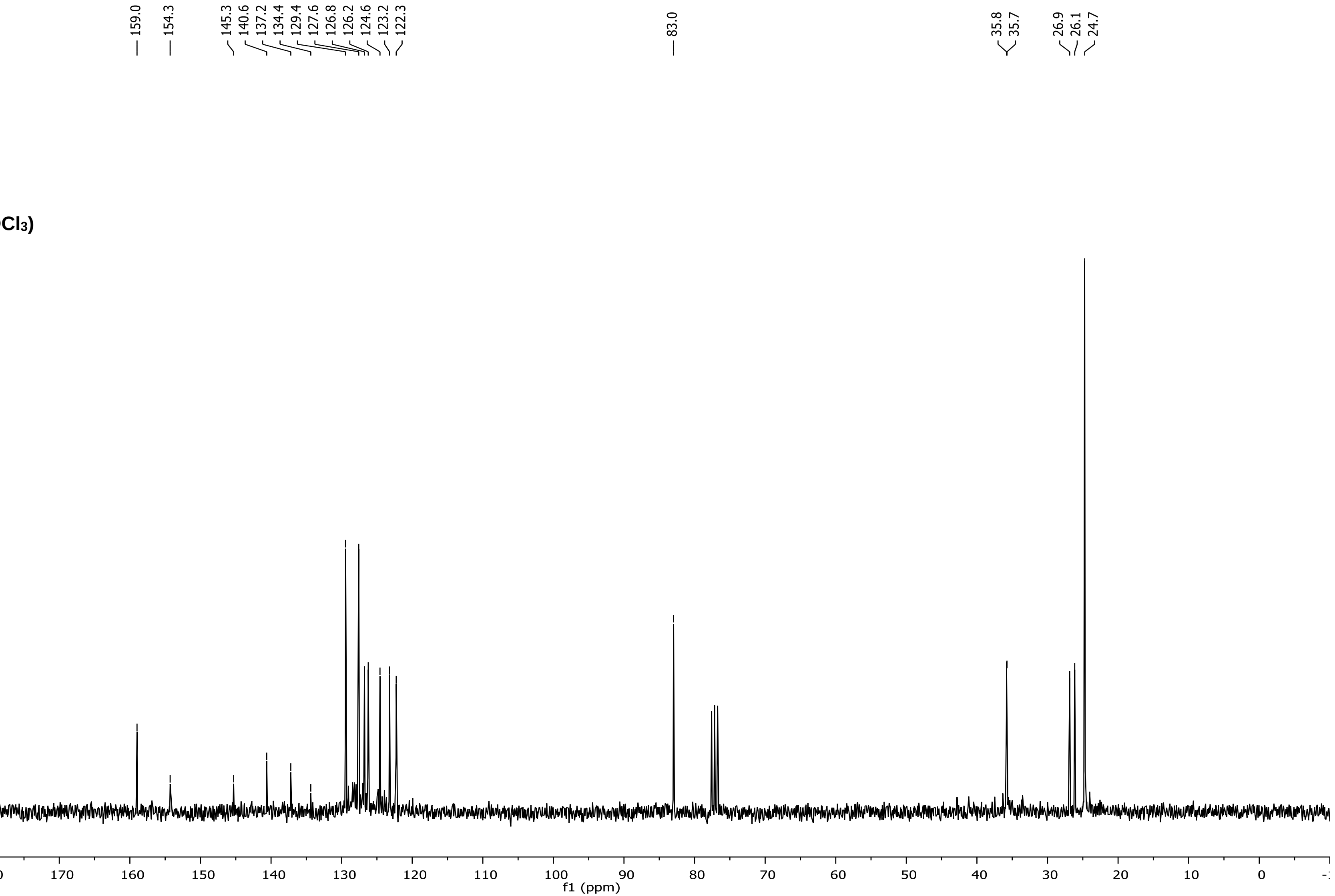

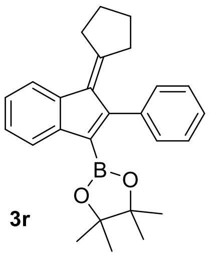

3r

^{11}B-NMR (96 MHz, $CDCl_3$)

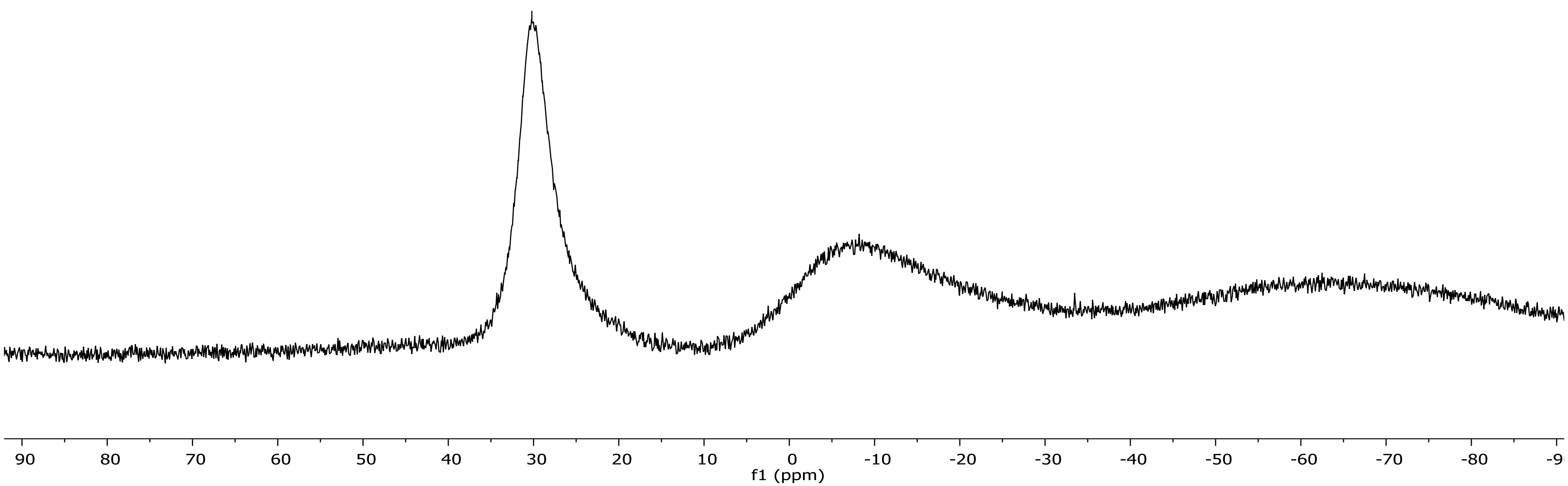

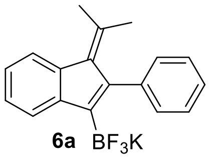

6a

¹H-NMR (400 MHz, Acetone-d₆)

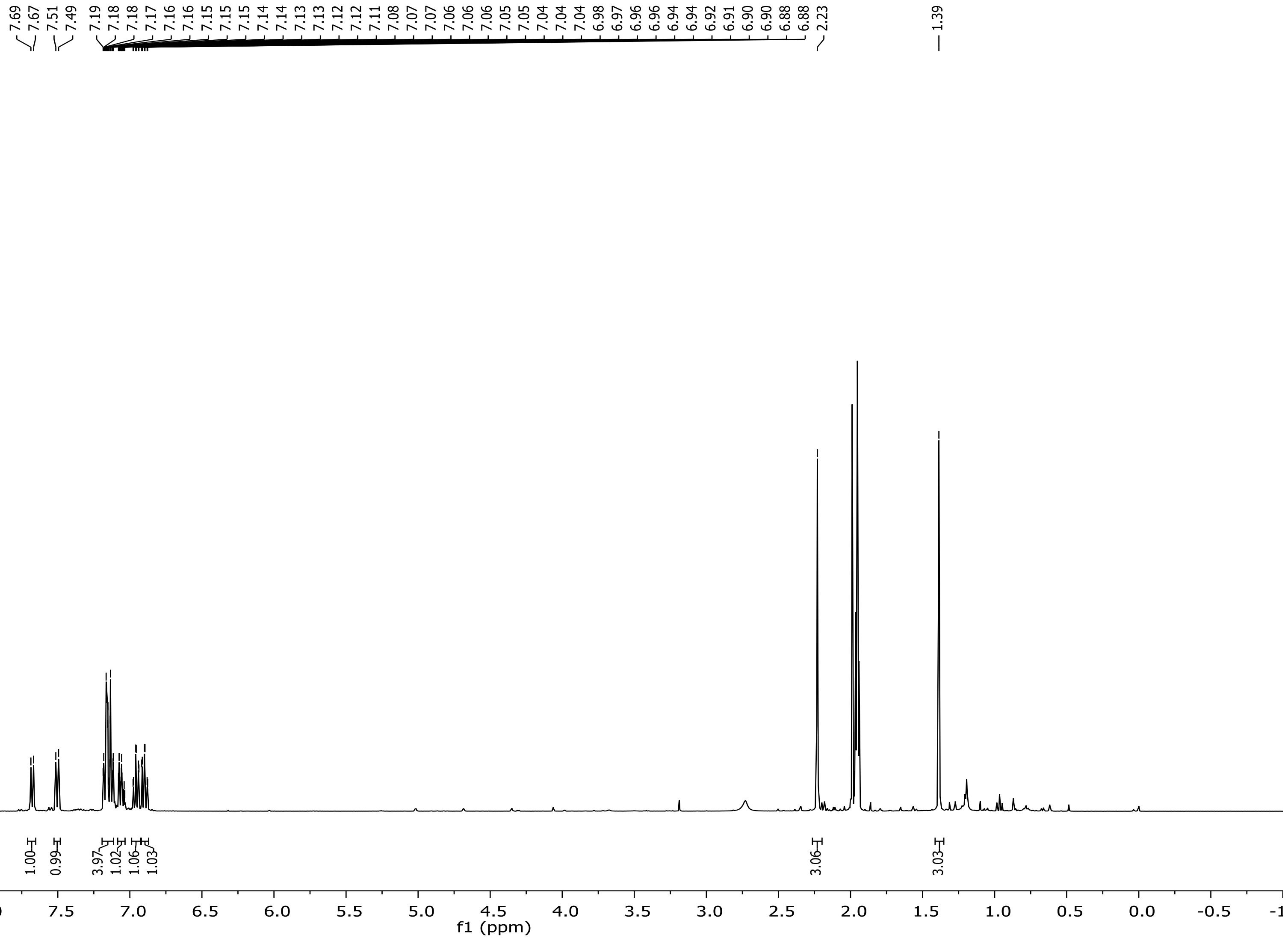

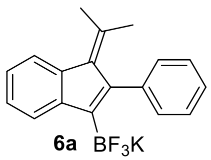

6a

^{13}C-NMR (100 MHz, Acetone-d6)

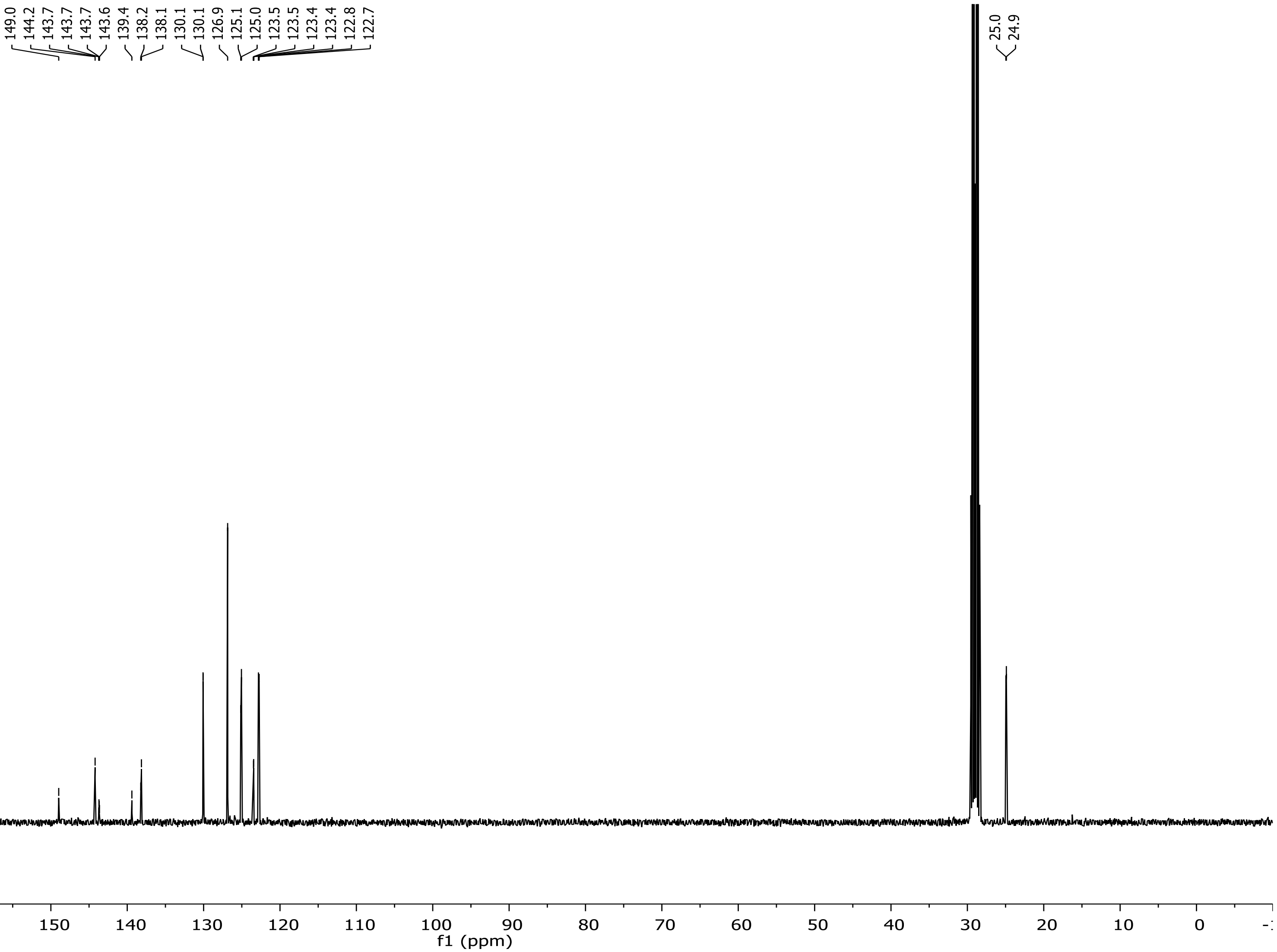

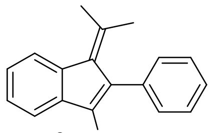

6a BF_3K

^{11}B-NMR (128 MHz, Acetone-d6)

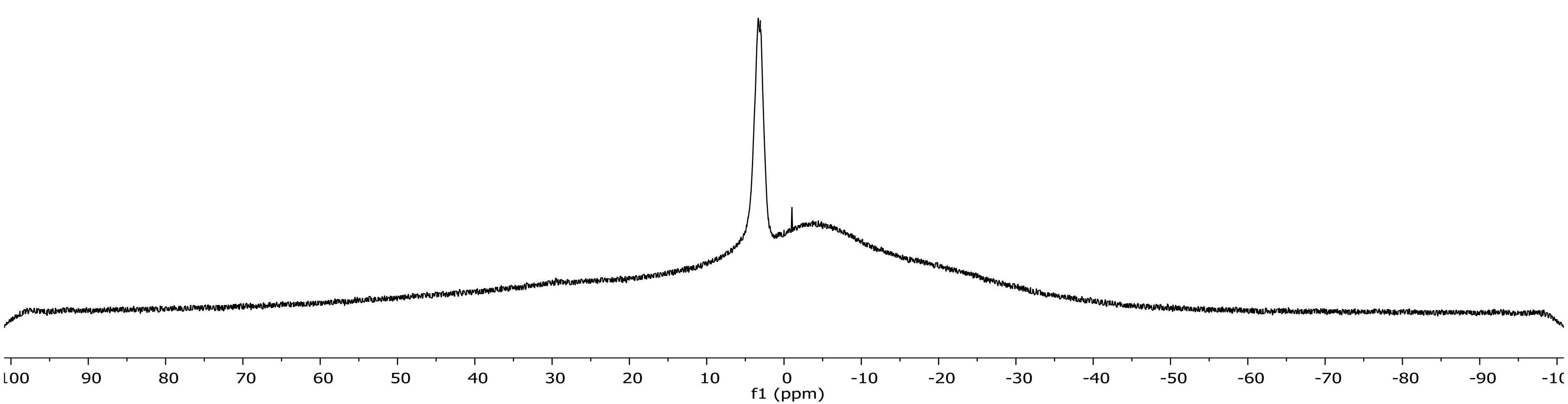

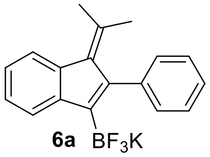

6a

^{19}F-NMR (376 MHz, Acetone-d$_6$)

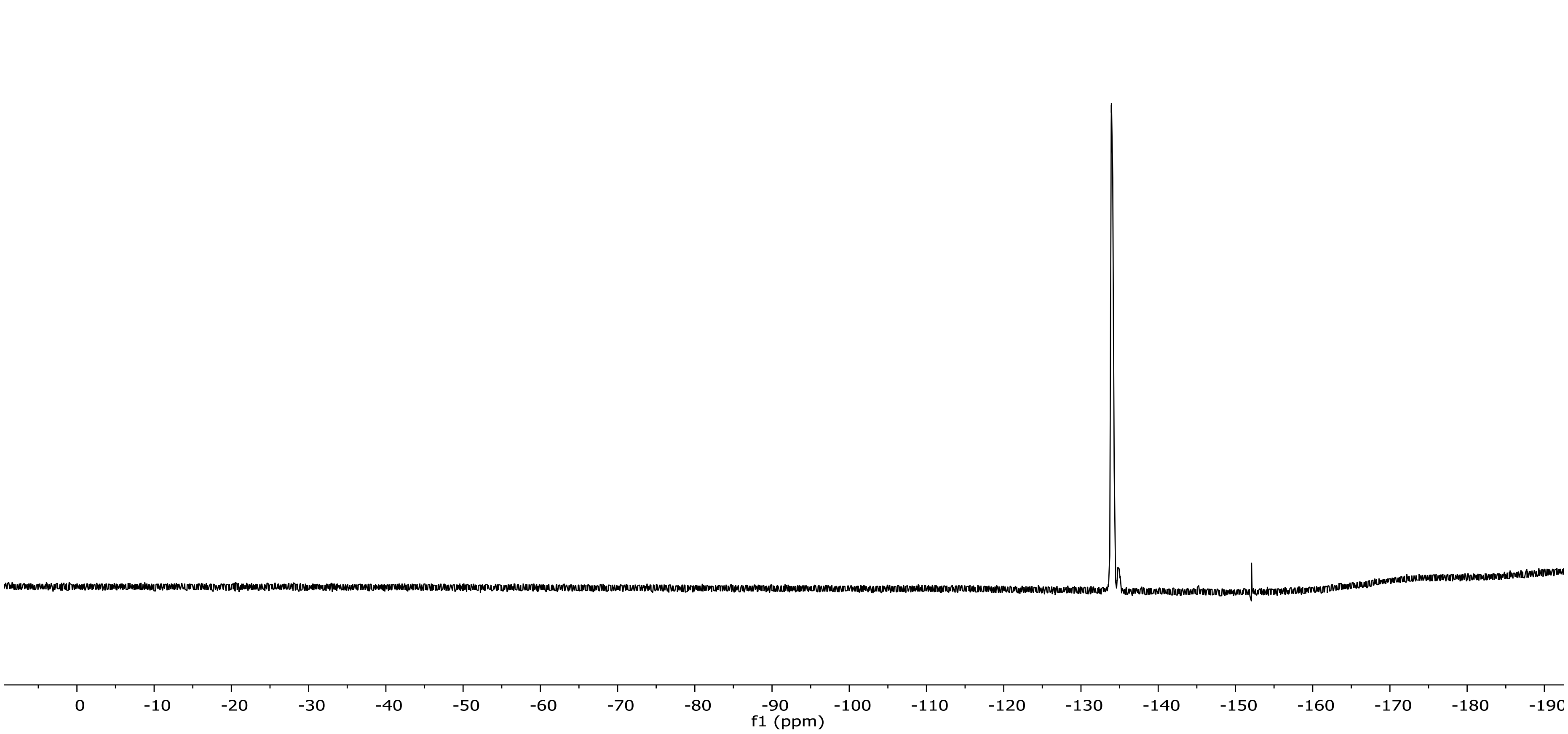

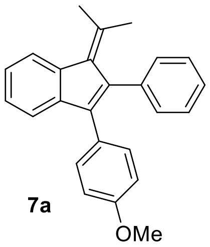

7a

^{1}H-NMR (400 MHz, CDCl3)

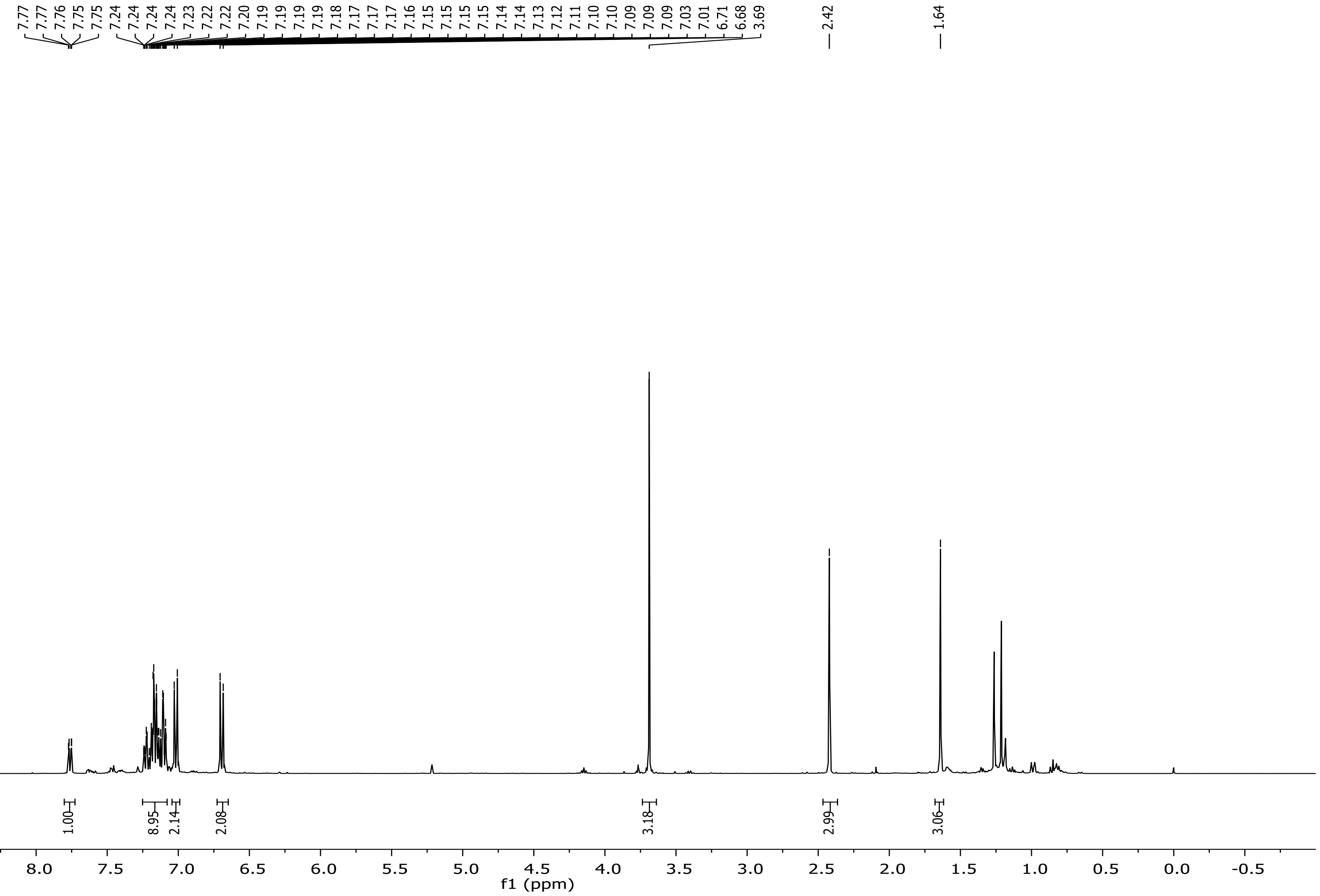

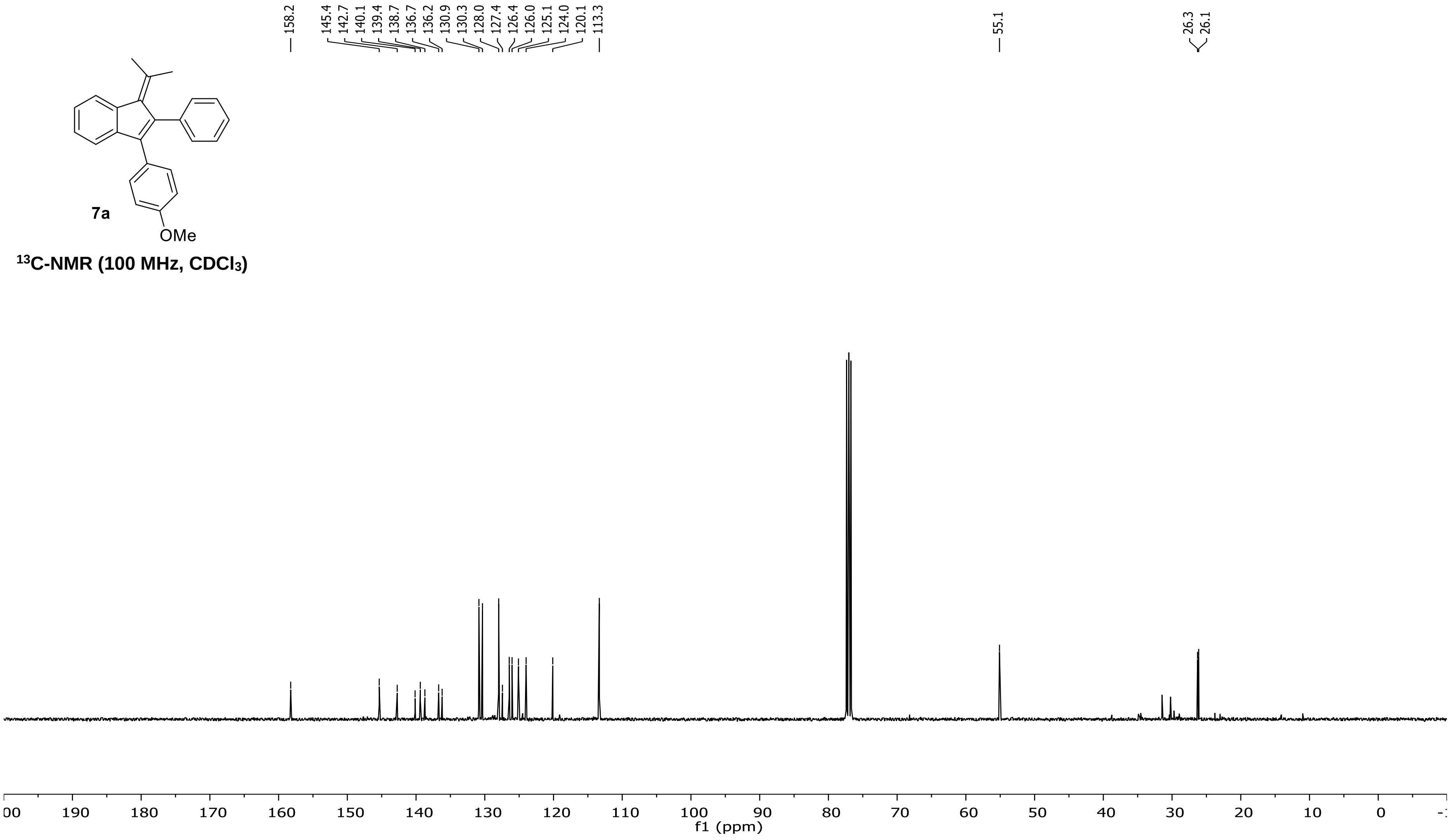

^{13}C-NMR (100 MHz, $CDCl_3$)

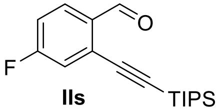

IIs

^{1}H-NMR (400 MHz, $CDCl_3$)

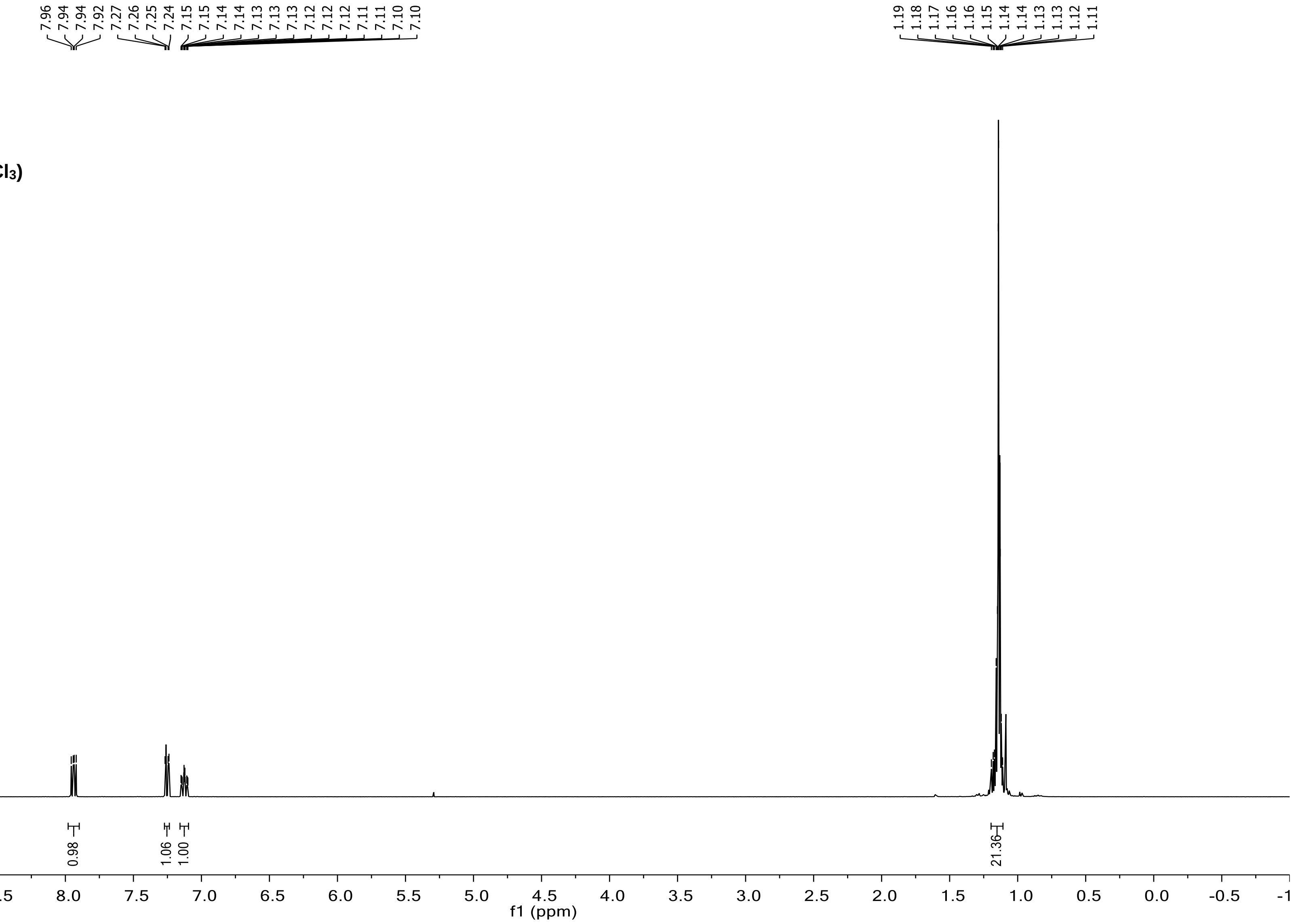

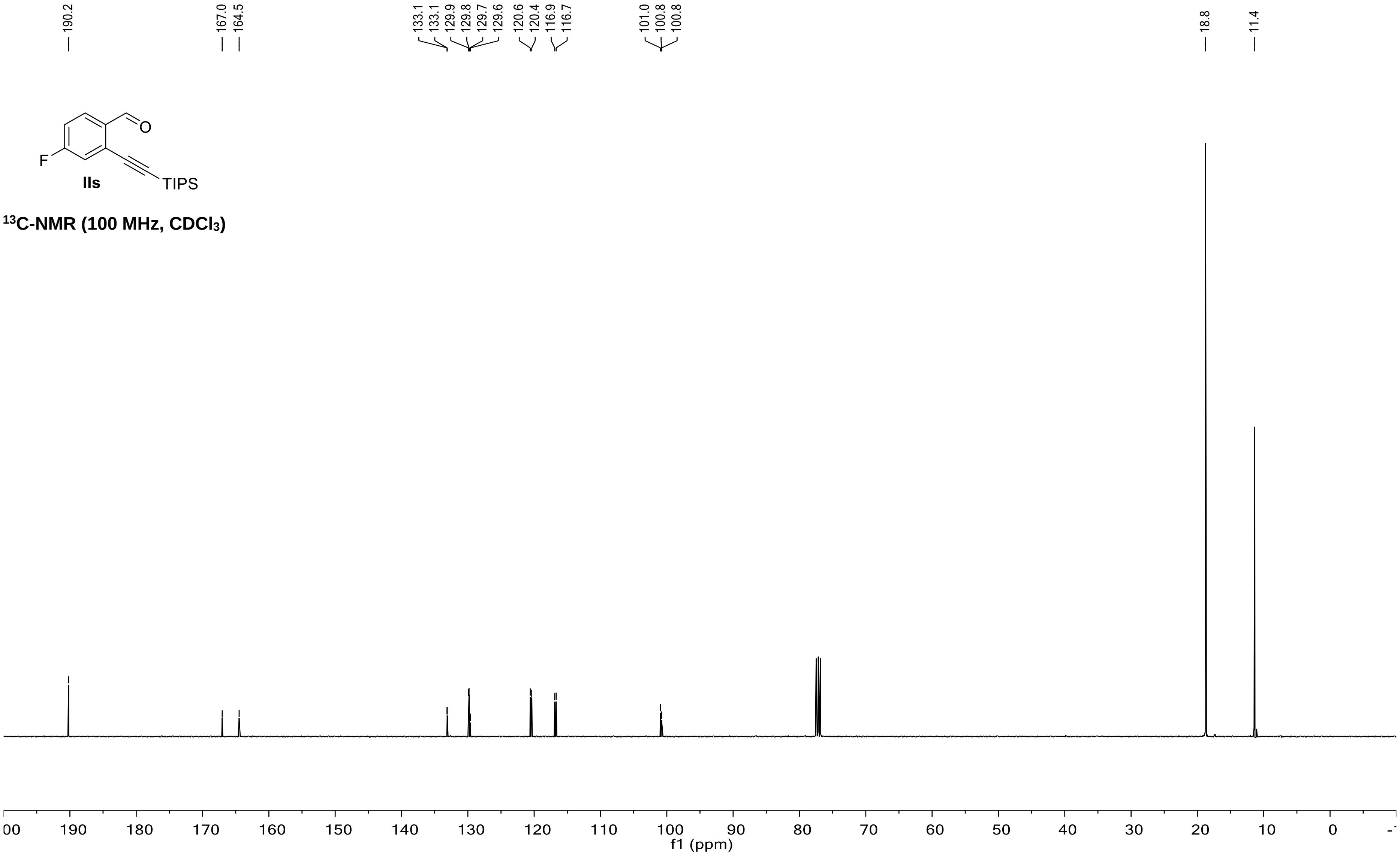

^{13}C-NMR (100 MHz, $CDCl_3$)

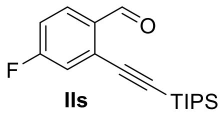

IIs

^{19}F-NMR (376 MHz, CDCl$_3$)

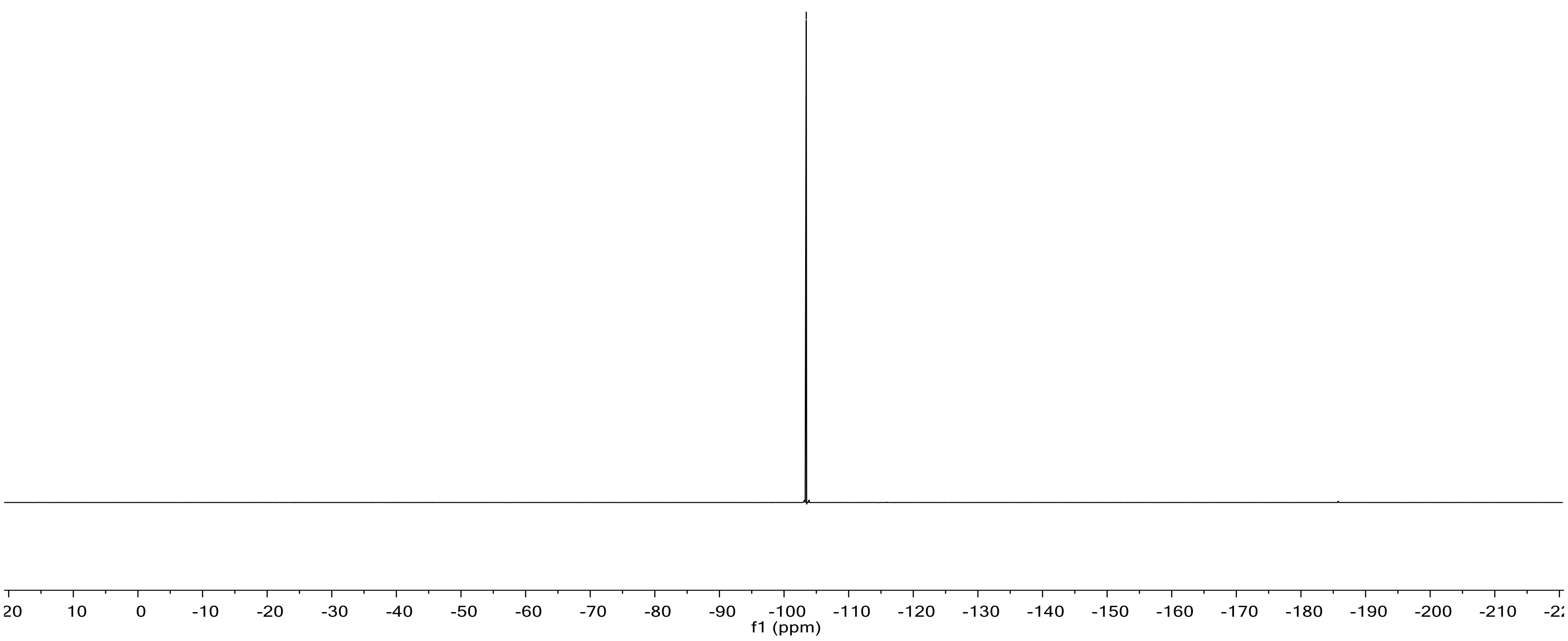

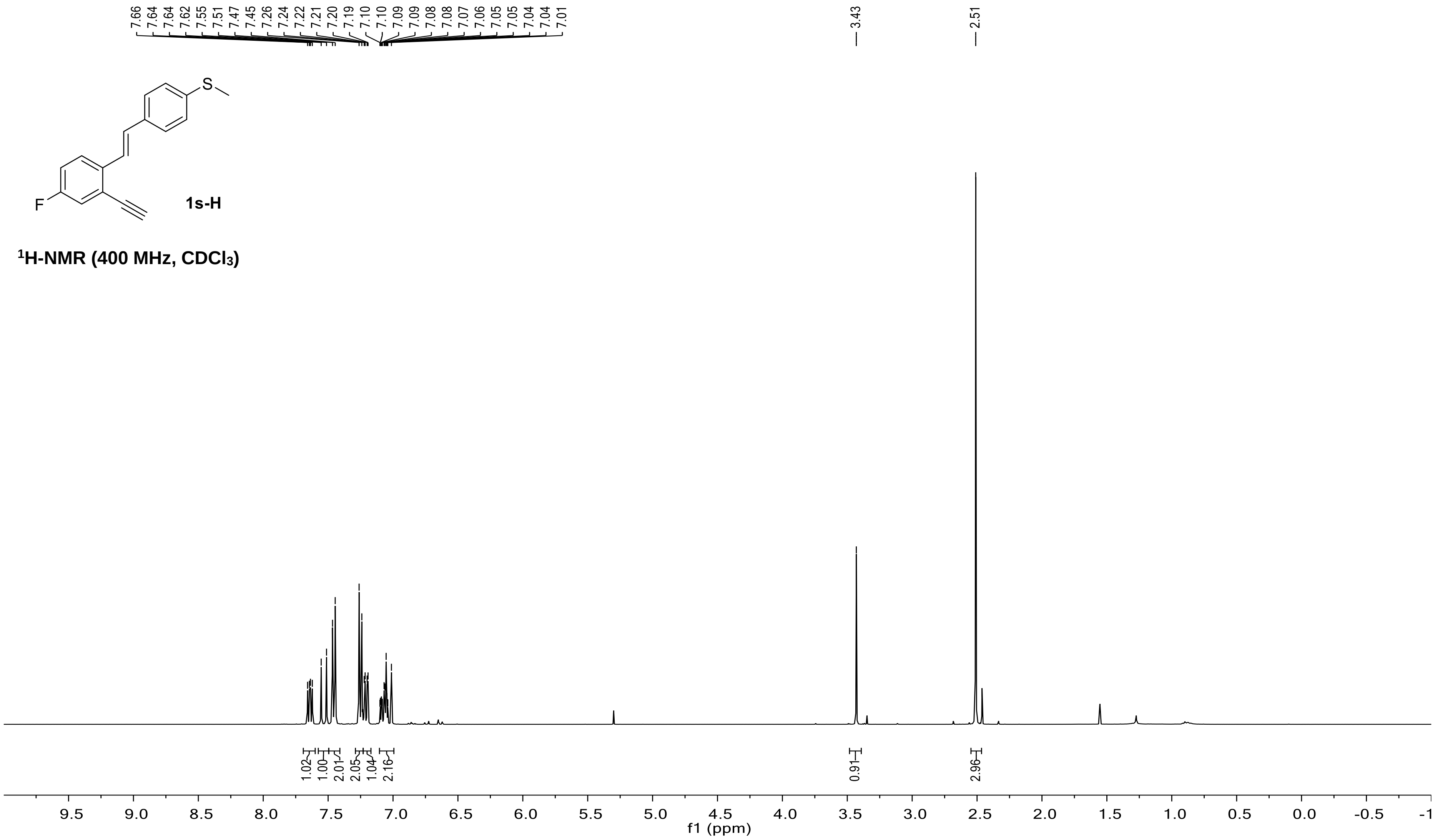
7.66 7.64 7.64 7.62 7.55 7.51 7.47 7.45 7.26 7.24 7.22 7.21 7.20 7.19 7.10 7.10 7.09 7.09 7.08 7.08 7.07 7.06 7.05 7.05 7.04 7.04 7.01
3.43
2.51
S
F
1s-H
¹H-NMR (400 MHz, CDCl₃)
1.02 1.00 2.01 2.05 1.04 2.16
0.91
2.96
9.5 9.0 8.5 8.0 7.5 7.0 6.5 6.0 5.5 5.0 4.5 4.0 3.5 3.0 2.5 2.0 1.5 1.0 0.5 0.0 -0.5 -1
f1 (ppm)

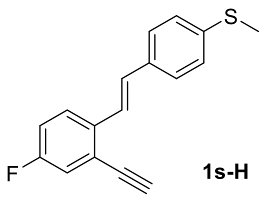

1s-H

^{13}C-NMR (100 MHz, CDCl$_3$)

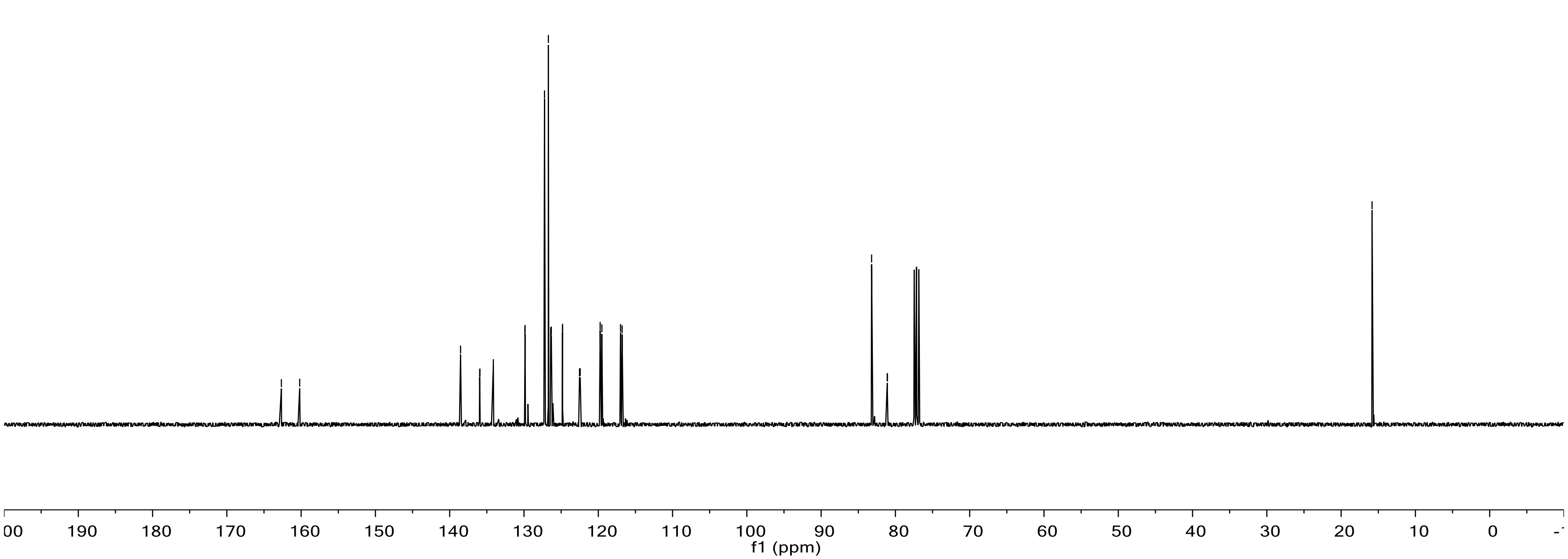

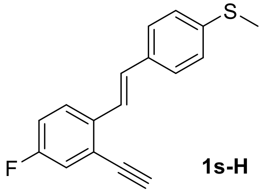

1s-H

^{19}F-NMR (376 MHz, CDCl$_3$)

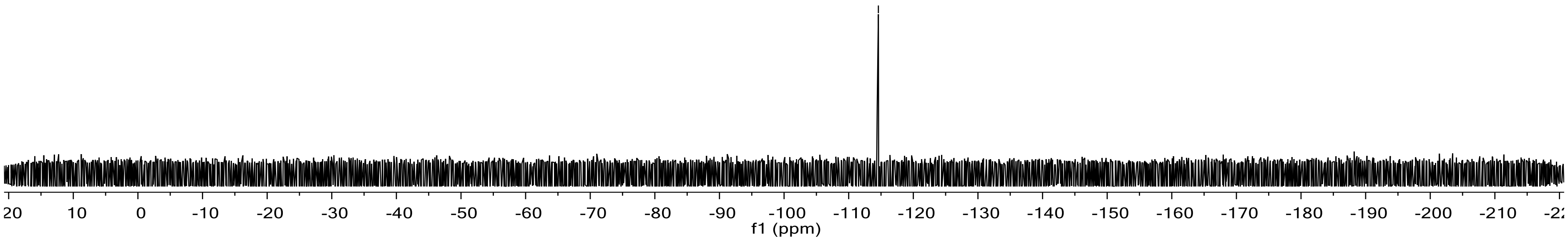

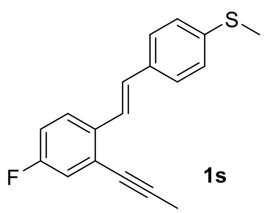

1s

^{1}H-NMR (400 MHz, $CDCl_3$)

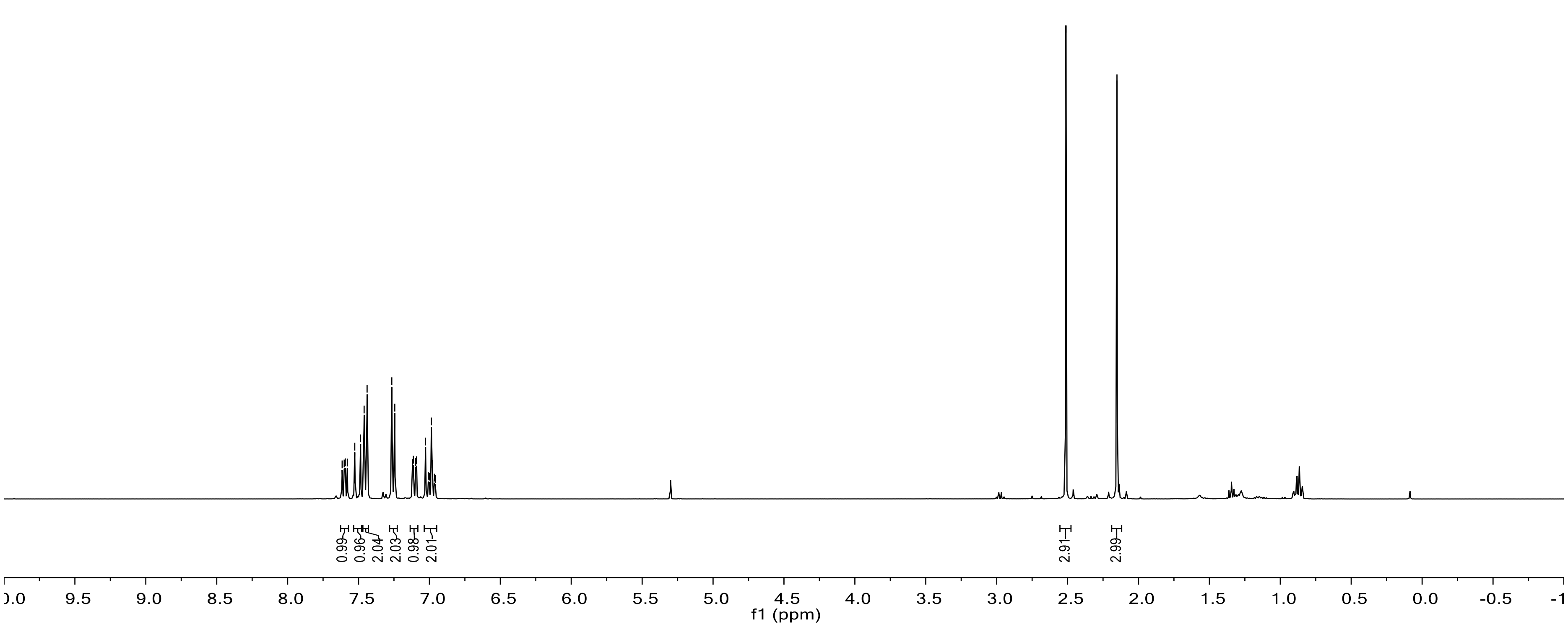

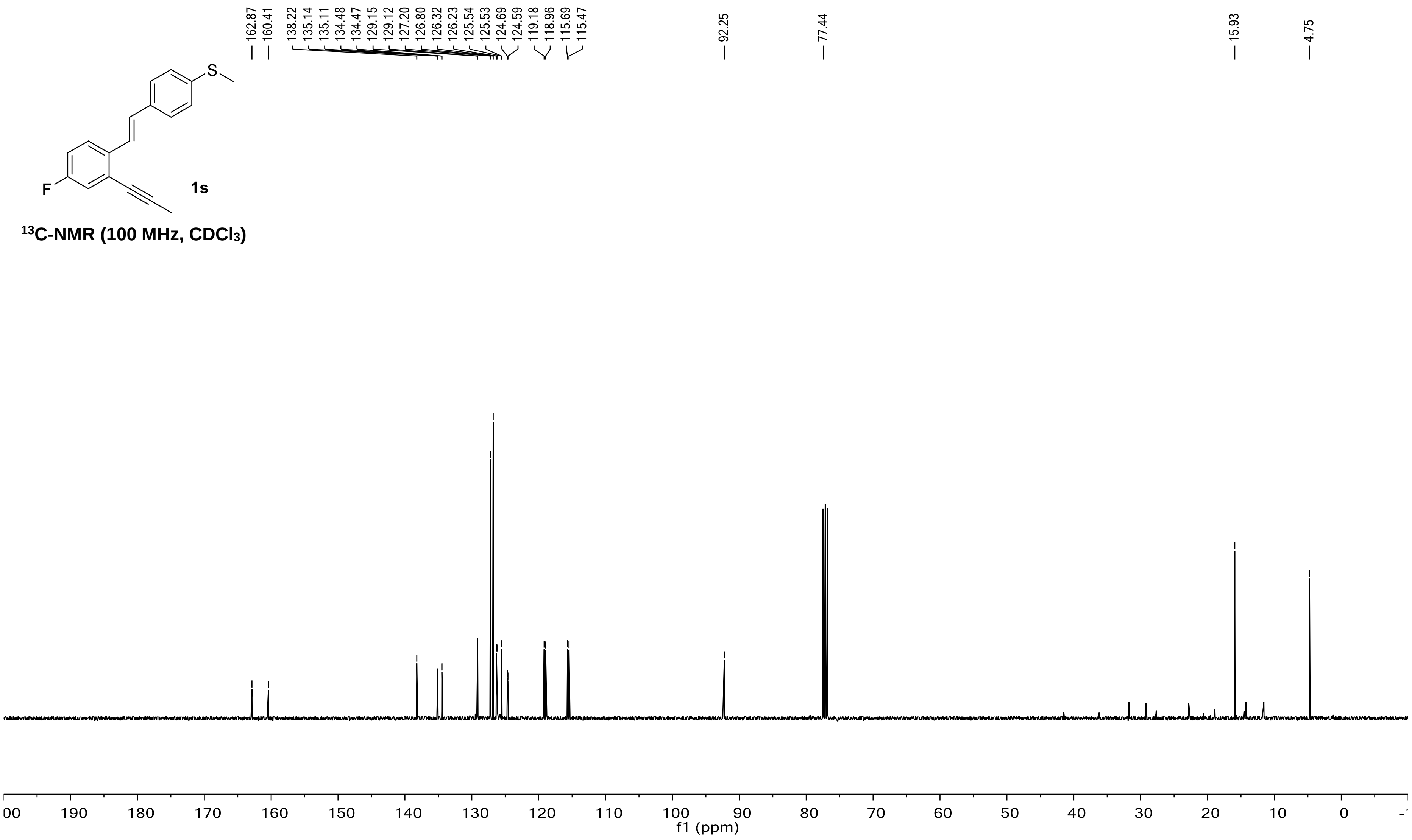

^{13}C-NMR (100 MHz, $CDCl_3$)

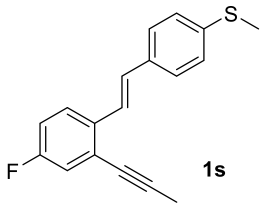

1s

^{19}F-NMR (376 MHz, $CDCl_3$)

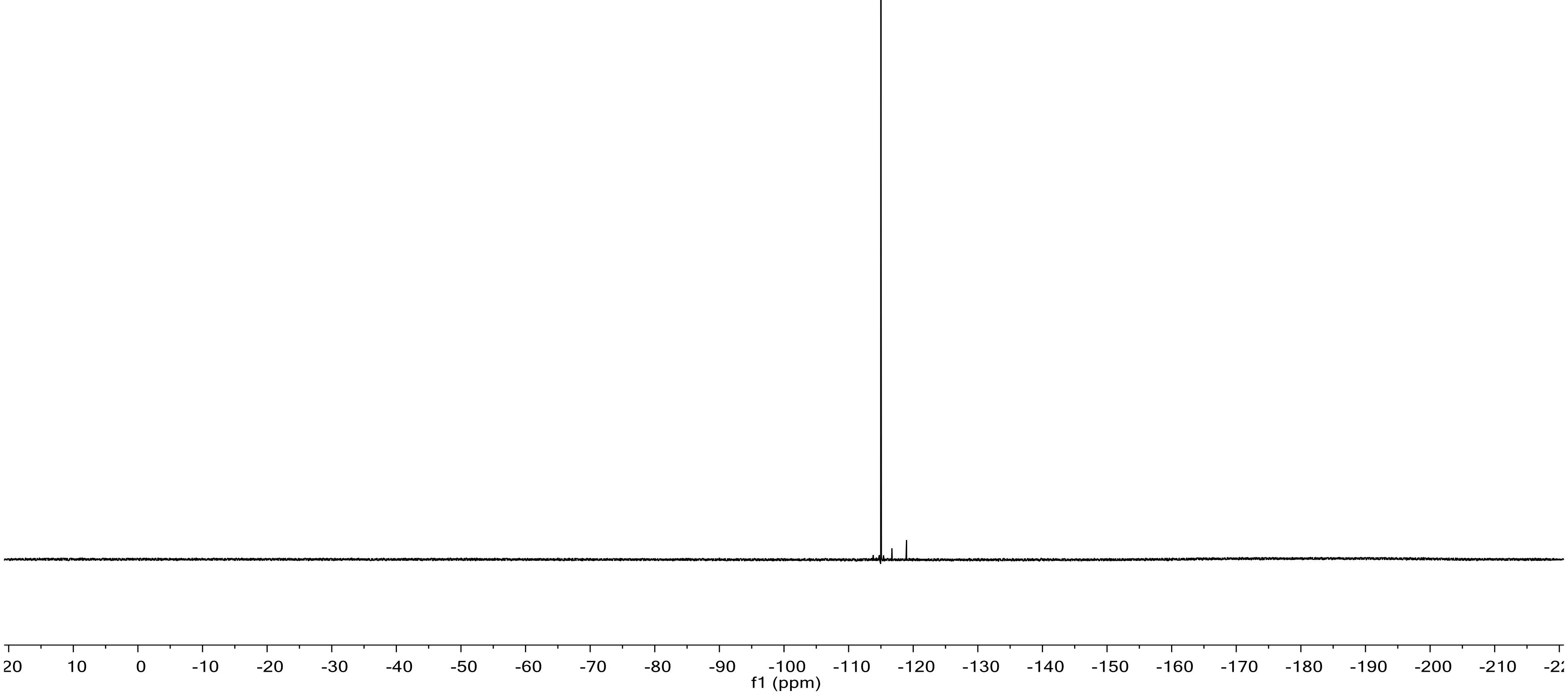

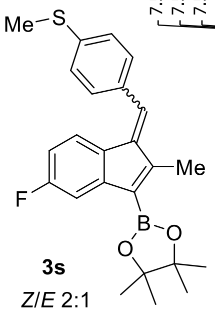

3s

Z/*E* 2:1

^{1}H-NMR (400 MHz, CDCl$_3$)

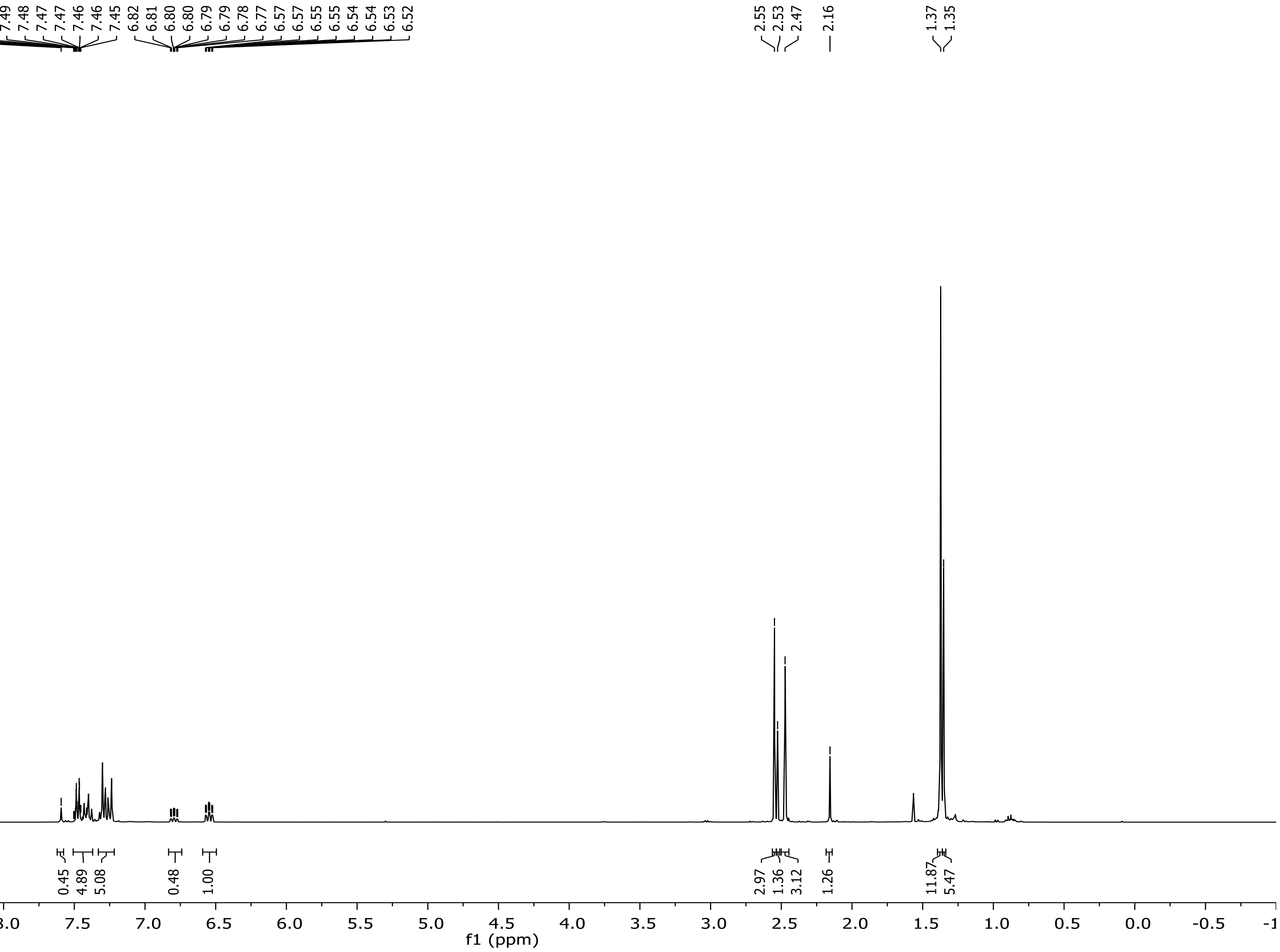

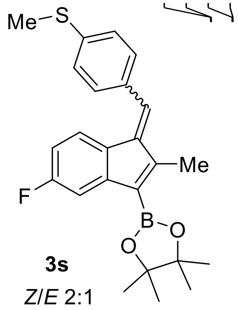

3s

Z/*E* 2:1

^{13}C-NMR (100 MHz, $CDCl_3$)

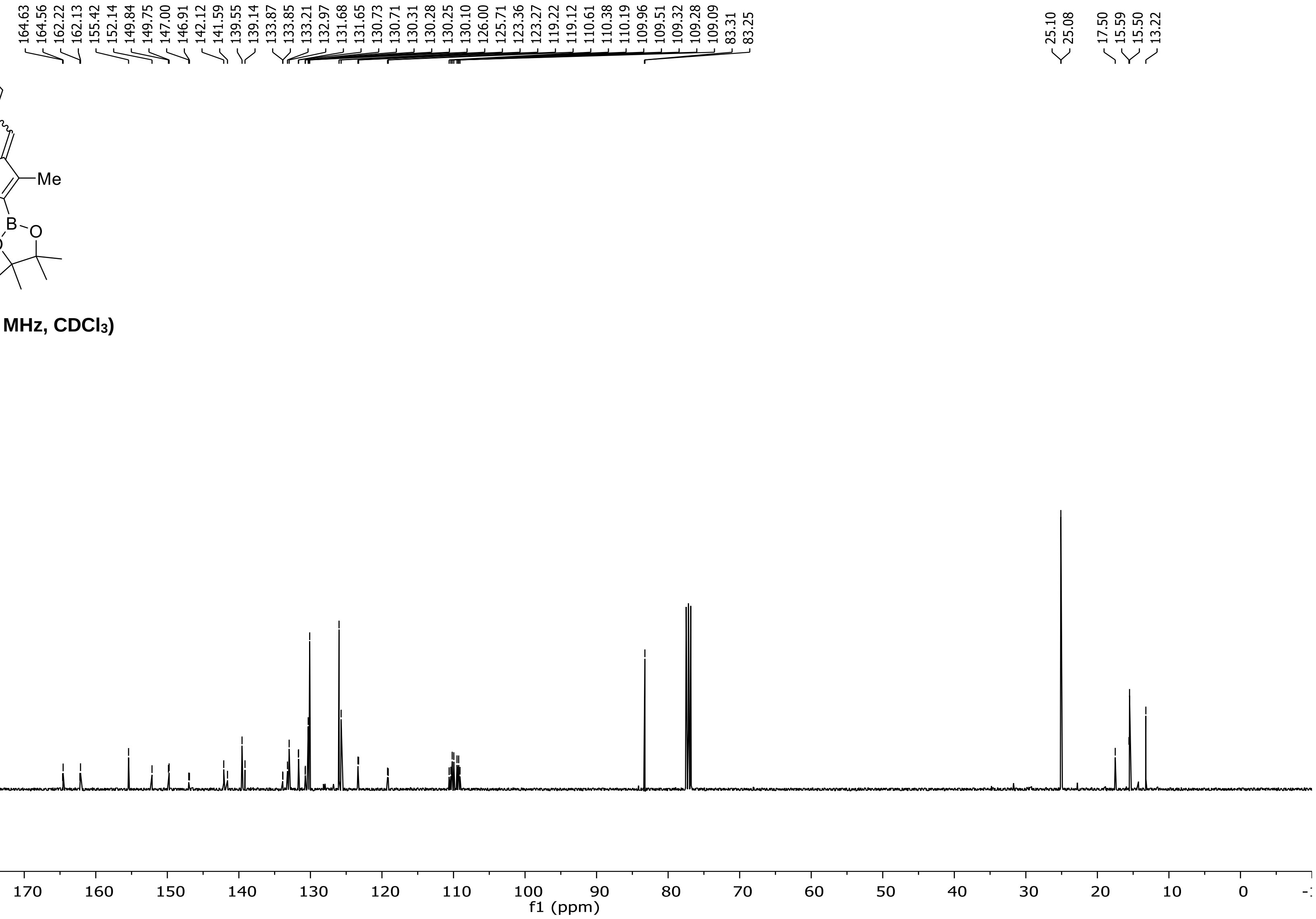

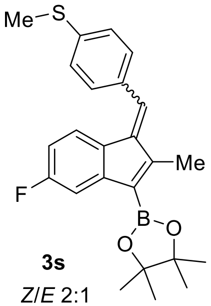

3s

Z/*E* 2:1

^{11}B-NMR (128 MHz, CDCl$_3$)

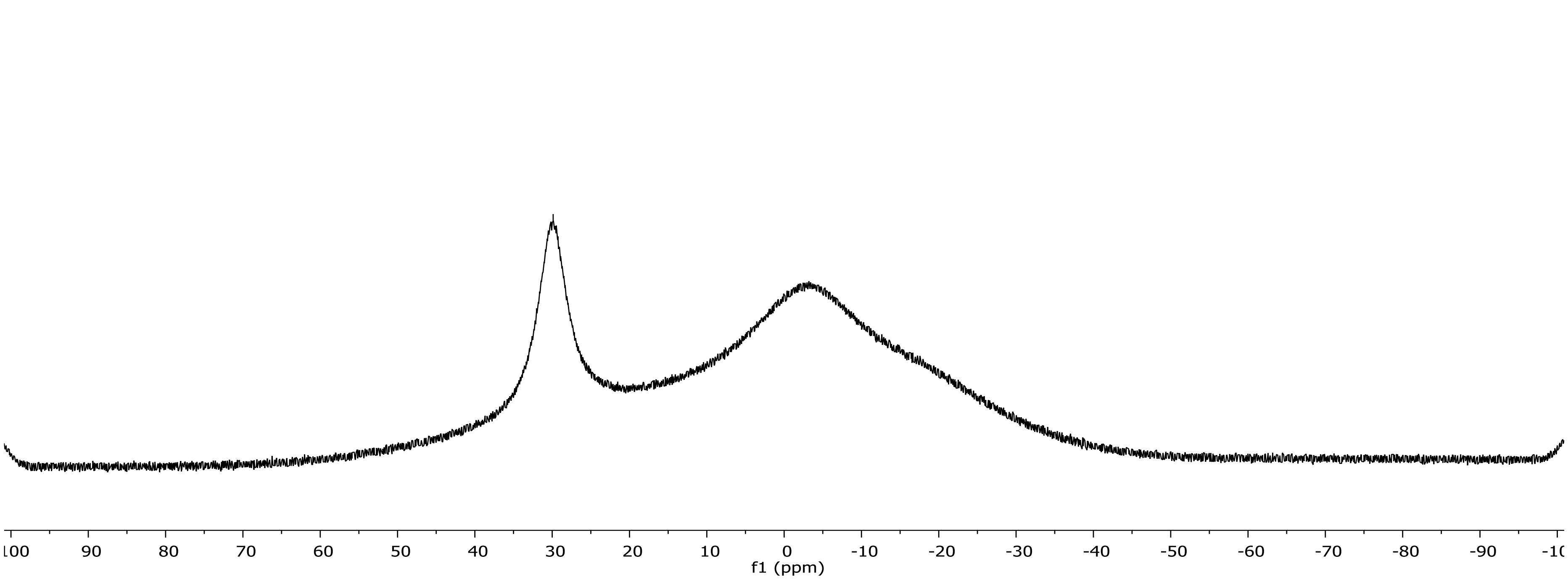

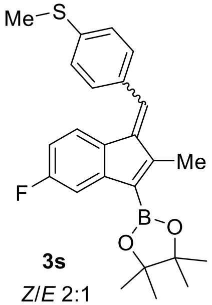

3s

Z/*E* 2:1

^{19}F-NMR (376 MHz, CDCl$_3$)

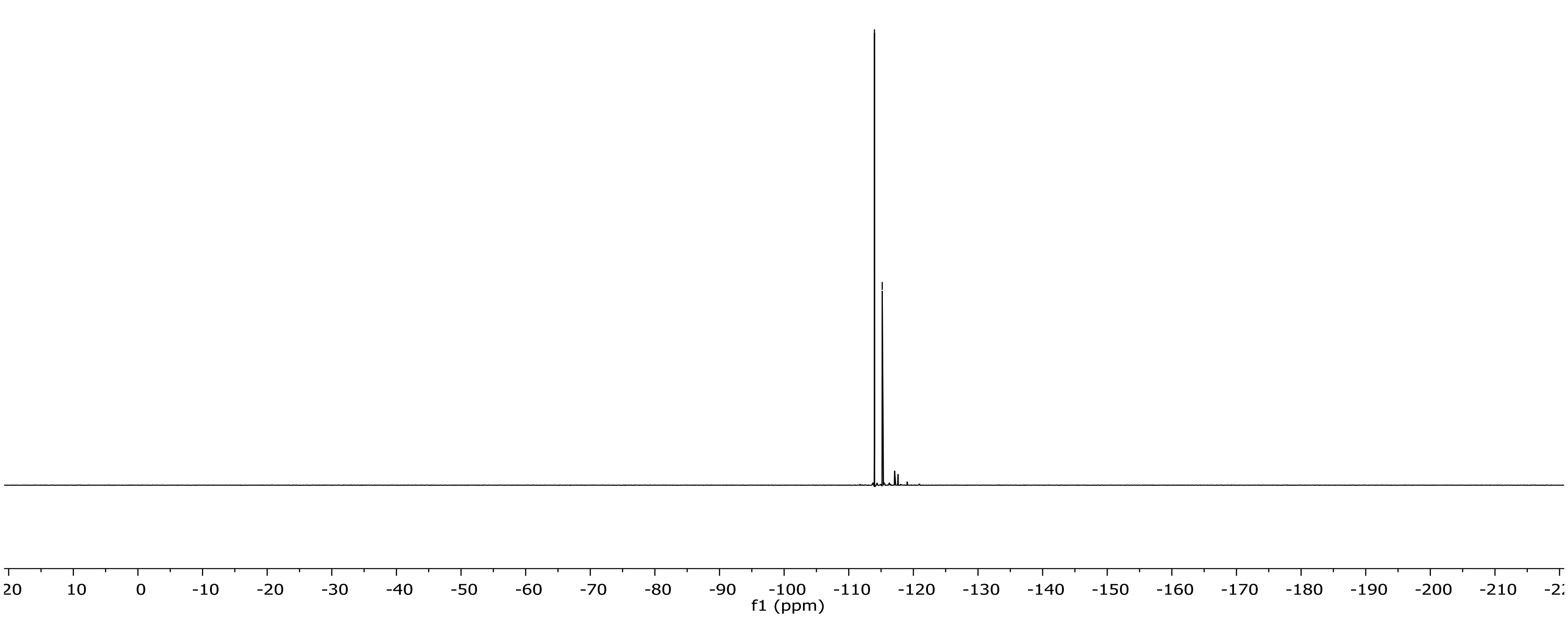

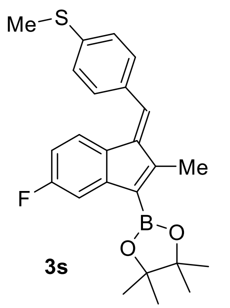

3s

^{1}H-NMR (400 MHz, $CDCl_3$)

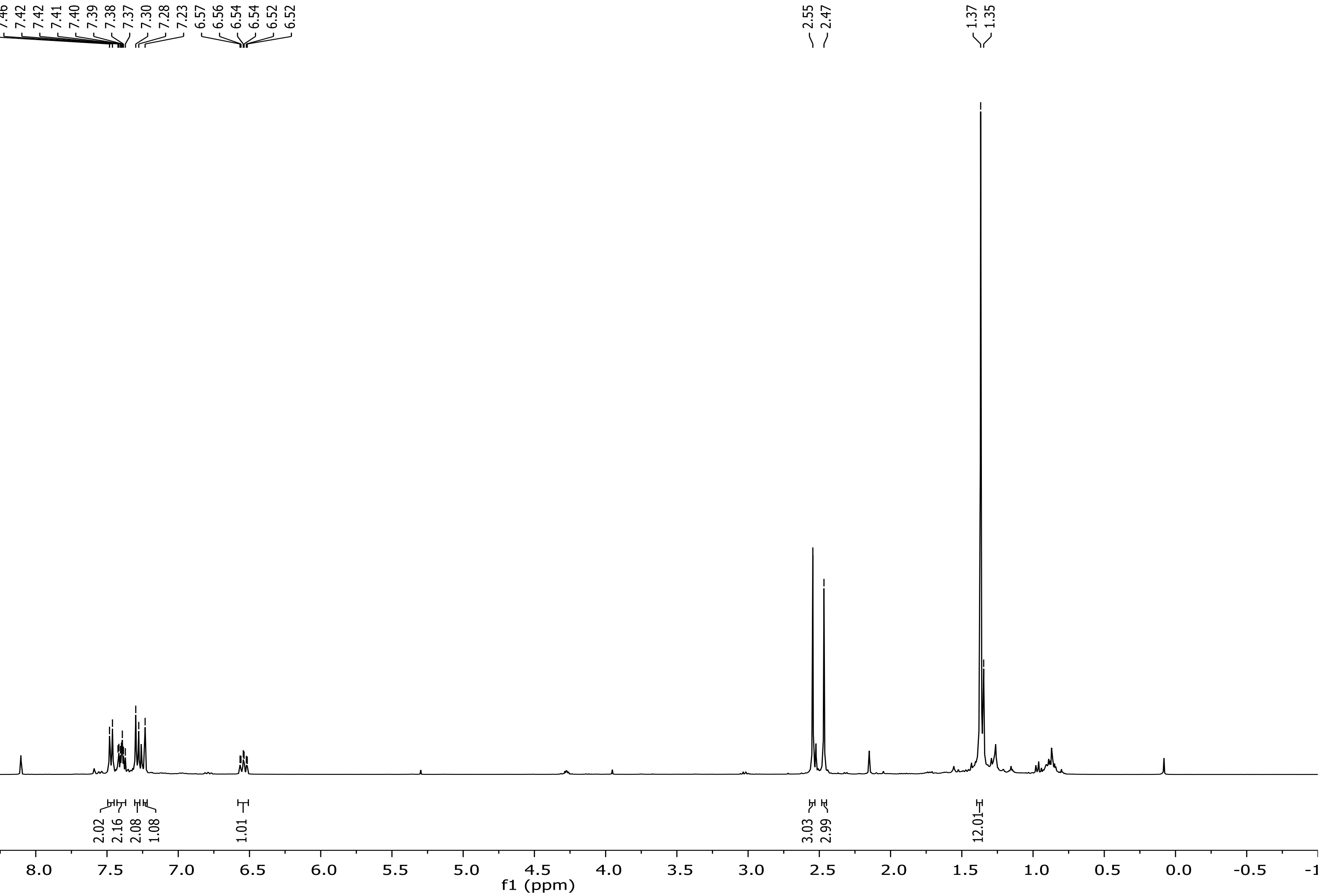

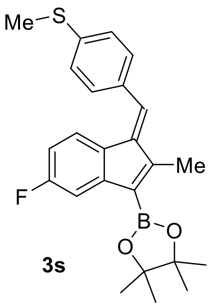

3s

^{13}C-NMR (100 MHz, CDCl$_3$)

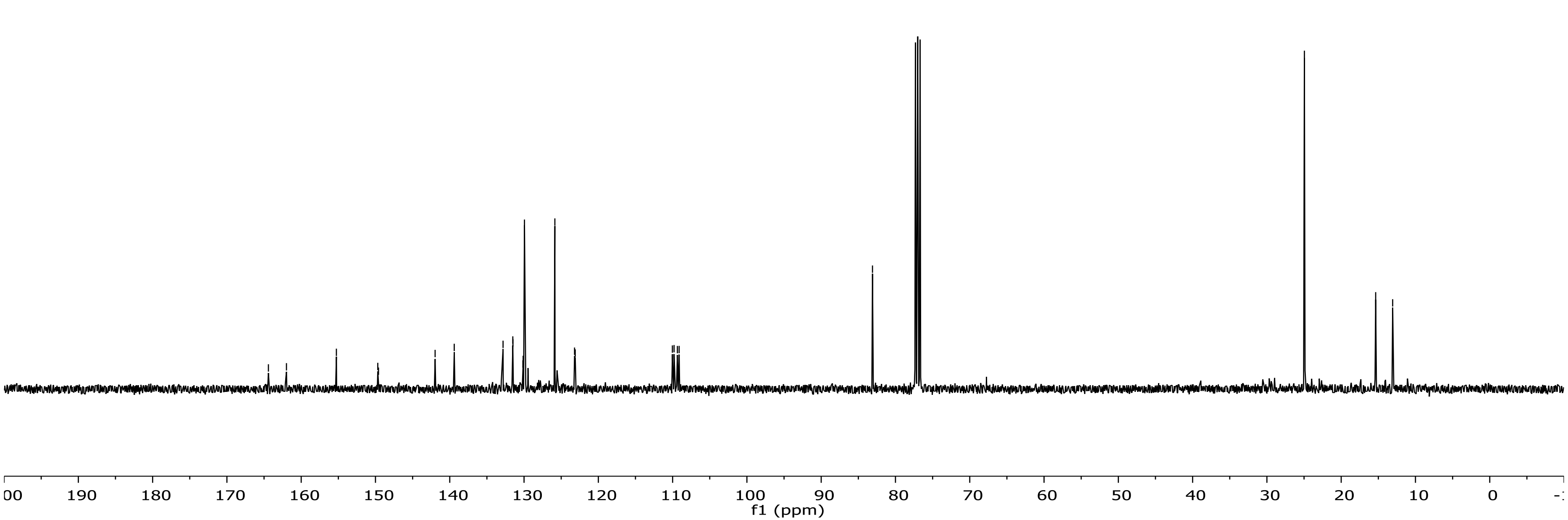

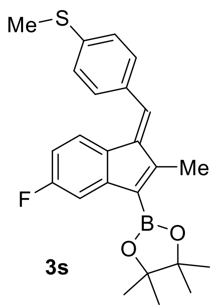

3s

^{11}B-NMR (128 MHz, CDCl$_3$)

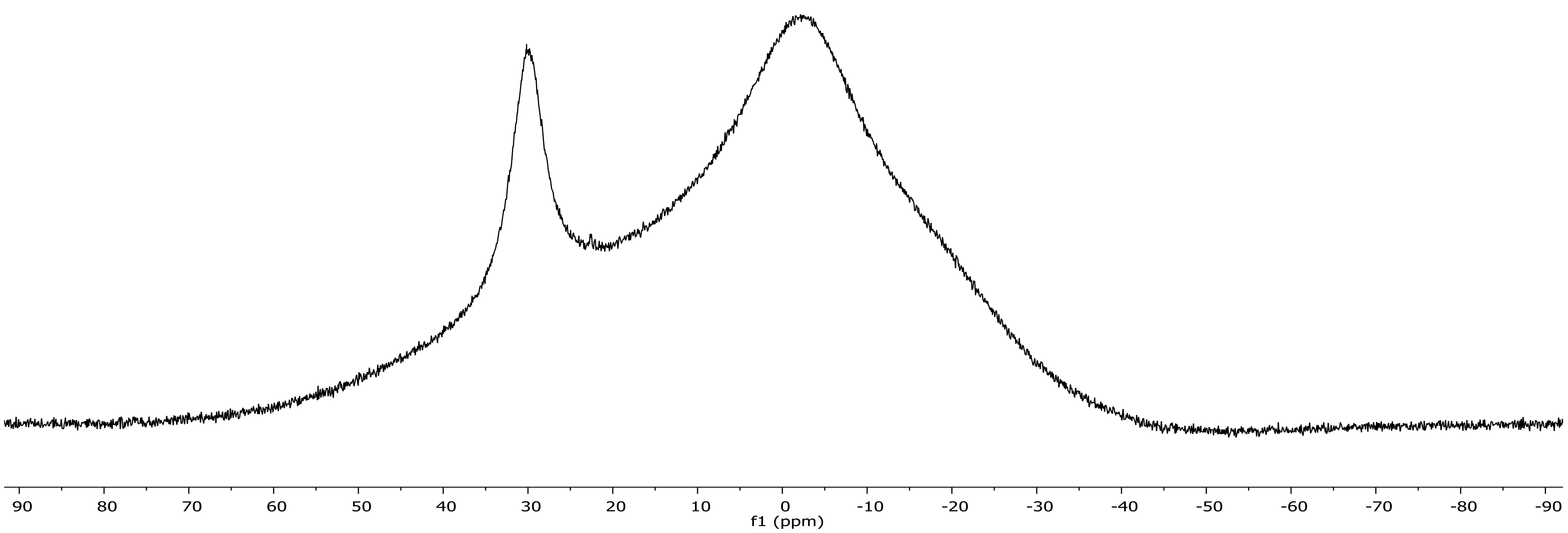

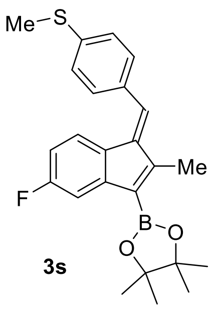

3s

^{19}F-NMR (376 MHz, $CDCl_3$)

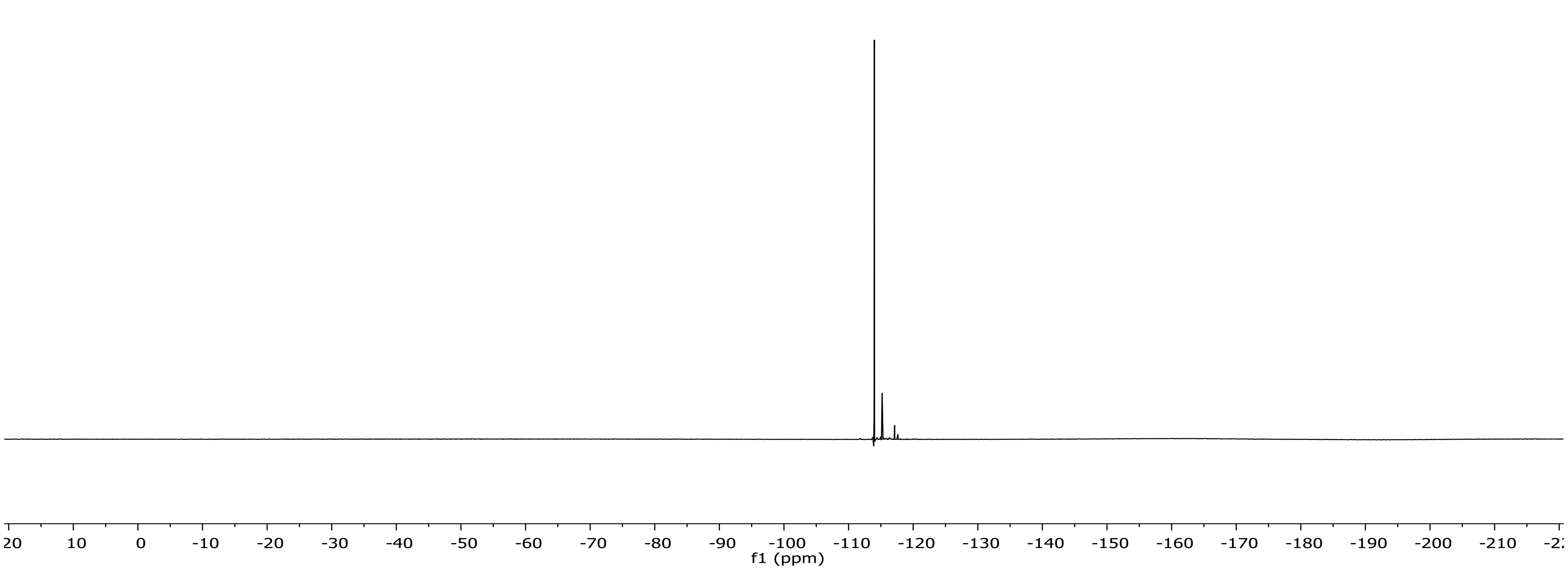

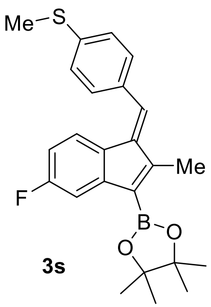

3s

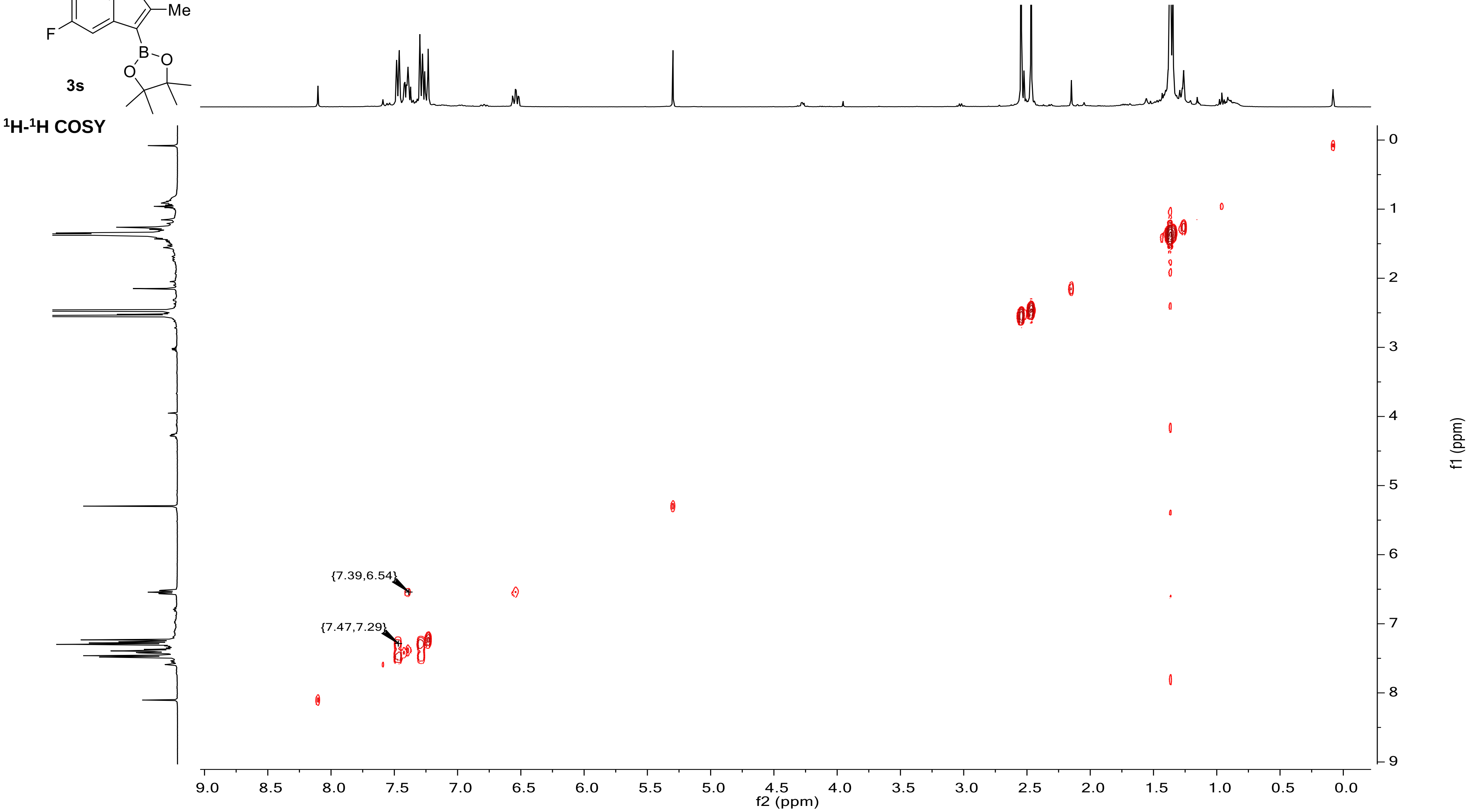

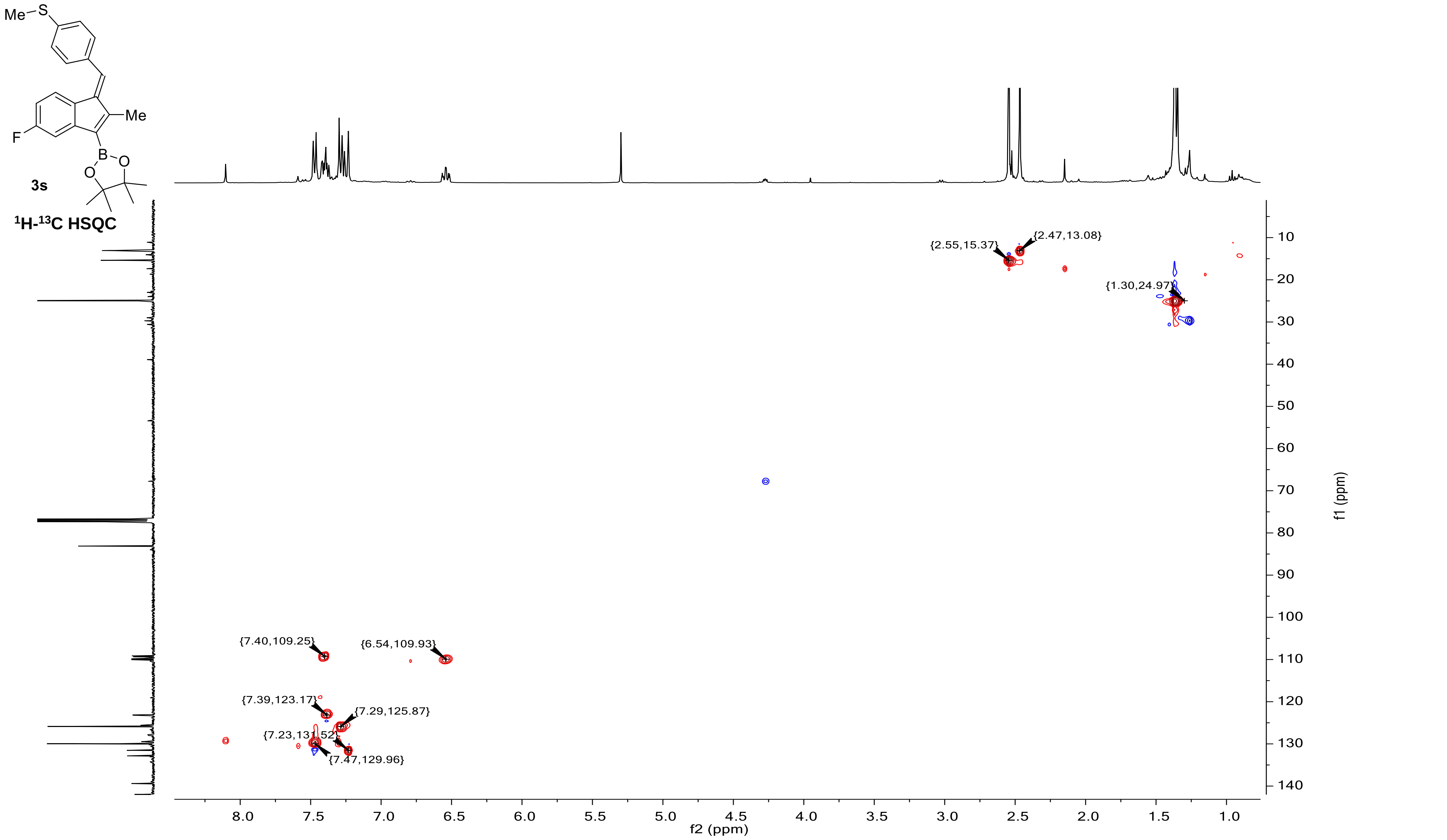

Me
S
Me
F
B
O
O
3s
^{1}H-^{13}C HSQC
{2.55,15.37}
{2.47,13.08}
{1.30,24.97}
{7.40,109.25}
{6.54,109.93}
{7.39,123.17}
{7.29,125.87}
{7.23,131.52}
{7.47,129.96}
8.0 7.5 7.0 6.5 6.0 5.5 5.0 4.5 4.0 3.5 3.0 2.5 2.0 1.5 1.0
f2 (ppm)
10 20 30 40 50 60 70 80 90 100 110 120 130 140
f1 (ppm)

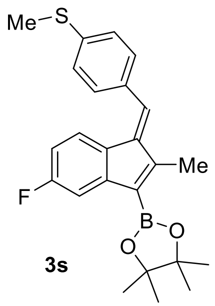

3s

¹H-¹³C HMBC

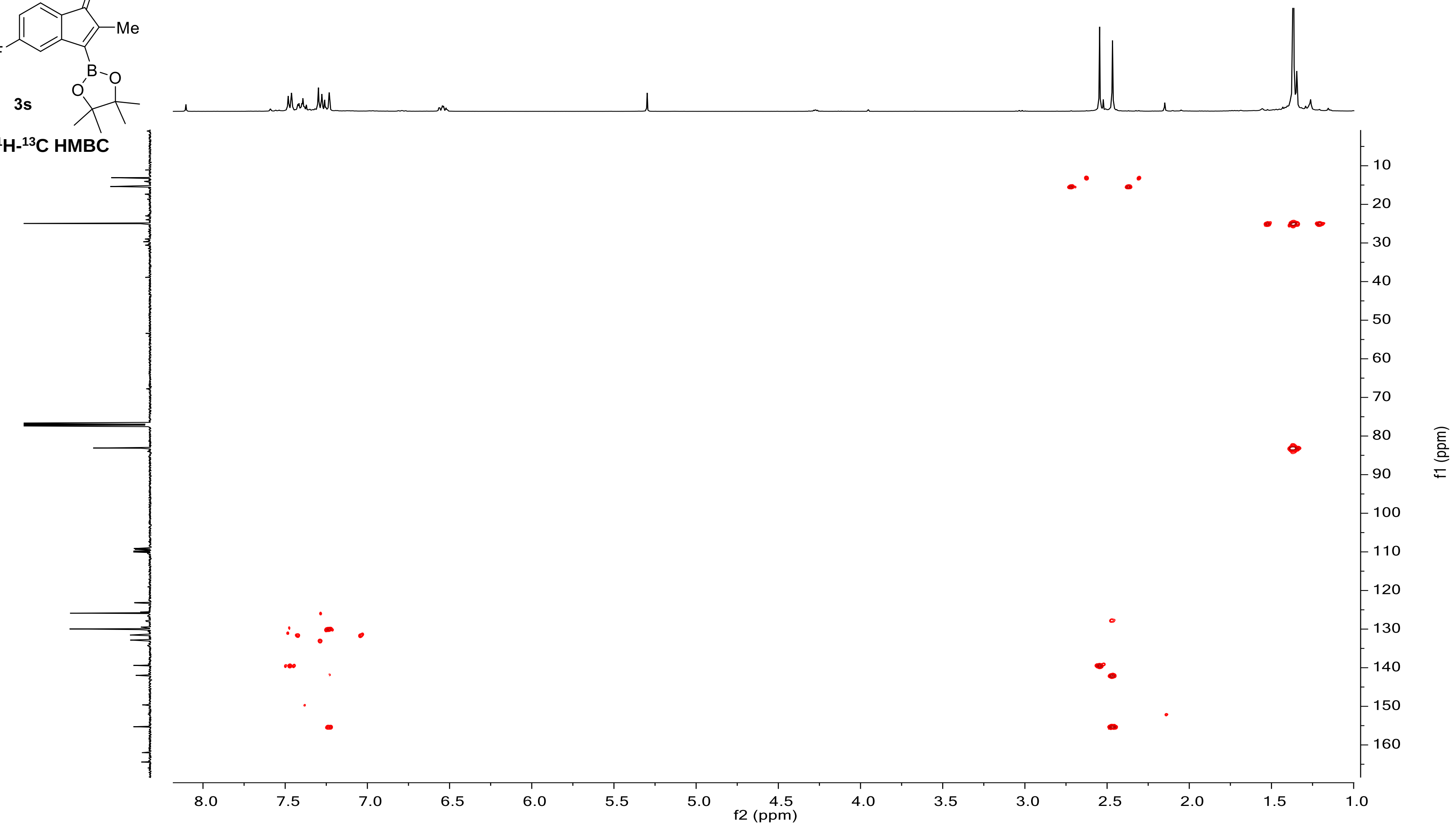

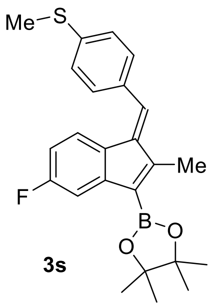

3s

^{1}H-^{1}H NOESY

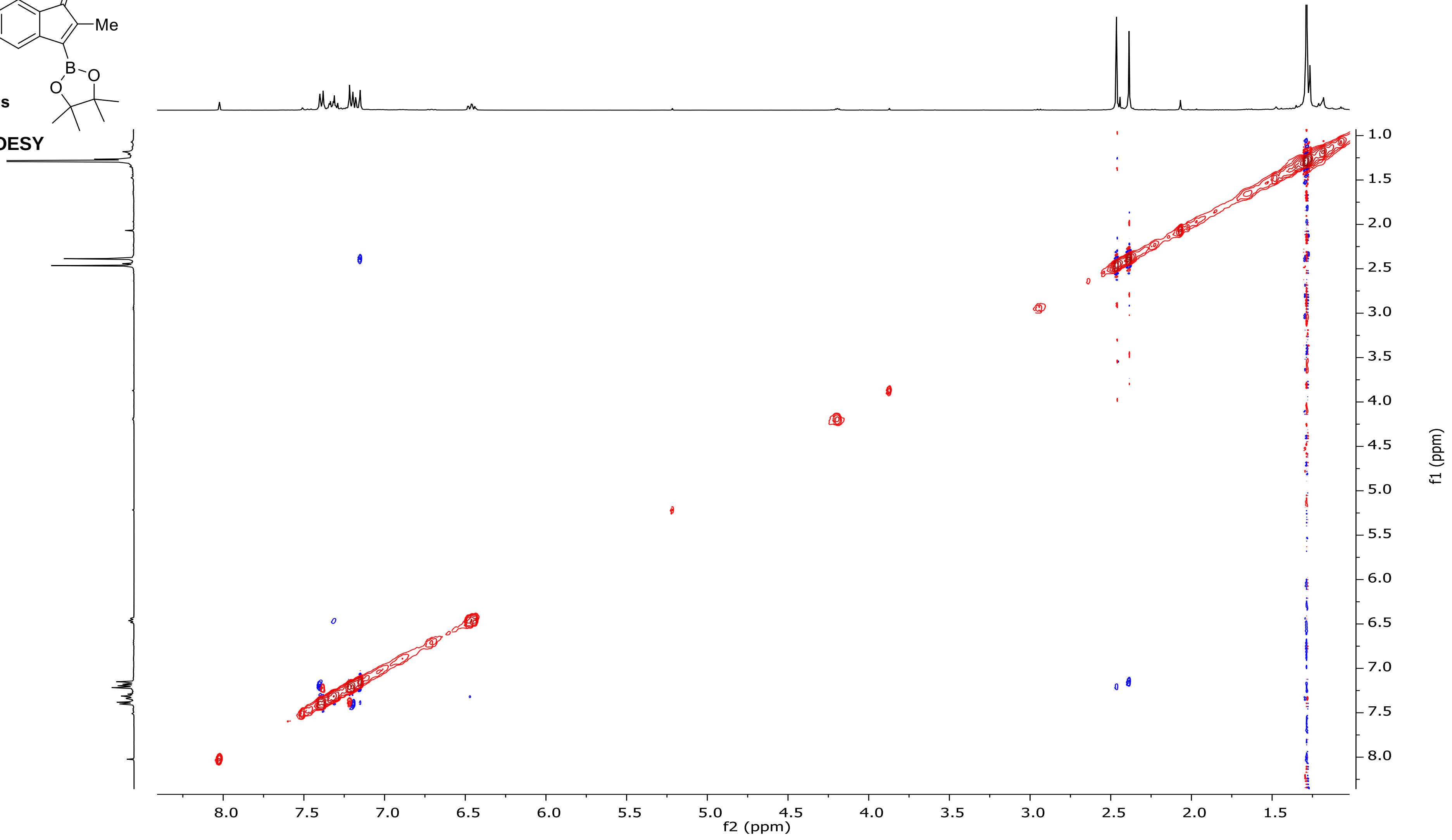

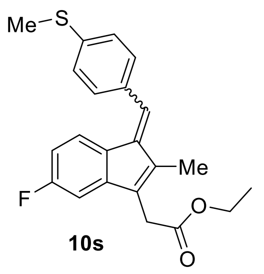

10s

^{1}H-NMR (400 MHz, $CDCl_3$)

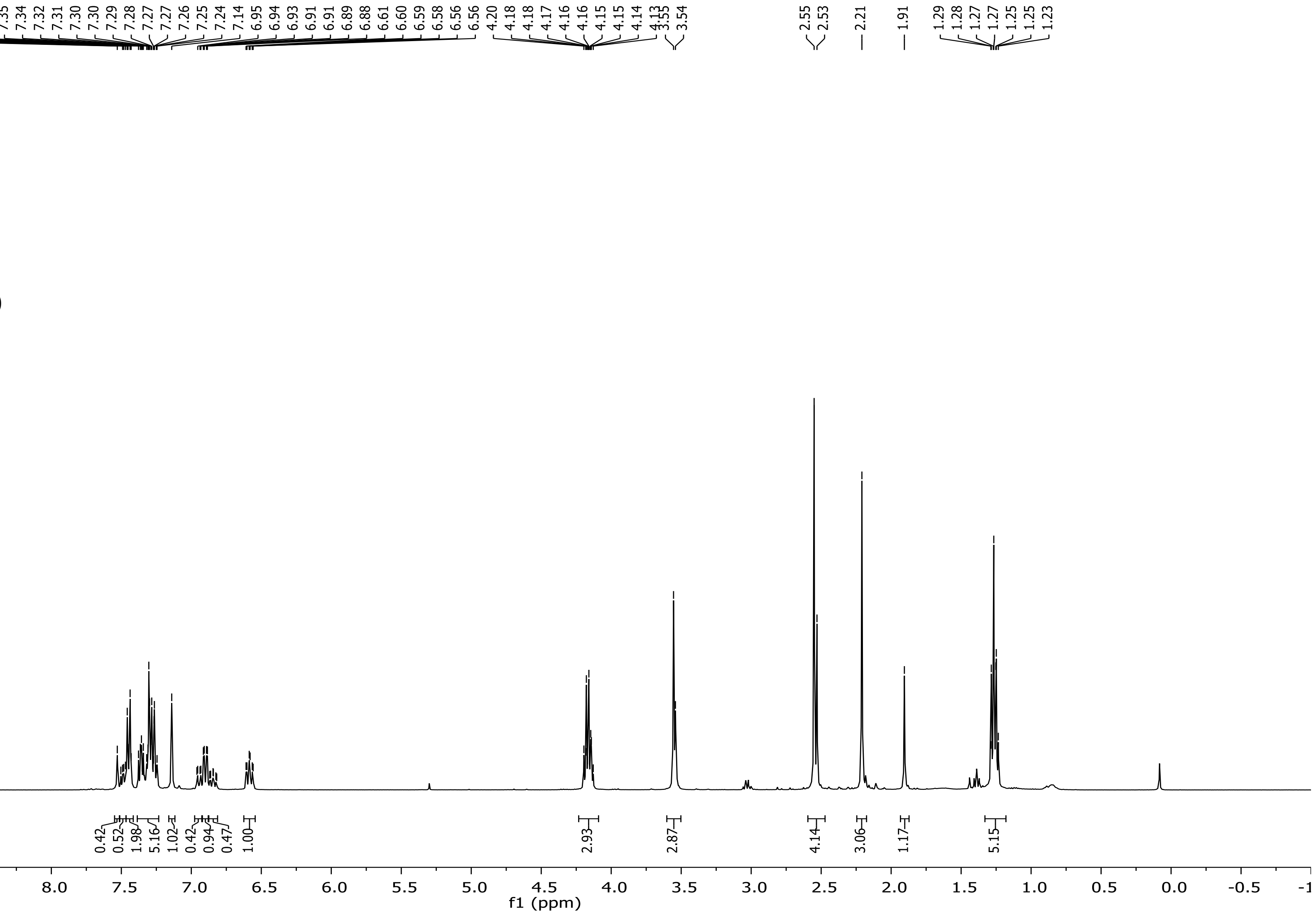

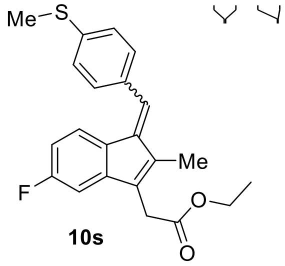

10s

^{13}C-NMR (100 MHz, $CDCl_3$)

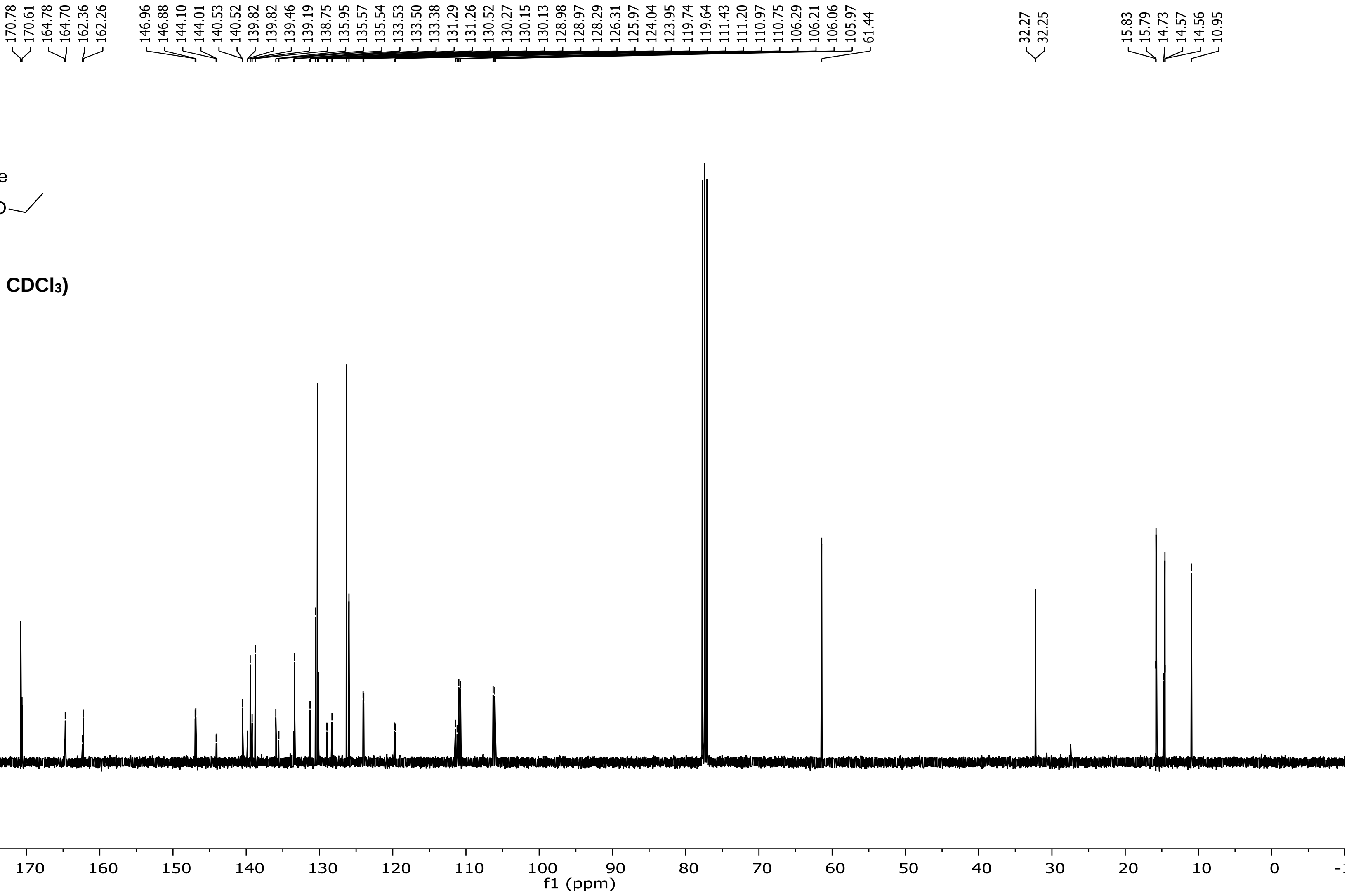

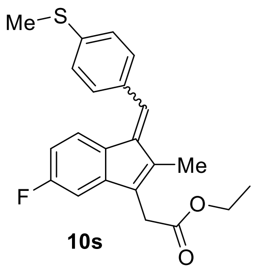

10s

^{19}F-NMR (376 MHz, CDCl₃)

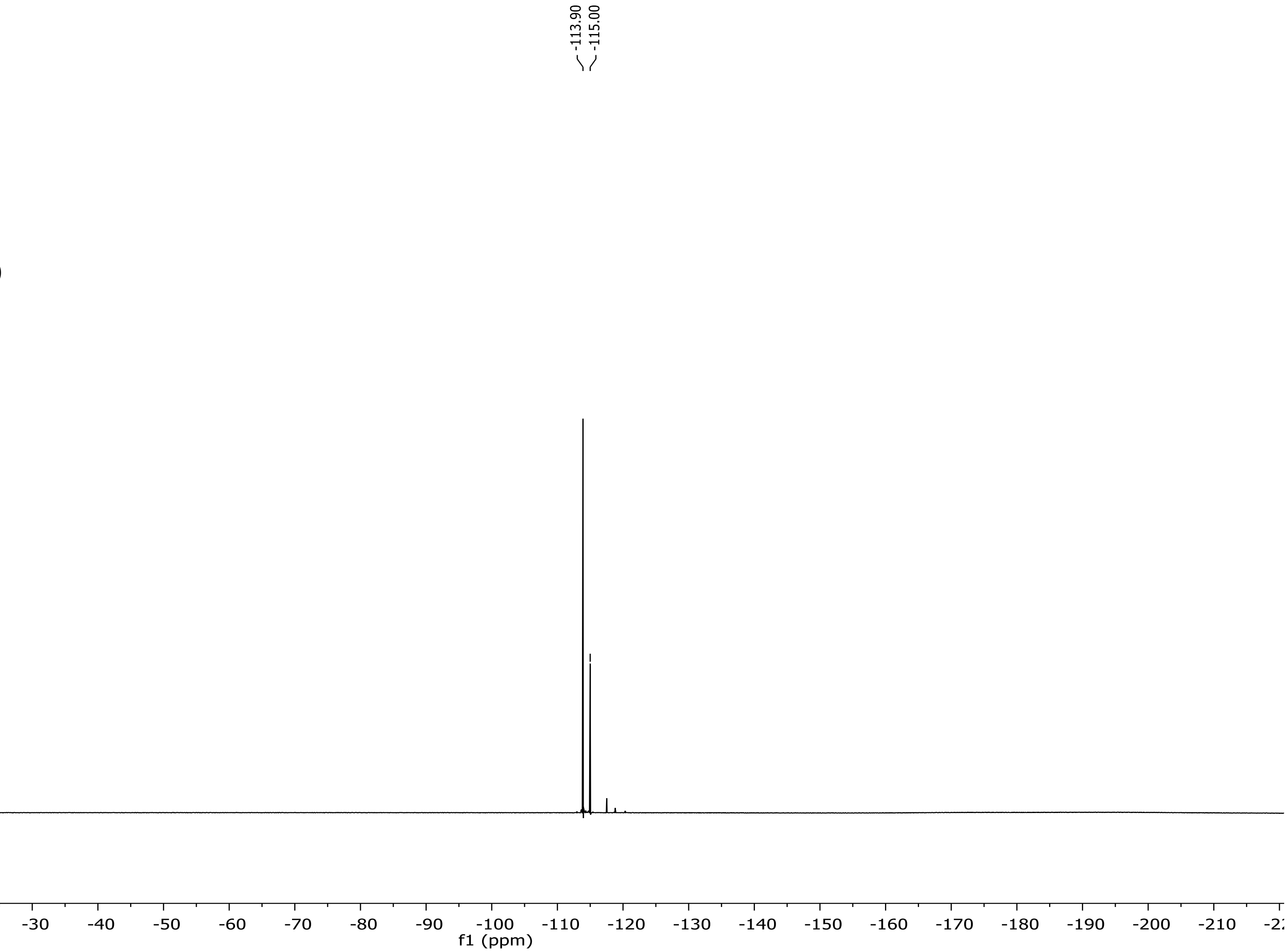

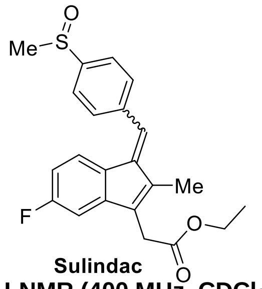

Sulindac

^{1}H-NMR (400 MHz, CDCl$_3$)

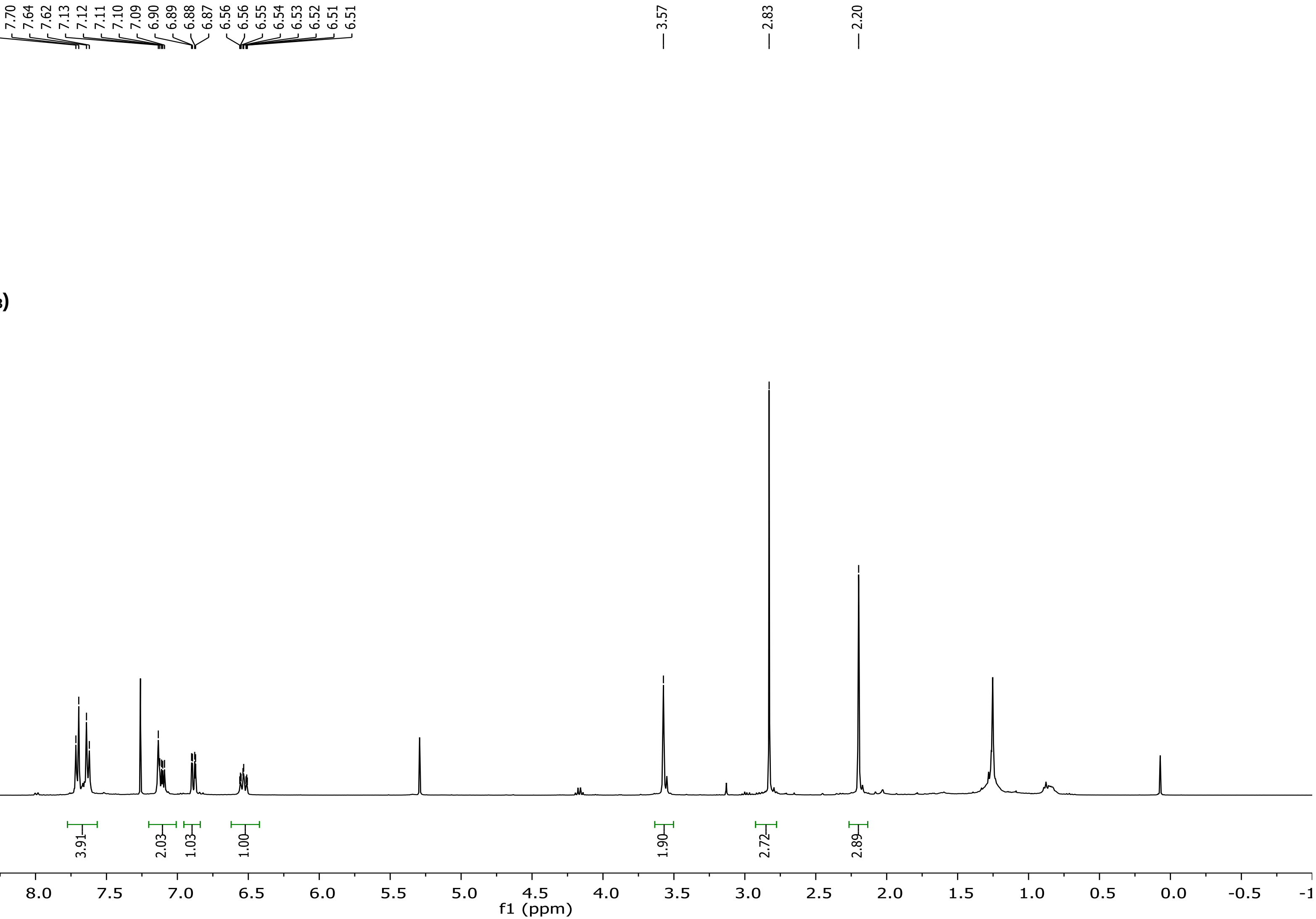

Supplement: Supplementary file 1 — ol4c02092_si_001.pdf [file ol4c02092_si_001.pdf]
